# Association between 19 medication use and risk of common cancers: A cross-sectional and Mendelian randomisation study

**DOI:** 10.7189/jogh.14.04057

**Published:** 2024-03-15

**Authors:** Zhangjun Yun, Yang Shen, Xiang Yan, Shaodan Tian, Jing Wang, Chiah Shean Teo, Hongbin Zhao, Chengyuan Xue, Qing Dong, Li Hou

**Affiliations:** 1 Department of Oncology and Haematology, Dongzhimen Hospital, Beijing University of Chinese Medicine, Beijing, China; 2 First School of Clinical Medicine, Beijing University of Chinese Medicine, Beijing, China; 3 School of Traditional and Complementary Medicine, Faculty of Medicine and Health Sciences, UCSI University, Kuala Lumpur, Malaysia

## Abstract

**Background:**

Previous studies have yielded inconsistent results concerning drug use and the risk of cancers. We conducted a large-scale cross-sectional study and a two-sample Mendelian randomisation (MR) study to reveal the causal effect between the use of 19 medications and the risk of four common cancers (breast, lung, colorectal, and prostate).

**Methods:**

We obtained information on medication use and cancer diagnosis from National Health and Nutrition Examination Survey participants. After propensity score matching, we conducted survey-weighted multivariate logistic regression and restricted cubic spline analysis to assess the observed correlation between medication use and cancer while adjusting for multiple covariates. We also performed MR analysis to investigate causality based on summary data from genome-wide association studies on medication use and cancers. We performed sensitivity analyses, replication analysis, genetic correlation analysis, and reverse MR analysis to improve the reliability of MR findings.

**Results:**

We found that the use of agents acting on the renin-angiotensin system was associated with reduced risk of prostate cancer (odds ratio (OR) = 0.42; 95% confidence interval (CI) = 0.27–0.63, *P* < 0.001), and there was a nonlinear association of ‘decrease-to-increase-to-decrease’ (*P* < 0.0001). The random-effects inverse variance weighted (IVW) model-based primary MR analysis (OR = 0.94, 95% CI = 0.91–0.97, *P* = 0.0007) and replication MR analysis (OR = 0.90, 95% CI = 0.85–0.96, *P* = 0.0006) both provided robust evidence of the causality of genetic liability for the use of agents acting on the renin-angiotensin system on a decreased risk of prostate cancer.

**Conclusions:**

Our study provides robust evidence that the use of drugs acting on the renin-angiotensin system can reduce prostate cancer risk. Given the high prevalence of prostate cancer, these findings have important implications for drug selection and prostate cancer prevention in patients with cardiovascular disease.

According to the latest cancer statistics, an estimated 19.3 million new cancer cases and 10 million cancer-related deaths occurred around the world in 2020 [1]. Breast, lung, colorectal, and prostate cancer have become the most prevalent cancers worldwide. Cancer has been identified as the primary or secondary cause of mortality in individuals under the age of 70 years old in a total of 112 countries. Alarmingly, the global burden of cancer is expected to increase by approximately 50% over the next two decades [1]. Given the taxing burden that cancer imposes on global health care systems, economies, and social progress, the timely implementation of cancer screening and prevention measures has become an urgent objective.

Everyone inevitably encounters situations where they use or depend on medications. However, prescription drugs, while being administered for their respective indications, may also increase or decrease the risks of other ailments, especially for individuals with a history of pharmaceutical addiction. Although clinical trials provide evidence to support the safety of these drugs in the short term, the risks associated with long-term use of the drugs are unknown. Considering the significant global burden of disease associated with cancer, exploring drug use and cancer risk is not only beneficial for screening high-risk populations but also contributes to clinical drug selection and the discovery of potential cancer targets. However, previous studies have produced inconsistent conclusions regarding the relationship between commonly prescribed medications and the risk of cancer incidence. For example, hypertension affects approximately 1.5 billion people worldwide, which requires the lifetime use of antihypertensive agents in most cases [2]. A meta-analysis [3] of five randomised controlled trials (RCTs) encompassing 61 590 participants with at least one year of follow-up suggested that participants receiving angiotensin-receptor blockers (ARBs) therapy had a significantly higher risk of new cancers. However, another meta-analysis [4], including 70 RCTs covering 324 168 participants with at least one year of follow-up, found no evidence of increased cancer risk with ARBs. Similarly, the results of a meta-analysis by Yoon et al. [5] of 12 cohort studies and 16 case-control studies published in MEDLINE, Embase, and the Cochrane Library before January 2011 did not find an association between angiotensin-converting enzyme inhibitors (ACEIs) or ARBs and cancer risk. Only meta-analysis of cohort and nested case-control studies or studies with a long-term follow-up of more than five years found evidence that the use of ACEIs or ARBs was significantly associated with reducing cancer risk [5]. A subgroup analysis excluding traditional case-control studies found a reduced risk of prostate cancer [5].

Studies in recent years have shown greater support for ACEIs or ARBs in reducing the risk of cancer in terms of biological mechanisms. For example, angiotensin II, a major signalling molecule of the renin-angiotensin system, can promote tumour growth by inducing angiogenesis and tumour cell proliferation through increased expression of vascular endothelial growth factor (VEGF) or epidermal growth factor receptor (EGFR) [6–8]. Angiotensin II can also stimulate cell growth and proliferation through the transforming growth factor-β [9], tyrosine kinase [10], and mechanistic target of rapamycin (mTOR) pathways [11]. Uemur et al. also found that ARBs can inhibit prostate cancer cell proliferation by suppressing epidermal growth factor (EGF) expression and mitogen-activated protein kinases (MAPK) and signal transducer and activator of transcription 3 (STAT3) phosphorylation pathways induced by angiotensin II [12]. In addition, antidepressants are also among the most prescribed medications in Western countries, with more than 10% of Americans using antidepressants at least once a year [13]. A nested case-control study involving 7767 cases of prostate cancer diagnosed between 1981 and 2000 at the Saskatchewan Cancer Agency revealed that the use of tricyclic antidepressants was associated with an increased risk of prostate cancer [14]. Another nested case-control study of 1151 cases of prostate cancer based on a nationwide insurance claim database indicated that there was no association between mechanistically different antidepressants and an increased hazard of prostate cancer [15]. Differences in the findings of these clinical studies may be explained by study design, follow-up time, and control for confounders. In particular, traditional observational studies have struggled to clarify the causal association between drug use and cancer risk due to methodological shortcomings. Given the inconsistent evidence on the association between drug use and cancer and the fact that cancer has become a major health problem for the global population, revealing the potential impact of drug use on cancer is of great value to public health.

The National Health and Nutrition Examination Survey (NHANES) is a cross-sectional study of health, nutrition, and potential risk factors among non-institutionalised residents of the USA under the age of 85 years [16]. Over recent years, the NHANES has been used by researchers and federal agencies around the world. The questionnaire in the NHANES covers information on participant medication use and cancer diagnosis. Therefore, the NHANES database consists of high-quality cohort data from the USA to assess the relationship between drug use and cancer. However, observational studies can only indicate correlations between characteristics of interest but cannot prove causality. Although RCTs are the gold standard for demonstrating causality, they are difficult to implement on this topic due to the large number of human resources required and the long duration of follow-up.

To address methodological gaps, we conducted a Mendelian randomisation (MR) study to investigate the causality between drug use and cancer. In recent years, MR has been widely employed to investigate the causality between an exposure of interest and disease [17]. MR using single nucleotide polymorphisms (SNP) as unconfounded instrumental variables (IVs) for exposure of interest provides an effective means of circumventing residual confounding and reverse causality, which commonly affects conventional observational studies [18]. The exposure factors measured in observational studies are often biased due to the influence of behavioural, social, and psychological factors, whereas such factors do not influence genetic variations. In the absence of RCTs, MR has emerged as a critical strategy for inferring causal relationships, as human genetic inheritance follows Mendelian genetics, where alleles segregate independently, and nonallelic genes are freely and randomly transmitted to offspring, thereby mimicking the process of RCTs.

This study aimed to quantify the relationship between 19 commonly prescribed medications and four common cancers (breast, lung, colorectal, and prostate) using the NHANES and to assess the causal effect via MR analysis. To our knowledge, this is the first work to date to comprehensively assess prescription drugs and cancer risk by combining large cross-sectional studies and MR analysis. For the 19 medication categories (Table S1 in the **Online Supplementary Document**), we referred to the study by Wu et al. and used the Anatomical Therapeutic Chemistry (ATC) classification system to classify the drugs [19].

## METHODS

### Ascertainment of medication use and cancers in the NHANES database

**Figure 1:**
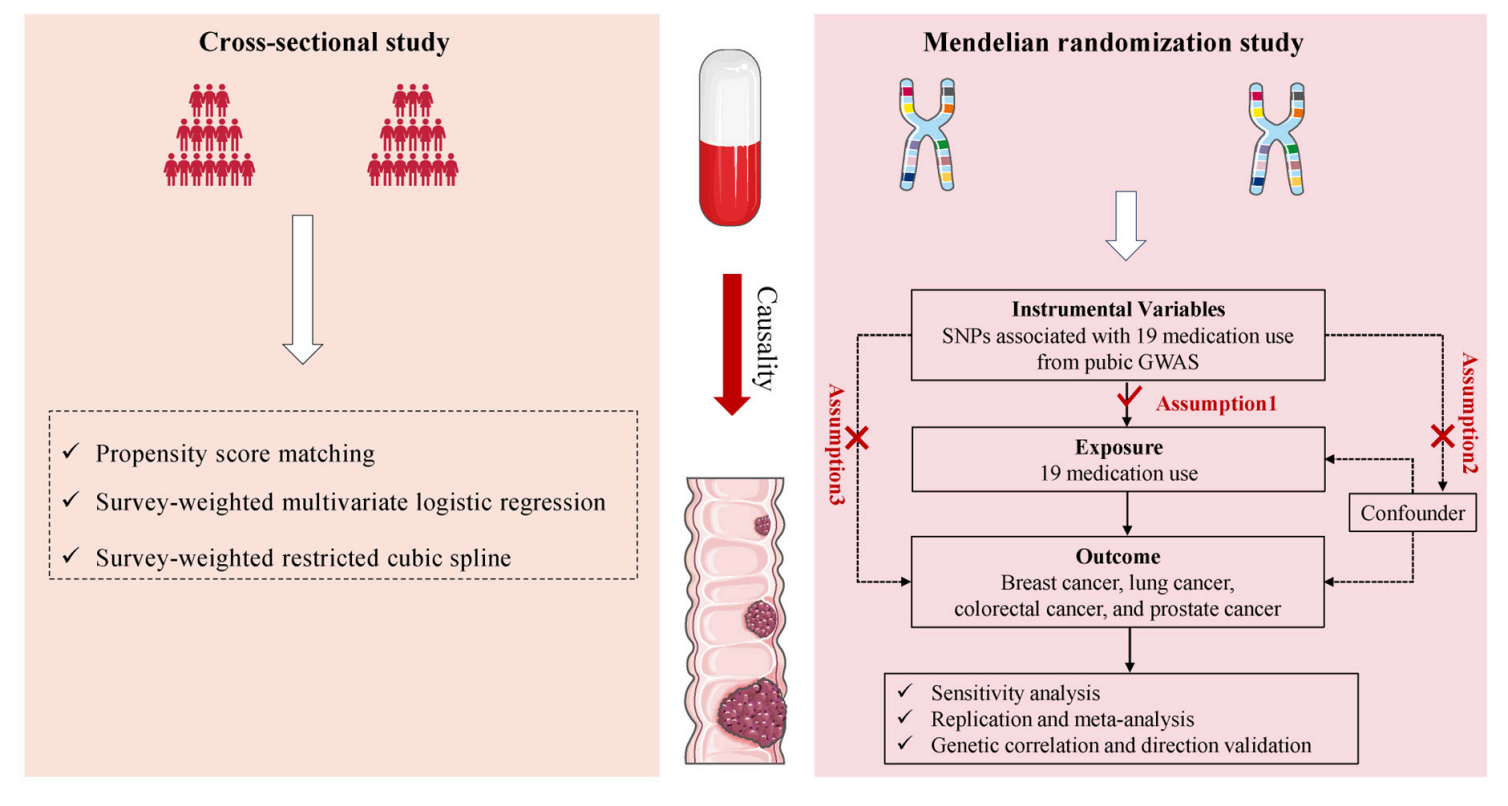
Outline of the study design. SNPs – single nucleotide polymorphisms, GWAS – genome-wide association studies

An overview diagram of the research design for this study is shown in **Figure 1**. We extracted information on drug use and cancer diagnosis from the NHANES from 1999–2022, which was obtained by questionnaires. The use of medications was determined by participants who reported the name of the medication, the duration of taking each drug, and whether they took prescription medications in the last month (Table S2 in the **Online Supplementary Document**). The type of cancer and the time to cancer diagnosis were determined by self-reporting by the participants. Medication use was defined as medication initiation at least six months before cancer diagnosis and use of current medication in the last month at the time of the survey. Participants with missing data were excluded.

#### Assessment of covariates

We extracted age, sex, body mass index (BMI), race/ethnicity, educational attainment, family income, drinking status, and smoking status collected by the questionnaire as covariates [20,21]. This study analysed adults who were 20 years and older and had completed the data. The BMI was classified as underweight (<18.5 kg/m^2^), normal weight (18.5–25 kg/m^2^), overweight (25–30 kg/m^2^), and obese (≥30 kg/m^2^). Race/ethnicity was classified as Mexican American, other Hispanic, non-Hispanic white, non-Hispanic black, and other/multiracial. Educational attainment was grouped into less than high school, high school, and more than high school. Family income classification is based on the ratio of family income to the federal poverty level (<1.3, 1.3–3.5, >3.5). Higher income-poverty ratios imply a higher state of household income. Those who had smoked fewer than 100 cigarettes in their lifetime were defined as never smokers; those who had smoked more than 100 cigarettes but had not smoked at the time of the survey were defined as former smokers; and those who had smoked more than 100 cigarettes in their lifetime and had smoked at the time of the survey were defined as current smokers. Alcohol use was categorised according to the number of drinks per month as never drink, 1–5 drinks per month, 5–10 drinks per month, and more than 10 drinks per month.

#### Statistical analysis in the NHANES study

Given the complex sampling design of NHANES, all analyses in this study considered sample weights, clustering, and stratification. The Mobile Examination Centre exam weights (WTMEC4YR, WTMEC2YR, WTMECPRP) were used to calculate the new sample weights to generate a nationally representative estimate. To create a comparable control group and minimise the interference of confounders, propensity score matching (PSM) strategies were applied. Specifically, confounders (age, sex, BMI, race, educational attainment, family income, drinking status, and smoking status) were used as covariates to calculate individual propensity scores via logistic regression modelling. For medication-using participants, a 1:4 nearest-neighbour match and a calliper value of 0.05 were used to select a match that was not treated with that medication. PSM can better explain significant differences in covariates between the two groups. The survey-weighted multivariate logistic regression analysis correcting for covariate interference was then used to explore the linear association between medication use and four site-specific cancers. Survey-weighted restricted cubic spline (RCS) was applied to assess the nonlinear relationship between days of medication use and the risk of cancer. Continuous variables are expressed as weighted mean and standard deviations, and categorical variables are displayed as unweighted frequencies and weighted percentages. The χ^2^ and t-test were used to compare categorical and continuous variables between groups. All *P*-values were two-tailed; *P* < 0.05 was considered nominally significant, and *P* < 0.0026 (Bonferroni correction *P* = 0.05/19) was considered a significant association. All statistical analyses were performed using R, version 4.3.1 (R Core Team, Vienna, Austria). Packages ‘MatchIt 4.5.5′, ‘gtsummary 1.7.2’, ‘survey 4.2-1’, and the ‘rms 6.7-0’ were used to perform PSM, survey-weighted generalised logistic regression, and RCS. The number of knots for the RCS analysis was set to four.

#### MR analysis

We have quantified the relationship between 19 drug use and cancer in the NHANES study. Then, we performed a two-sample MR study based on large-scale genome-wide association studies (GWAS) to assess the potential causality. A convincing MR study should adhere to three key assumptions: i) the IVs are significantly associated with the exposure of interest, ii) the IVs are independent of confounding factors and solely affect the outcome via the exposure of interest, iii) the IVs are not directly related to the outcome [22]. All MR analyses in this investigation were conducted using R software, employing the ‘two-sample MR 0.5.7’, ‘Mendelian randomisation 0.7.0’, ‘MRPRESSO 1.0’, and ‘radial MR 1.1’ packages. This study followed the Strengthening the Reporting of Observational Studies in Epidemiology-Mendelian randomisation reporting guidelines (Table S3 in the **Online Supplementary Document**) [23].

#### GWAS data for medication use

Summary data for 19 medication categories were obtained from a GWAS case-control meta-analysis of self-reported medication use from 320 000 participants of the UK Biobank by Wu et al. [19]. Specifically, this study was conducted through nurse-led interviews with participants, and only regular medications taken weekly, monthly, or for three months were recorded based on the self-reports of the participants, but the duration and dose of medication taken were not obtained [24]. Wu et al. coded all drug use for participants, and there were 1809 medication categories with at least 10 participants that were manually assigned to their corresponding active ingredients using the online database (mainly Electronic Medicines Compendium, Drugs, and NetDoctor) and classified using the ATC classification system [25]. Categories named according to their active ingredients were directly mapped to ATC codes. Together with data on drug use in the NHANES, only 19 medications were used in this study. The specific active ingredient and ATC code information for the 19 medication categories are shown in Table S4 in the **Online Supplementary Document**. During the initial visit for the UK Biobank assessment, roughly 54% of the participants were women. The mean age of the participants was 56.53 years (standard deviation (SD) = 8.09), and the mean BMI was 27.43 (SD = 4.80) [19]. The number of cases with the use of 19 medications ranged from 3954 to 93 218 (**Table 1**).

**Table 1:** Summary of 19 medication use GWAS

Medication category	Medication-taking traits	Cases (n)	Control (n)	Total (n)
A02B	Drugs for peptic ulcer and GORD	53 137	79 230	132 367
A10	Drugs used in diabetes	15 272	290 641	305 913
B01A	Antithrombotic agents	67 653	85 986	153 639
C01D	Vasodilators used in cardiac diseases	5546	237 113	242 659
C02	Antihypertensives	6431	145 949	152 380
C03	Diuretics	34 453	194 633	229 086
C07	Beta blocking agents	31 700	192 324	224 024
C08	Calcium channel blockers	31 904	172 474	204 378
C09	Agents acting on the renin-angiotensin system	62 752	174 778	237 530
C10AA	HMG CoA Preductase inhibitors	73 475	216 910	290 385
H03A	Thyroid preparations	24 832	280 750	305 582
L04	Immunosuppressants	3954	268 648	272 602
M01A	Anti-inflammatory and antirheumatic products, non-steroids	74 150	90 370	164 520
M05B	Drugs affecting bone structure and mineralisation	7870	207 798	215 668
N02A	Opioids	22 982	55 826	78 808
N02C	Antimigraine preparations	5521	114 323	119 844
N06A	Antidepressants	33 757	270 405	304 162
R03BA	Glucocorticoids	17 352	188 348	205 700
R06A	Antihistamines for systemic use	13 984	137 652	151 636

GORD – gastroesophageal reflux disease, GWAS – genome-wide association studies, HMG CoA – 3-hydroxy-3-methylglutaryl coenzyme A

#### GWAS data for cancers

We performed primary MR analysis using large-scale GWAS data for four site-specific cancers as our outcome. The GWAS data for breast cancer were obtained from a meta-analysis conducted by the Breast Cancer Association Consortium and included 122 977 patients with breast cancer [26]. Regarding lung cancer, data from the GWAS of 11 348 cases of lung cancer conducted by the International Lung Cancer Consortium were used [27]. Summary data for colorectal cancer were obtained from a meta-analysis of GWAS studies conducted by Huyghe et al., which included 19 938 cases of colorectal cancer [28]. GWAS data for 79 148 cases of prostate cancer were obtained from the Prostate Cancer Association Group to Investigate Cancer-Associated Alterations [29]. GWAS data for breast cancer, lung cancer, and prostate cancer were obtained from the OpenGWAS project database, and data for patients with colorectal cancer were downloaded from the GWAS catalogue. Detailed GWAS information for the four site-specific cancers is shown in Table S5 in the **Online Supplementary Document**. All GWAS data in this study were derived from European populations.

#### Selection of IVs for MR

To comply with three assumptions of MR, we performed a series of rigorous quality controls on the selection of genetic variants for the exposure of interest. To satisfy the first hypothesis, we extracted SNPs strongly associated with the use of medications at the threshold of *P *< 5 × 10^−8^. Subsequently, based on the 1000 Genomes European reference panel [30], we calculated the linkage disequilibrium between selected SNPs and clumped SNPs by removing the linkage disequilibrium (LD; *R^2 ^*< 0.001 within a window of 10 000 kb) to ensure the independence of the SNPs. To avoid bias from weak IVs, we quantified the strength of genetic variation by calculating *R^2^* and *F* statistics [31,32]:



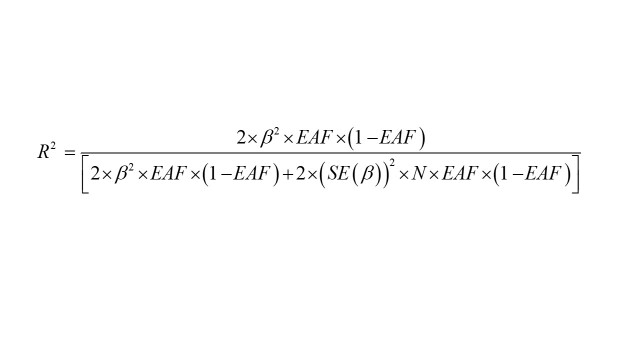





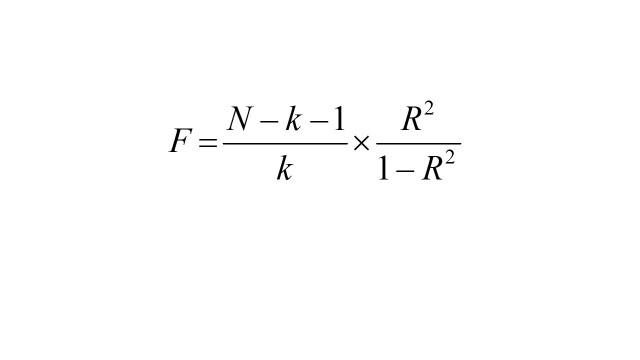



where *β* is the effect size for the genetic variation of medication use; EAF is the frequency of the effect allele for the genetic variation of medication use; SE (*β*) is the standard error of the effect size for the genetic variation of medication use; and *R^2^* is the instrumental variable that explains the degree of medication use (determinant coefficient of the regression equation). N is the sample size of the medication use; k is the number of SNPs (instrumental variants). SNPs with *F*-statistics less than 10 were excluded due to their insufficient strength. After filtering by the above-mentioned conditions, the SNPs were considered as IVs instead of 19 medication use (Table S6 in the **Online Supplementary Document**).

To comply with the third hypothesis, IVs strongly associated (*P* < 5 × 10^−8^) with the four site-specific cancers were discarded. If SNPs for exposure were unavailable in the outcome data, we searched for proxy SNPs based on the 1000 Genomes European reference panel at the threshold of LD *R^2 ^*> 0.8 [30]. We discarded those SNPs that were unavailable in the outcome while no appropriate substitutes were accessible. Finally, we harmonised SNPs for exposure and outcome, and non-concordant alleles and palindromic SNPs with ambiguous strands that could not be corrected were removed. We performed radial MR to identify and remove potential outliers for significant associations to obtain more reliable causal estimates [33]. Furthermore, SNPs that were significantly associated with cancer confounders (*P *< 5 × 10^−8^), such as BMI, educational level, income level, smoking status, and alcohol consumption were also removed (checked on the PhenoScanner website to meet the second hypothesis. After radial MR analysis and confounder scanning, we assessed significance associations to determine final causal effects.

#### Statistical analysis in MR

For MR analysis, when IVs ≥ 2, SNPs were available, the random-effects inverse variance weighted (IVW) method was used as the primary analysis, and four additional MR models were used as complementary analyses, including MR-Egger, weighted median, maximum likelihood, and robust adjusted profile score (RAPS). For IVs with only one SNP, causal effects were assessed according to the Wald ratio test. IVW is a meta-analysis of the Wald ratio estimates for each SNP to obtain consistent estimates of the causal effect of exposure on the outcome. IVW provided the most accurate estimates of causality in the absence of horizontal pleiotropy for all SNPs [34]. The weighted median allows less than 50% of genetic variation to be invalid, while MR-Egger allows for horizontal pleiotropy of all genetic variation and tests for pleiotropy and heterogeneity of MR estimates [34,35].

Simultaneously, MR-Egger regression can provide unbiased estimates when IVs meet the InSIDE hypothesis (strength of IVs independent of direct effects) [35]. The maximum likelihood model is similar to IVW, providing unbiased estimates under the assumption of no heterogeneity and horizontal pleiotropy in IVs, and with smaller standard errors than IVW [36]. Additionally, maximum likelihood takes adequate account of the uncertainty in the association of SNPs with exposure and considers the sample overlap in two-sample MR [37]. As an extension of IVW, RAPS allows for relatively weak genetic variation. RAPS can provide accurate estimates in the presence of weak IVs [38]. We defined that IVW-derived *P* < 0.05 was considered nominally significant, and IVW-derived *P* < 0.0026 (Bonferroni correction *P* = 0.05/19) was considered a significant association.

Sensitivity analyses were performed to assess whether the causal estimates violated the MR assumptions. We performed sensitivity analyses with the Cochran Q-test, MR-Egger intercept test, MR-Pleiotropy RESidual sum and outlier (MR-PRESSO), and leave-one-out (LOO) analysis. The heterogeneity of the IVW model was assessed using the Cochran Q-test [39]. Cochran Q test-derived *P* < 0.05 was considered heterogeneity of the MR estimates. Excessive heterogeneity suggested violating modelling assumptions or incorporating invalid SNPs that would induce horizontal pleiotropy [40]. The MR-Egger intercept was implemented to detect horizontal pleiotropy and bias due to invalid SNPs [35]. MR-PRESSO was performed to recheck outliers and heterogeneity. The robustness of the MR estimates was assessed on the basis of LOO analysis to detect whether a single SNP drove the MR estimates. The online tool mRnd was used to assess the statistical power of the sample and calculate the results. For the identified significant associations, reverse MR was performed to exclude reverse causal effects.

Significant associations between medication use and cancer were established by several criteria: a) IVW-derived *P* < 0.05, b) the directionality of the five MR models was consistent, c) there was no heterogeneity or horizontal pleiotropy in the MR estimates, d) the MR results were not seriously confounded by single SNP.

#### Replication and meta-analysis for MR analysis

To further examine our ﬁndings, replication analysis was performed using GWAS data for cancer from the FinnGenR9 Consortium (Table S7 in the **Online Supplementary Document**) [41]. Specifically, a meta-analysis was performed on the random-effects multiplicative IVW estimates from primary analysis and replication analysis to verify the accuracy of the causal effect. A meta-analysis was implemented based on Review Manager (RevMan), version 5.4.1 (Cochrane, London, UK), with heterogeneity greater than 50% using the IVW random-effects model and otherwise using the IVW fixed-effects model.

#### Genetic correlation and direction validation

Previous studies have found that MR results are likely to produce false positives when a genetic correlation exists between two traits [42]. Although SNPs associated with outcome (cancer) have been discarded during the selection of genetic proxies for exposure, SNPs not significantly associated with cancer may also mediate the genetic risk of cancer. Therefore, for the identified significant MR estimates, we estimated the heritability and genetic correlation between drug use and cancer using the linkage disequilibrium score regression (LDSC) method to assess whether MR findings are influenced by shared genetics [43].

Furthermore, consistent with the previous strategy for the MR study, reverse MR analysis was performed for significant associations to detect potential reverse causality. We also performed the Steiger test to ensure the accuracy of the association release between drug use and cancer. The results were considered statistically significant at *P* < 0.05.

## RESULTS

### Baseline characteristics of the participants in the NHANES study

The NHANES study included 30 899 participants. There were significant differences in numerous baseline characteristics between participants with cancer and participants without cancer. Compared to non-cancer participants, participants with cancer were characterised by being older, more likely to be female and non-Latino whites, more often educated beyond high school, and most often part of middle or high-income families. A high percentage of participants with cancer smoked and consumed alcohol more than 10 times a month (**Table 2**). The characteristics of the 19 medications used in the NHANES study are shown in Table S8 in the **Online Supplementary Document**.

**Table 2:** Sample characteristics in the NHANES population 1999–2022*

Characteristics		Overall (n = 30 899)		Cancer (n = 2938)	No cancer (n = 27 961)	*P*-value
Age in years, mean (SD)	49.32 (18.02)		65.46 (14.26)		47.63 (17.53)	<0.001
Sex						0.039
*Female*	15 742 (50.9)		1550 (52.8)		14 192 (50.8)	
*Male*	15 157 (49.1)		1388 (47.2)		13 769 (49.2)	
Race						<0.001
*Mexican American*	4951 (16.0)		172 (5.9)		4779 (17.1)	
*Non-Hispanic black*	6414 (20.8)		406 (13.8)		6008 (21.5)	
*Non-Hispanic white*	14 335 (46.4)		2104 (71.6)		12 231 (43.7)	
*Other Hispanic*	2564 (8.3)		151 (5.1)		2413 (8.6)	
*Other/multiracial*	2635 (8.5)		105 (3.6)		2530 (9.0)	
BMI						0.144
*Underweight*	468 (1.5)		48 (1.6)		420 (1.5)	
*Normal*	8593 (27.8)		794 (27.0)		7799 (27.9)	
*Overweight*	10 312 (33.4)		1034 (35.2)		9278 (33.2)	
*Obese*	11 526 (37.3)		1062 (36.1)		10 464 (37.4)	
Educational attainment						<0.001
*Less than high school*	7691 (24.9)		645 (22.0)		7046 (25.2)	
*High school*	7150 (23.1)		662 (22.5)		6488 (23.2)	
*More than high school*	16 058 (52.0)		1631 (55.5)		14 427 (51.6)	
Family income						<0.001
*Low*	9599 (31.0)		708 (24.1)		8891 (31.8)	
*Middle*	11 732 (38.0)		1208 (41.1)		10 524 (37.6)	
*High*	9568 (31.1)		1022 (34.8)		8546 (30.6)	
Smoking status						<0.001
*Never smokers*	16 622 (53.8)		1281 (43.6)		15 341 (54.9)	
*Former smokers*	7723 (25.0)		1195 (40.7)		6528 (23.3)	
*Current smokers*	6554 (21.2)		462 (15.7)		6092 (21.8)	
Alcohol use						<0.001
*Non-drinker*	8866 (28.7)		891 (30.3)		7975 (28.5)	
*1–5 drinks/mo*	15 270 (49.4)		1365 (46.5)		13 905 (49.7)	
*5–10 drinks/mo*	2395 (7.8)		178 (6.1)		2217 (7.9)	
*10 and more drinks/mo*	4361 (14.1)		503 (17.1)		3858 (13.8)	
*Unknown*	7 (0.0)		1 (0.0)		6 (0.0)	
Cancer						
*Yes*	2938 (9.5)					
*No*	27 961 (90.5)					
Breast cancer						
*Yes*	464 (1.5)					
*No*	30 435 (98.5)					
Lung cancer						
*Yes*	77 (0.2)					
*No*	30 822 (99.8)					
Colorectal cancer						
*Yes*	222 (0.7)					
*No*	30 677 (99.3)					
Prostate cancer						
*Yes*	469 (1.5)					
*No*	30 430 (98.5)					

BMI – body mass index, NHANES – the National Health and Nutrition Examination

*Presented as n (%) unless specified otherwise.

#### Association between the use of medication and cancers in the NHANES database

**Figure 2:**
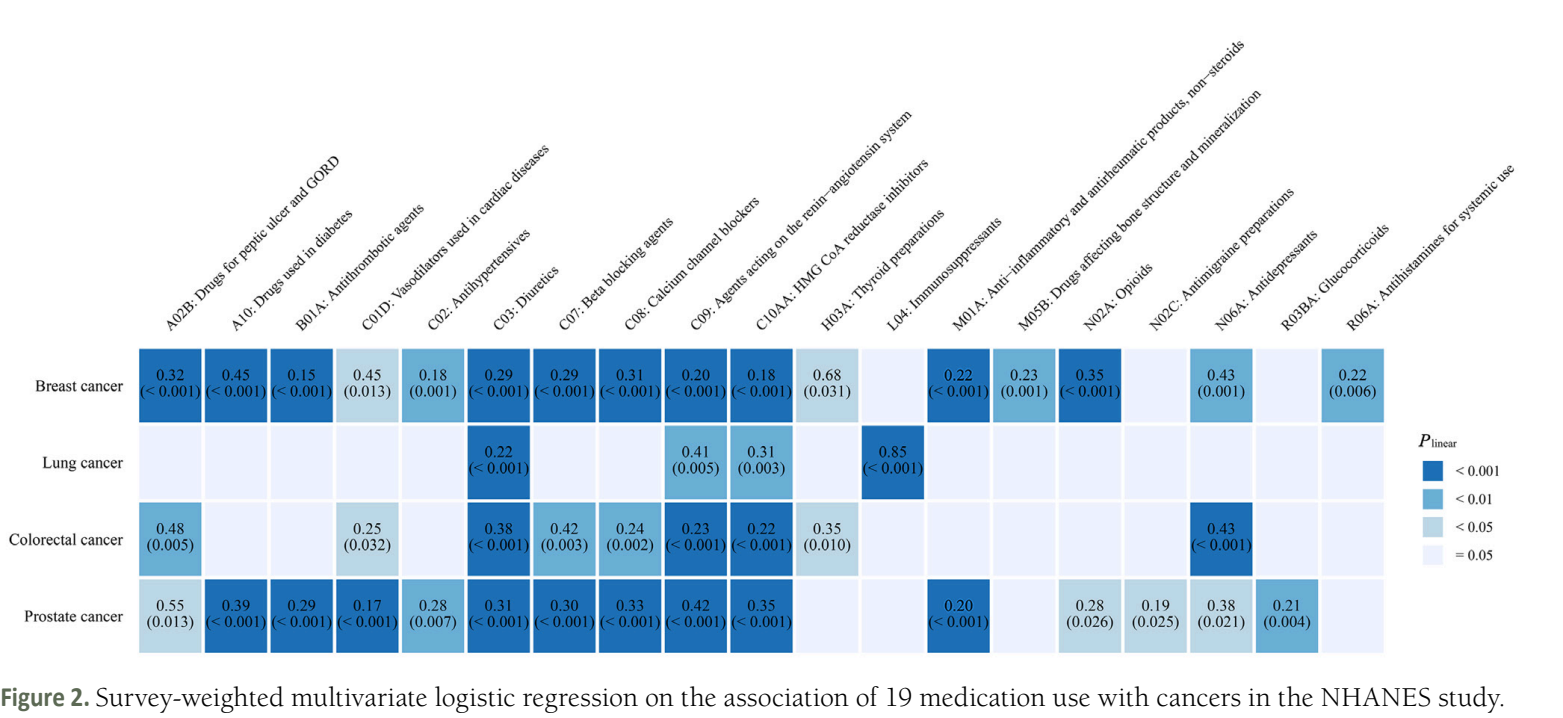
Survey-weighted multivariate logistic regression on the association of 19 medication use with cancers in the NHANES study. All models were adjusted for age, sex, BMI, race, educational attainment, family income, drinking status, and smoking status. Only the findings with significant association (*P* < 0.05) are shown in the figure. The colour of each square is related to the significance of the association, and the legend on the right is used as a reference. The number above the box is the odds ratio, and the *P*-value is displayed in parentheses below. BMI – body mass index, NHANES – the National Health and Nutrition

After controlling for covariates between the two groups through PSM, the survey-weighted multivariate logistic regression analysis showed a reduced risk of four site-specific cancers in those with partial medication use compared with those without medication use (**Figure 2****,** Tables S9–12 in the **Online Supplementary Document**).

To explore the potential nonlinear relationship between the duration of drug use and cancer risk, we estimated the correlation using RCS analysis. The results showed nonlinear v-shaped significant associations between the duration of use of drugs for peptic ulcer and gastroesophageal reflux disease (GORD), drugs used in diabetes, antithrombotic agents, vasodilators used in cardiac diseases, diuretics, beta-blocking agents, calcium channel blockers, agents acting on the renin-angiotensin system, 3-hydroxy-3-methylglutaryl coenzyme A (HMG CoA) reductase inhibitors, thyroid preparations, anti-inflammatory and antirheumatic products, non-steroids, drugs affecting bone structure and mineralisation, antidepressants, glucocorticoids, and antihistamines for systemic use and the risk of breast cancer (*P* < 0.0026) (Figure S1 in the **Online Supplementary Document**).

The duration of antihypertensive medication administration showed a v-shaped nonlinear nominal significant association with breast cancer risk, but immunosuppressants showed an s-shaped nonlinear nominal significant association (*P* < 0.05). For lung cancer, the vasodilators’ duration in cardiac diseases and calcium channel blockers showed significant nonlinear v-shaped associations (*P* < 0.0026). Duration of drugs for peptic ulcer and GORD, beta-blocking agents, and agents acting on the renin-angiotensin system and lung cancer presented a v-shaped nonlinear nominal significant association (*P* < 0.05). For colorectal cancer, we observed two significant patterns of nonlinear associations (*P* < 0.0026), including s-shaped for the duration of drugs used in diabetes, beta-blocking agents, HMG CoA reductase inhibitors, and glucocorticoids, and l-shaped for the duration of agents acting on the renin-angiotensin system. We also observed a nominally significant s-shaped association between the duration of use of drugs for peptic ulcer and GORD and diuretics and the risk of colorectal cancer (*P* < 0.05). S-shaped associations of the duration of vasodilators used in cardiac diseases, anti-inflammatory and antirheumatic products, non-steroids, and antidepressants used with the risk of colorectal cancer were observed (*P* < 0.05). In terms of prostate cancer, we also discovered two significant patterns of nonlinear associations (*P* < 0.0026), including s-shaped for the duration of drugs used in diabetes, antithrombotic agents, beta-blockers and agents acting on the renin-angiotensin system, and v-shaped for the duration of drugs for peptic ulcer and GORD, drugs for peptic ulcer and GORD and HMG CoA reductase inhibitors (*P* < 0.0026). Nonlinear nominal significant associations of s-shaped and v-shaped were observed in the duration of antihypertensives, calcium channel blockers, and prostate cancer (Figures S1–4 in the **Online Supplementary Document**).

#### Causality between medication use and cancers in the MR analysis

**Figure 3:**
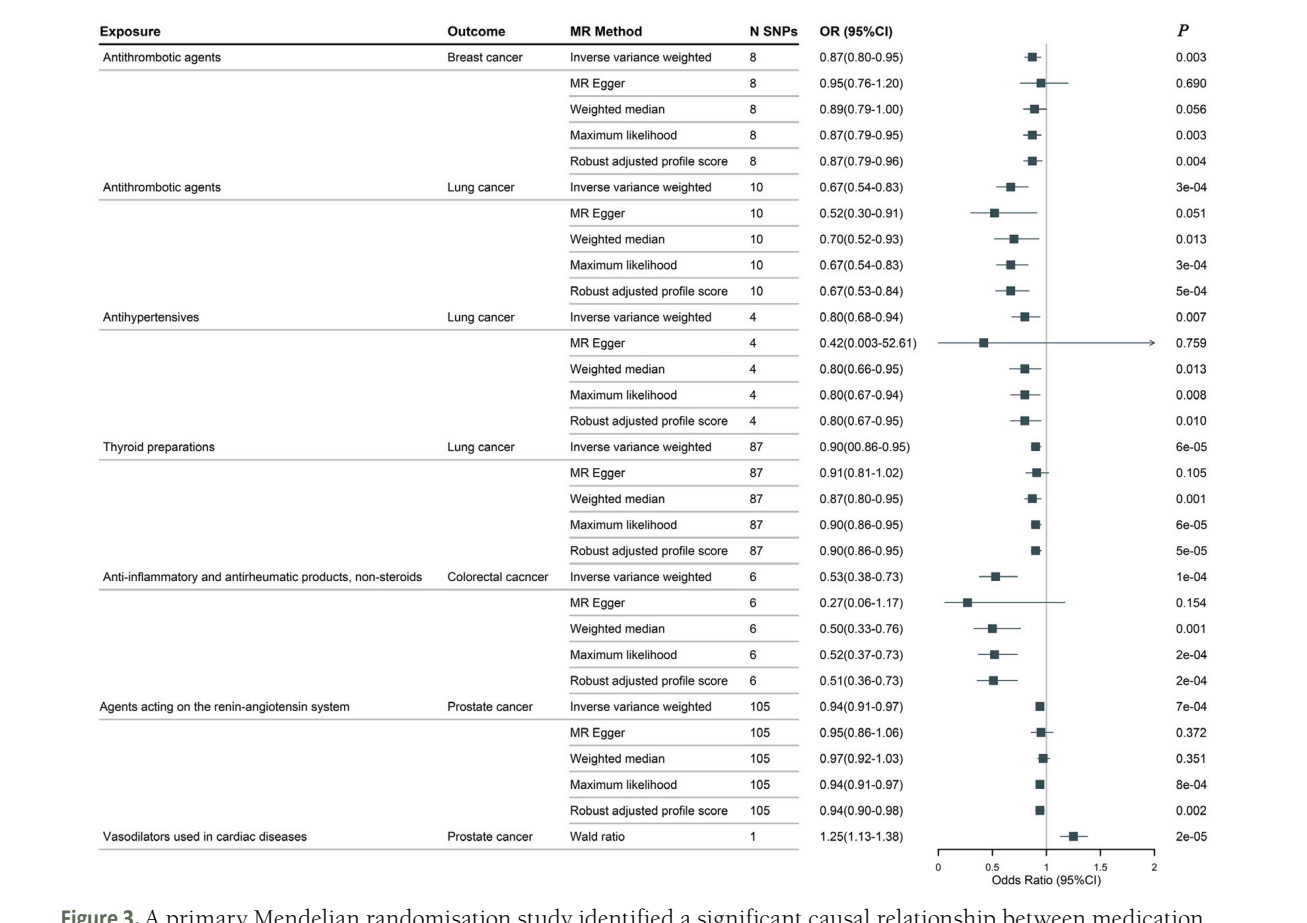
A primary Mendelian randomisation study identified a significant causal relationship between medication use and cancers. CI – confidence interval, MR method – Mendelian randomisation method, N SNPs – number of SNPs, OR – odds ratio

**Figure 4:**
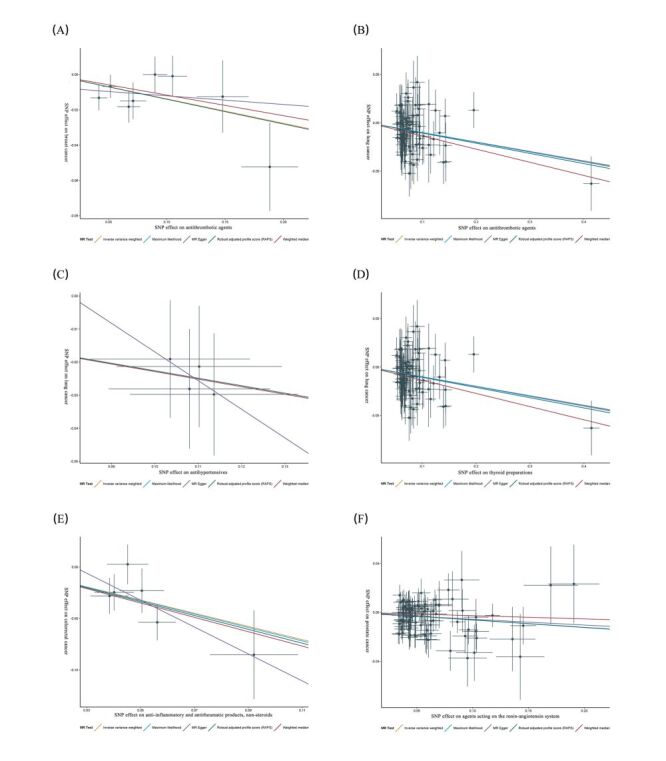
Scatterplot for the significant Mendelian randomisation association (*P* < 0.05) between medication use and cancers. **Panel A**. Antithrombotic agents and breast cancer. **Panel B**. Antithrombotic agents and lung cancer. **Panel C**. Antihypertensives and lung cancer. **Panel D**. Thyroid preparations and lung cancer. **Panel E**. Anti-inflammatory and antirheumatic products, non-steroids and colorectal cancer. **Panel F**. Agents acting on the renin-angiotensin system and prostate cancer. Horizontal and vertical lines represented standard errors for medication use and cancer, respectively. The slope of each line corresponds to the estimated MR effect from different methods. SNP – single nucleotide polymorphism

After rigorously screening IVs for using 19 medications, we performed two-sample MR on four site-specific cancers. For the 11 significant causal effects found, IVW radial MR was performed to identify and remove outliers prior to MR analysis (Table S13 in the **Online Supplementary Document**). At the same time, we performed phenotype scanning, discarding confounder-associated SNPs to avoid horizontal pleiotropy bias (Table S14 in the **Online Supplementary Document**). The summary data used for the final MR analysis are presented in Table S15 in the **Online Supplementary Document**. The *F*-statistics of all SNPs used for MR analysis were much greater than 10, indicating strong IVs. MR analysis revealed that genetic predisposition to the intake of antithrombotic agents reduced the odds of breast cancer (odds ratio (OR) = 0.87; 95% confidence interval (CI) = 0.80–0.95, *P* = 0.003), whereas anti-inflammatory and antirheumatic products (OR = 1.25; 95% CI = 1.00–1.55, *P* = 0.040) and antihistamines for systemic use (OR = 1.11; 95% CI = 1.04–1.18, *P* = 0.002) increased the odds of breast cancer. In terms of lung cancer, the intake of genetically predicted antithrombotic agents (OR = 0.67; 95% CI = 0.54–0.83, *P* = 0.0003), antihypertensives (OR = 0.80; 95% CI = 0.68–0.94, *P* = 0.007), salicylic acid and derivatives (OR = 0.75; 95% CI = 0.59–0.95, *P* = 0.018), HMG CoA reductase inhibitors (OR = 0.91; 95% CI = 0.84–0.99, *P* = 0.026) and thyroid preparations (OR = 0.90; 95% CI = 0.86–0.95, *P* = 0.00006) intake decreased the odds. For colorectal cancer, genetic predisposition to the use of anti-inflammatory and antirheumatic products showed a negative causal effect (OR = 0.53; 95% CI = 0.38–0.73, *P* = 0.0001). We also found a negative causal effect of genetic predisposition towards agents acting on the renin-angiotensin system (OR = 0.94; 95% CI = 0.91–0.97, *P* = 0.0007) in prostate cancer, whereas vasodilators used in cardiac diseases were detrimental (OR = 1.25; 95% CI = 1.13–1.38, *P* = 2.48E–05). The Cochran Q-test, MR-PRESSO, and MR-Egger intercept tests indicated no heterogeneity and pleiotropy in the MR estimates (Table S16 in the **Online Supplementary Document**). The proportion of variance explained for IVs and the statistical power of the MR analysis are shown in Table S17 in the **Online Supplementary Document**. However, the LOO analysis suggested that the causal effects between anti-inflammatory and antirheumatic products, nonsteroids, and antihistamines for systemic use and breast cancer, HMG CoA reductase inhibitors, salicylic acid and derivatives, and lung cancer are influenced by individual SNPs, and these MR estimates were not robust (Figure S5 in the **Online Supplementary Document**). Therefore, they were excluded in the subsequent analysis. Robust causality must satisfy the following criteria. First, IVW-derived *P* < 0.0026 (0.0026 < *P* < 0.05 is considered nominally significant). Second, the five MR estimates are directionally consistent. Third, sensitivity analyses suggested no heterogeneity or pleiotropy, and the LOO analysis was robust. Based on the above guidelines, we initially screened a convincing causality between medication use and cancer (**Figure 3**). The scatterplot showed the consistency of the estimated effects of the different MR models (**Figure 4**). MR estimates without heterogeneity and pleiotropy (Table S16 in the **Online Supplementary Document**). The LOO analysis results were robust, indicating that the MR estimates were not driven by a single SNP (Figure S6 in the **Online Supplementary Document**).

#### Replication and meta-analysis

**Figure 5:**
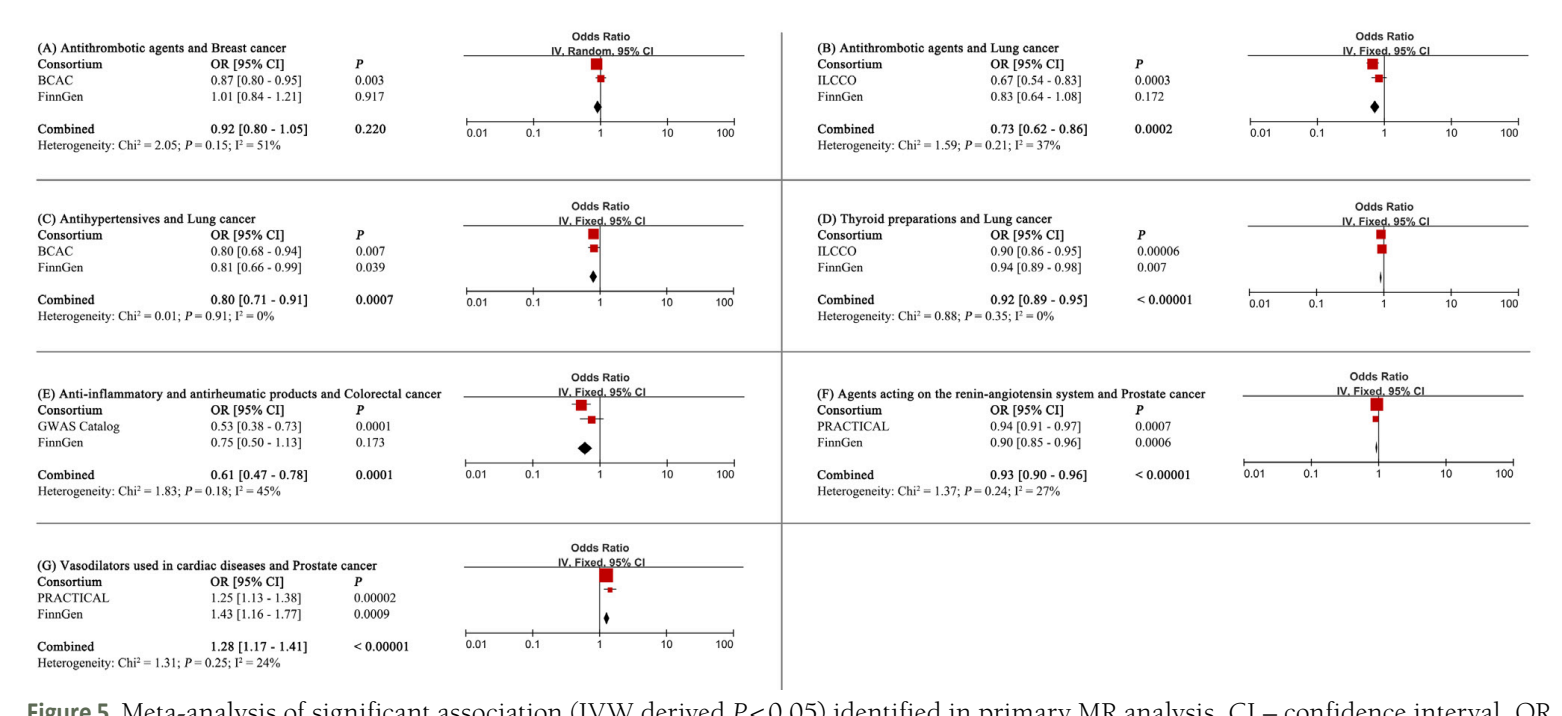
Meta-analysis of significant association (IVW derived *P* < 0.05) identified in primary MR analysis. CI – confidence interval, OR – odds ratio

To verify the causal effect identified, replication analysis and meta-analysis were performed in the FinnGen cohort, thus improving the sample size and statistical efficacy. The results of the meta-analysis (**Figure 5**) provided additional evidence supporting the use of genetically predisposed antithrombotic agents (OR = 0.73; 95% CI = 0.62–0.86, *P* = 0.0002), antihypertensives (OR = 0.80; 95% CI = 0.71–0.91, *P* = 0.0007), and thyroid preparations (OR = 0.92; 95% CI = 0.89–0.95, *P* < 0.00001) and a decreased odds of lung cancer. Genetic predisposition to anti-inflammatory and antirheumatic products uses reduced the odds of colorectal cancer. Regarding prostate cancer, we obtained consistent findings that taking genetically susceptible agents acting on the renin-angiotensin system (OR = 0.93; 95% CI = 0.90–0.96, *P* < 0.00001) reduced the odds of prostate cancer, while vasodilators used in cardiac diseases (OR = 1.28; 95% CI = 1.17–1.41, *P* < 0.00001) increased the odds of prostate cancer. In particular, the causality of antithrombotic agents (OR = 0.92; 95% CI = 0.80–1.05, *P* = 0.220) in breast cancer has not been confirmed.

#### Heritability, genetic correlation, and direction validation

For the identified associations, the genetic correlation (RG) and heritability between the medication-taking traits and cancer-related to them were calculated using the LDSC method (Table S18 **in the** **Online Supplementary Document**). We found little evidence of a genetic correlation between lung cancer and antihypertensive medications (RG = 0.217, SE (standard error) = 0.115, *P* = 0.059) and thyroid preparations (RG = –0.134, SE = 0.072, *P* = 0.063). Similarly, there were no shared genetic alterations between colorectal cancer and anti-inflammatory and antirheumatic products, nonsteroids (RG = 0.046, SE = 0.068, *P* = 0.498), or prostate cancer and agents acting on the renin-angiotensin system (RG = 0.018, SE = 0.031, *P* = 0.552) and vasodilators used in cardiac diseases (RG = –0.008, SE = 0.064, *P* = 0.899). However, the analysis of the genetic correlation between antithrombotic agents and lung cancer (RG = 0.267, SE = 0.075, *P* = 0.0003) suggests that a shared genetic component may confound the MR estimates for both. We further performed reverse MR and the Steiger test, and there was no evidence to support a reverse causality from medication-taking to cancer (Tables S19–20 in the **Online Supplementary Document**).

#### Comprehensive NHANES study and MR analysis

**Figure 6:**
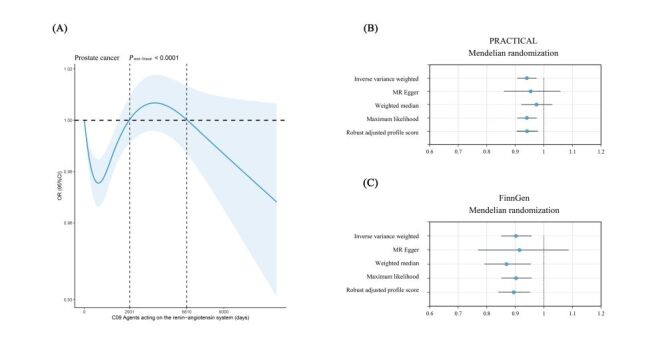
Causal evidence between agents acting on the renin-angiotensin system and prostate cancer was established by the cross-sectional study and MR analysis. **Panel A**. Analysis of the shape of the nonlinear association between agents acting on the renin-angiotensin system and prostate cancer using restricted cubic spline based on NHANES database. The solid blue line is the estimated odds ratio, and the shaded blue area is the 95% confidence interval. **Panels B** and (**C**). Two-sample Mendelian randomisation reveals causal evidence for agents acting on the renin-angiotensin system and prostate cancer. The forest plots illustrate the standardised odds ratio (OR) (95% confidence interval (CI)) for each two-sample MR method. PRACTICAL – Prostate Cancer Association Group to Investigate Cancer Associated Alterations in the Genome.

Combining the cross-sectional study results in NHANES and the MR analysis, we observed an association between agents acting on the renin-angiotensin system use and prostate cancer. Specifically, survey-weighted multivariate logistic regression showed that the use of agents that act on the renin-angiotensin system reduced the chances of prostate cancer (OR = 0.42; 95% CI = 0.27–0.63, *P* < 0.001). Further subgroup analysis found that users of ACEIs (OR = 0.51; 95% CI = 0.33–0.79, *P* = 0.003) and ARBs (OR = 0.38; 95% CI = 0.20–0.73, *P* = 0.004) showed low odds of subsequent prostate cancer. Survey-weighted RCS analysis revealed an s-shaped nonlinear relationship between the use of agents acting on the renin-angiotensin system and prostate cancer (**Figure 6**, panel A). The use of ACEIs and ARBs showed a similar nonlinear s-shaped correlation with prostate cancer (Figure S7 in the **Online Supplementary Document**). Similarly, MR analysis based on the Prostate Cancer Association Group to Investigate Cancer-Associated Alterations (**Figure 6**, panel B) and the FinnGen cohort (**Figure 6****,** panel C) provided genetic evidence that the use of agents acting on the renin-angiotensin system decreased the odds of prostate cancer.

## DISCUSSION

This study, which combined a large-scale cross-sectional study and MR analysis, provided evidence for the use of 19 medication types and the risk of four common cancers. The key findings of this work can be summarised as follows. There was a negative association between the use of agents acting on the renin-angiotensin system and prostate cancer risk. A nonlinear relationship of ‘decrease-to-increase-to-decrease’ was observed between the duration of use of agents acting on the renin-angiotensin system and prostate cancer risk. Comprehensive MR analysis provided evidence for a causal effect of agents acting on the renin-angiotensin system on a decreased risk of prostate cancer.

We provided sufficient evidence for the relationship between the use of 19 medications and four site-specific cancers. At first, we initially evaluated the linear and nonlinear association between medication use and cancer by multivariate logistic regression and RCS analysis in the context of weighted random sampling and PSM analysis based on data from the NHANES database. Further MR analysis investigated the causality between drug use and cancer. Multiple MR methods and sensitivity analyses showed that the MR findings were robust, and consistent MR estimates were obtained using two different data sets. Genetic variations in medication use possessed strong *F*-statistics, suggesting the absence of weak tools in our MR study. The weak and nonsignificant correlation between exposure and outcome determined by the LDSC method suggested that the IVs of interest for exposure did not directly influence outcome. The sufficiently large statistical power of MR analysis also ensured that true causal effects existed, if they existed at all. In general, the combined results of large cross-sectional studies and MR analysis identified robust evidence that using agents acting on the renin-angiotensin system can reduce the risk of prostate cancer.

The most recent and largest meta-analysis [4] involving 70 RCTs and 324 168 participants did not show a significant association between ACEIs or ARBs use and cancer. However, most RCTs had a follow-up of one to five years. Considering that events are commonly evenly distributed in trials, an average time of less than three years of drug treatment before a cancer diagnosis is considered too short, making it difficult to reach a convincing conclusion [44,45]. Furthermore, high blood pressure is in itself a risk factor for cancer at certain sites. In contrast, the results of another retrospective cohort study with up to 16 years of follow-up were more convincing. Lever et al. [46] followed hypertensive patients who began ACEIs therapy after 1 January 1980, at the Glasgow Blood Pressure Clinic to the date of death or 31 December 1995. Compared with the incidence of cancer in the general population of 2.7 million people in the west of Scotland, the relative risks of new and fatal cancers in the 1559 patients who received ACEIs were 0.72 (95% CI = 0.55–0.92), and 0.65 (95% CI = 0.44–0.93), respectively. Another meta-analysis [5] including 12 cohort and 16 case-control studies found no evidence that ACEIs or ARBs were associated with cancer risk. However, reduced cancer risk was found in the meta-analysis of only 11 studies with up to five years of follow-up. In particular, subgroup meta-analyses of five cohort studies and nested case-control studies provided evidence that the use of ACEIs or ARBs can reduce the risk of prostate cancer. This difference was consistent with the results of our RCS analysis that the duration of ACEIs or ARBs administration showed a ‘decrease-to-increase-to-decrease’ s-shaped nonlinear relationship with prostate cancer risk. Furthermore, our findings are also supported by several fundamental studies. It is known that the renin-angiotensin system is expressed on the walls of the kidneys, heart, and blood vessels and plays an important role in regulating blood pressure, body fluids, and electrolytes through the release of enzymes and peptides, among other substances. Similar to the cardiovascular system, the primary components of the renin-angiotensin system, such as angiotensin receptors and renin, have been recognised locally in the prostate [47]. The anticancer effects of ACEIs and ARBs have been shown to depend on the down-regulation of the renin-angiotensin system of angiotensin II levels and the promotion of bradykinin [48]. Specifically, as a key active peptide of the renin-angiotensin system, binding of angiotensin II to the angiotensin II type one (AT1) receptor promotes cell proliferation, angiogenesis, and inflammation, increasing the risk of cancer [49]. In particular, significantly high expression of the AT1 receptor in prostate cancer tissues was confirmed by Uemura et al. using RT-PCR analysis [12]. Research-based on mRNA levels similarly found that the AT1 receptor was expressed in normal human prostate epithelial cells, PrSC, a prostate stromal cell line, human prostate cancer tissue, and human cancer cell lines [12,50]. In hormone-refractory prostate cancer cells, angiotensin II up-regulation of the transcription factors hypoxia-inducible factor-1α (HIF-1α) and E26 transformation-specific-1 (Ets-1) promotion of VEGF production can be blocked by ARBs [51]. Therefore, it is reasonable to believe that ACEIs decreased angiotensin II levels by inhibiting angiotensin-converting enzyme activity, while ARBs inhibited angiogenesis and tumour cell proliferation by suppressing angiotensin II binding to the AT1 receptor to produce antiprostate cancer effects. In addition, other experimental studies have also provided evidence supporting the role of angiotensin II in cancer initiation, progression, invasion, and metastasis [52, 53]. However, we should note that patients receiving ARBs had higher levels of angiotensin II compared to those who took ACEIs [54], which increased cancer risk to some extent. Blocking the AT1 receptor by ARBs increases AT2 receptor expression, which can stimulate tumour angiogenesis [55]. Although elevated levels of angiotensin II and the stimulation of the AT2 receptor appear to explain the different results from some clinical studies, these are not the main effects of ARBs. In addition, sulfhydryl groups in ACEIs can inhibit angiogenesis to prevent tumour invasion by generating angiostatins, removing free radicals and reducing reactive oxygen species to inhibit the activation of matrix metalloproteinase and VEGF [56]. Along with the mechanisms of action mentioned above, several indirect effects may explain the preventive effects of ACEIs and ARBs on prostate cancer. For example, the use of ACEIs and ARBs can alleviate insulin resistance [57], which is considered a risk factor for the prostate, through the renin-angiotensin system [58]. Another study found that individuals with the ACE genotype tend to smoke [59] and that smoking increases renin activity, which promotes angiotensin II production, increasing the risk of prostate cancer [60]. Additionally, when individuals have been diagnosed with cardiovascular disease, they deliberately modify their unhealthy lifestyle habits or undergo regular check-ups to prevent cancer.

Some of the significant causal associations eventually identified by MR analysis, such as the causal effects of antithrombotic agents, antihypertensives, and thyroid preparations on lung cancer, and anti-inflammatory and antirheumatic products, nonsteroids on colorectal cancer, were not confirmed in the cross-sectional study. In particular, it is not a coincidence that the MR analysis and the cross-sectional study showed consistent directionality (negative), although the cross-sectional findings were non-significant. Chronic inflammation is a definite risk factor for gastrointestinal cancer, and non-steroidal anti-inflammatory drugs can reduce the risk of gastrointestinal cancer by inhibiting the cyclooxygenase-2-prostaglandin E2 pathway [61]. Rothwell et al. [62] found that aspirin significantly reduced the 20-year risk of colon cancer after 20 years of follow-up of five RCTs involving 14 033 patients. Pawitan et al. also showed that anti-inflammatory drug use significantly inhibited the growth of colorectal cancer, while antithrombotic drug use reduced the risk of metastasis in both colorectal and lung cancers [63]. However, previous findings on the relationship between the use of thyroid preparations and lung cancer contradict our findings. A retrospective cohort study [64] of 85 733 313 Swedes found that the use of levothyroxine increased the overall risk of cancer in men and women, although no significant association with lung cancer was found in men and women. LT4 has also been reported to be one of several endogenous factors that favour lung cancer cell proliferation [65]. Thus, the finding that thyroid preparations use can reduce lung cancer risk may need to be interpreted with caution. Future experimental studies and larger RCTs with longer follow-ups are necessary. For vasodilators used in cardiac diseases and prostate cancer, the MR study and cross-sectional study obtained opposite results. In the MR analysis, since the IVs had only one SNP rs55730499, other MR models and sensitivity analyses were not performed, making the MR results insufficiently evaluable. MR estimates obtained for a single SNP may be biased due to the weak statistical efficacy of IVs, the inability to probe with multiple MR models, and the inability to implement sensitivity analyses. Overall, further rigorous large RCTs and basic experiments are necessary to clarify the causality between thyroid preparations and lung cancer, vasodilators used in cardiac diseases, and prostate cancer.

Some limitations should be considered when interpreting the findings of this study. First, the NHANES database is derived from participant self-reports, and there may be recall bias in the timing of medication use and cancer diagnosis. Second, this study classified drugs into 19 categories, and it is necessary to refine the causal relationship between specific drugs, the timing and dosage of medications, and cancer in the future. Third, although the MR study was able to avoid confounders, genetic variation has limited explanations for the propensity to medication use in humans. In particular, when people have been treated with medications for a prolonged time, they can deliberately correct bad habits and have regular medical check-ups to the extent that it reduces the risk of cancer, making MR estimates weakly biased from real-world findings. Thus, further in-depth mechanistic studies and large RCTs with sufficiently long follow-up assessments are necessary. Fourth, another limitation of the MR study is the unavoidable presence of pleiotropy, although we implemented sensitivity analyses and phenotypic scans to discard exposure-related SNPs to diminish the occurrence of horizontal pleiotropy. However, IVs strongly associated with exposure may also influence outcomes through other unmeasured factors, and this weak horizontal pleiotropy is challenging to quantify and address. Fifth, despite efforts to address the issue of weak genetic variation through various statistical tests, it is necessary to recognise that the issue may still exist to some extent. Sixth, the population in this study was of European ancestry, and caution should be taken when generalising the findings to other populations. Therefore, the interpretation of our findings should be cautious and more investigation is needed to confirm our findings and solve potential sources of bias.

However, this study also showed outstanding strengths. First, this study systematically evaluated the causal relationship between treatment with 19 drug types and cancer by combining a large-scale cross-sectional study and MR analysis. Second, the cross-sectional study applied weighted random sampling to make the sample sufficiently representative, and multiple confounder corrections after PSM made the findings more convincing. Third, linear and nonlinear analyses were performed to more fully reveal the associations between the use of 19 medications and cancer, whereas we clarified the timing of drug use and cancer diagnosis and combined rigorous MR analysis to identify causal effects. Fourth, MR analysis effectively avoided confounding and consisted of five MR models, multiple sensitivity analysis methods, and mutual validation in two different databases, which improved the persuasiveness of the findings.

## CONCLUSIONS

Our study provides robust evidence that the use of drugs acting on the renin-angiotensin system can reduce the risk of prostate cancer. Given the high prevalence of prostate cancer, this finding has important implications for drug selection in patients with cardiovascular disease and drug development and prevention of prostate cancer.

## Additional material


Online Supplementary Document

## References

[1] Sung H Ferlay J Siegel RL Laversanne M Soerjomataram I Jemal A Global Cancer Statistics 2020: GLOBOCAN Estimates of Incidence and Mortality Worldwide for 36 Cancers in 185 Countries. CA Cancer J Clin. 2021;71:209-49. 10.3322/caac.21660 33538338

[2] Bloch MJ Worldwide prevalence of hypertension exceeds 1.3 billion. J Am Soc Hypertens. 2016;10:753-4. 10.1016/j.jash.2016.08.006 27660007

[3] Sipahi I Debanne SM Rowland DY Simon DI Fang JC Angiotensin-receptor blockade and risk of cancer: meta-analysis of randomised controlled trials. Lancet Oncol. 2010;11:627-36. 10.1016/S1470-2045(10)70106-6 20542468 PMC4070221

[4] Bangalore S Kumar S Kjeldsen SE Makani H Grossman E Wetterslev J Antihypertensive drugs and risk of cancer: network meta-analyses and trial sequential analyses of 324,168 participants from randomised trials. Lancet Oncol. 2011;12:65-82. 10.1016/S1470-2045(10)70260-6 21123111

[5] Yoon C Yang HS Jeon I Chang Y Park SM Use of angiotensin-converting-enzyme inhibitors or angiotensin-receptor blockers and cancer risk: a meta-analysis of observational studies. CMAJ. 2011;183:E1073-84. 10.1503/cmaj.101497 21876027 PMC3185099

[6] Yang X Zhu MJ Sreejayan N Ren J Du M Angiotensin II promotes smooth muscle cell proliferation and migration through release of heparin-binding epidermal growth factor and activation of EGF-receptor pathway. Mol Cells. 2005;20:263-70. 10.1016/S1016-8478(23)13226-2 16267402

[7] Chua CC Hamdy RC Chua BH Upregulation of vascular endothelial growth factor by angiotensin II in rat heart endothelial cells. Biochim Biophys Acta. 1998;1401:187-94. 10.1016/S0167-4889(97)00129-8 9531974

[8] Deshayes F Nahmias C Angiotensin receptors: a new role in cancer? Trends Endocrinol Metab. 2005;16:293-9. 10.1016/j.tem.2005.07.009 16061390

[9] Daemen MJ Lombardi DM Bosman FT Schwartz SM Angiotensin II induces smooth muscle cell proliferation in the normal and injured rat arterial wall. Circ Res. 1991;68:450-6. 10.1161/01.RES.68.2.450 1991349

[10] Buharalioglu CK Song CY Yaghini FA Ghafoor HU Motiwala M Adris T Angiotensin II-induced process of angiogenesis is mediated by spleen tyrosine kinase via VEGF receptor-1 phosphorylation. Am J Physiol Heart Circ Physiol. 2011;301:H1043-55. 10.1152/ajpheart.01018.2010 21642504 PMC3191073

[11] Li SH Lu HI Chang AY Huang WT Lin WC Lee CC Angiotensin II type I receptor (AT1R) is an independent prognosticator of esophageal squamous cell carcinoma and promotes cells proliferation via mTOR activation. Oncotarget. 2016;7:67150-65. 10.18632/oncotarget.11567 27564102 PMC5341864

[12] Uemura H Ishiguro H Nakaigawa N Nagashima Y Miyoshi Y Fujinami K Angiotensin II receptor blocker shows antiproliferative activity in prostate cancer cells: a possibility of tyrosine kinase inhibitor of growth factor. Mol Cancer Ther. 2003;2:1139-47. 14617787

[13] Boursi B Lurie I Mamtani R Haynes K Yang YX Anti-depressant therapy and cancer risk: a nested case-control study. Eur Neuropsychopharmacol. 2015;25:1147-57. 10.1016/j.euroneuro.2015.04.010 25934397

[14] Tamim HM Mahmud S Hanley JA Boivin JF Stang MR Collet JP Antidepressants and risk of prostate cancer: a nested case-control study. Prostate Cancer Prostatic Dis. 2008;11:53-60. 10.1038/sj.pcan.4501003 17684479

[15] Lin WY Chen VC Chiu WC Yim SJ Ho PT McIntyre RS Prostate cancer and antidepressants: A nationwide population-based nested case-control study. J Affect Disord. 2018;227:834-9. 10.1016/j.jad.2017.11.039 29689697

[16] Ahluwalia N Dwyer J Terry A Moshfegh A Johnson C Update on NHANES Dietary Data: Focus on Collection, Release, Analytical Considerations, and Uses to Inform Public Policy. Adv Nutr. 2016;7:121-34. 10.3945/an.115.009258 26773020 PMC4717880

[17] Smith GD Ebrahim S ‘Mendelian randomization’: can genetic epidemiology contribute to understanding environmental determinants of disease? Int J Epidemiol. 2003;32:1-22. 10.1093/ije/dyg070 12689998

[18] Burgess S Butterworth A Thompson SG Mendelian randomization analysis with multiple genetic variants using summarized data. Genet Epidemiol. 2013;37:658-65. 10.1002/gepi.21758 24114802 PMC4377079

[19] Wu Y Byrne EM Zheng Z Kemper KE Yengo L Mallett AJ Genome-wide association study of medication-use and associated disease in the UK Biobank. Nat Commun. 2019;10:1891. 10.1038/s41467-019-09572-5 31015401 PMC6478889

[20] Smith L Parris C Veronese N Shang C Lopez-Sanchez GF Jacob L Cross-sectional associations between angiotensin-converting enzyme inhibitor use and cancer diagnosis in US adults. Clin Exp Med. 2020;20:409-16. 10.1007/s10238-020-00622-7 32219665

[21] Bao W Liu B Simonsen DW Lehmler HJ Association Between Exposure to Pyrethroid Insecticides and Risk of All-Cause and Cause-Specific Mortality in the General US Adult Population. JAMA Intern Med. 2020;180:367-74. 10.1001/jamainternmed.2019.6019 31886824 PMC6990752

[22] Boef AG Dekkers OM le Cessie S Mendelian randomization studies: a review of the approaches used and the quality of reporting. Int J Epidemiol. 2015;44:496-511. 10.1093/ije/dyv071 25953784

[23] Skrivankova VW Richmond RC Woolf BAR Yarmolinsky J Davies NM Swanson SA Strengthening the Reporting of Observational Studies in Epidemiology Using Mendelian Randomization: The STROBE-MR Statement. JAMA. 2021;326:1614-21. 10.1001/jama.2021.18236 34698778

[24] Nevado-Holgado AJ Kim CH Winchester L Gallacher J Lovestone S Commonly prescribed drugs associate with cognitive function: a cross-sectional study in UK Biobank. BMJ Open. 2016;6:e012177. 10.1136/bmjopen-2016-012177 27903560 PMC5168501

[25] Santos R Ursu O Gaulton A Bento AP Donadi RS Bologa CG A comprehensive map of molecular drug targets. Nat Rev Drug Discov. 2017;16:19-34. 10.1038/nrd.2016.230 27910877 PMC6314433

[26] Michailidou K Lindstrom S Dennis J Beesley J Hui S Kar S Association analysis identifies 65 new breast cancer risk loci. Nature. 2017;551:92-4. 10.1038/nature24284 29059683 PMC5798588

[27] Wang Y McKay JD Rafnar T Wang Z Timofeeva MN Broderick P Rare variants of large effect in BRCA2 and CHEK2 affect risk of lung cancer. Nat Genet. 2014;46:736-41. 10.1038/ng.3002 24880342 PMC4074058

[28] Huyghe JR Bien SA Harrison TA Kang HM Chen S Schmit SL Discovery of common and rare genetic risk variants for colorectal cancer. Nat Genet. 2019;51:76-87. 10.1038/s41588-018-0286-6 30510241 PMC6358437

[29] Schumacher FR Al Olama AA Berndt SI Benlloch S Ahmed M Saunders EJ Association analyses of more than 140,000 men identify 63 new prostate cancer susceptibility loci. Nat Genet. 2018;50:928-36. 10.1038/s41588-018-0142-8 29892016 PMC6568012

[30] Clarke L Zheng-Bradley X Smith R Kulesha E Xiao C Toneva I The 1000 Genomes Project: data management and community access. Nat Methods. 2012;9:459-62. 10.1038/nmeth.1974 22543379 PMC3340611

[31] Yun Z Guo Z Li X Shen Y Nan M Dong Q Genetically predicted 486 blood metabolites in relation to risk of colorectal cancer: A Mendelian randomization study. Cancer Med. 2023;12:13784-99. 10.1002/cam4.6022 37132247 PMC10315807

[32] Gill D Efstathiadou A Cawood K Tzoulaki I Dehghan A Education protects against coronary heart disease and stroke independently of cognitive function: evidence from Mendelian randomization. Int J Epidemiol. 2019;48:1468-77. 10.1093/ije/dyz200 31562522 PMC6857750

[33] Bowden J Spiller W Del Greco MF Sheehan N Thompson J Minelli C Improving the visualization, interpretation and analysis of two-sample summary data Mendelian randomization via the Radial plot and Radial regression. Int J Epidemiol. 2018;47:2100. 10.1093/ije/dyy265 30423109 PMC6280936

[34] Pierce BL Burgess S Efficient design for Mendelian randomization studies: subsample and 2-sample instrumental variable estimators. Am J Epidemiol. 2013;178:1177-84. 10.1093/aje/kwt084 23863760 PMC3783091

[35] Burgess S Thompson SG Interpreting findings from Mendelian randomization using the MR-Egger method. Eur J Epidemiol. 2017;32:377-89. 10.1007/s10654-017-0255-x 28527048 PMC5506233

[36] Milligan BG Maximum-likelihood estimation of relatedness. Genetics. 2003;163:1153-67. 10.1093/genetics/163.3.1153 12663552 PMC1462494

[37] Patel A Ye T Xue H Lin Z Xu S Woolf B MendelianRandomization v0.9.0: updates to an R package for performing Mendelian randomization analyses using summarized data. Wellcome Open Res. 2023;8:449. 10.12688/wellcomeopenres.19995.2 37915953 PMC10616660

[38] Zhao QY Wang JS Hemani G Bowden J Small DS Statistical Inference in Two-Sample Summary-Data Mendelian Randomization Using Robust Adjusted Profile Score. Ann Stat. 2020;48:1742-69. 10.1214/19-AOS1866

[39] Cohen JF Chalumeau M Cohen R Korevaar DA Khoshnood B Bossuyt PM Cochran’s Q test was useful to assess heterogeneity in likelihood ratios in studies of diagnostic accuracy. J Clin Epidemiol. 2015;68:299-306. 10.1016/j.jclinepi.2014.09.005 25441698

[40] Bowden J Del Greco MF Minelli C Zhao Q Lawlor DA Sheehan NA Improving the accuracy of two-sample summary-data Mendelian randomization: moving beyond the NOME assumption. Int J Epidemiol. 2019;48:728-42. 10.1093/ije/dyy258 30561657 PMC6659376

[41] Kurki MI Karjalainen J Palta P Sipila TP Kristiansson K Donner KM FinnGen provides genetic insights from a well-phenotyped isolated population. Nature. 2023;613:508-18. 10.1038/s41586-022-05473-8 36653562 PMC9849126

[42] O’Connor LJ Price AL Distinguishing genetic correlation from causation across 52 diseases and complex traits. Nat Genet. 2018;50:1728-34. 10.1038/s41588-018-0255-0 30374074 PMC6684375

[43] Bulik-Sullivan BK Loh PR Finucane HK Ripke S Yang J Schizophrenia Working Group of the Psychiatric Genomics C LD Score regression distinguishes confounding from polygenicity in genome-wide association studies. Nat Genet. 2015;47:291-5. 10.1038/ng.3211 25642630 PMC4495769

[44] Lindholm LH Carlberg B Blood-pressure drugs and cancer: much ado about nothing? Lancet Oncol. 2011;12:6-8. 10.1016/S1470-2045(10)70271-0 21123112

[45] Julius S Kjeldsen SE Weber MA Angiotensin-receptor blockade, cancer, and concerns. Lancet Oncol. 2010;11:820-1. 10.1016/S1470-2045(10)70159-5 20816379

[46] Lever AF Hole DJ Gillis CR McCallum IR McInnes GT MacKinnon PL Do inhibitors of angiotensin-I-converting enzyme protect against risk of cancer? Lancet. 1998;352:179-84. 10.1016/S0140-6736(98)03228-0 9683206

[47] Uemura H Hasumi H Ishiguro H Teranishi J Miyoshi Y Kubota Y Renin-angiotensin system is an important factor in hormone refractory prostate cancer. Prostate. 2006;66:822-30. 10.1002/pros.20407 16482568

[48] Lindberg H Nielsen D Jensen BV Eriksen J Skovsgaard T Angiotensin converting enzyme inhibitors for cancer treatment? Acta Oncol. 2004;43:142-52. 10.1080/02841860310022346 15163162

[49] Shen J Huang YM Wang M Hong XZ Song XN Zou X Renin-angiotensin system blockade for the risk of cancer and death. J Renin Angiotensin Aldosterone Syst. 2016;17:1470320316656679. 10.1177/1470320316656679 27402638 PMC5843874

[50] Lin J Freeman MR Transactivation of ErbB1 and ErbB2 receptors by angiotensin II in normal human prostate stromal cells. Prostate. 2003;54:1-7. 10.1002/pros.10160 12481249

[51] Kosaka T Miyajima A Shirotake S Kikuchi E Hasegawa M Mikami S Ets-1 and hypoxia inducible factor-1alpha inhibition by angiotensin II type-1 receptor blockade in hormone-refractory prostate cancer. Prostate. 2010;70:162-9. 10.1002/pros.21049 19760626

[52] Nogueira EF Vargas CA Otis M Gallo-Payet N Bollag WB Rainey WE Angiotensin-II acute regulation of rapid response genes in human, bovine, and rat adrenocortical cells. J Mol Endocrinol. 2007;39:365-74. 10.1677/JME-07-0094 18055484

[53] Ribatti D Conconi MT Nussdorfer GG Nonclassic endogenous novel [corrected] regulators of angiogenesis. Pharmacol Rev. 2007;59:185-205. 10.1124/pr.59.2.3 17540906

[54] Levy BI How to explain the differences between renin angiotensin system modulators. Am J Hypertens. 2005;18:134S-41S. 10.1016/j.amjhyper.2005.05.005 16125050

[55] Walther T Menrad A Orzechowski HD Siemeister G Paul M Schirner M Differential regulation of in vivo angiogenesis by angiotensin II receptors. FASEB J. 2003;17:2061-7. 10.1096/fj.03-0129com 14597675

[56] Hanif K Bid HK Konwar R Reinventing the ACE inhibitors: some old and new implications of ACE inhibition. Hypertens Res. 2010;33:11-21. 10.1038/hr.2009.184 19911001

[57] Perkins JM Davis SN The renin-angiotensin-aldosterone system: a pivotal role in insulin sensitivity and glycemic control. Curr Opin Endocrinol Diabetes Obes. 2008;15:147-52. 10.1097/MED.0b013e3282f7026f 18316950

[58] Kaiser A Haskins C Siddiqui MM Hussain A D’Adamo C The evolving role of diet in prostate cancer risk and progression. Curr Opin Oncol. 2019;31:222-9. 10.1097/CCO.0000000000000519 30893147 PMC7379157

[59] Arima H Kiyohara Y Tanizaki Y Nakabeppu Y Kubo M Kato I Angiotensin I-converting enzyme gene polymorphism modifies the smoking-cancer association: the Hisayama Study. Eur J Cancer Prev. 2006;15:196-201. 10.1097/01.cej.0000199506.15571.37 16679861

[60] Laustiola KE Lassila R Nurmi AK Enhanced activation of the renin-angiotensin-aldosterone system in chronic cigarette smokers: a study of monozygotic twin pairs discordant for smoking. Clin Pharmacol Ther. 1988;44:426-30. 10.1038/clpt.1988.175 3048842

[61] Wang D Cabalag CS Clemons NJ DuBois RN Cyclooxygenases and Prostaglandins in Tumor Immunology and Microenvironment of Gastrointestinal Cancer. Gastroenterology. 2021;161:1813-29. 10.1053/j.gastro.2021.09.059 34606846

[62] Rothwell PM Wilson M Elwin CE Norrving B Algra A Warlow CP Long-term effect of aspirin on colorectal cancer incidence and mortality: 20-year follow-up of five randomised trials. Lancet. 2010;376:1741-50. 10.1016/S0140-6736(10)61543-7 20970847

[63] Pawitan Y Yin L Setiawan A Auer G Smedby KE Czene K Distinct effects of anti-inflammatory and anti-thrombotic drugs on cancer characteristics at diagnosis. Eur J Cancer. 2015;51:751-7. 10.1016/j.ejca.2015.02.004 25726091

[64] Wändell P Carlsson AC Li X Sundquist J Sundquist K Levothyroxine treatment is associated with an increased relative risk of overall and organ specific incident cancers - a cohort study of the Swedish population. Cancer Epidemiol. 2020;66:101707. 10.1016/j.canep.2020.101707 32222650

[65] Meng R Tang HY Westfall J London D Cao JH Mousa SA Crosstalk between integrin alphavbeta3 and estrogen receptor-alpha is involved in thyroid hormone-induced proliferation in human lung carcinoma cells. PLoS One. 2011;6:e27547. 10.1371/journal.pone.0027547 22132110 PMC3222665

