# Supplementary material for: Association between 19 medication use and risk of common cancers: A cross-sectional and Mendelian randomisation study
Source: J Glob Health. 2024 Mar 15;14:04057. doi: 10.7189/jogh.14.04057 (PMC10940964; doi:10.7189/jogh.14.04057)
Supplement: Online Supplementary Document [file jogh-14-04057-s001.pdf]

Association between 19 medication use and risk of 4 site-specific cancers: evidence from a cross-sectional and Mendelian randomization study

Table S1. Classification of 19 medications
Table S2. Information regarding 19 drugs in the NHANES database
Table S3. STROBE-MR checklist of recommended items to address in reports of Mendelian randomization study
Table S4. Active ingredients and ATC code of medications in the UK Biobank
Table S5. Detailed information on summary data for 4 site-specific cancers in primary MR analysis
Table S6. Instrumental variables were used to perform MR analysis instead of 19 medication use
Table S7. Detailed information on summary data for 4 site-specific cancers in replication MR analysis
Table S8. Characteristics of the 19 medications used in the NHANES study
Table S9. The results of survey-weighted multivariate logistic regression analysis for medication use and breast cancer in the NHANES study
Table S10. The results of survey-weighted multivariate logistic regression analysis for medication use and lung cancer in the NHANES study
Table S11. The results of survey-weighted multivariate logistic regression analysis for medication use and colorectal cancer in the NHANES study
Table S12. The results of survey-weighted multivariate logistic regression analysis for medication use and prostate cancer in the NHANES study
Table S13. Outliers identified by IVW Radial MR in primary MR analysis
Table S14. SNPs associated with confounders identified by phenotype scanning in primary MR analysis
Table S15. Summary data on eleven significant causal associations between medication use and cancer ultimately used in primary MR analysis
Table S16. Results of MR estimates and sensitivity analyses of eleven significant causal effects of medication use and cancer
Table S17. The proportion of variance explained for the instrumental variables and the statistical power of the primary MR analysis

Table S18. Heritability and genetic correlation between medication use and cancer
Table S19. Reverse MR analysis from cancer on medication use
Table S20. Steiger direction test from medication use to cancer
Figure S1. Analysis of the shape of the non-linear association between the duration of drug use and breast cancer using restricted cubic spline based on the NHANES database.
Figure S2. Analysis of the shape of the non-linear association between the duration of drug use and lung cancer using restricted cubic spline based on the NHANES database.
Figure S3. Analysis of the shape of the non-linear association between duration of drug use and colorectal cancer using restricted cubic spline based on the NHANES database.
Figure S4. Analysis of the shape of the non-linear association between duration of drug use and prostate cancer using restricted cubic spline based on the NHANES database.
Figure S5. Unrobust leave one out analysis in significant associations between genetically determined medication use and cancer.
Figure S6. Robust leave-one-out sensitivity analyses in significant associations between genetically determined medication use and cancer.
Figure S7. Analysis of the shape of the non-linear association between duration of angiotensin-converting enzyme inhibitor and angiotensin receptor blocker and prostate cancer using the restricted cubic spline model based on the NHANES database.

Table S1. Classification of 19 medications

#	Medication category	Medications
1	A02B	Drugs for peptic ulcer and GORD
2	A10	Drugs used in diabetes
3	B01A	Antithrombotic agents
4	C01D	Vasodilators used in cardiac diseases
5	C02	Antihypertensives
6	C03	Diuretics
7	C07	Beta blocking agents
8	C08	Calcium channel blockers
9	C09	Agents acting on the renin-angiotensin system
10	C10AA	HMG CoA reductase inhibitors
11	H03A	Thyroid preparations
12	L04	Immunosuppressants
13	M01A	Antiinflammatory and antirheumatic products, non-steroids
14	M05B	Drugs affecting bone structure and mineralization
15	N02A	Opioids
16	N02C	Antimigraine preparations
17	N06A	Antidepressants
18	R03BA	Glucocorticoids
19	R06A	Antihistamines for systemic use

Table S2. Information regarding 19 drugs in the NHANES database

Medication category	Medications	Drug name in NHANES
A02B	Drugs for peptic ulcer and GORD	CIMETIDINE
A02B	Drugs for peptic ulcer and GORD	DEXLANSOPRAZOLE
A02B	Drugs for peptic ulcer and GORD	ESOMEPRAZOLE
A02B	Drugs for peptic ulcer and GORD	FAMOTIDINE
A02B	Drugs for peptic ulcer and GORD	LANSOPRAZOLE
A02B	Drugs for peptic ulcer and GORD	MISOPROSTOL
A02B	Drugs for peptic ulcer and GORD	NIZATIDINE
A02B	Drugs for peptic ulcer and GORD	OMEPRAZOLE
A02B	Drugs for peptic ulcer and GORD	PANTOPRAZOLE
A02B	Drugs for peptic ulcer and GORD	RABEPRAZOLE
A02B	Drugs for peptic ulcer and GORD	RANITIDINE
A02B	Drugs for peptic ulcer and GORD	SODIUM BICARBONATE
A02B	Drugs for peptic ulcer and GORD	SUCRALFATE
A10	Drugs used in diabetes	ACARBOSE
A10	Drugs used in diabetes	ALBIGLUTIDE
A10	Drugs used in diabetes	ALOGLIPTIN
A10	Drugs used in diabetes	ANTIDIABETIC AGENTS - UNSPECIFIED
A10	Drugs used in diabetes	CANAGLIFLOZIN
A10	Drugs used in diabetes	CHLORPROPAMIDE
A10	Drugs used in diabetes	DAPAGLIFLOZIN
A10	Drugs used in diabetes	DULAGLUTIDE
A10	Drugs used in diabetes	EMPAGLIFLOZIN
A10	Drugs used in diabetes	ERTUGLIFLOZIN
A10	Drugs used in diabetes	EXENATIDE
A10	Drugs used in diabetes	GLICLAZIDE
A10	Drugs used in diabetes	GLIMEPIRIDE
A10	Drugs used in diabetes	GLIPIZIDE
A10	Drugs used in diabetes	GLYBURIDE
A10	Drugs used in diabetes	INSULIN
A10	Drugs used in diabetes	INSULIN ASPART
A10	Drugs used in diabetes	INSULIN ASPART; INSULIN ASPART PROTAMINE
A10	Drugs used in diabetes	INSULIN DEGLUDEC
A10	Drugs used in diabetes	INSULIN DEGLUDEC; LIRAGLUTIDE
A10	Drugs used in diabetes	INSULIN DETEMIR
A10	Drugs used in diabetes	INSULIN GLARGINE
A10	Drugs used in diabetes	INSULIN GLARGINE; LIXISENATIDE
A10	Drugs used in diabetes	INSULIN GLULISINE
A10	Drugs used in diabetes	INSULIN ISOPHANE (NPH)
A10	Drugs used in diabetes	INSULIN ISOPHANE; INSULIN

		REGULAR
A10	Drugs used in diabetes	INSULIN LISPRO
A10	Drugs used in diabetes	INSULIN LISPRO; INSULIN LISPRO PROTAMINE
A10	Drugs used in diabetes	INSULIN REGULAR
A10	Drugs used in diabetes	INSULIN ZINC
A10	Drugs used in diabetes	LINAGLIPTIN
A10	Drugs used in diabetes	LIRAGLUTIDE
A10	Drugs used in diabetes	METFORMIN
A10	Drugs used in diabetes	MIGLITOL
A10	Drugs used in diabetes	NATEGLINIDE
A10	Drugs used in diabetes	PIOGLITAZONE
A10	Drugs used in diabetes	REPAGLINIDE
A10	Drugs used in diabetes	ROSIGLITAZONE
A10	Drugs used in diabetes	SAXAGLIPTIN
A10	Drugs used in diabetes	SEMAGLUTIDE
A10	Drugs used in diabetes	SITAGLIPTIN
B01A	Antithrombotic agents	ANAGRELIDE
B01A	Antithrombotic agents	APIXABAN
B01A	Antithrombotic agents	ASPIRIN
B01A	Antithrombotic agents	CILOSTAZOL
B01A	Antithrombotic agents	CLOPIDOGREL
B01A	Antithrombotic agents	DABIGATRAN
B01A	Antithrombotic agents	DALTEPARIN
B01A	Antithrombotic agents	DIPYRIDAMOLE
B01A	Antithrombotic agents	ENOXAPARIN
B01A	Antithrombotic agents	HEPARIN
B01A	Antithrombotic agents	PRASUGREL
B01A	Antithrombotic agents	RIVAROXABAN
B01A	Antithrombotic agents	TICAGRELOR
B01A	Antithrombotic agents	VORAPAXAR
B01A	Antithrombotic agents	WARFARIN
C01D	Vasodilators used in cardiac diseases	ISOSORBIDE DINITRATE
C01D	Vasodilators used in cardiac diseases	ISOSORBIDE MONONITRATE
C01D	Vasodilators used in cardiac diseases	NICORANDIL
C01D	Vasodilators used in cardiac diseases	NITROGLYCERIN
C02	Antihypertensives	ALFUZOSIN
C02	Antihypertensives	CLONIDINE
C02	Antihypertensives	DOXAZOSIN
C02	Antihypertensives	HYDRALAZINE
C02	Antihypertensives	METHYLDOPA
C02	Antihypertensives	MINOXIDIL
C02	Antihypertensives	PRAZOSIN
C02	Antihypertensives	TERAZOSIN

C03	Diuretics	ACETAZOLAMIDE
C03	Diuretics	AMILORIDE
C03	Diuretics	AMILORIDE
C03	Diuretics	BENDROFLUMETHIAZIDE
C03	Diuretics	BUMETANIDE
C03	Diuretics	CHLOROTHIAZIDE
C03	Diuretics	CHLORTHALIDONE
C03	Diuretics	EPLERENONE
C03	Diuretics	FUROSEMIDE
C03	Diuretics	HYDROCHLOROTHIAZIDE
C03	Diuretics	INDAPAMIDE
C03	Diuretics	METOLAZONE
C03	Diuretics	SPIRONOLACTONE
C03	Diuretics	TRIAMTERENE
C07	Beta blocking agents	ACEBUTOLOL
C07	Beta blocking agents	ATENOLOL
C07	Beta blocking agents	BETAXOLOL
C07	Beta blocking agents	BISOPROLOL
C07	Beta blocking agents	CARVEDILOL
C07	Beta blocking agents	LABETALOL
C07	Beta blocking agents	METOPROLOL
C07	Beta blocking agents	NADOLOL
C07	Beta blocking agents	NEBIVOLOL
C07	Beta blocking agents	PINDOLOL
C07	Beta blocking agents	PROPRANOLOL
C07	Beta blocking agents	SOTALOL
C07	Beta blocking agents	TIMOLOL
C08	Calcium channel blockers	AMLODIPINE
C08	Calcium channel blockers	DILTIAZEM
C08	Calcium channel blockers	FELODIPINE
C08	Calcium channel blockers	ISRADIPINE
C08	Calcium channel blockers	NICARDIPINE
C08	Calcium channel blockers	NIFEDIPINE
C08	Calcium channel blockers	NIMODIPINE
C08	Calcium channel blockers	NISOLDIPINE
C08	Calcium channel blockers	VERAPAMIL
C09	Agents acting on the renin-angiotensin system	ALISKIREN
C09	Agents acting on the renin-angiotensin system	AZILSARTAN
C09	Agents acting on the renin-angiotensin system	BENAZEPRIL
C09	Agents acting on the renin-angiotensin system	CANDESARTAN

C09	Agents acting on the renin-angiotensin system	CAPTOPRIL
C09	Agents acting on the renin-angiotensin system	CILAZAPRIL
C09	Agents acting on the renin-angiotensin system	ENALAPRIL
C09	Agents acting on the renin-angiotensin system	EPROSARTAN
C09	Agents acting on the renin-angiotensin system	FOSINOPRIL
C09	Agents acting on the renin-angiotensin system	IRBESARTAN
C09	Agents acting on the renin-angiotensin system	LISINOPRIL
C09	Agents acting on the renin-angiotensin system	LOSARTAN
C09	Agents acting on the renin-angiotensin system	MOEXIPRIL
C09	Agents acting on the renin-angiotensin system	OLMESARTAN
C09	Agents acting on the renin-angiotensin system	PERINDOPRIL
C09	Agents acting on the renin-angiotensin system	QUINAPRIL
C09	Agents acting on the renin-angiotensin system	RAMIPRIL
C09	Agents acting on the renin-angiotensin system	TELMISARTAN
C09	Agents acting on the renin-angiotensin system	TRANDOLAPRIL
C09	Agents acting on the renin-angiotensin system	VALSARTAN
C10AA	HMG CoA reductase inhibitors	ATORVASTATIN
C10AA	HMG CoA reductase inhibitors	FLUVASTATIN
C10AA	HMG CoA reductase inhibitors	LOVASTATIN
C10AA	HMG CoA reductase inhibitors	PITAVASTATIN
C10AA	HMG CoA reductase inhibitors	PRAVASTATIN
C10AA	HMG CoA reductase inhibitors	ROSUVASTATIN
C10AA	HMG CoA reductase inhibitors	SIMVASTATIN
H03A	Thyroid preparations	LEVOTHYROXINE
H03A	Thyroid preparations	LIOTHYRONINE
H03A	Thyroid preparations	THYROID DESICCATED
L04	Immunosuppressants	ABATACEPT
L04	Immunosuppressants	ADALIMUMAB

L04	Immunosuppressants	AZATHIOPRINE
L04	Immunosuppressants	CYCLOSPORINE
L04	Immunosuppressants	ETANERCEPT
L04	Immunosuppressants	EVOLOCUMAB
L04	Immunosuppressants	GOLIMUMAB
L04	Immunosuppressants	HYDROXYCHLOROQUINE
L04	Immunosuppressants	INFLIXIMAB
L04	Immunosuppressants	LEFLUNOMIDE
L04	Immunosuppressants	METHOTREXATE
L04	Immunosuppressants	MYCOPHENOLATE MOFETIL
L04	Immunosuppressants	MYCOPHENOLIC ACID
L04	Immunosuppressants	NATALIZUMAB
L04	Immunosuppressants	PIMECROLIMUS TOPICAL
L04	Immunosuppressants	RITUXIMAB
L04	Immunosuppressants	SIROLIMUS
L04	Immunosuppressants	TACROLIMUS
L04	Immunosuppressants	TOFACITINIB
L04	Immunosuppressants	USTEKINUMAB
M01A	Antiinflammatory and antirheumatic products, non-steroids	ACECLOFENAC
M01A	Antiinflammatory and antirheumatic products, non-steroids	ACETAMINOPHEN
M01A	Antiinflammatory and antirheumatic products, non-steroids	ASPIRIN
M01A	Antiinflammatory and antirheumatic products, non-steroids	CELECOXIB
M01A	Antiinflammatory and antirheumatic products, non-steroids	DICLOFENAC
M01A	Antiinflammatory and antirheumatic products, non-steroids	DICLOFENAC
M01A	Antiinflammatory and antirheumatic products, non-steroids	ETODOLAC
M01A	Antiinflammatory and antirheumatic products, non-steroids	ETORICOXIB
M01A	Antiinflammatory and antirheumatic products, non-steroids	FLURBIPROFEN
M01A	Antiinflammatory and antirheumatic products, non-steroids	GLUCOSAMINE
M01A	Antiinflammatory and antirheumatic products, non-steroids	IBUPROFEN
M01A	Antiinflammatory and antirheumatic products, non-steroids	INDOMETHACIN
M01A	Antiinflammatory and antirheumatic products, non-steroids	KETOPROFEN

M01A	Antiinflammatory and antirheumatic products, non-steroids	KETOROLAC
M01A	Antiinflammatory and antirheumatic products, non-steroids	MEFENAMIC ACID
M01A	Antiinflammatory and antirheumatic products, non-steroids	MELOXICAM
M01A	Antiinflammatory and antirheumatic products, non-steroids	MISOPROSTOL
M01A	Antiinflammatory and antirheumatic products, non-steroids	NABUMETONE
M01A	Antiinflammatory and antirheumatic products, non-steroids	NAPROXEN
M01A	Antiinflammatory and antirheumatic products, non-steroids	OXAPROZIN
M01A	Antiinflammatory and antirheumatic products, non-steroids	PIROXICAM
M01A	Antiinflammatory and antirheumatic products, non-steroids	SULINDAC
M05B	Drugs affecting bone structure and mineralization	ALENDRONATE
M05B	Drugs affecting bone structure and mineralization	CALCIUM CARBONATE
M05B	Drugs affecting bone structure and mineralization	ETIDRONATE
M05B	Drugs affecting bone structure and mineralization	IBANDRONATE
M05B	Drugs affecting bone structure and mineralization	PAMIDRONATE
M05B	Drugs affecting bone structure and mineralization	RISEDRONATE
M05B	Drugs affecting bone structure and mineralization	ZOLEDRONIC
N02A	Opioids	BUPRENORPHINE
N02A	Opioids	CODEINE
N02A	Opioids	DIHYDROCODEINE
N02A	Opioids	HYDROCODONE
N02A	Opioids	HYDROMORPHONE
N02A	Opioids	METHADONE
N02A	Opioids	MORPHINE
N02A	Opioids	NALTREXONE
N02A	Opioids	OXYCODONE
N02A	Opioids	OXYMORPHONE
N02A	Opioids	TRAMADOL
N02C	Antimigraine preparations	ALMOTRIPTAN

N02C	Antimigraine preparations	CLONIDINE
N02C	Antimigraine preparations	ELETRIPTAN
N02C	Antimigraine preparations	ERENUMAB
N02C	Antimigraine preparations	ERGOTAMINE
N02C	Antimigraine preparations	FROVATRIPTAN
N02C	Antimigraine preparations	NARATRIPTAN
N02C	Antimigraine preparations	RIZATRIPTAN
N02C	Antimigraine preparations	SUMATRIPTAN
N02C	Antimigraine preparations	ZOLMITRIPTAN
N06A	Antidepressants	AMITRIPTYLINE
N06A	Antidepressants	ANTIDEPRESSANTS-UNSPECIFIED
N06A	Antidepressants	BREXPIPRAZOLE
N06A	Antidepressants	BUPROPION
N06A	Antidepressants	CARIPRAZINE
N06A	Antidepressants	CITALOPRAM
N06A	Antidepressants	CLOMIPRAMINE
N06A	Antidepressants	DESVENLAFAXINE
N06A	Antidepressants	DOXEPIN
N06A	Antidepressants	DULOXETINE
N06A	Antidepressants	ESCITALOPRAM
N06A	Antidepressants	FLUOXETINE
N06A	Antidepressants	FLUVOXAMINE
N06A	Antidepressants	IMIPRAMINE
N06A	Antidepressants	MIRTAZAPINE
N06A	Antidepressants	NORTRIPTYLINE
N06A	Antidepressants	PALIPERIDONE
N06A	Antidepressants	PAROXETINE
N06A	Antidepressants	PHENELZINE
N06A	Antidepressants	SERTRALINE
N06A	Antidepressants	TRAZODONE
N06A	Antidepressants	TRIMIPRAMINE
N06A	Antidepressants	VENLAFAXINE
N06A	Antidepressants	VORTIOXETINE
R03BA	Glucocorticoids	BECLOMETHASONE
R03BA	Glucocorticoids	BETAMETHASONE
R03BA	Glucocorticoids	BUDESONIDE
R03BA	Glucocorticoids	DEXAMETHASONE
R03BA	Glucocorticoids	FLUNISOLIDE
R03BA	Glucocorticoids	FLUTICASONE
R03BA	Glucocorticoids	HYDROCORTISONE
R03BA	Glucocorticoids	METHYLPREDNISOLONE
R03BA	Glucocorticoids	MOMETASONE
R03BA	Glucocorticoids	PREDNISOLONE
R03BA	Glucocorticoids	TRIAMCINOLONE

R06A	Antihistamines for systemic use	ACRIVASTINE
R06A	Antihistamines for systemic use	ALIMEMAZINE
R06A	Antihistamines for systemic use	AZELASTINE
R06A	Antihistamines for systemic use	CETIRIZINE
R06A	Antihistamines for systemic use	CHLORPHENAMINE
R06A	Antihistamines for systemic use	CLEMASTINE
R06A	Antihistamines for systemic use	CYCLIZINE
R06A	Antihistamines for systemic use	DESLORATADINE
R06A	Antihistamines for systemic use	DIPHENHYDRAMINE
R06A	Antihistamines for systemic use	FEXOFENADINE
R06A	Antihistamines for systemic use	KETOTIFEN
R06A	Antihistamines for systemic use	LEVOCETIRIZINE
R06A	Antihistamines for systemic use	LORATADINE
R06A	Antihistamines for systemic use	MIZOLASTINE
R06A	Antihistamines for systemic use	PROMETHAZINE
R06A	Antihistamines for systemic use	TERFENADINE

Table S3. STROBE-MR checklist of recommended items to address in reports of Mendelian randomization study

Item No.	Section	Checklist item	Section (paragraph number)
1	TITLE and ABSTRACT	Indicate Mendelian randomisation as the study's design in the title and/or the abstract if that is a main purpose of the study	Title page & Abstract page
	INTRODUCTION		
2	Background	Explain the scientific background and rationale for the reported study. What is the exposure? Is a potential causal relationship between exposure and outcome plausible? Justify why MR is a helpful method to address the study question	Introduction (paragraphs 1-5)
3	Objectives	State specific objectives clearly, including pre-specified causal hypotheses (if any). State that MR is a method that, under specific assumptions, intends to estimate causal effects	Introduction (paragraph 5)
	METHODS		
4	Study design and data sources	Present key elements of the study design early in the article. Consider including a table listing sources of data for all phases of the study. For each data source contributing to the analysis, describe the following:	
	a)	Setting: Describe the study design and the underlying population, if possible. Describe the setting, locations, and relevant dates, including periods of recruitment, exposure, follow-up, and data collection, when available.	Methods and materials (GWAS data for medication use & GWAS data for cancer & IVs selection for MR & Replication and meta-analysis)
	b)	Participants: Give the eligibility criteria, and the sources and methods of selection of participants. Report the sample size, and whether any power or sample size calculations were carried out prior to the main analysis	Methods and materials (GWAS data for medication use & GWAS data for cancer & Replication and meta-analysis) & Table1 &

			TableS5 & TableS7 & TableS16 & TableS17
	c)	Describe measurement, quality control and selection of genetic variants	Methods and materials (IVs selection for MR)
	d)	For each exposure, outcome, and other relevant variables, describe methods of assessment and diagnostic criteria for diseases	Methods and materials (GWAS data for medication use & GWAS data for cancer & Replication and meta-analysis)
	e)	Provide details of ethics committee approval and participant informed consent, if relevant	Methods and materials (Ethics statement)
5	Assumptions	Explicitly state the three core IV assumptions for the main analysis (relevance, independence and exclusion restriction) as well assumptions for any additional or sensitivity analysis	Methods and materials (MR analysis & Statistical analysis in MR paragraph 1-2 & Replication and meta-analysis & Genetic correlation and direction validation)
6	Statistical methods: main analysis	Describe statistical methods and statistics used	
	a)	Describe how quantitative variables were handled in the analyses (i.e., scale, units, model)	Methods and materials (GWAS data for medication use)
	b)	Describe how genetic variants were handled in the analyses and, if applicable, how their weights were selected	Methods and materials (IVs selection for MR) & TableS17
	c)	Describe the MR estimator (e.g. two-stage least squares, Wald ratio) and related statistics. Detail the included covariates and, in case of two-sample MR, whether the same covariate set was used for	Methods and materials (Statistical analysis in MR

		adjustment in the two samples	paragraph 1)
	d)	Explain how missing data were addressed	N/A
	e)	If applicable, indicate how multiple testing was addressed	NA
7	Assessment of assumptions	Describe any methods or prior knowledge used to assess the assumptions or justify their validity	Methods and materials (IVs selection for MR & Statistical analysis in MR)
8	Sensitivity analyses and additional analyses	Describe any sensitivity analyses or additional analyses performed (e.g. comparison of effect estimates from different approaches, independent replication, bias analytic techniques, validation of instruments, simulations)	Methods and materials (Statistical analysis in MR paragraph 1-2 & Replication and meta-analysis & Genetic correlation and direction validation)
9	Software and pre-registration		
	a)	Name statistical software and package(s), including version and settings used	Methods and materials (MR analysis)
	b)	State whether the study protocol and details were pre-registered (as well as when and where)	N/A
	RESULTS		
10	Descriptive data		
	a)	Report the numbers of individuals at each stage of included studies and reasons for exclusion. Consider use of a flow-diagram	Methods and materials (IVs selection for MR & Statistical analysis in MR paragraph 2) & TableS13 & TableS14
	b)	Report summary statistics for phenotypic exposure(s), outcome(s) and other relevant variables (e.g. means, SDs, proportions)	Methods and materials (GWAS data for medication use & GWAS data for cancer

			& Replication and meta-analysis) & Table1 & TableS5 & TableS7
	c)	If the data sources include meta-analyses of previous studies, provide the assessments of heterogeneity across these studies	N/A
	d)	For two-sample Mendelian randomisation:<br>i. Provide justification of the similarity of the genetic variant-exposure associations between the exposure and outcome samples<br>ii. Provide information on the number of individuals who were in both samples for the exposure and for the outcome	N/A
11	Main results		
	a)	Report the associations between genetic variant and exposure, and between genetic variant and outcome, preferably on an interpretable scale	Results (MR analysis & Replication and meta-analysis) & TableS15 & TableS16 & TableS17 & TableS18
	b)	Report MR estimates of the relationship between exposure and outcome, and the measures of uncertainty from the MR analysis, on an interpretable scale, such as odds ratio or relative risk per SD difference	Results (MR analysis & Replication and meta-analysis) & TableS16 & TableS19 & TableS20
	c)	If relevant, consider translating estimates of relative risk into absolute risk for a meaningful time period	N/A
	d)	Consider plots to visualize results (e.g. forest plot, scatterplot of associations between genetic variants and outcome versus between genetic variants and exposure)	Figures 3-6
12	Assessment of assumptions		
	a)	Report the assessment of the validity of the assumptions	Results (MR analysis & Replication and meta-analysis)

			& TableS16 & TableS18 & TableS19 & TableS20
	b)	Report any additional statistics (e.g., assessments of heterogeneity across genetic variants, such as I2, Q statistic or E-value)	TableS16 & TableS17 & TableS18 & TableS19 & TableS20
13	Sensitivity analyses and additional analyses		
	a)	Report any sensitivity analyses to assess the robustness of the main results to violations of the assumptions	Results (MR analysis & Replication and meta-analysis) & TableS16 & TableS18 & TableS19 & TableS20
	b)	Report results from other sensitivity analyses or additional analyses	TableS16 & TableS17 & TableS18 & TableS19 & TableS20
	c)	Report any assessment of direction of causal relationship (e.g., bidirectional MR)	TableS19 & TableS20
	d)	When relevant, report and compare with estimates from non-MR analyses	TableS9-S12
	e)	Consider additional plots to visualize results (e.g., leave-one-out analyses)	Figure S5-S6
	DISCUSSION		
14	Key results	Summarize key results with reference to study objectives	Discussion (paragraph 1)
15	Limitations	Discuss limitations of the study, taking into account the validity of the IV assumptions, other sources of potential bias, and imprecision. Discuss both direction and magnitude of any potential bias and any efforts to address them	Discussion (paragraph 6)
16	Interpretation		
	a)	Meaning: Give a cautious overall interpretation of results in the context of their limitations and in comparison with other studies	Discussion (paragraph 1-6)
	b)	Mechanism: Discuss underlying biological mechanisms that could drive a potential causal relationship	Discussion (paragraph 2-6)

		between the investigated exposure and the outcome, and whether the gene-environment equivalence assumption is reasonable.<br>Use causal language carefully, clarifying that IV estimates may provide causal effects only under certain assumptions	
	c)	Clinical relevance: Discuss whether the results have clinical or public policy relevance, and to what extent they inform effect sizes of possible interventions	Discussion (paragraph 7)
17	Generalizability	Discuss the generalizability of the study results (a) to other populations, (b) across other exposure periods/timings, and (c) across other levels of exposure	Discussion (paragraph 6)
	OTHER INFORMATION		
18	Funding	Describe sources of funding and the role of funders in the present study and, if applicable, sources of funding for the databases and original study or studies on which the present study is based	Funding section
19	Data and data sharing	Provide the data used to perform all analyses or report where and how the data can be accessed, and reference these sources in the article. Provide the statistical code needed to reproduce the results in the article,or report whether the code is publicly accessible and if so, where	Data share statement & Table S5 & Table S7
20	Conflicts of Interest	All authors should declare all potential conflicts of interest	Declaration of interests section

Table S4. Active ingredients and ATC code of medications in the UK Biobank

Medication	Category	Coding reported by UK Biobank	Medication ATC code	Drug name
Drugs for peptic ulcer and GORD	omeprazole	1140865634	A02BC01	Omeprazole
Drugs for peptic ulcer and GORD	lansoprazole	1140864752	A02BC03	Lansoprazole
Drugs for peptic ulcer and GORD	ranitidine	1140879406	A02BA02	Ranitidine
Drugs for peptic ulcer and GORD	gaviscon liquid	1140865354	A02BX13	Sodium Bicarbonate |Calcium Carbonate |Alginic Acid
Drugs for peptic ulcer and GORD	esomeprazole	1141177526	A02BC05	Esomeprazole
Drugs for peptic ulcer and GORD	pantoprazole	1140929012	A02BC02	Pantoprazole
Drugs for peptic ulcer and GORD	rabeprazole sodium	1141168584	A02BC04	Rabeprazole
Drugs for peptic ulcer and GORD	nexium 20mg tablet	1141177532	A02BC05	Esomeprazole
Drugs for peptic ulcer and GORD	cimetidine	1140865426	A02BA01	Cimetidine
Drugs for peptic ulcer and GORD	losec 10mg capsule	1140909578	A02BC01	Omeprazole
Drugs for peptic ulcer and GORD	zoton 15mg capsule	1140923688	A02BC03	Lansoprazole
Drugs for peptic ulcer and GORD	pariet 10mg e/c tablet	1141168590	A02BC04	Rabeprazole
Drugs for peptic ulcer and GORD	zantac 75 tablet	1140916980	A02BA02	Ranitidine
Drugs for peptic ulcer and GORD	protium 20mg e/c tablet	1141164616	A02BC02	Pantoprazole
Drugs for peptic ulcer and GORD	nizatidine	1140865618	A02BA04	Nizatidine
Drugs for peptic ulcer and GORD	gavilast-p 75mg tablet	1141188426	A02BA	Ranitidine
Drugs for peptic ulcer and GORD	sucralfate	1140865536	A02BX02	Sucralfate
Drugs for peptic ulcer and GORD	misoprostol	1140865628	A02BB01 |G02AD06	Misoprostol
Drugs for peptic ulcer and GORD	antepsin 1g tablet	1140865538	A02BX02	Sucralfate
Drugs for peptic ulcer and GORD	famotidine	1140865608	A02BA03	Famotidine
Drugs for peptic ulcer and GORD	tagamet 100 tablet	1140909500	A02BA01	Cimetidine

Drugs for peptic ulcer and GORD	pepcid ac indigestion tablet	1140909496	A02BA03	Famotidine
Drugs for peptic ulcer and GORD	acidex oral suspension	1141172224	A02BX13	Sodium Bicarbonate |Calcium Carbonate |Alginic Acid
Drugs for peptic ulcer and GORD	topal tablet	1140865370	A02BX13	Aluminum Hydroxide |Magnesium Carbonate |Alginic Acid
Drugs used in diabetes	insulin product	1140883066	A10A	Insulin
Drugs used in diabetes	metformin	1140884600	A10BA02	Metformin
Drugs used in diabetes	glucophage 500mg tablet	1140874686	A10BA02	Metformin
Drugs used in diabetes	glibenclamide	1140874718	A10BB01	Glyburide
Drugs used in diabetes	tolbutamide	1140874674	A10BB03 |V04CA01	Tolbutamide
Drugs used in diabetes	glipizide	1140874646	A10BB07	Glipizide
Drugs used in diabetes	gliclazide	1140874744	A10BB09	Gliclazide
Drugs used in diabetes	glyclizide	1140910566	A10BB09	Gliclazide
Drugs used in diabetes	diamicron 80mg tablet	1140874746	A10BB09	Gliclazide
Drugs used in diabetes	glimepiride	1141152590	A10BB12	Glimepiride
Drugs used in diabetes	amaryl 1mg tablet	1141156984	A10BB12	Glimepiride
Drugs used in diabetes	rosiglitazone 1mg / metformin 500mg tablet	1141189090	A10BD03	Metformin |Rosiglitazone
Drugs used in diabetes	avandamet 1mg / 500mg tablet	1141189094	A10BD03	Metformin |Rosiglitazone
Drugs used in diabetes	acarbose	1140868902	A10BF01	Acarbose
Drugs used in diabetes	rosiglitazone	1141177600	A10BG02	Rosiglitazone
Drugs used in diabetes	avandia 4mg tablet	1141177606	A10BG02	Rosiglitazone
Drugs used in diabetes	pioglitazone	1141171646	A10BG03	Pioglitazone
Drugs used in diabetes	actos 15mg tablet	1141171652	A10BG03	Pioglitazone
Drugs used in diabetes	repaglinide	1141168660	A10BX02	Repaglinide
Drugs used in diabetes	nateglinide	1141173882	A10BX03	Nateglinide

Antithrombotic agents	aspirin	1140868226	A01AD05 |B01AC06 |N02BA01	Acetylsalicylic Acid
Antithrombotic agents	warfarin	1140888266	B01AA03	Warfarin
Antithrombotic agents	clopidogrel	1141168318	B01AC04	Clopidogrel
Antithrombotic agents	aspirin 75mg tablet	1140861806	A01AD05 |B01AC06 |N02BA01	Acetylsalicylic Acid
Antithrombotic agents	dipyridamole	1140861778	B01AC07	Dipyridamole
Antithrombotic agents	sodium warfarin	1140910832	B01AA03	Warfarin
Antithrombotic agents	persantin 25mg tablet	1140861780	B01AC07	Dipyridamole
Antithrombotic agents	plavix 75mg tablet	1141168322	B01AC04	Clopidogrel
Antithrombotic agents	asasantin retard m/r capsule	1141167848	B01AC30	Acetylsalicylic Acid |Dipyridamole
Antithrombotic agents	micropirin 75mg e/c tablet	1141177826	B01AC06	Acetylsalicylic Acid
Antithrombotic agents	dipyridamole+aspirin	1141167844	B01AC30	Acetylsalicylic Acid |Dipyridamole
Antithrombotic agents	cilostazol	1141181150	B01AC23	Cilostazol
Antithrombotic agents	clexane 20mg/0.2ml prefilled syringe	1140861594	B01AB05	Enoxaparin
Antithrombotic agents	sinthrome 1mg tablet	1140861698	B01AA07	Acenocoumarol
Antithrombotic agents	heparin	1140881842	B01AB01 |C05BA03 |S01XA14	Heparin
Antithrombotic agents	phenindione	1140861702	B01AA02	Phenindione
Antithrombotic agents	enoxaparin	1140861588	B01AB05	Enoxaparin
Antithrombotic agents	marevan 0.5mg tablet	1141164760	B01AA03	Warfarin
Antithrombotic agents	fragmin 10000iu/1ml injection	1140861584	B01AB04	Dalteparin
Antithrombotic agents	tinzaparin	1140888206	B01AB10	Tinzaparin
Antithrombotic agents	pletal 100mg tablet	1141181154	B01AC23	Cilostazol
Vasodilators used in cardiac	isosorbide mononitrate	1140860954	C01DA14	Isosorbide Mononitrate

diseases				
Vasodilators used in cardiac diseases	nicorandil	1140910766	C01DX16	Nicorandil
Vasodilators used in cardiac diseases	glyceryl trinitrate	1140860834	C01DA02 |C05AE01	Nitroglycerin
Vasodilators used in cardiac diseases	gtn - glyceryl trinitrate	1140923670	C01DA02	Nitroglycerin
Vasodilators used in cardiac diseases	nitrolingual 400micrograms spray	1140860840	C01DA02	Nitroglycerin
Vasodilators used in cardiac diseases	glyceryl trinitrate product	1141157252	C01DA02 |C05AE01	Nitroglycerin
Vasodilators used in cardiac diseases	ismn - isosorbide mononitrate	1140888762	C01DA14	Isosorbide Mononitrate
Vasodilators used in cardiac diseases	isotard 25xl m/r tablet	1141168108	C01DA14	Isosorbide Mononitrate
Vasodilators used in cardiac diseases	imdur 60mg durule	1140860976	C01DA14	Isosorbide Mononitrate
Vasodilators used in cardiac diseases	ismo - isosorbide mononitrate	1140910512	C01DA14	Isosorbide Mononitrate
Vasodilators used in cardiac diseases	monomax xl 60mg m/r tablet	1141172698	C01DA14	Isosorbide Mononitrate
Vasodilators used in cardiac diseases	nitromin 400micrograms cfc-free spray	1141145630	C01DA02	Nitroglycerin
Vasodilators used in cardiac diseases	isosorbide dinitrate	1140861008	C01DA08 |C05AE02	Isosorbide Dinitrate
Vasodilators used in cardiac	isosorbide mononitrate product	1141157254	C01DA14	Isosorbide Mononitrate

diseases				
Vasodilators used in cardiac diseases	xismox xl 60 m/r tablet	1141173888	C01DA14	Isosorbide Mononitrate
Vasodilators used in cardiac diseases	monomil xl 60mg m/r tablet	1141189776	C01DA14	Isosorbide Mononitrate
Vasodilators used in cardiac diseases	monomax sr 40 m/r capsule	1140923788	C01DA14	Isosorbide Mononitrate
Vasodilators used in cardiac diseases	gtn 300micrograms sublingual tablet	1140860838	C01DA02	Nitroglycerin
Vasodilators used in cardiac diseases	ikorel 10mg tablet	1140910772	C01DX16	Nicorandil
Vasodilators used in cardiac diseases	suscard 1mg m/r buccal tablet	1140860846	C01DA02	Nitroglycerin
Vasodilators used in cardiac diseases	glytrin 400micrograms spray	1140861000	C01DA02	Nitroglycerin
Vasodilators used in cardiac diseases	elantan 10 tablet	1140860982	C01DA14	Isosorbide Mononitrate
Vasodilators used in cardiac diseases	monosorb xl 60 m/r tablet	1141162550	C01DA14	Isosorbide Mononitrate
Vasodilators used in cardiac diseases	isib 20mg tablet	1140860994	C01DA14	Isosorbide Mononitrate
Vasodilators used in cardiac diseases	nitroglycerin	1140910570	C01DA02 |C05AE01	Nitroglycerin
Vasodilators used in cardiac diseases	glyceryl trinitrate patch	1140927544	C01DA02	Nitroglycerin
Vasodilators used in cardiac	chemydur 60xl m/r tablet	1141166858	C01DA14	Isosorbide Mononitrate

diseases				
Vasodilators used in cardiac diseases	ismo 10 tablet	1140860972	C01DA14	Isosorbide Mononitrate
Vasodilators used in cardiac diseases	gtn - glyceryl trinitrate patch	1140927548	C01DA02	Nitroglycerin
Antihypertensives	methyldopa	1140860470	C02AB	Methyldopa
Antihypertensives	clonidine	1140883468	C02AC01 |N02CX02 |S01EA04	Clonidine
Antihypertensives	moxonidine	1140928284	C02AC05	Moxonidine
Antihypertensives	physiotens 200micrograms tablet	1140928290	C02AC05	Moxonidine
Antihypertensives	prazosin	1140879794	C02CA01	Prazosin
Antihypertensives	hypovase 500mcg tablet	1140860580	C02CA01	Prazosin
Antihypertensives	indoramin	1140879782	C02CA02	Indoramin
Antihypertensives	doxazosin	1140879778	C02CA04	Doxazosin
Antihypertensives	cardura 1mg tablet	1140860690	C02CA04	Doxazosin
Antihypertensives	doxadura 1mg tablet	1141194372	C02CA04	Doxazosin
Antihypertensives	hydralazine	1140888686	C02DB02	Hydralazine
Antihypertensives	minoxidil	1140860532	C02DC01 |D11AX01	Minoxidil
Diuretics	bendroflumethiazide	1141194794	C03AA01	Bendroflumethiazide
Diuretics	bendrofluazide	1140866122	C03AA01	Bendroflumethiazide
Diuretics	bzt - bendrofluazide	1140910442	C03AA01	Bendroflumethiazide
Diuretics	hydrochlorothiazide	1140866162	C03AA03	Hydrochlorothiazide
Diuretics	cyclopenthiazide	1140866156	C03AA07	Cyclopenthiazide
Diuretics	bendroflumethiazide+potassium 2.5mg/7.7mmol m/r tablet	1141194800	C03AB01	Bendroflumethiazide |Potassium
Diuretics	bendrofluazide+potassium	1140866450	C03AB01	Bendroflumethiazide |Potassium

	2.5mg/7.7mmol m/r tablet			
Diuretics	chlortalidone	1140909706	C03BA04	Chlorthalidone
Diuretics	hygroton 50mg tablet	1140866146	C03BA04	Chlorthalidone
Diuretics	metolazone	1140866092	C03BA08	Metolazone
Diuretics	xipamide	1140866108	C03BA10	Xipamide
Diuretics	indapamide	1140866078	C03BA11	Indapamide
Diuretics	natrilix sr 1.5mg m/r tablet	1141146378	C03BA11	Indapamide
Diuretics	furosemide	1140909708	C03CA01	Furosemide
Diuretics	frusemide	1140866116	C03CA01	Furosemide
Diuretics	frumil tablet	1140866406	C03CA01	Amiloride |Furosemide
Diuretics	bumetanide	1140866280	C03CA02	Bumetanide
Diuretics	burinex a tablet	1140866356	C03CA02	Bumetanide
Diuretics	torasemide	1140888496	C03CA04	Torasemide
Diuretics	spironolactone	1140866236	C03DA01	Spironolactone
Diuretics	spirolone 25mg tablet	1140866318	C03DA01	Spironolactone
Diuretics	eplerenone	1141201244	C03DA04	Eplerenone
Diuretics	amiloride	1140888512	C03DB01	Amiloride
Diuretics	co-amilozide	1140923276	C03EA01	Amiloride |Hydrochlorothiazide
Diuretics	dyazide tablet	1140866402	C03EA01	Triamterene |Hydrochlorothiazide
Diuretics	moduretic tablet	1140866420	C03EA01	Amiloride |Hydrochlorothiazide
Diuretics	co-triamterzide	1140923272	C03EA01	Triamterene |Hydrochlorothiazide
Diuretics	moduret 25 tablet	1140866416	C03EA01	Amiloride |Hydrochlorothiazide
Diuretics	navispare tablet	1140866352	C03EA07	Amiloride |Cyclopenthiazide
Diuretics	co-amilofruse	1140923402	C03EB01	Amiloride |Furosemide
Beta blocking agents	oxprenolol	1140879830	C07AA02	Oxprenolol
Beta blocking agents	pindolol	1140860292	C07AA03	Pindolol

Beta blocking agents	propranolol	1140879842	C07AA05	Propranolol
Beta blocking agents	half-inderal la 80mg m/r capsule	1140866800	C07AA05	Propranolol
Beta blocking agents	inderal 10mg tablet	1140866804	C07AA05	Propranolol
Beta blocking agents	bedranol 10mg tablet	1140851556	C07AA05	Propranolol
Beta blocking agents	half beta-prograne 80mg m/r capsule	1140866802	C07AA05	Propranolol
Beta blocking agents	timolol	1140879866	C07AA06 |S01ED01	Timolol
Beta blocking agents	sotalol	1140879854	C07AA07	Sotalol
Beta blocking agents	beta-cardone 40mg tablet	1140860304	C07AA07	Sotalol
Beta blocking agents	nadolol	1140860192	C07AA12	Nadolol
Beta blocking agents	carteolol	1140879822	C07AA15 |S01ED05	Carteolol
Beta blocking agents	prindolol	1140910614	C07AA17	Pindolol
Beta blocking agents	metoprolol	1140879818	C07AB02	Metoprolol
Beta blocking agents	atenolol	1140866738	C07AB03	Atenolol
Beta blocking agents	tenormin 25 tablet	1140866756	C07AB03	Atenolol
Beta blocking agents	acebutolol	1140866724	C07AB04	Acebutolol
Beta blocking agents	betaxolol	1140879758	C07AB05 |S01ED02	Betaxolol
Beta blocking agents	bisoprolol	1140879760	C07AB07	Bisoprolol
Beta blocking agents	cardicor 1.25mg tablet	1141171152	C07AB07	Bisoprolol
Beta blocking agents	celiprolol	1140879762	C07AB08	Celiprolol
Beta blocking agents	celectol 200mg tablet	1140860498	C07AB08	Celiprolol
Beta blocking agents	nebivolol	1141164276	C07AB12	Nebivolol
Beta blocking agents	nebilet 5mg tablet	1141164280	C07AB12	Nebivolol
Beta blocking agents	labetalol	1140879824	C07AG01	Labetalol
Beta blocking agents	carvedilol	1140909368	C07AG02	Carvedilol
Beta blocking agents	propranolol	1140860418	C07BA05	Bendroflumethiazide |Propranolol

	hydrochloride+bendrofluazide 80mg/2.5mg capsule			
Beta blocking agents	sotalol hydrochloride+hydrochlorothiazide 80mg/12.5mg tablet	1140860332	C07BA07	Sotalol |Hydrochlorothiazide
Beta blocking agents	atenolol+bendroflumethiazide	1141194810	C07BB03	Atenolol |Bendroflumethiazide
Beta blocking agents	atenolol+bendrofluazide	1141146126	C07BB03	Atenolol |Bendroflumethiazide
Beta blocking agents	atenolol+chlortalidone	1141180778	C07BB03	Chlorthalidone |Atenolol
Beta blocking agents	bisoprolol fumarate+hydrochlorothiazide 10mg/6.25mg tablet	1140864950	C07BB07	Bisoprolol |Hydrochlorothiazide
Beta blocking agents	metoprolol tartrate+chlorthalidone 100mg/12.5mg tablet	1140860308	C07CB02	Metoprolol |Chlorthalidone
Beta blocking agents	co-tenidone	1140923336	C07CB03	Chlorthalidone |Atenolol
Beta blocking agents	tenoret 50 tablet	1140860324	C07CB03	Chlorthalidone |Atenolol
Beta blocking agents	tenoretic tablet	1140860328	C07CB03	Chlorthalidone |Atenolol
Beta blocking agents	kalten capsule	1140860398	C07DB01	Atenolol |Amiloride |Hydrochlorothiazide
Beta blocking agents	tenif capsule	1140860358	C07FB03	Atenolol |Nifedipine
Beta blocking agents	beta-adalat capsule	1140860356	C07FB03	Atenolol |Nifedipine
Calcium channel blockers	diltiazem	1140879806	C05AE03 |C08DB01	Diltiazem
Calcium channel blockers	amlodipine	1140879802	C08CA01	Amlodipine
Calcium channel blockers	istin 5mg tablet	1140861202	C08CA01	Amlodipine
Calcium channel blockers	amlostin 5mg tablet	1141200400	C08CA01	Amlodipine
Calcium channel blockers	felodipine	1140888646	C08CA02	Felodipine
Calcium channel blockers	cardioplen xl 5mg m/r tablet	1141199858	C08CA02	Felodipine

Calcium channel blockers	vascalpha 5mg m/r tablet	1141190160	C08CA02	Felodipine
Calcium channel blockers	felendil xl 5mg m/r tablet	1141188836	C08CA02	Felodipine
Calcium channel blockers	plendil 2.5mg m/r tablet	1140928212	C08CA02	Felodipine
Calcium channel blockers	felotens xl 5mg m/r tablet	1141188152	C08CA02	Felodipine
Calcium channel blockers	neofel xl 5mg m/r tablet	1141200782	C08CA02	Felodipine
Calcium channel blockers	felogen xl 5mg m/r tablet	1141188576	C08CA02	Felodipine
Calcium channel blockers	cabren 2.5mg m/r tablet	1141187094	C08CA02	Felodipine
Calcium channel blockers	nicardipine	1140879810	C08CA04	Nicardipine
Calcium channel blockers	cardene 20mg capsule	1140861176	C08CA04	Nicardipine
Calcium channel blockers	nifedipine	1140861088	C08CA05	Nifedipine
Calcium channel blockers	adalat 5mg capsule	1140861090	C08CA05	Nifedipine
Calcium channel blockers	coracten sr 10mg m/r capsule	1140861120	C08CA05	Nifedipine
Calcium channel blockers	adalate 10mg capsule	1140881702	C08CA05	Nifedipine
Calcium channel blockers	adipine mr 10 m/r tablet	1140923572	C08CA05	Nifedipine
Calcium channel blockers	fortipine la40 m/r tablet	1141145870	C08CA05	Nifedipine
Calcium channel blockers	nifedipress mr 10 m/r tablet	1141157140	C08CA05	Nifedipine
Calcium channel blockers	tensipine mr 10 m/r tablet	1140927940	C08CA05	Nifedipine
Calcium channel blockers	lacidipine	1140861276	C08CA09	Lacidipine
Calcium channel blockers	motens 2mg tablet	1140861282	C08CA09	Lacidipine
Calcium channel blockers	lercanidipine	1141153026	C08CA13	Lercanidipine
Calcium channel blockers	zanidip 10mg tablet	1141153032	C08CA13	Lercanidipine
Calcium channel blockers	verapamil	1140888510	C08DA01	Verapamil
Calcium channel blockers	securon 40mg tablet	1140866466	C08DA01	Verapamil
Calcium channel blockers	half securon sr 120mg m/r tablet	1140866460	C08DA01	Verapamil
Calcium channel blockers	univer 120mg m/r capsule	1140881692	C08DA01	Verapamil
Calcium channel blockers	vertab sr 240 m/r tablet	1141169710	C08DA01	Verapamil

Calcium channel blockers	tildiem 60mg m/r tablet	1140861128	C08DB01	Diltiazem
Calcium channel blockers	adizem-60 m/r tablet	1140861138	C08DB01	Diltiazem
Calcium channel blockers	adizem-xl plus m/r capsule	1140926780	C08DB01	Diltiazem
Calcium channel blockers	dilzem sr 60mg long acting m/r capsule	1140861166	C08DB01	Diltiazem
Calcium channel blockers	slozem 120mg m/r capsule	1140911698	C08DB01	Diltiazem
Calcium channel blockers	angitil sr 90 m/r capsule	1140917428	C08DB01	Diltiazem
Calcium channel blockers	viazem xl 120mg m/r capsule	1141151474	C08DB01	Diltiazem
Calcium channel blockers	zemtard 120 xl m/r capsule	1141167832	C08DB01	Diltiazem
Calcium channel blockers	calcicard 60mg tablet	1140851730	C08DB01	Diltiazem
Calcium channel blockers	diltiazem hcl+hydrochlorothiazide 150mg/12.5mg m/r capsule	1140926778	C08GA	Diltiazem |Hydrochlorothiazide
Agents acting on the renin-angiotensin system	captopril	1140860750	C09AA01	Captopril
Agents acting on the renin-angiotensin system	capoten 12.5mg tablet	1140860758	C09AA01	Captopril
Agents acting on the renin-angiotensin system	enalapril	1140888552	C09AA02	Enalapril
Agents acting on the renin-angiotensin system	innovace 2.5mg tablet	1140860776	C09AA02	Enalapril
Agents acting on the renin-angiotensin system	lisinopril	1140860696	C09AA03	Lisinopril
Agents acting on the renin-angiotensin system	zestril 2.5mg tablet	1140860714	C09AA03	Lisinopril
Agents acting on the renin-angiotensin system	perindopril	1140888560	C09AA04	Perindopril

Agents acting on the renin-angiotensin system	coversyl 2mg tablet	1140860802	C09AA04	Perindopril
Agents acting on the renin-angiotensin system	ramipril	1140860806	C09AA05	Ramipril
Agents acting on the renin-angiotensin system	tritace 1.25mg tablet	1141188408	C09AA05	Ramipril
Agents acting on the renin-angiotensin system	lopace 2.5mg capsule	1141199940	C09AA05	Ramipril
Agents acting on the renin-angiotensin system	quinapril	1140860728	C09AA06	Quinapril
Agents acting on the renin-angiotensin system	accupro 5mg tablet	1140881706	C09AA06	Quinapril
Agents acting on the renin-angiotensin system	cilazapril	1140860882	C09AA08	Cilazapril
Agents acting on the renin-angiotensin system	fosinopril	1140888556	C09AA09	Fosinopril
Agents acting on the renin-angiotensin system	trandolapril	1140860904	C09AA10	Trandolapril
Agents acting on the renin-angiotensin system	gopten 500micrograms capsule	1140860912	C09AA10	Trandolapril
Agents acting on the renin-angiotensin system	imidapril hydrochloride	1141164148	C09AA16	Imidapril
Agents acting on the renin-angiotensin system	capozide tablet	1140881714	C09BA01	Hydrochlorothiazide |Captopril
Agents acting on the renin-angiotensin system	enalapril maleate+hydrochlorothiazide	1140860790	C09BA02	Enalapril |Hydrochlorothiazide

	20mg/12.5mg tablet			
Agents acting on the renin-angiotensin system	innozide tablet	1140860784	C09BA02	Enalapril |Hydrochlorothiazide
Agents acting on the renin-angiotensin system	lisinopril+hydrochlorothiazide 10mg/12.5mg tablet	1140864952	C09BA03	Lisinopril |Hydrochlorothiazide
Agents acting on the renin-angiotensin system	zestoretic 10 tablet	1140864618	C09BA03	Lisinopril |Hydrochlorothiazide
Agents acting on the renin-angiotensin system	carace 10 plus tablet	1140864910	C09BA03	Lisinopril |Hydrochlorothiazide
Agents acting on the renin-angiotensin system	perindopril+indapamide	1141180592	C09BA04	Perindopril |Indapamide
Agents acting on the renin-angiotensin system	coversyl plus 4mg/1.25mg tablet	1141180598	C09BA04	Perindopril |Indapamide
Agents acting on the renin-angiotensin system	felodipine+ramipril	1141165470	C09BB05	Ramipril |Felodipine
Agents acting on the renin-angiotensin system	triapin mite 2.5mg/2.5mg tablet	1141165476	C09BB05	Ramipril |Felodipine
Agents acting on the renin-angiotensin system	losartan	1140916356	C09CA01	Losartan
Agents acting on the renin-angiotensin system	cozaar 25mg tablet	1141179974	C09CA01	Losartan
Agents acting on the renin-angiotensin system	eprosartan	1141171336	C09CA02	Eprosartan
Agents acting on the renin-angiotensin system	teveten 300mg tablet	1141171344	C09CA02	Eprosartan
Agents acting on the	valsartan	1141145660	C09CA03	Valsartan

renin-angiotensin system				
Agents acting on the renin-angiotensin system	diovan 40mg capsule	1141145668	C09CA03	Valsartan
Agents acting on the renin-angiotensin system	irbesartan	1141152998	C09CA04	Irbesartan
Agents acting on the renin-angiotensin system	aprovel 75mg tablet	1141153006	C09CA04	Irbesartan
Agents acting on the renin-angiotensin system	candesartan cilexetil	1141156836	C09CA06	Candesartan
Agents acting on the renin-angiotensin system	amias 2mg tablet	1141156846	C09CA06	Candesartan
Agents acting on the renin-angiotensin system	telmisartan	1141166006	C09CA07	Telmisartan
Agents acting on the renin-angiotensin system	micardis 20mg tablet	1141172492	C09CA07	Telmisartan
Agents acting on the renin-angiotensin system	olmesartan	1141193282	C09CA08	Olmesartan
Agents acting on the renin-angiotensin system	olmetec 10mg tablet	1141193346	C09CA08	Olmesartan
Agents acting on the renin-angiotensin system	losartan potassium+hydrochlorothiazide 50mg/12.5mg tablet	1141151016	C09DA01	Losartan |Hydrochlorothiazide
Agents acting on the renin-angiotensin system	cozaar-comp 50mg/12.5mg tablet	1141151018	C09DA01	Losartan |Hydrochlorothiazide
Agents acting on the renin-angiotensin system	co-diovan 80mg/12.5mg tablet	1141201040	C09DA03	Valsartan |Hydrochlorothiazide

Agents acting on the renin-angiotensin system	valsartan+hydrochlorothiazide 80mg/12.5mg tablet	1141201038	C09DA03	Valsartan |Hydrochlorothiazide
Agents acting on the renin-angiotensin system	coaprovel 150mg/12.5mg tablet	1141172686	C09DA04	Hydrochlorothiazide |Irbesartan
Agents acting on the renin-angiotensin system	irbesartan+hydrochlorothiazide 150mg/12.5mg tablet	1141172682	C09DA04	Hydrochlorothiazide |Irbesartan
Agents acting on the renin-angiotensin system	micardisplus 40mg/12.5mg tablet	1141187790	C09DA07	Telmisartan |Hydrochlorothiazide
HMG CoA reductase inhibitors	simvastatin	1140861958	C10AA01	Simvastatin
HMG CoA reductase inhibitors	atorvastatin	1141146234	C10AA05	Atorvastatin
HMG CoA reductase inhibitors	lipitor 10mg tablet	1141146138	C10AA05	Atorvastatin
HMG CoA reductase inhibitors	rosuvastatin	1141192410	C10AA07	Rosuvastatin
HMG CoA reductase inhibitors	pravastatin	1140888648	C10AA03	Pravastatin
HMG CoA reductase inhibitors	simvador 10mg tablet	1141188146	C10AA01	Simvastatin
HMG CoA reductase inhibitors	crestor 10mg tablet	1141192414	C10AA07	Rosuvastatin
HMG CoA reductase inhibitors	fluvastatin	1140888594	C10AA04	Fluvastatin
HMG CoA reductase inhibitors	zocor 10mg tablet	1140881748	C10AA01	Simvastatin
HMG CoA reductase inhibitors	lipostat 10mg tablet	1140861970	C10AA03	Pravastatin
HMG CoA reductase inhibitors	lescol 20mg capsule	1140864592	C10AA04	Fluvastatin
Thyroid preparations	levothyroxine sodium	1141191044	H03AA01	Levothyroxine
Thyroid preparations	thyroxine product	1140884516	H03AA01	Levothyroxine
Thyroid preparations	thyroxine sodium	1140874852	H03AA01	Levothyroxine
Thyroid preparations	sodium thyroxine	1140910814	H03AA01	Levothyroxine
Thyroid preparations	eltroxin 25micrograms tablet	1141178036	H03AA01	Levothyroxine
Thyroid preparations	liothyronine	1140884512	H03AA02	Liothyronine
Thyroid preparations	sodium liothyronine	1140910520	H03AA02	Liothyronine

Immunosuppressants	tacrolimus	1140911642	D11AH01 |L04AD02	Tacrolimus
Immunosuppressants	methotrexateb	1140869848	L01BA01 |L04AX03	Methotrexate
Immunosuppressants	mtx - methotrexate	1140910036	L01BA01 |L04AX03	Methotrexate
Immunosuppressants	mycophenolate	1140925978	L04AA06	Mycophenolate Mofetil
Immunosuppressants	cellcept 250mg capsule	1140925986	L04AA06	Mycophenolate Mofetil
Immunosuppressants	myfortic 180mg gastro-resistant tablet	1141200450	L04AA06	Mycophenolic Acid
Immunosuppressants	sirolimus	1141173926	L04AA10 |S01XA23	Sirolimus
Immunosuppressants	leflunomide	1141166294	L04AA13	Leflunomide
Immunosuppressants	arava 10mg tablet	1141166302	L04AA13	Leflunomide
Immunosuppressants	humira 40mg injection solution 0.8ml prefilled syringe	1141188594	L04AB04	Adalimumab
Immunosuppressants	adalimumab	1141188588	L04AB04	Adalimumab
Immunosuppressants	neoral 10mg capsule	1141167744	L04AD01	Cyclosporine
Immunosuppressants	cyclosporin	2038459888	L04AD01 |S01XA18	Cyclosporine
Immunosuppressants	ciclosporin	1140909844	L04AD01 |S01XA18	Cyclosporine
Immunosuppressants	cya - cyclosporin	1140910382	L04AD01 |S01XA18	Cyclosporine
Immunosuppressants	prograf 0.5mg capsule	1141171242	L04AD02	Tacrolimus
Immunosuppressants	azathioprine	1140869930	L04AX01	Azathioprine
Immunosuppressants	imuran 10mg tablet	1141145996	L04AX01	Azathioprine
Immunosuppressants	azt - azathioprine	1140909864	L04AX01	Azathioprine
Antiinflammatory and antirheumatic products, non-steroids	benzydamine	1140884396	A01AD02 |G02CC03 |M01AX07 |M02AA05 |R02AX03	Benzydamine
Antiinflammatory and antirheumatic products,	indometacin	1140909936	C01EB03 |M01AB01 |M02AA23 |S01BC01	Indomethacin

non-steroids				
Antiinflammatory and antirheumatic products, non-steroids	indomethacin	1140871336	C01EB03 |M01AB01 |M02AA23 |S01BC01	Indomethacin
Antiinflammatory and antirheumatic products, non-steroids	indomethacin product	1141157452	C01EB03 |M01AB01 |M02AA23 |S01BC01	Indomethacin
Antiinflammatory and antirheumatic products, non-steroids	indometacin product	1141181656	C01EB03 |M01AB01 |M02AA23 |S01BC01	Indomethacin
Antiinflammatory and antirheumatic products, non-steroids	ibuprofen	1140871310	C01EB16 |G02CC01 |M01AE01 |M02AA13 |R02AX02	Ibuprofen
Antiinflammatory and antirheumatic products, non-steroids	ibuprofen product	1141157412	C01EB16 |G02CC01 |M01AE01 |M02AA13 |R02AX02	Ibuprofen
Antiinflammatory and antirheumatic products, non-steroids	brufen 200mg tablet	1140871374	C01EB16 |M01AE01 |R02AX02	Ibuprofen
Antiinflammatory and antirheumatic products, non-steroids	diclofenac	1140884488	D11AX18 |M01AB05 |M02AA15 |S01BC03	Diclofenac
Antiinflammatory and antirheumatic products, non-steroids	naproxen	1140871462	G02CC02 |M01AE02 |M02AA12	Naproxen
Antiinflammatory and	celecoxib	1141176662	L01XX33 |M01AH01	Celecoxib

antirheumatic products, non-steroids				
Antiinflammatory and antirheumatic products, non-steroids	indocid 25mg capsule	1140871354	M01AB01	Indomethacin
Antiinflammatory and antirheumatic products, non-steroids	sulindac	1140871604	M01AB02	Sulindac
Antiinflammatory and antirheumatic products, non-steroids	dicloflex 25mg e/c tablet	1140921828	M01AB05	Diclofenac
Antiinflammatory and antirheumatic products, non-steroids	voltarol 25mg e/c tablet	1140871168	M01AB05	Diclofenac
Antiinflammatory and antirheumatic products, non-steroids	diclomax sr 75mg m/r capsule	1140917394	M01AB05	Diclofenac
Antiinflammatory and antirheumatic products, non-steroids	rhumalgan 25mg e/c tablet	1140871180	M01AB05	Diclofenac
Antiinflammatory and antirheumatic products, non-steroids	volsaid retard 75mg m/r tablet	1140923920	M01AB05	Diclofenac
Antiinflammatory and antirheumatic products, non-steroids	motifene 75mg e/c+m/r capsule	1140909354	M01AB05	Diclofenac

Antiinflammatory and antirheumatic products, non-steroids	voltaren retard 100mg m/r tablet	1140877872	M01AB05	Diclofenac
Antiinflammatory and antirheumatic products, non-steroids	fenactol 25mg e/c tablet	1141182674	M01AB05	Diclofenac
Antiinflammatory and antirheumatic products, non-steroids	voltarol 100mg suppository	1140871174	M01AB05	Diclofenac
Antiinflammatory and antirheumatic products, non-steroids	rheumatac retard 75mg m/r tablet	1141167426	M01AB05	Diclofenac
Antiinflammatory and antirheumatic products, non-steroids	voltarene lp 100mg m/r tablet	1140877874	M01AB05	Diclofenac
Antiinflammatory and antirheumatic products, non-steroids	etodolac	1140871188	M01AB08	Etodolac
Antiinflammatory and antirheumatic products, non-steroids	lodine 200mg tablet	1140871196	M01AB08	Etodolac
Antiinflammatory and antirheumatic products, non-steroids	eccoxolac 300mg capsule	1141193170	M01AB08	Etodolac
Antiinflammatory and antirheumatic products,	acemetacin	1140875278	M01AB11	Acemetacin

non-steroids				
Antiinflammatory and antirheumatic products, non-steroids	ketorolac	1140884558	M01AB15 |S01BC05	Ketorolac
Antiinflammatory and antirheumatic products, non-steroids	aceclofenac	1140925806	M01AB16 |M02AA25	Aceclofenac
Antiinflammatory and antirheumatic products, non-steroids	arthrotec tablet	1140871266	M01AB55	Diclofenac |Misoprostol
Antiinflammatory and antirheumatic products, non-steroids	arthrotec 50 tablet	1140927086	M01AB55	Diclofenac |Misoprostol
Antiinflammatory and antirheumatic products, non-steroids	diclofenac sodium+misoprostol	1140878036	M01AB55	Diclofenac |Misoprostol
Antiinflammatory and antirheumatic products, non-steroids	feldene 10mg capsule	1140871672	M01AC01	Piroxicam
Antiinflammatory and antirheumatic products, non-steroids	brexidol 20mg tablet	1141169530	M01AC01	Piroxicam
Antiinflammatory and antirheumatic products, non-steroids	piroxicam	1140871666	M01AC01 |M02AA07 |S01BC06	Piroxicam
Antiinflammatory and	tenoxicam	1140875346	M01AC02	Tenoxicam

antirheumatic products, non-steroids				
Antiinflammatory and antirheumatic products, non-steroids	meloxicam	1140926732	M01AC06	Meloxicam
Antiinflammatory and antirheumatic products, non-steroids	mobic 15mg tablet	1140926796	M01AC06	Meloxicam
Antiinflammatory and antirheumatic products, non-steroids	mobic 7.5mg tablet	1140926794	M01AC06	Meloxicam
Antiinflammatory and antirheumatic products, non-steroids	nurofen 200mg tablet	1141187776	M01AE01	Ibuprofen
Antiinflammatory and antirheumatic products, non-steroids	cuprofen 200mg tablet	1140871388	M01AE01	Ibuprofen
Antiinflammatory and antirheumatic products, non-steroids	anadin ibuprofen 200mg tablet	1141153134	M01AE01	Ibuprofen
Antiinflammatory and antirheumatic products, non-steroids	naprosyn 250mg tablet	1140871472	M01AE02	Naproxen
Antiinflammatory and antirheumatic products, non-steroids	oruvail 100 m/r capsule	1140871522	M01AE03	Ketoprofen

Antiinflammatory and antirheumatic products, non-steroids	ketoprofen	1140871506	M01AE03 |M02AA10	Ketoprofen
Antiinflammatory and antirheumatic products, non-steroids	froben 50mg tablet	1140871238	M01AE09	Flurbiprofen
Antiinflammatory and antirheumatic products, non-steroids	flurbiprofen	1140871236	M01AE09 |M02AA19 |R02AX01 |S01BC04	Flurbiprofen
Antiinflammatory and antirheumatic products, non-steroids	surgam 200mg tablet	1140871616	M01AE11	Tiaprofenic Acid
Antiinflammatory and antirheumatic products, non-steroids	ibuprofen+menthol 5%/3% gel	1140911748	M01AE51	Ibuprofen |Menthol
Antiinflammatory and antirheumatic products, non-steroids	napratec tablet combination pack	1140871638	M01AE56	Naproxen |Misoprostol
Antiinflammatory and antirheumatic products, non-steroids	naproxen+misoprostol	1140881612	M01AE56	Naproxen |Misoprostol
Antiinflammatory and antirheumatic products, non-steroids	mefenamic acid	1140871542	M01AG01	Mefenamic Acid
Antiinflammatory and antirheumatic products,	ponstan 250mg capsule	1140871546	M01AG01	Mefenamic Acid

non-steroids				
Antiinflammatory and antirheumatic products, non-steroids	tolfenamic acid	1140928840	M01AG02	Tolfenamic Acid
Antiinflammatory and antirheumatic products, non-steroids	celebrex 100mg capsule	1141176668	M01AH01	Celecoxib
Antiinflammatory and antirheumatic products, non-steroids	celebrex 200mg capsule	1141176670	M01AH01	Celecoxib
Antiinflammatory and antirheumatic products, non-steroids	etoricoxib	1141180140	M01AH05	Etoricoxib
Antiinflammatory and antirheumatic products, non-steroids	arcoxia 60mg tablet	1141180148	M01AH05	Etoricoxib
Antiinflammatory and antirheumatic products, non-steroids	arcoxia 90mg tablet	1141180150	M01AH05	Etoricoxib
Antiinflammatory and antirheumatic products, non-steroids	arcoxia 120mg tablet	1141180152	M01AH05	Etoricoxib
Antiinflammatory and antirheumatic products, non-steroids	nabumetone	1140875336	M01AX01	Nabumetone
Antiinflammatory and	relifex 500mg tablet	1140875338	M01AX01	Nabumetone

antirheumatic products, non-steroids				
Antiinflammatory and antirheumatic products, non-steroids	glucosamine product	1141188442	M01AX05	Glucosamine
Antiinflammatory and antirheumatic products, non-steroids	chondroitin product	1187	M01AX25	Chondroitin Sulfate
Drugs affecting bone structure and mineralization	alendronate sodium	1140922174	M05BA04	Alendronic Acid
Drugs affecting bone structure and mineralization	risedronate sodium	1141175684	M05BA07	Risedronate
Drugs affecting bone structure and mineralization	fosamax 5mg tablet	1141176570	M05BA04	Alendronic Acid
Drugs affecting bone structure and mineralization	ibandronic acid	1141180314	M05BA06	Ibandronate
Drugs affecting bone structure and mineralization	actonel 5mg tablet	1141175690	M05BA07	Risedronate
Drugs affecting bone structure and mineralization	didronel 200mg tablet	1140868772	M05BA01	Etidronic Acid
Drugs affecting bone structure and mineralization	protelos 2g sachets	1141200768	M05BX03	Strontium Ranelate
Drugs affecting bone structure and mineralization	zoledronic acid	1141173814	M05BA08	Zoledronic Acid
Drugs affecting bone structure and mineralization	sodium clodronate	1140868794	M05BA02	Clodronic Acid

Drugs affecting bone structure and mineralization	disodium pamidronate	1140868784	M05BA03	Pamidronate
Drugs affecting bone structure and mineralization	bonefos 400mg capsule	1140868804	M05BA02	Clodronic Acid
Drugs affecting bone structure and mineralization	bondronat 50mg tablet	1141190534	M05BA06	Ibandronate
Drugs affecting bone structure and mineralization	clodronate disodium	1140910728	M05BA02	Clodronic Acid
Drugs affecting bone structure and mineralization	etidronate disodium	1140913322	M05BA01	Etidronic Acid
Drugs affecting bone structure and mineralization	disodium etidronate+calcium carbonate	1140882980	M05BB01	Etidronic Acid |Calcium Carbonate
Opioids	fentanyl	1140880956	N01AH01 |N02AB03	Fentanyl
Opioids	fentanyl product	1141157470	N01AH01 |N02AB03	Fentanyl
Opioids	morphine	1140871692	N02AA01	Morphine
Opioids	oramorph 10mg/5ml oral solution	1140882272	N02AA01	Morphine
Opioids	mst continus 30mg m/r tablet	1140871780	N02AA01	Morphine
Opioids	mst continus 10mg m/r tablet	1140871776	N02AA01	Morphine
Opioids	zomorph 10mg m/r capsule	1141152986	N02AA01	Morphine
Opioids	oramorph 100mg/5ml concentrated oral solution	1140882274	N02AA01	Morphine
Opioids	sevredol 10mg tablet	1140871700	N02AA01	Morphine
Opioids	zomorph 30mg m/r capsule	1141152988	N02AA01	Morphine
Opioids	oramorph 10mg/5ml oral unit dose vial	1140871778	N02AA01	Morphine
Opioids	mst continus 100mg m/r tablet	1140871786	N02AA01	Morphine

Opioids	oramorph sr 10mg m/r tablet	1140871712	N02AA01	Morphine
Opioids	mst continus 60mg m/r tablet	1140871782	N02AA01	Morphine
Opioids	sevredol 20mg tablet	1140871704	N02AA01	Morphine
Opioids	zomorph 100mg m/r capsule	1141152992	N02AA01	Morphine
Opioids	oxycodone hydrochloride	1141171038	N02AA05	Oxycodone
Opioids	oxycontin 10mg m/r tablet	1141171048	N02AA05	Oxycodone
Opioids	oxycontin 20mg m/r tablet	1141171050	N02AA05	Oxycodone
Opioids	oxynorm 5mg capsule	1141171066	N02AA05	Oxycodone
Opioids	oxynorm 10mg capsule	1141170964	N02AA05	Oxycodone
Opioids	oxycontin 5mg m/r tablet	1141180792	N02AA05	Oxycodone
Opioids	oxycontin 40mg m/r tablet	1141171052	N02AA05	Oxycodone
Opioids	oxynorm 20mg capsule	1141170966	N02AA05	Oxycodone
Opioids	oxycontin 80mg m/r tablet	1141171054	N02AA05	Oxycodone
Opioids	dihydrocodeine	1140884464	N02AA08	Dihydrocodeine
Opioids	dhc continus 60mg m/r tablet	1140871920	N02AA08	Dihydrocodeine
Opioids	df118 30mg tablet	1140856454	N02AA08	Dihydrocodeine
Opioids	dhc continus 120mg m/r tablet	1140871926	N02AA08	Dihydrocodeine
Opioids	codeine phosphate+kaolin 10mg/3g/10ml mixture	1140865654	N02AA59	Codeine |Kaolin
Opioids	migraleve duopack tablet	1140872026	N02AA79	Acetaminophen |Codeine |Buclizine
Opioids	feminax tablet	1141188522	N02AA79	Caffeine |Acetaminophen |Codeine |Scopolamine
Opioids	pethidine	1140884388	N02AB02	Pethidine
Opioids	durogesic 25micrograms/hour patch	1140911830	N02AB03	Fentanyl
Opioids	durogesic 50micrograms/hour	1140911832	N02AB03	Fentanyl

	patch			
Opioids	durogesic 75micrograms/hour patch	1140911834	N02AB03	Fentanyl
Opioids	durogesic 100micrograms/hour patch	1140911836	N02AB03	Fentanyl
Opioids	co-proxamol	1140923348	N02AC54	Acetaminophen |Dextropropoxyphene
Opioids	temgesic 200mcg sublingual tablet	1140871734	N02AE01	Buprenorphine
Opioids	temgesic 400mcg sublingual tablet	1140871738	N02AE01	Buprenorphine
Opioids	transtec 35micrograms/hour transdermal patch	1141180012	N02AE01	Buprenorphine
Opioids	transtec 70micrograms/hour transdermal patch	1141180020	N02AE01	Buprenorphine
Opioids	buprenorphine	1140871732	N02AE01 |N07BC01	Buprenorphine
Opioids	distalgesic tablet	1140868260	N02AJ	Acetaminophen |Dextropropoxyphene
Opioids	co-dydramol	1140923350	N02AJ01	Acetaminophen |Dihydrocodeine
Opioids	remedeine tablet	1140871684	N02AJ01	Acetaminophen |Dihydrocodeine
Opioids	paracetamol+dihydrocodeine tartrate	1140882396	N02AJ01	Acetaminophen |Dihydrocodeine
Opioids	paramol tablet	1140863552	N02AJ01	Acetaminophen |Dihydrocodeine
Opioids	remedeine forte tablet	1140871686	N02AJ01	Acetaminophen |Dihydrocodeine
Opioids	co-codamol	1140923346	N02AJ06	AD22:D1744D22:G1290cetaminophen |Codeine
Opioids	paracetamol + codeine	1140882394	N02AJ06	Acetaminophen |Codeine
Opioids	solpadol caplet	1140871688	N02AJ06	Acetaminophen |Codeine
Opioids	syndol tablet	1140856342	N02AJ06	Acetaminophen |Codeine |Ketorolac
Opioids	solpadol capsule	1141168122	N02AJ06	Acetaminophen |Codeine

Opioids	tylex capsule	1140871680	N02AJ06	Acetaminophen |Codeine
Opioids	zapain caplet	1141178052	N02AJ06	Acetaminophen |Codeine
Opioids	solpadeine capsule	1141168650	N02AJ06	Caffeine |Acetaminophen |Codeine
Opioids	kapake tablet	1140864070	N02AJ06	Acetaminophen |Codeine
Opioids	codipar caplet	1141187304	N02AJ06	Acetaminophen |Codeine
Opioids	solpadeine tablet	1141168648	N02AJ06	Acetaminophen |Codeine
Opioids	kapake capsule	1141165512	N02AJ06	Acetaminophen |Codeine
Opioids	solpadol effervescent tablet	1140871682	N02AJ06	Acetaminophen |Codeine
Opioids	zapain capsule	1141178054	N02AJ06	Acetaminophen |Codeine
Opioids	paracodol capsule	1140925778	N02AJ06	Acetaminophen |Codeine
Opioids	migraleve yellow tablet	1140872030	N02AJ06	Acetaminophen |Codeine
Opioids	kapake 30/500 effervescent tablet	1141190656	N02AJ06	Acetaminophen |Codeine
Opioids	solpadeine forte dispersible tablet	1140856340	N02AJ06	Caffeine |Acetaminophen |Codeine
Opioids	codis dispersible tablet	1140856336	N02AJ07	Codeine |Acetylsalicylic Acid
Opioids	aspirin+codeine	1140882392	N02AJ07	Codeine |Acetylsalicylic Acid
Opioids	co-codaprin	1140923344	N02AJ07	Codeine |Acetylsalicylic Acid
Opioids	ibuprofen+codeine phosphate	1140878030	N02AJ08	Codeine |Ibuprofen
Opioids	cuprofen plus tablet	1141190952	N02AJ08	Codeine |Ibuprofen
Opioids	migraleve tablet	1141168554	N02AJ09	Acetaminophen |Codeine |Buclizine
Opioids	solpadeine soluble effervescent tablet	1140856442	N02AJ09	Caffeine |Acetaminophen |Codeine
Opioids	solpadeine plus soluble effervescent tablet	1141189064	N02AJ09	Caffeine |Acetaminophen |Codeine
Opioids	solpadeine plus capsule	1141189008	N02AJ09	Caffeine |Acetaminophen |Codeine
Opioids	solpadeine max tablet	1141167748	N02AJ09	Acetaminophen |Codeine
Opioids	solpadeine plus tablet	1141189010	N02AJ09	Caffeine |Acetaminophen |Codeine

Opioids	veganin tablet	1140856348	N02AJ09	Caffeine |Acetaminophen |Codeine
Opioids	propain tablet	1140856436	N02AJ09	Caffeine |Acetaminophen |Codeine |Diphenhydramine
Opioids	propain caplet	1141172966	N02AJ09	Caffeine |Acetaminophen |Codeine |Diphenhydramine
Opioids	tramacet 325mg/37.5mg tablet	1141190960	N02AJ13	Tramadol |Acetaminophen
Opioids	tramadol	1140864992	N02AX02	Tramadol
Opioids	zydol 50mg capsule	1140865000	N02AX02	Tramadol
Opioids	zamadol 50mg capsule	1140928742	N02AX02	Tramadol
Opioids	zamadol sr 100mg m/r capsule	1141153424	N02AX02	Tramadol
Opioids	zydol sr 100mg m/r tablet	1140922636	N02AX02	Tramadol
Opioids	zydol soluble 50mg tablet	1140928372	N02AX02	Morphine
Opioids	meptazinol	1140881026	N02AX05	Meptazinol Hydrochloride
Opioids	meptid 200mg tablet	1140881028	N02AX05	Meptazinol Hydrochloride
Opioids	paracetamol+tramadol	1141190956	N02AX52	Tramadol |Acetaminophen
Antimigraine preparations	sumatriptan	1140884412	N02CC01	Sumatriptan
Antimigraine preparations	imigran 50mg tablet	1140911658	N02CC01	Sumatriptan
Antimigraine preparations	pizotifen	1140883664	N02CX01	Pizotifen
Antimigraine preparations	rizatriptan	1141167932	N02CC04	Rizatriptan
Antimigraine preparations	clonidine	1140883468	C02AC01 |N02CX02 |S01EA04	Clonidine
Antimigraine preparations	maxalt 5mg tablet	1141167940	N02CC04	Rizatriptan
Antimigraine preparations	dixarit 25mcg tablet	1140871984	N02CX02	Clonidine
Antimigraine preparations	migril tablet	1140872058	N02CA52	Caffeine |Ergotamine |Cyclizine
Antimigraine preparations	almotriptan	1141172728	N02CC05	Almotriptan
Antimigraine preparations	clonidine hydrochloride	1140871986	N02CX02	Clonidine

	25micrograms tablet			
Antimigraine preparations	sanomigran 500micrograms tablet	1140871996	N02CX01	Pizotifen
Antimigraine preparations	sumatriptan product	1141157332	N02CC01	Sumatriptan
Antimigraine preparations	almogran 12.5mg tablet	1141172628	N02CC05	Almotriptan
Antimigraine preparations	naratriptan	1141151284	N02CC02	Naratriptan
Antimigraine preparations	naramig 2.5mg tablet	1141151288	N02CC02	Naratriptan
Antimigraine preparations	eletriptan	1141185436	N02CC06	Eletriptan
Antimigraine preparations	cafergot tablet	1140872050	N02CA52	Caffeine |Ergotamine
Antimigraine preparations	zomig 2.5mg tablet	1141150624	N02CC03	Zolmitriptan
Antimigraine preparations	zolmitriptan	1141150620	N02CC03	Zolmitriptan
Antimigraine preparations	frovatriptan	1141192666	N02CC07	Frovatriptan
Antimigraine preparations	migard 2.5mg tablet	1141192670	N02CC07	Frovatriptan
Antimigraine preparations	relpax 20mg tablet	1141185448	N02CC06	Eletriptan
Antidepressants	imipramine	1140879630	N06AA02	Imipramine
Antidepressants	clomipramine	1140879620	N06AA04	Clomipramine
Antidepressants	anafranil 10mg capsule	1140867690	N06AA04	Clomipramine
Antidepressants	trimipramine	1140867756	N06AA06	Trimipramine
Antidepressants	surmontil 10mg tablet	1140867758	N06AA06	Trimipramine
Antidepressants	lofepramine	1140867726	N06AA07	Lofepramine
Antidepressants	amitriptyline	1140879616	N06AA09	Amitriptyline
Antidepressants	nortriptyline	1140867818	N06AA10	Nortriptyline
Antidepressants	allegron 10mg tablet	1140867820	N06AA10	Nortriptyline
Antidepressants	doxepin	1140867640	N06AA12	Doxepin
Antidepressants	dosulepin	1140909806	N06AA16	Dosulepin
Antidepressants	dothiepin	1140879628	N06AA16	Dosulepin
Antidepressants	prothiaden 25mg capsule	1140867624	N06AA16	Dosulepin

Antidepressants	fluoxetine	1140879540	N06AB03	Fluoxetine
Antidepressants	prozac 20mg capsule	1140867876	N06AB03	Fluoxetine
Antidepressants	oxactin 20mg capsule	1141174756	N06AB03	Fluoxetine
Antidepressants	citalopram	1140921600	N06AB04	Citalopram
Antidepressants	cipramil 10mg tablet	1141151946	N06AB04	Citalopram
Antidepressants	paroxetine	1140867888	N06AB05	Paroxetine
Antidepressants	seroxat 20mg tablet	1140882236	N06AB05	Paroxetine
Antidepressants	sertraline	1140867878	N06AB06	Sertraline
Antidepressants	lustral 50mg tablet	1140867884	N06AB06	Sertraline
Antidepressants	fluvoxamine	1140879544	N06AB08	Fluvoxamine
Antidepressants	escitalopram	1141180212	N06AB10	Escitalopram
Antidepressants	cipralex 5mg tablet	1141190158	N06AB10	Escitalopram
Antidepressants	phenelzine	1140867850	N06AF03	Phenelzine
Antidepressants	tranylcypromine	1140867914	N06AF04	Tranylcypromine
Antidepressants	moclobemide	1140867920	N06AG02	Moclobemide
Antidepressants	tryptophan product	1140867960	N06AX02	Tryptophan
Antidepressants	trazodone	1140879634	N06AX05	Trazodone
Antidepressants	mirtazapine	1141152732	N06AX11	Mirtazapine
Antidepressants	zispin 30mg tablet	1141152736	N06AX11	Mirtazapine
Antidepressants	zyban 150mg m/r tablet	1141176858	N06AX12	Bupropion
Antidepressants	bupropion	1141176854	N06AX12	Bupropion
Antidepressants	venlafaxine	1140916282	N06AX16	Venlafaxine
Antidepressants	efexor 37.5mg tablet	1140916288	N06AX16	Venlafaxine
Antidepressants	reboxetine	1141151978	N06AX18	Reboxetine
Antidepressants	duloxetine	1141200564	N06AX21	Duloxetine
Antidepressants	cymbalta 30mg gastro-resistant	1141201834	N06AX21	Duloxetine

	capsule			
Antidepressants	yentreve 20mg gastro-resistant capsule	1141200570	N06AX21	Duloxetine
Antidepressants	st john's wort/hypericum [ctsu]	1201	N06AX25	St. John'S Wort
Glucocorticoids	budesonide	1140862572	A07EA06 |D07AC09 |R01AD05 |R03BA02	Budesonide
Glucocorticoids	budesonide product	1141157418	A07EA06 |D07AC09 |R01AD05 |R03BA02	Budesonide
Glucocorticoids	beclometasone	1140909786	A07EA07 |D07AC15 |R01AD01 |R03BA01	Beclomethasone Dipropionate
Glucocorticoids	becotide 50 inhaler	1140862382	R03BA01	Beclomethasone Dipropionate
Glucocorticoids	fluticasone	1140888098	D07AC17 |R01AD08 |R03BA05	Fluticasone Propionate
Glucocorticoids	beclomethasone	1140884654	A07EA07 |D07AC15 |R01AD01 |R03BA01	Beclomethasone Dipropionate
Glucocorticoids	qvar 50 inhaler	1141167594	R03BA01	Beclomethasone Dipropionate
Glucocorticoids	beclazone 50 inhaler	1140862476	R03BA01	Beclomethasone Dipropionate
Glucocorticoids	flixotide 25micrograms inhaler	1140864286	R03BA05	Fluticasone Propionate
Glucocorticoids	betamethasone	1140874790	A07EA04 |C05AA05 |D07AC01 |D07XC01 |H02AB01 |R01AD06 |R03BA04 |S01BA06 |S01CB04 |S02BA07 |S03BA03	Betamethasone
Glucocorticoids	pulmicort ls 50micrograms inhaler	1140862574	R03BA02	Budesonide
Glucocorticoids	respiratory mometasone	1141191748	R01AD09 |R03BA07	Mometasone

Glucocorticoids	becloforte 250micrograms inhaler	1140862380	R03BA01	Beclomethasone Dipropionate
Glucocorticoids	triamcinolone	1140868426	A01AC01 |C05AA12 |D07AB09 |D07XB02 |H02AB08 |R01AD11 |R03BA06 |S01BA05	Triamcinolone
Glucocorticoids	novolizer budesonide 200micrograms/dose cartridge+inhaler	1141195280	R03BA02	Budesonide
Glucocorticoids	asmabec 50micrograms spacehaler	1141153270	R03BA01	Beclomethasone Dipropionate
Glucocorticoids	pulvinal beclomethasone diprop 100mcg breath-act dry pdr inh	1141179072	R03BA01	Beclomethasone Dipropionate
Glucocorticoids	becodisks 100micrograms disks+diskhaler	1140881922	R03BA01	Beclomethasone Dipropionate
Glucocorticoids	flunisolide	1140876154	R01AD04 |R03BA03	Flunisolide
Glucocorticoids	mometasone	1140888172	D07AC13 |D07XC03 |R01AD09 |R03BA07	Mometasone
Glucocorticoids	aerobec 50mcg autohaler	1140862474	R03BA01	Beclomethasone Dipropionate
Glucocorticoids	asmanex twisthaler 200mcg breath-actuated dry powder inhaler	1141191818	R03BA07	Mometasone
Antihistamines for systemic use	promethazine product	1140882082	D04AA10 |R06AD02	Promethazine
Antihistamines for systemic use	clemastine	1140862638	D04AA14 |R06AA04	Clemastine
Antihistamines for systemic use	diphenhydramine product	1140883600	D04AA32 |R06AA02	Diphenhydramine
Antihistamines for systemic use	azelastine	1140883500	R01AC03 |R06AX19 |S01GX07	Azelastine
Antihistamines for systemic use	nytol 25mg tablet	1140862722	R06AA02	Diphenhydramine
Antihistamines for systemic use	benadryl 25mg capsule	1140855562	R06AA02	Diphenhydramine

Antihistamines for systemic use	nytol 25mg caplet	1141153080	R06AA02	Diphenhydramine
Antihistamines for systemic use	piriton 4mg tablet	1140862628	R06AB04	Chlorphenamine
Antihistamines for systemic use	chlorphenamine	1140909790	R06AB04	Chlorphenamine
Antihistamines for systemic use	allergy relief antihistamine 4mg tablet	1141194224	R06AB04	Chlorphenamine
Antihistamines for systemic use	chlorpheniramine	1140883520	R06AB04	Chlorphenamine
Antihistamines for systemic use	chlorpheniramine maleate	1140862624	R06AB04	Chlorphenamine
Antihistamines for systemic use	piriton 10mg/1ml injection	1140862632	R06AB04	Chlorphenamine
Antihistamines for systemic use	vallergan 10mg tablet	1140862742	R06AD01	Alimemazine
Antihistamines for systemic use	alimemazine	1140909792	R06AD01	Alimemazine
Antihistamines for systemic use	phenergan 10mg tablet	1140862810	R06AD02	Promethazine
Antihistamines for systemic use	cyclizine	1140868080	R06AE03	Cyclizine
Antihistamines for systemic use	cetirizine	1140883504	R06AE07	Cetirizine
Antihistamines for systemic use	zirtek allergy 10mg tablet	1141180662	R06AE07	Cetirizine
Antihistamines for systemic use	zirtek 10mg tablet	1140862770	R06AE07	Cetirizine
Antihistamines for systemic use	care cetirizine hayfever relief 10mg tablet	1141187230	R06AE07	Cetirizine
Antihistamines for systemic use	piriteze 10mg tablet	1141179830	R06AE07	Cetirizine
Antihistamines for systemic use	benadryl one a day 10mg tablet	1141181562	R06AE07	Cetirizine
Antihistamines for systemic use	galpharm hayfever and allergy relief 10mg tablet	1141179944	R06AE07	Cetirizine
Antihistamines for systemic use	numark hayfever and allergy relief 10mg tablet	1141194228	R06AE07	Cetirizine
Antihistamines for systemic use	levocetirizine	1141184748	R06AE09	Levocetirizine
Antihistamines for systemic use	xyzal 5mg tablet	1141184752	R06AE09	Levocetirizine
Antihistamines for systemic use	terfenadine	1140862830	R06AX12	Terfenadine

Antihistamines for systemic use	loratadine	1140862772	R06AX13	Loratadine
Antihistamines for systemic use	clarityn 10mg tablet	1140862776	R06AX13	Loratadine
Antihistamines for systemic use	loratadine product	1141157324	R06AX13	Loratadine
Antihistamines for systemic use	ketotifen	1140862668	R06AX17 |S01GX08	Ketotifen
Antihistamines for systemic use	acrivastine	1140862760	R06AX18	Acrivastine
Antihistamines for systemic use	benadryl allergy relief 8mg capsule	1141162526	R06AX18	Acrivastine
Antihistamines for systemic use	mizolastine product	1141157056	R06AX25	Mizolastine
Antihistamines for systemic use	mizollen 10mg m/r tablet	1141157060	R06AX25	Mizolastine
Antihistamines for systemic use	fexofenadine	1141146428	R06AX26	Fexofenadine
Antihistamines for systemic use	telfast 30 tablet	1141194386	R06AX26	Fexofenadine
Antihistamines for systemic use	desloratadine	1141172924	R06AX27	Desloratadine
Antihistamines for systemic use	neoclarityn 5mg tablet	1141172928	R06AX27	Desloratadine

Table S5. Detailed information on summary data for 4 site-specific cancers in primary MR analysis

Cancer	GWAS ID in IEU	Consortium	Cases (n)	Controls (n)	Sample size (n)	PMID
Breast cancer	ieu-a-1126	BCAC	122977	105974	228951	29059683
Lung cancer	ieu-a-966	ILCCO	11348	15861	27209	24880342
Colorectal cancer	NA	A meta-analysis of GWAS	19948	12124	32072	30510241
Prostate cancer	ieu-b-85	PRACTICAL	79148	61106	140254	29892016

Table S6. Instrumental variables were used to perform MR analysis instead of 19 medication use

Exposure	SNP	Effect_ allele. exposure	Other_ allele. exposure	Eaf.exposure	Beta.exposure	Se.exposure	Pval.exposure	Samplesize. exposure	R2	F
Drugs for peptic ulcer and GORD	rs1619179	C	A	0.154861	-0.070056871	0.010626536	4.3E-11	132367	0.000328243	43.46217884
Drugs for peptic ulcer and GORD	rs6965956	A	G	0.4279	0.050471478	0.007786131	9E-11	132367	0.000317345	42.01864242
Drugs for peptic ulcer and GORD	rs3858461	C	T	0.448978	-0.044241407	0.007762368	0.000000012	132367	0.000245348	32.48350139
Drugs for peptic ulcer and GORD	rs11171710	A	G	0.447089	0.046545742	0.007827957	2.7E-09	132367	0.000267035	35.35546481
Drugs for peptic ulcer and GORD	rs2051815	A	G	0.320032	-0.04516697	0.008278086	0.000000049	132367	0.000224856	29.76976747
Drugs used in diabetes	rs2293476	C	G	0.228804	0.077206	0.013822208	0.000000023	305913	0.000101978	31.19927005
Drugs used in diabetes	rs2476601	A	G	0.102882	0.110628642	0.019065163	6.5E-09	305913	0.000110055	33.67063345
Drugs used in diabetes	rs79687284	C	G	0.0346315	0.199636214	0.031752396	3.2E-10	305913	0.000129203	39.52963616
Drugs used in diabetes	rs780093	T	C	0.383979	-0.081971326	0.01192995	6.4E-12	305913	0.000154306	47.21106654
Drugs used in diabetes	rs77059113	G	T	0.0734371	-0.175301046	0.022359209	4.5E-15	305913	0.000200896	61.46859883
Drugs used in diabetes	rs2723063	G	A	0.411067	-0.065654269	0.011799927	0.000000026	305913	0.000101187	30.95739637
Drugs used in diabetes	rs10184004	T	C	0.405503	-0.076864867	0.011829002	8.1E-11	305913	0.000138007	42.22374189
Drugs used in diabetes	rs2972155	C	G	0.346629	-0.086238024	0.012217194	1.7E-12	305913	0.000162849	49.82551736
Drugs used in diabetes	rs17036160	T	C	0.116465	-0.11809141	0.018121775	7.2E-11	305913	0.000138796	42.4651156
Drugs used in diabetes	rs1496653	G	A	0.20471	-0.115987473	0.014375717	7.1E-16	305913	0.000212752	65.09690183
Drugs used in diabetes	rs11720108	T	C	0.247134	-0.093975934	0.013475255	3.1E-12	305913	0.000158962	48.63584426
Drugs used in diabetes	rs9854769	G	A	0.31516	0.112895556	0.012502245	1.7E-19	305913	0.00026648	81.54077836
Drugs used in diabetes	rs10755148	A	G	0.359115	-0.111188202	0.012095099	3.8E-20	305913	0.000276173	84.50759746
Drugs used in diabetes	rs464605	C	T	0.253257	-0.086158117	0.013943375	6.4E-10	305913	0.000124797	38.18156391
Drugs used in diabetes	rs116782923	T	A	0.052571	0.207473217	0.02604948	1.7E-15	305913	0.000207318	63.43415173
Drugs used in diabetes	rs9379084	A	G	0.104171	-0.10827128	0.019512389	0.000000029	305913	0.000100638	30.7895116
Drugs used in diabetes	rs7766070	A	C	0.265183	0.11792	0.013141332	2.9E-19	305913	0.000263138	80.51805977
Drugs used in diabetes	rs2395040	G	A	0.466143	0.070285293	0.011671191	1.7E-09	305913	0.000118536	36.26567305

Drugs used in diabetes	rs3104415	C	A	0.340586	0.107304383	0.012247913	1.9E-18	305913	0.000250844	76.75521882
Drugs used in diabetes	rs9273364	G	T	0.302909	0.231936996	0.012629949	2.5E-75	305913	0.001101185	337.2360962
Drugs used in diabetes	rs4715207	T	C	0.179898	0.09093272	0.015124692	1.8E-09	305913	0.000118146	36.1463483
Drugs used in diabetes	rs11759026	G	A	0.226203	0.090316867	0.013935279	9.1E-11	305913	0.000137293	42.00524535
Drugs used in diabetes	rs3383	T	C	0.365922	-0.067971362	0.012099948	0.000000019	305913	0.000103144	31.55601214
Drugs used in diabetes	rs1974619	C	T	0.449969	-0.069310806	0.011680278	0.000000003	305913	0.000115093	35.21216439
Drugs used in diabetes	rs849140	T	C	0.411436	0.081292854	0.011819008	6.1E-12	305913	0.000154624	47.30857205
Drugs used in diabetes	rs515071	A	G	0.238099	-0.075833455	0.01364169	0.000000027	305913	0.000101005	30.90171033
Drugs used in diabetes	rs3802177	A	G	0.309928	-0.095131107	0.012563303	3.7E-14	305913	0.000187395	57.33694937
Drugs used in diabetes	rs10965246	C	T	0.17563	-0.143406968	0.015294879	6.8E-21	305913	0.000287293	87.9114718
Drugs used in diabetes	rs9410573	C	T	0.421202	-0.087119321	0.011811566	1.6E-13	305913	0.000177803	54.40153862
Drugs used in diabetes	rs11257655	T	C	0.208417	0.099439972	0.014271732	3.2E-12	305913	0.000158673	48.54737701
Drugs used in diabetes	rs697238	T	G	0.420872	0.108555698	0.01176904	2.9E-20	305913	0.000278038	85.0786002
Drugs used in diabetes	rs1112718	G	A	0.405782	-0.101585444	0.01183558	9.2E-18	305913	0.000240758	73.66836667
Drugs used in diabetes	rs7903146	T	C	0.291287	0.327302553	0.012760456	4.3E-145	305913	0.002146028	657.905527
Drugs used in diabetes	rs231361	A	G	0.250479	0.073669429	0.013429525	0.000000041	305913	9.83587E-05	30.09195996
Drugs used in diabetes	rs2237895	C	A	0.415789	0.083675939	0.011755483	1.1E-12	305913	0.000165596	50.66608931
Drugs used in diabetes	rs2074314	C	T	0.354389	0.069879643	0.01215135	8.9E-09	305913	0.000108095	33.07116369
Drugs used in diabetes	rs1056387	G	T	0.450353	-0.064100826	0.011663284	0.000000039	305913	9.8729E-05	30.20526741
Drugs used in diabetes	rs77464186	C	A	0.156347	-0.105954187	0.015973225	3.3E-11	305913	0.000143811	43.99954631
Drugs used in diabetes	rs76895963	G	T	0.0141213	-0.442334936	0.049928609	8E-19	305913	0.000256504	78.48753994
Drugs used in diabetes	rs2258238	T	A	0.104567	0.112517315	0.019022363	3.3E-09	305913	0.000114357	34.98701993
Drugs used in diabetes	rs1359790	A	G	0.288114	-0.102855312	0.012846225	1.2E-15	305913	0.000209514	64.10613466
Drugs used in diabetes	rs8017808	T	G	0.242765	-0.079489992	0.013695558	6.5E-09	305913	0.000110108	33.68701907
Drugs used in diabetes	rs2268967	A	G	0.228056	0.08002151	0.013956616	9.8E-09	305913	0.000107451	32.87383641
Drugs used in diabetes	rs11070332	A	G	0.347665	0.07004768	0.012274731	0.000000012	305913	0.000106444	32.56572018
Drugs used in diabetes	rs12910361	A	G	0.28639	-0.083818465	0.012831087	6.5E-11	305913	0.000139474	42.67265791
Drugs used in diabetes	rs28678152	T	C	0.262906	0.073226251	0.013281349	0.000000035	305913	9.93592E-05	30.39809602
Drugs used in diabetes	rs56094641	G	A	0.404982	0.117936656	0.01181671	1.9E-23	305913	0.000325511	99.60969789
Drugs used in diabetes	rs72802358	C	G	0.1004	-0.112404307	0.019383779	6.7E-09	305913	0.000109912	33.6268487
Drugs used in diabetes	rs7501939	T	C	0.397921	0.080408395	0.011856895	1.2E-11	305913	0.000150313	45.98942362
Drugs used in diabetes	rs9957264	A	C	0.16477	-0.092831513	0.015698315	3.3E-09	305913	0.000114298	34.96889726
Drugs used in diabetes	rs7409148	C	T	0.185829	-0.082887832	0.015064625	0.000000038	305913	9.89519E-05	30.27346114

Drugs used in diabetes	rs112972879	A	G	0.361546	-0.083148637	0.012265265	1.2E-11	305913	0.000150208	45.95720217
Drugs used in diabetes	rs1800961	T	C	0.0308713	0.237393444	0.033523211	1.4E-12	305913	0.000163899	50.14681723
Antithrombotic agents	rs12740374	T	G	0.221168	-0.074709755	0.008105044	3E-20	153639	0.000552716	84.96464583
Antithrombotic agents	rs6025	T	C	0.0233795	0.147872685	0.022256377	3.1E-11	153639	0.000287237	44.14294563
Antithrombotic agents	rs4299376	G	T	0.323265	0.041627513	0.007198171	7.3E-09	153639	0.000217631	33.44344022
Antithrombotic agents	rs117733303	G	A	0.0194872	0.188305588	0.024340005	1E-14	153639	0.000389417	59.85210589
Antithrombotic agents	rs74617384	T	A	0.0815418	0.114872005	0.012311457	1.1E-20	153639	0.00056632	87.05703411
Antithrombotic agents	rs17482753	T	G	0.10142	-0.070946572	0.011154625	2E-10	153639	0.000263231	40.45267346
Antithrombotic agents	rs28601761	G	C	0.415801	-0.051481012	0.006971504	1.5E-13	153639	0.000354802	54.53000944
Antithrombotic agents	rs1831733	C	T	0.480371	0.058895787	0.006789594	4.2E-18	153639	0.000489516	75.24458514
Antithrombotic agents	rs532436	A	G	0.185935	0.066340996	0.008661608	1.9E-14	153639	0.00038168	58.66259144
Antithrombotic agents	rs964184	G	C	0.132603	0.067194752	0.009910024	1.2E-11	153639	0.000299151	45.9743568
Antithrombotic agents	rs56214516	C	A	0.192373	0.049012261	0.008626306	0.000000013	153639	0.000210071	32.2815321
Antithrombotic agents	rs73015016	A	G	0.119006	-0.089790481	0.010400082	5.9E-18	153639	0.000484925	74.53863917
Antithrombotic agents	rs7412	T	C	0.0800838	-0.104794922	0.01239736	2.8E-17	153639	0.000464856	71.45233347
Vasodilators used in cardiac diseases	rs55730499	T	C	0.0811461	0.298491837	0.034986724	1.4E-17	242659	0.000299869	72.78716046
Vasodilators used in cardiac diseases	rs1333047	T	A	0.492809	0.165319679	0.019089253	4.7E-18	242659	0.000308987	75.00110694
Antihypertensives	rs34071855	G	C	0.339889	0.110219373	0.019059309	7.3E-09	152380	0.000219421	33.44228011
Antihypertensives	rs35021474	C	G	0.385032	0.103529253	0.018403903	0.000000019	152380	0.000207629	31.64467093
Antihypertensives	rs4980379	T	C	0.365232	0.107983973	0.018611412	6.6E-09	152380	0.00022087	33.66311026
Antihypertensives	rs758374	C	T	0.304289	0.11363902	0.01936612	4.4E-09	152380	0.000225914	34.43214206
Diuretics	rs880315	C	T	0.337723	0.089395954	0.008414763	2.3E-26	229086	0.000492424	112.8620953
Diuretics	rs72993045	C	T	0.03029	0.201280792	0.022972393	1.9E-18	229086	0.000335003	76.76944628
Diuretics	rs57748895	T	A	0.0174202	0.184074663	0.029953456	8E-10	229086	0.000164825	37.76507673
Diuretics	rs1275988	C	T	0.384127	0.099095875	0.008102494	2.1E-34	229086	0.000652517	149.57876
Diuretics	rs6738113	A	G	0.366048	-0.045671256	0.008183105	0.000000024	229086	0.000135954	31.14912748
Diuretics	rs7599224	G	T	0.446085	0.045817295	0.008064615	0.000000013	229086	0.000140874	32.27660578
Diuretics	rs268263	T	A	0.244695	-0.05207466	0.009254369	0.000000018	229086	0.000138198	31.66324573
Diuretics	rs591668	A	G	0.396391	-0.075242162	0.008042858	8.3E-21	229086	0.000381889	87.51812206
Diuretics	rs6442105	A	G	0.332355	-0.045588923	0.008339005	0.000000046	229086	0.000130447	29.88728246
Diuretics	rs2421647	C	A	0.463343	0.044606174	0.007884923	0.000000015	229086	0.000139681	32.00304366

Diuretics	rs28702684	G	C	0.498848	0.04827632	0.007863087	8.3E-10	229086	0.000164518	37.69452871
Diuretics	rs10857147	T	A	0.286176	0.104208016	0.008750747	1.1E-32	229086	0.00061865	141.8104521
Diuretics	rs13112725	G	C	0.240779	-0.056514365	0.009202481	8.2E-10	229086	0.000164603	37.71412718
Diuretics	rs7685862	C	A	0.203166	0.054590894	0.009790706	0.000000025	229086	0.000135692	31.08913338
Diuretics	rs300934	T	G	0.312059	0.05524486	0.00850054	8.1E-11	229086	0.000184337	42.23640668
Diuretics	rs6536076	A	T	0.307892	0.054945583	0.008558846	1.4E-10	229086	0.00017987	41.21272929
Diuretics	rs12656497	T	C	0.404841	-0.073033892	0.0080067	7.4E-20	229086	0.000363066	83.20280881
Diuretics	rs6894014	T	G	0.485217	0.056612507	0.007896427	7.5E-13	229086	0.00022432	51.39959432
Diuretics	rs10066170	G	A	0.392928	0.063519455	0.008152661	6.6E-15	229086	0.000264912	60.70311952
Diuretics	rs2984644	A	G	0.268206	0.054463326	0.008881524	8.7E-10	229086	0.000164121	37.6036077
Diuretics	rs7701003	G	A	0.370273	-0.073908247	0.008135208	1E-19	229086	0.000360158	82.5362431
Diuretics	rs3778546	T	C	0.284027	0.048194926	0.00874652	0.000000036	229086	0.000132518	30.36184849
Diuretics	rs198851	T	G	0.150702	0.066807363	0.010987504	1.2E-09	229086	0.000161355	36.96977058
Diuretics	rs9394951	C	T	0.433625	0.051841201	0.007916384	5.8E-11	229086	0.000187161	42.88373078
Diuretics	rs9375459	T	C	0.435739	0.087651471	0.007926246	2E-28	229086	0.000533523	122.2867986
Diuretics	rs6918911	T	A	0.0698601	-0.100521045	0.015772663	1.9E-10	229086	0.000177267	40.61628078
Diuretics	rs17080089	G	T	0.0716652	-0.106669389	0.015238674	2.6E-12	229086	0.000213842	48.99834991
Diuretics	rs916880	G	A	0.0730081	-0.169045029	0.015202282	1E-28	229086	0.000539454	123.6469639
Diuretics	rs6961048	G	C	0.10066	0.118298494	0.013091684	1.6E-19	229086	0.000356299	81.65139124
Diuretics	rs17477177	C	T	0.200037	0.066040307	0.009839307	1.9E-11	229086	0.00019661	45.04901827
Diuretics	rs3918226	T	C	0.0763907	0.166541395	0.014925387	6.5E-29	229086	0.000543199	124.5057438
Diuretics	rs17153559	G	A	0.375293	0.066706247	0.008276551	7.6E-16	229086	0.000283474	64.95767049
Diuretics	rs13278931	T	G	0.443525	0.047323154	0.008009674	3.5E-09	229086	0.000152354	34.90710987
Diuretics	rs7011889	C	A	0.444985	-0.047267039	0.00796209	2.9E-09	229086	0.000153814	35.24186141
Diuretics	rs1779209	T	C	0.279256	0.057617013	0.008792148	5.6E-11	229086	0.000187427	42.94451718
Diuretics	rs976785	T	C	0.330782	-0.057924587	0.008390972	5.1E-12	229086	0.000207976	47.65380726
Diuretics	rs72831344	T	C	0.145464	-0.090673989	0.011139335	4E-16	229086	0.00028915	66.25873787
Diuretics	rs10995307	C	T	0.500042	-0.0455296	0.007890792	7.9E-09	229086	0.000145306	33.29221104
Diuretics	rs7076938	C	T	0.263878	-0.053165881	0.008969648	3.1E-09	229086	0.000153338	35.13268969
Diuretics	rs11199835	G	A	0.319005	0.051952882	0.008433155	7.2E-10	229086	0.000165641	37.95205586
Diuretics	rs569550	G	T	0.385649	0.09494089	0.008128008	1.6E-31	229086	0.000595225	136.4377641
Diuretics	rs6483656	G	C	0.35589	-0.056152007	0.008215897	8.2E-12	229086	0.000203861	46.71074534
Diuretics	rs7110547	G	C	0.380033	0.050580227	0.008138025	5.1E-10	229086	0.000168598	38.6295523

Diuretics	rs12801188	A	G	0.363738	0.055872217	0.008195314	9.3E-12	229086	0.00020285	46.47900564
Diuretics	rs557675	G	T	0.47008	-0.043455708	0.00789721	0.000000037	229086	0.000132157	30.27906708
Diuretics	rs2455569	T	C	0.273535	-0.064772524	0.008903907	3.5E-13	229086	0.000230952	52.91960309
Diuretics	rs2024385	A	T	0.427823	-0.050581401	0.007969838	2.2E-10	229086	0.000175796	40.27902174
Diuretics	rs117913411	A	T	0.0324951	0.131361377	0.022249712	3.5E-09	229086	0.000152132	34.85642258
Diuretics	rs10878947	A	T	0.27337	0.05999934	0.008835427	1.1E-11	229086	0.000201257	46.11413193
Diuretics	rs11105352	A	G	0.166833	-0.087141979	0.010606129	2.1E-16	229086	0.000294588	67.50520664
Diuretics	rs7310615	C	G	0.48155	0.064379096	0.007930315	4.7E-16	229086	0.000287598	65.90297948
Diuretics	rs35436	T	C	0.382191	-0.0799003	0.008091302	5.4E-23	229086	0.000425477	97.51157393
Diuretics	rs9506725	C	T	0.370441	-0.066165998	0.00814593	4.6E-16	229086	0.000287915	65.97578968
Diuretics	rs57340570	A	C	0.0862868	0.080068565	0.013983961	0.00000001	229086	0.000143088	32.78384454
Diuretics	rs277168	C	T	0.494125	-0.04917564	0.007866296	4.1E-10	229086	0.000170564	39.08010045
Diuretics	rs12428857	T	C	0.0495341	0.108705011	0.018154207	2.1E-09	229086	0.000156487	35.85425983
Diuretics	rs72681698	C	T	0.0110045	-0.224507696	0.037732019	2.7E-09	229086	0.000154517	35.40287957
Diuretics	rs11632414	A	G	0.3264	0.066837416	0.008456791	2.7E-15	229086	0.000272591	62.46321689
Diuretics	rs7174222	T	C	0.467487	0.054932513	0.007902219	3.6E-12	229086	0.000210897	48.32334965
Diuretics	rs28483160	A	G	0.047166	0.10379244	0.018590054	0.000000024	229086	0.000136054	31.17212061
Diuretics	rs1894401	G	A	0.472142	0.070338357	0.007909967	6E-19	229086	0.000345054	79.07356381
Diuretics	rs77924615	A	G	0.194072	-0.068199584	0.010057113	1.2E-11	229086	0.000200692	45.98465986
Diuretics	rs2278557	G	C	0.40521	0.058077356	0.00800263	3.9E-13	229086	0.000229853	52.66770261
Diuretics	rs62039768	A	C	0.0954804	0.07656379	0.013471417	0.000000013	229086	0.000140981	32.30104134
Diuretics	rs56094641	G	A	0.40302	0.059995114	0.008004352	6.6E-14	229086	0.000245174	56.17920641
Diuretics	rs12928482	A	G	0.276392	-0.053540761	0.008843723	1.4E-09	229086	0.000159967	36.65177468
Diuretics	rs154656	A	T	0.425003	-0.04966361	0.00797469	4.7E-10	229086	0.000169269	38.78333184
Diuretics	rs5417	C	A	0.426897	-0.051768025	0.007955829	7.7E-11	229086	0.000184788	42.33977233
Diuretics	rs35317317	A	T	0.374293	0.055267322	0.008188818	1.5E-11	229086	0.000198797	45.55023293
Diuretics	rs3785837	G	A	0.228623	-0.055156658	0.009527429	7.1E-09	229086	0.000146279	33.51510984
Diuretics	rs1436138	G	A	0.359306	-0.051573776	0.00826129	4.3E-10	229086	0.000170094	38.97250938
Diuretics	rs72915163	T	C	0.253582	0.054368549	0.009172663	3.1E-09	229086	0.000153334	35.13183598
Diuretics	rs12978472	G	C	0.127718	-0.101797515	0.011856302	9E-18	229086	0.00032169	73.71774381
Diuretics	rs113701136	T	C	0.318913	0.053559701	0.008515879	3.2E-10	229086	0.000172641	39.55610258
Diuretics	rs6039216	C	T	0.36254	-0.076972714	0.008192966	5.7E-21	229086	0.000385146	88.26479222
Diuretics	rs6077947	T	G	0.433441	0.048510091	0.00792695	9.4E-10	229086	0.000163449	37.44968405

Diuretics	rs1407256	G	A	0.145224	0.064974991	0.011292261	8.7E-09	229086	0.000144501	33.10753753
Diuretics	rs6073354	C	T	0.499418	-0.047011589	0.007988621	0.000000004	229086	0.000151148	34.63079064
Diuretics	rs78302204	A	G	0.105716	0.106527733	0.012842886	1.1E-16	229086	0.000300242	68.80124774
Diuretics	rs2229742	C	G	0.10298	0.076235633	0.012948072	3.9E-09	229086	0.000151301	34.66586517
Beta blocking agents	rs2050265	G	A	0.164122	-0.083174712	0.011092184	6.5E-14	224024	0.000250926	56.22695804
Beta blocking agents	rs778124	A	G	0.374347	0.047101221	0.00852121	0.000000032	224024	0.000136367	30.55328651
Beta blocking agents	rs4970834	T	C	0.18364	-0.061627654	0.010692198	8.2E-09	224024	0.000148272	33.22105352
Beta blocking agents	rs10776752	T	G	0.0719762	0.10393464	0.015904859	6.4E-11	224024	0.000190583	42.70287516
Beta blocking agents	rs57748895	T	A	0.0175751	0.195610394	0.031256701	3.9E-10	224024	0.000174794	39.16459982
Beta blocking agents	rs2493136	T	C	0.40279	0.051549224	0.008391064	8.1E-10	224024	0.000168439	37.74038349
Beta blocking agents	rs1275988	C	T	0.382355	0.056638242	0.008493221	2.6E-11	224024	0.00019847	44.47037122
Beta blocking agents	rs7368883	A	G	0.359437	0.055433427	0.008596778	1.1E-10	224024	0.000185565	41.57843406
Beta blocking agents	rs55988870	C	T	0.367162	-0.053607436	0.008535039	3.4E-10	224024	0.000176063	39.44892195
Beta blocking agents	rs12494396	G	C	0.334855	-0.050804817	0.008717046	5.6E-09	224024	0.000151604	33.96778692
Beta blocking agents	rs7652333	T	C	0.483816	0.047401272	0.008436339	0.000000019	224024	0.000140902	31.56952421
Beta blocking agents	rs13125101	A	G	0.288018	0.097112074	0.009128556	2E-26	224024	0.000504927	113.1718406
Beta blocking agents	rs17042098	A	G	0.101276	0.079749929	0.013626774	4.8E-09	224024	0.000152867	34.25079341
Beta blocking agents	rs9286351	G	A	0.416748	0.051066837	0.008374271	1.1E-09	224024	0.000165965	37.18604648
Beta blocking agents	rs11731886	C	A	0.236995	-0.053222102	0.009705774	0.000000042	224024	0.000134206	30.06905562
Beta blocking agents	rs12656497	T	C	0.405186	-0.052527004	0.008381021	3.7E-10	224024	0.000175308	39.27964075
Beta blocking agents	rs17677603	G	A	0.390229	0.057320334	0.008528454	1.8E-11	224024	0.000201602	45.17237669
Beta blocking agents	rs7442660	A	G	0.36088	-0.058263787	0.008569942	1.1E-11	224024	0.00020628	46.2208301
Beta blocking agents	rs62436821	A	G	0.435368	0.060421275	0.008293105	3.2E-13	224024	0.000236891	53.0813034
Beta blocking agents	rs2105092	A	G	0.290392	-0.057990489	0.009096041	1.8E-10	224024	0.000181399	40.64479119
Beta blocking agents	rs117733303	G	A	0.0193349	0.169112037	0.029826333	0.000000014	224024	0.00014348	32.14736862
Beta blocking agents	rs74617384	T	A	0.0800491	0.110204024	0.015174364	3.8E-13	224024	0.000235384	52.74363532
Beta blocking agents	rs3735533	T	C	0.0746658	-0.104314952	0.015703754	3.1E-11	224024	0.000196927	44.12474945
Beta blocking agents	rs4515482	C	A	0.224624	-0.055357858	0.009883747	0.000000021	224024	0.00014001	31.36977647
Beta blocking agents	rs185963	T	C	0.434857	0.050627008	0.008369661	1.5E-09	224024	0.000163299	36.58852443
Beta blocking agents	rs3918226	T	C	0.0761934	0.146365761	0.01563296	7.8E-21	224024	0.00039114	87.6582218
Beta blocking agents	rs7820612	A	C	0.304079	-0.050677634	0.008944821	0.000000015	224024	0.000143263	32.09855309
Beta blocking agents	rs513159	T	C	0.233686	-0.053433496	0.009780355	0.000000047	224024	0.000133219	29.84791968
Beta blocking agents	rs2891168	G	A	0.488953	0.071484648	0.008223027	3.5E-18	224024	0.000337226	75.57153548

Beta blocking agents	rs7076100	A	T	0.405545	-0.055563161	0.008452227	4.9E-11	224024	0.000192865	43.21432457
Beta blocking agents	rs17210692	A	G	0.188106	-0.069548268	0.010543942	4.2E-11	224024	0.000194172	43.50735779
Beta blocking agents	rs1973765	C	T	0.384178	0.066691154	0.00851874	4.9E-15	224024	0.000273509	61.28890138
Beta blocking agents	rs11023910	T	A	0.205274	0.057553295	0.010154822	0.000000014	224024	0.000143364	32.12120672
Beta blocking agents	rs4923536	G	A	0.456313	0.046465392	0.008249064	0.000000018	224024	0.00014161	31.72823631
Beta blocking agents	rs604723	T	C	0.273879	-0.060166911	0.009330484	1.1E-10	224024	0.00018558	41.58179326
Beta blocking agents	rs7310615	C	G	0.482401	0.07970556	0.008299443	7.7E-22	224024	0.000411534	92.23068032
Beta blocking agents	rs35429	G	A	0.383864	-0.055391609	0.008464163	6E-11	224024	0.000191136	42.82684182
Beta blocking agents	rs1543927	T	C	0.26383	0.052594505	0.00940383	0.000000022	224024	0.00013961	31.28004383
Beta blocking agents	rs7183988	T	G	0.472301	0.078119033	0.008276394	3.8E-21	224024	0.000397525	89.08970118
Beta blocking agents	rs77924615	A	G	0.194395	-0.068638894	0.010518341	6.8E-11	224024	0.00019005	42.58357919
Beta blocking agents	rs11646715	G	A	0.477716	-0.05062956	0.008238775	8E-10	224024	0.000168545	37.76410414
Beta blocking agents	rs62043959	C	A	0.27972	-0.052480659	0.009259525	0.000000014	224024	0.000143372	32.12308403
Beta blocking agents	rs258317	T	C	0.427246	-0.056158983	0.008348011	1.7E-11	224024	0.000201972	45.25520332
Beta blocking agents	rs11649807	G	A	0.370876	-0.047116861	0.008623614	0.000000047	224024	0.000133236	29.85177744
Beta blocking agents	rs167479	T	G	0.473349	-0.056873344	0.008244125	5.2E-12	224024	0.000212394	47.59096088
Beta blocking agents	rs6039216	C	T	0.362735	-0.067307226	0.008573564	4.1E-15	224024	0.000275034	61.6306404
Beta blocking agents	rs62185663	A	C	0.252689	0.055121522	0.009511173	6.8E-09	224024	0.000149904	33.58693198
Beta blocking agents	rs78302204	A	G	0.105587	0.095600705	0.013445344	1.2E-12	224024	0.000225624	50.55620816
Calcium channel blockers	rs880315	C	T	0.339218	0.09883418	0.008580194	1.1E-30	204378	0.00064879	132.683191
Calcium channel blockers	rs59180873	G	A	0.0777984	-0.083082077	0.01507787	0.000000036	204378	0.000148537	30.36200661
Calcium channel blockers	rs61772626	G	A	0.123313	0.070881731	0.01219822	6.2E-09	204378	0.000165185	33.76536386
Calcium channel blockers	rs3790604	A	C	0.0725619	0.1520593	0.01547951	8.9E-23	204378	0.000471924	96.49555479
Calcium channel blockers	rs57748895	T	A	0.0175421	0.175209601	0.030480331	0.000000009	204378	0.000161649	33.04244642
Calcium channel blockers	rs11126666	A	G	0.255998	0.106201255	0.009194838	7.4E-31	204378	0.000652309	133.4034067
Calcium channel blockers	rs13387221	A	G	0.174669	-0.060807096	0.010603273	9.8E-09	204378	0.000160888	32.88700704

Calcium channel blockers	rs1439211	A	C	0.273839	-0.055266418	0.009025103	9.1E-10	204378	0.000183444	37.49851678
Calcium channel blockers	rs952227	A	G	0.326886	-0.057653563	0.008540113	1.5E-11	204378	0.000222943	45.57438452
Calcium channel blockers	rs2643826	T	C	0.452509	0.075560754	0.008097102	1E-20	204378	0.000425906	87.08214463
Calcium channel blockers	rs35593046	T	G	0.263655	-0.054404004	0.009303389	0.000000005	204378	0.000167291	34.19598432
Calcium channel blockers	rs9841978	A	G	0.322134	0.063494085	0.008657698	2.2E-13	204378	0.000263095	53.78453882
Calcium channel blockers	rs9855086	T	A	0.4614	-0.057911126	0.008047229	6.2E-13	204378	0.00025333	51.78775252
Calcium channel blockers	rs10857147	T	A	0.286785	0.113139437	0.008920802	7.4E-37	204378	0.000786402	160.8481098
Calcium channel blockers	rs56388530	C	T	0.240756	-0.052418575	0.009384992	0.000000023	204378	0.000152617	31.19595796
Calcium channel blockers	rs7685862	C	A	0.203951	0.068732413	0.009977091	5.6E-12	204378	0.000232156	47.45817767
Calcium channel blockers	rs10015412	A	C	0.313756	0.047643527	0.00863872	0.000000035	204378	0.000148802	30.41618445
Calcium channel blockers	rs13140296	G	A	0.46728	0.045077918	0.008067345	0.000000023	204378	0.000152745	31.22210494
Calcium channel blockers	rs72689147	T	G	0.184352	-0.066058708	0.010371672	1.9E-10	204378	0.000198446	40.56564365
Calcium channel blockers	rs11726072	A	G	0.122139	-0.06785178	0.012292728	0.000000034	204378	0.000149048	30.46643692
Calcium channel blockers	rs7733331	T	C	0.400308	-0.060675316	0.008208006	1.4E-13	204378	0.0002673	54.64424996
Calcium channel blockers	rs27041	C	T	0.148646	-0.067071121	0.011306505	0.000000003	204378	0.000172149	35.18926161
Calcium channel blockers	rs10061288	A	G	0.485343	0.045694383	0.008036829	0.000000013	204378	0.000158144	32.32599644
Calcium channel	rs1422278	T	G	0.131748	0.075955638	0.011840077	1.4E-10	204378	0.000201321	41.15349828

blockers										
Calcium channel blockers	rs6595839	T	C	0.313861	0.059347497	0.00871797	9.9E-12	204378	0.000226695	46.34146702
Calcium channel blockers	rs2962383	T	C	0.301942	0.048286941	0.008783025	0.000000038	204378	0.000147868	30.22503833
Calcium channel blockers	rs7701003	G	A	0.370667	-0.059909914	0.008302363	5.4E-13	204378	0.000254712	52.07026046
Calcium channel blockers	rs58367598	G	T	0.0364257	0.134095177	0.021406646	3.7E-10	204378	0.00019196	39.23962217
Calcium channel blockers	rs553108	A	G	0.418103	-0.051669798	0.008151226	2.3E-10	204378	0.000196566	40.18125032
Calcium channel blockers	rs9369409	C	G	0.403623	0.051312717	0.008163599	3.3E-10	204378	0.000193272	39.50776345
Calcium channel blockers	rs998584	A	C	0.481178	0.049987934	0.008051252	5.3E-10	204378	0.000188576	38.54777186
Calcium channel blockers	rs9398823	G	A	0.437024	0.08327413	0.008121924	1.1E-24	204378	0.000514097	105.1230796
Calcium channel blockers	rs2105092	A	G	0.289797	-0.053243794	0.00887556	0.000000002	204378	0.00017605	35.9867173
Calcium channel blockers	rs62434123	T	C	0.0713639	-0.090459779	0.015589352	6.5E-09	204378	0.000164721	33.67058593
Calcium channel blockers	rs13230181	A	G	0.235101	0.053742903	0.009460599	0.000000013	204378	0.000157871	32.27013148
Calcium channel blockers	rs3735533	T	C	0.0735793	-0.141444033	0.015402461	4.2E-20	204378	0.000412454	84.33051687
Calcium channel blockers	rs6961048	G	C	0.101332	0.111753319	0.013312125	4.7E-17	204378	0.000344701	70.47284944
Calcium channel blockers	rs143524414	A	G	0.0650858	-0.089636155	0.016399388	0.000000046	204378	0.000146155	29.87492854
Calcium channel blockers	rs12705389	T	C	0.241001	0.06615352	0.00938393	1.8E-12	204378	0.000243107	49.69722896
Calcium channel blockers	rs11556924	T	C	0.389692	-0.048157666	0.008222885	4.7E-09	204378	0.000167794	34.29874254

Calcium channel blockers	rs3918226	T	C	0.0761387	0.161264917	0.015257245	4.1E-26	204378	0.000546331	111.7180357
Calcium channel blockers	rs10254101	T	C	0.287323	0.049741682	0.008863186	0.00000002	204378	0.000154085	31.49610957
Calcium channel blockers	rs891556	G	C	0.397703	0.051189439	0.008223948	4.8E-10	204378	0.000189532	38.74323043
Calcium channel blockers	rs73207888	T	C	0.0339351	0.123120096	0.022212128	0.00000003	204378	0.000150307	30.72368556
Calcium channel blockers	rs2977324	T	G	0.298571	-0.04855126	0.008838592	0.000000039	204378	0.000147617	30.17384304
Calcium channel blockers	rs3110291	C	A	0.195127	-0.059019261	0.010337057	0.000000011	204378	0.000159474	32.59788991
Calcium channel blockers	rs7463212	T	A	0.459666	0.04745489	0.008064536	0.000000004	204378	0.000169393	34.62572623
Calcium channel blockers	rs76038906	T	G	0.034493	0.123353899	0.022045885	0.000000022	204378	0.000153162	31.30736004
Calcium channel blockers	rs6271	T	C	0.0739659	-0.096342052	0.01532435	3.2E-10	204378	0.000193352	39.52423044
Calcium channel blockers	rs12258967	G	C	0.298591	-0.05833311	0.008768982	2.9E-11	204378	0.000216473	44.25146545
Calcium channel blockers	rs72821787	A	C	0.156182	-0.095073594	0.011068223	8.7E-18	204378	0.000360889	73.78359223
Calcium channel blockers	rs57866767	C	T	0.433102	-0.051913773	0.008102719	1.5E-10	204378	0.000200809	41.04869665
Calcium channel blockers	rs12414028	A	T	0.0771978	-0.088384397	0.01503741	4.2E-09	204378	0.000169004	34.54624888
Calcium channel blockers	rs7082671	A	G	0.0159975	0.19940822	0.032044686	4.9E-10	204378	0.000189434	38.72307341
Calcium channel blockers	rs569550	G	T	0.386833	0.095690744	0.008293405	8.5E-31	204378	0.000650964	133.1281303
Calcium channel blockers	rs893311	T	G	0.20571	0.056468227	0.009893514	0.000000011	204378	0.000159369	32.5763857
Calcium channel	rs10835916	C	A	0.406175	0.046684858	0.008185157	0.000000012	204378	0.000159146	32.53072645

blockers										
Calcium channel blockers	rs10896795	G	A	0.241124	-0.058248167	0.009389243	5.5E-10	204378	0.000188273	38.48567263
Calcium channel blockers	rs633185	G	C	0.283039	-0.090648795	0.008984415	6.1E-24	204378	0.000497845	101.7982311
Calcium channel blockers	rs494356	T	C	0.132695	0.083987685	0.011858067	1.4E-12	204378	0.000245393	50.16480742
Calcium channel blockers	rs4547172	A	G	0.284436	0.049543405	0.008968398	0.000000033	204378	0.000149294	30.5167106
Calcium channel blockers	rs7308105	C	T	0.36704	-0.045739778	0.008371669	0.000000047	204378	0.000146038	29.85106262
Calcium channel blockers	rs317685	G	A	0.281102	0.057492406	0.008943272	1.3E-10	204378	0.000202165	41.32604825
Calcium channel blockers	rs11105352	A	G	0.166874	-0.07447448	0.010823489	6E-12	204378	0.000231604	47.34522461
Calcium channel blockers	rs3184504	T	C	0.481977	0.056394442	0.008023773	2.1E-12	204378	0.000241644	49.39820131
Calcium channel blockers	rs35429	G	A	0.382183	-0.07223407	0.008266229	2.4E-18	204378	0.000373485	76.35983935
Calcium channel blockers	rs4981000	C	T	0.49161	-0.048286333	0.00807691	2.3E-09	204378	0.000174843	35.73993484
Calcium channel blockers	rs7985544	G	T	0.461277	-0.04786397	0.008232905	6.1E-09	204378	0.00016535	33.79924359
Calcium channel blockers	rs7983337	C	T	0.493522	-0.077298487	0.008079718	1.1E-21	204378	0.000447632	91.5261716
Calcium channel blockers	rs8009025	C	T	0.206232	0.057095396	0.009965932	0.00000001	204378	0.000160569	32.8217749
Calcium channel blockers	rs2168518	G	A	0.321597	0.05984615	0.008581712	3.1E-12	204378	0.000237896	48.63179881
Calcium channel blockers	rs7174222	T	C	0.468408	0.06535783	0.00806947	5.5E-16	204378	0.000320872	65.59955945
Calcium channel blockers	rs1894400	T	C	0.323994	0.075941215	0.008576929	8.4E-19	204378	0.000383434	78.39478187

Calcium channel blockers	rs13329952	C	T	0.192068	-0.055942094	0.010179543	0.000000039	204378	0.000147748	30.2006714
Calcium channel blockers	rs62033406	G	A	0.407052	0.055290178	0.00816952	1.3E-11	204378	0.000224064	45.80349823
Calcium channel blockers	rs7203816	A	G	0.31252	-0.052671659	0.008698537	1.4E-09	204378	0.00017937	36.66547208
Calcium channel blockers	rs460879	T	C	0.430755	-0.067857549	0.008151074	8.4E-17	204378	0.000338989	69.30465993
Calcium channel blockers	rs12938899	T	C	0.199939	-0.058853246	0.010041159	4.6E-09	204378	0.000168061	34.35333348
Calcium channel blockers	rs17608766	C	T	0.145965	0.067002878	0.011356909	3.6E-09	204378	0.000170278	34.80666232
Calcium channel blockers	rs11653468	A	G	0.235338	-0.056122836	0.009464622	0.000000003	204378	0.000172014	35.16158246
Calcium channel blockers	rs4305	A	G	0.444171	0.059748909	0.008193735	3.1E-13	204378	0.000260105	53.17308414
Calcium channel blockers	rs72915163	T	C	0.254784	0.057151266	0.009350833	9.8E-10	204378	0.000182742	37.35483919
Calcium channel blockers	rs12978472	G	C	0.127677	-0.108858108	0.012116541	2.6E-19	204378	0.000394783	80.71606713
Calcium channel blockers	rs12463045	T	C	0.158528	0.062711073	0.011115742	0.000000017	204378	0.000155708	31.82784667
Calcium channel blockers	rs7412	T	C	0.082871	-0.091432187	0.014629545	4.1E-10	204378	0.000191082	39.06000983
Calcium channel blockers	rs2207070	A	G	0.22629	-0.054568197	0.0095983	0.000000013	204378	0.00015812	32.32111588
Calcium channel blockers	rs6078002	A	C	0.289318	0.073390788	0.008855823	1.2E-16	204378	0.000335927	68.67852819
Calcium channel blockers	rs6031431	G	A	0.460604	0.0502499	0.008173999	7.9E-10	204378	0.000184879	37.7917518
Calcium channel blockers	rs259983	C	A	0.132896	0.081478097	0.011900197	7.6E-12	204378	0.000229319	46.87801098
Calcium channel	rs6062536	A	G	0.233334	-0.053996746	0.009493088	0.000000013	204378	0.000158277	32.35309871

blockers										
Calcium channel blockers	rs71313931	G	C	0.283681	0.048869627	0.008960731	0.000000049	204378	0.00014551	29.74314743
Agents acting on the renin-angiotensin system	rs35005772	T	C	0.345444	0.034732723	0.006331053	0.000000041	237530	0.000126693	30.09692148
Agents acting on the renin-angiotensin system	rs880315	C	T	0.339584	0.064730866	0.006427611	7.4E-24	237530	0.000426795	101.4191516
Agents acting on the renin-angiotensin system	rs71647010	A	G	0.063423	-0.090900283	0.01230957	1.5E-13	237530	0.000229523	54.53069887
Agents acting on the renin-angiotensin system	rs193084249	G	A	0.0222331	0.135676011	0.020481295	3.5E-11	237530	0.000184711	43.88212727
Agents acting on the renin-angiotensin system	rs61772578	G	A	0.110553	0.067210919	0.00963028	3E-12	237530	0.000205019	48.70776696
Agents acting on the renin-angiotensin system	rs4970834	T	C	0.185684	-0.056275744	0.007776593	4.6E-13	237530	0.000220419	52.36728548
Agents acting on the renin-angiotensin system	rs3790604	A	C	0.0727071	0.10309992	0.011591173	5.9E-19	237530	0.000332965	79.11489614
Agents acting on the renin-angiotensin system	rs146754848	G	T	0.0177044	0.19072176	0.022752644	5.2E-17	237530	0.000295726	70.26403516
Agents acting on the renin-angiotensin system	rs2760061	A	T	0.481262	0.038789546	0.006154759	2.9E-10	237530	0.000167192	39.71948536
Agents acting on the renin-angiotensin system	rs2478543	C	T	0.403731	0.03772458	0.006156097	8.9E-10	237530	0.00015807	37.5521055

Agents acting on the renin-angiotensin system	rs1731243	C	T	0.394283	0.064796713	0.006167826	8.1E-26	237530	0.000464431	110.3667155
Agents acting on the renin-angiotensin system	rs79744918	A	G	0.079463	-0.061117017	0.01117212	0.000000045	237530	0.000125974	29.92605238
Agents acting on the renin-angiotensin system	rs7368883	A	G	0.36012	0.038084781	0.006283983	1.4E-09	237530	0.000154613	36.7307027
Agents acting on the renin-angiotensin system	rs1861410	C	T	0.444736	0.035801703	0.006103934	4.5E-09	237530	0.000144813	34.40203065
Agents acting on the renin-angiotensin system	rs2252867	C	T	0.361669	-0.034094474	0.00624998	0.000000049	237530	0.000125267	29.75823188
Agents acting on the renin-angiotensin system	rs1486195	G	A	0.38746	0.035349059	0.006230534	0.000000014	237530	0.000135496	32.18855363
Agents acting on the renin-angiotensin system	rs76217384	G	A	0.231528	0.043917252	0.007115555	6.7E-10	237530	0.000160348	38.09334892
Agents acting on the renin-angiotensin system	rs268263	T	A	0.244312	-0.045830458	0.007088238	1E-10	237530	0.000175969	41.80498668
Agents acting on the renin-angiotensin system	rs10931284	C	T	0.361893	-0.035311814	0.006275495	0.000000018	237530	0.000133281	31.66214929
Agents acting on the renin-angiotensin system	rs2943660	T	G	0.363073	-0.038290346	0.006246173	8.8E-10	237530	0.000158184	37.57914831
Agents acting on the renin-angiotensin system	rs1000010	G	A	0.351806	-0.037241893	0.00638692	5.5E-09	237530	0.00014312	33.99983926

Agents acting on the renin-angiotensin system	rs2643826	T	C	0.453083	0.053948991	0.006072657	6.5E-19	237530	0.000332159	78.92335434
Agents acting on the renin-angiotensin system	rs6800730	A	G	0.332275	-0.046658119	0.006380335	2.6E-13	237530	0.000225087	53.47659847
Agents acting on the renin-angiotensin system	rs7340705	C	T	0.322424	0.041461943	0.006501483	1.8E-10	237530	0.000171191	40.6696829
Agents acting on the renin-angiotensin system	rs7631008	C	T	0.325219	-0.035248642	0.006438414	0.000000044	237530	0.000126169	29.97252271
Agents acting on the renin-angiotensin system	rs6766859	C	T	0.371212	0.040738042	0.006306927	1.1E-10	237530	0.000175618	41.72161097
Agents acting on the renin-angiotensin system	rs9844972	C	G	0.0671097	0.070060845	0.01214377	0.000000008	237530	0.000140108	33.284345
Agents acting on the renin-angiotensin system	rs77463690	G	A	0.0722319	-0.069927094	0.011657379	0.000000002	237530	0.000151462	35.98201235
Agents acting on the renin-angiotensin system	rs1290786	T	C	0.429711	0.038310872	0.006089684	3.2E-10	237530	0.000166596	39.5777297
Agents acting on the renin-angiotensin system	rs10155132	A	G	0.39295	-0.034312642	0.006186808	0.000000029	237530	0.000129479	30.758942
Agents acting on the renin-angiotensin system	rs28667801	T	A	0.406024	0.03892911	0.006205996	3.5E-10	237530	0.000165629	39.34796731
Agents acting on the renin-angiotensin system	rs10213458	A	G	0.319084	0.044955829	0.006448394	3.1E-12	237530	0.000204579	48.60323874

Agents acting on the renin-angiotensin system	rs13125101	A	G	0.2921	0.117785783	0.006652725	3.8E-70	237530	0.001317939	313.4606303
Agents acting on the renin-angiotensin system	rs13107325	T	C	0.0745797	-0.071593837	0.011465807	4.3E-10	237530	0.000164117	38.98866784
Agents acting on the renin-angiotensin system	rs13118687	A	G	0.470423	-0.042658706	0.006081145	2.3E-12	237530	0.000207127	49.20860245
Agents acting on the renin-angiotensin system	rs9683944	G	A	0.204222	0.043979446	0.007481672	4.1E-09	237530	0.000145452	34.55401347
Agents acting on the renin-angiotensin system	rs300934	T	G	0.312643	0.043723107	0.006509148	1.9E-11	237530	0.000189921	45.12010391
Agents acting on the renin-angiotensin system	rs4691666	C	T	0.466701	0.039503828	0.006045701	6.4E-11	237530	0.000179717	42.69543387
Agents acting on the renin-angiotensin system	rs56329057	T	C	0.183334	-0.057476931	0.007785802	1.6E-13	237530	0.000229384	54.49753126
Agents acting on the renin-angiotensin system	rs10059884	C	A	0.403208	-0.058516588	0.006140458	1.6E-21	237530	0.000382184	90.81399581
Agents acting on the renin-angiotensin system	rs458356	A	G	0.236286	-0.050707637	0.007393448	7E-12	237530	0.000197992	47.03802138
Agents acting on the renin-angiotensin system	rs3814424	T	C	0.157566	0.049400067	0.008257171	2.2E-09	237530	0.000150664	35.79223821
Agents acting on the renin-angiotensin system	rs28081	A	G	0.150332	-0.05214078	0.008431305	6.2E-10	237530	0.000160982	38.24386972

Agents acting on the renin-angiotensin system	rs1422278	T	G	0.131869	0.053985	0.008887087	1.2E-09	237530	0.000155325	36.89977426
Agents acting on the renin-angiotensin system	rs11743404	C	T	0.360119	-0.045967347	0.006289591	2.7E-13	237530	0.000224822	53.41343032
Agents acting on the renin-angiotensin system	rs7716114	G	A	0.287064	-0.042465127	0.006743932	3E-10	237530	0.000166897	39.6492322
Agents acting on the renin-angiotensin system	rs2984644	A	G	0.269037	0.044590843	0.00679841	5.4E-11	237530	0.000181084	43.02025327
Agents acting on the renin-angiotensin system	rs7701003	G	A	0.369354	-0.055057066	0.006235318	1E-18	237530	0.000328131	77.96598958
Agents acting on the renin-angiotensin system	rs36071027	T	C	0.357521	-0.036414643	0.006290722	7.1E-09	237530	0.000141049	33.50791649
Agents acting on the renin-angiotensin system	rs2655438	C	G	0.295517	-0.040770862	0.006583791	5.9E-10	237530	0.000161421	38.34811453
Agents acting on the renin-angiotensin system	rs1799945	G	C	0.152431	0.065564238	0.008364172	4.6E-15	237530	0.000258617	61.44470896
Agents acting on the renin-angiotensin system	rs3099849	G	A	0.181898	-0.048254952	0.007807562	6.4E-10	237530	0.000160792	38.19875983
Agents acting on the renin-angiotensin system	rs9472136	T	C	0.398083	-0.035204247	0.006207591	0.000000014	237530	0.000135384	32.16179648
Agents acting on the renin-angiotensin system	rs13205180	T	C	0.493653	0.036774742	0.00603891	1.1E-09	237530	0.000156097	37.08330668

Agents acting on the renin-angiotensin system	rs11752759	C	A	0.438169	0.063558487	0.006059693	9.7E-26	237530	0.000462942	110.0125395
Agents acting on the renin-angiotensin system	rs2105092	A	G	0.28919	-0.043208886	0.006663373	8.9E-11	237530	0.000176996	42.04885828
Agents acting on the renin-angiotensin system	rs57139556	G	A	0.070976	-0.09007257	0.011714481	1.5E-14	237530	0.000248836	59.12012927
Agents acting on the renin-angiotensin system	rs55730499	T	C	0.0795143	0.086630025	0.011135236	7.3E-15	237530	0.000254747	60.52494172
Agents acting on the renin-angiotensin system	rs73033340	G	A	0.0317516	-0.095375799	0.017169671	0.000000028	237530	0.000129891	30.85665124
Agents acting on the renin-angiotensin system	rs78745308	G	C	0.0449878	0.081932792	0.014506791	0.000000016	237530	0.000134275	31.89841962
Agents acting on the renin-angiotensin system	rs7803355	T	C	0.114798	0.062577989	0.009447452	3.5E-11	237530	0.000184678	43.87429426
Agents acting on the renin-angiotensin system	rs3735533	T	C	0.0732746	-0.099081216	0.011582067	1.2E-17	237530	0.000308005	73.18243308
Agents acting on the renin-angiotensin system	rs6961048	G	C	0.101599	0.078311225	0.009979678	4.3E-15	237530	0.00025917	61.5759736
Agents acting on the renin-angiotensin system	rs35301188	A	G	0.169551	0.053642906	0.008088387	3.3E-11	237530	0.000185141	43.98423537
Agents acting on the renin-angiotensin system	rs34748838	T	C	0.489913	-0.04805376	0.006040814	1.8E-15	237530	0.000266336	63.27908866

Agents acting on the renin-angiotensin system	rs1722883	C	T	0.47102	-0.037937707	0.006060722	3.9E-10	237530	0.000164931	39.18228626
Agents acting on the renin-angiotensin system	rs3918226	T	C	0.0773003	0.145321004	0.011365751	2E-37	237530	0.00068777	163.4770815
Agents acting on the renin-angiotensin system	rs10254101	T	C	0.288171	0.044029603	0.006641664	3.4E-11	237530	0.000184985	43.94728292
Agents acting on the renin-angiotensin system	rs7460226	G	A	0.409371	0.044415886	0.006196788	7.6E-13	237530	0.000216237	51.37356975
Agents acting on the renin-angiotensin system	rs73563812	T	G	0.23594	-0.043877435	0.007101356	6.5E-10	237530	0.000160698	38.17650792
Agents acting on the renin-angiotensin system	rs67529638	G	C	0.348627	-0.042794361	0.006348081	1.6E-11	237530	0.000191288	45.44483468
Agents acting on the renin-angiotensin system	rs2247355	T	C	0.181421	-0.043164491	0.007826184	0.000000035	237530	0.00012805	30.41931841
Agents acting on the renin-angiotensin system	rs13280592	C	G	0.270034	-0.038423017	0.006861119	0.000000021	237530	0.000132013	31.36096198
Agents acting on the renin-angiotensin system	rs12549801	A	G	0.233986	-0.040760934	0.007146626	0.000000012	237530	0.000136933	32.52988188
Agents acting on the renin-angiotensin system	rs10217586	A	T	0.467959	-0.051771935	0.006058458	1.3E-17	237530	0.000307335	73.02320227
Agents acting on the renin-angiotensin system	rs563132	T	A	0.390895	-0.033873682	0.006169883	0.00000004	237530	0.000126881	30.14166529

Agents acting on the renin-angiotensin system	rs111820018	A	G	0.0960907	0.067282939	0.010229124	4.8E-11	237530	0.000182111	43.26426747
Agents acting on the renin-angiotensin system	rs10817003	A	G	0.0355255	0.101508797	0.016295196	4.7E-10	237530	0.000163342	38.80471762
Agents acting on the renin-angiotensin system	rs1888693	A	G	0.342875	0.040535718	0.006343554	1.7E-10	237530	0.000171877	40.83257805
Agents acting on the renin-angiotensin system	rs7070847	A	G	0.283561	-0.052491619	0.006706791	5E-15	237530	0.000257822	61.25573429
Agents acting on the renin-angiotensin system	rs57541197	A	G	0.144319	-0.096298166	0.008556207	2.2E-29	237530	0.000532995	126.6688326
Agents acting on the renin-angiotensin system	rs2236295	T	G	0.402916	-0.04304643	0.006192724	3.6E-12	237530	0.000203378	48.31780369
Agents acting on the renin-angiotensin system	rs2901761	A	G	0.418024	0.043702376	0.006106506	8.3E-13	237530	0.000215582	51.21779419
Agents acting on the renin-angiotensin system	rs79668541	T	C	0.0759178	-0.093270249	0.011364979	2.3E-16	237530	0.00028347	67.35120048
Agents acting on the renin-angiotensin system	rs6584606	A	C	0.0157173	0.169909072	0.02421907	2.3E-12	237530	0.000207162	49.21693452
Agents acting on the renin-angiotensin system	rs1801253	G	C	0.259296	-0.050945097	0.006921153	1.8E-13	237530	0.00022805	54.18065861
Agents acting on the renin-angiotensin system	rs7938342	T	A	0.415375	-0.067405373	0.006136446	4.5E-28	237530	0.000507711	120.6567415

Agents acting on the renin-angiotensin system	rs415895	C	G	0.353354	-0.052075962	0.006300548	1.4E-16	237530	0.000287524	68.31472386
Agents acting on the renin-angiotensin system	rs56175671	C	A	0.0917925	0.06036904	0.01050424	9.1E-09	237530	0.000139034	33.02901595
Agents acting on the renin-angiotensin system	rs7940934	G	A	0.487472	0.033584574	0.006039219	0.000000027	237530	0.00013018	30.92534007
Agents acting on the renin-angiotensin system	rs2856653	T	C	0.358513	0.045660182	0.006321691	5.1E-13	237530	0.000219581	52.16811301
Agents acting on the renin-angiotensin system	rs751984	C	T	0.112856	-0.058711041	0.009556717	8.1E-10	237530	0.000158867	37.74144316
Agents acting on the renin-angiotensin system	rs3802932	A	G	0.0576598	0.08005359	0.01291788	5.7E-10	237530	0.000161655	38.40391383
Agents acting on the renin-angiotensin system	rs2306363	T	G	0.205555	-0.055492786	0.007455591	9.8E-14	237530	0.000233179	55.39942696
Agents acting on the renin-angiotensin system	rs604723	T	C	0.271336	-0.086341431	0.006842239	1.7E-36	237530	0.000669935	159.2349108
Agents acting on the renin-angiotensin system	rs4459340	G	A	0.275503	0.047695616	0.006775981	1.9E-12	237530	0.000208547	49.54603065
Agents acting on the renin-angiotensin system	rs12366015	A	G	0.203816	0.044017771	0.007511509	4.6E-09	237530	0.000144551	34.33982295
Agents acting on the renin-angiotensin system	rs3819536	G	A	0.295117	-0.036672422	0.006600922	0.000000028	237530	0.000129926	30.86500562

Agents acting on the renin-angiotensin system	rs73075659	G	A	0.343772	-0.042514512	0.006410327	3.3E-11	237530	0.000185146	43.98559213
Agents acting on the renin-angiotensin system	rs10876531	C	A	0.286877	-0.040295171	0.006720886	0.000000002	237530	0.00015131	35.94589344
Agents acting on the renin-angiotensin system	rs478863	G	A	0.274025	0.046254037	0.006766207	8.1E-12	237530	0.0001967	46.73099689
Agents acting on the renin-angiotensin system	rs7299436	G	T	0.157439	-0.062372733	0.008288963	5.3E-14	237530	0.000238324	56.62204264
Agents acting on the renin-angiotensin system	rs7310615	C	G	0.484183	0.065732456	0.006073943	2.7E-27	237530	0.000492817	117.1155807
Agents acting on the renin-angiotensin system	rs35441	T	C	0.380577	-0.066196469	0.006191438	1.1E-26	237530	0.000481016	114.3096662
Agents acting on the renin-angiotensin system	rs4981000	C	T	0.491405	-0.033718685	0.006057584	0.000000026	237530	0.000130427	30.98408807
Agents acting on the renin-angiotensin system	rs9506725	C	T	0.370015	-0.043205131	0.006246842	4.6E-12	237530	0.000201346	47.83502414
Agents acting on the renin-angiotensin system	rs3803266	G	C	0.231717	0.044118753	0.007159075	7.2E-10	237530	0.000159862	37.97772713
Agents acting on the renin-angiotensin system	rs28637873	T	C	0.0771092	-0.062732317	0.01133149	0.000000031	237530	0.000129013	30.64820087
Agents acting on the renin-angiotensin system	rs72683923	C	T	0.0199679	-0.142640324	0.021553926	3.6E-11	237530	0.000184346	43.79536915

Agents acting on the renin-angiotensin system	rs989501	G	A	0.351584	-0.035428743	0.006333831	0.000000022	237530	0.000131705	31.28778801
Agents acting on the renin-angiotensin system	rs7149242	T	G	0.281233	-0.036728752	0.006697017	0.000000041	237530	0.000126612	30.07778939
Agents acting on the renin-angiotensin system	rs1757463	G	A	0.284522	0.042849096	0.006746813	2.1E-10	237530	0.000169783	40.33502098
Agents acting on the renin-angiotensin system	rs11072508	C	T	0.327501	0.060410708	0.006462952	9E-21	237530	0.000367695	87.36998881
Agents acting on the renin-angiotensin system	rs7174222	T	C	0.468424	0.037073058	0.006064014	9.7E-10	237530	0.000157329	37.37599206
Agents acting on the renin-angiotensin system	rs1894400	T	C	0.325947	0.076663516	0.006426685	8.4E-33	237530	0.000598721	142.2983072
Agents acting on the renin-angiotensin system	rs77924615	A	G	0.19261	-0.064609976	0.007734256	6.6E-17	237530	0.000293708	69.78441243
Agents acting on the renin-angiotensin system	rs9939774	T	C	0.405631	0.035332648	0.00614231	8.8E-09	237530	0.000139287	33.08912257
Agents acting on the renin-angiotensin system	rs62039768	A	C	0.0957964	0.059182257	0.01031169	9.5E-09	237530	0.000138658	32.93970602
Agents acting on the renin-angiotensin system	rs55872725	T	C	0.402662	0.05885148	0.006133771	8.4E-22	237530	0.000387412	92.05684045
Agents acting on the renin-angiotensin system	rs62043959	C	A	0.278368	-0.048664179	0.006790334	7.7E-13	237530	0.000216184	51.36093202

Agents acting on the renin-angiotensin system	rs164749	G	T	0.430761	-0.048410926	0.006097195	2E-15	237530	0.000265334	63.04098446
Agents acting on the renin-angiotensin system	rs9897348	C	A	0.395725	-0.037037357	0.006194936	2.2E-09	237530	0.000150461	35.74396006
Agents acting on the renin-angiotensin system	rs12453576	T	C	0.177701	0.051418267	0.007874283	6.6E-11	237530	0.00017948	42.63921254
Agents acting on the renin-angiotensin system	rs74439044	C	T	0.0972493	0.057803078	0.010142134	0.000000012	237530	0.00013673	32.48175901
Agents acting on the renin-angiotensin system	rs9907781	C	G	0.242838	0.04417426	0.007034687	3.4E-10	237530	0.000165981	39.43169033
Agents acting on the renin-angiotensin system	rs17637472	A	G	0.39989	0.048654096	0.006254095	7.3E-15	237530	0.00025473	60.5210151
Agents acting on the renin-angiotensin system	rs11079428	T	A	0.223194	-0.044247206	0.007350751	1.8E-09	237530	0.000152519	36.23303095
Agents acting on the renin-angiotensin system	rs4277405	C	T	0.375555	0.056869393	0.006227962	6.8E-20	237530	0.000350909	83.37994278
Agents acting on the renin-angiotensin system	rs72915163	T	C	0.255546	0.048985953	0.007011692	2.8E-12	237530	0.000205442	48.80831744
Agents acting on the renin-angiotensin system	rs12985940	C	T	0.164948	-0.05392687	0.008127895	3.2E-11	237530	0.000185291	44.02005214
Agents acting on the renin-angiotensin system	rs167479	T	G	0.471256	-0.06187271	0.006038653	1.2E-24	237530	0.000441782	104.9819091

Agents acting on the renin-angiotensin system	rs12459970	A	G	0.235734	0.041964333	0.007129444	0.000000004	237530	0.000145837	34.6454059
Agents acting on the renin-angiotensin system	rs7412	T	C	0.0820167	-0.103000636	0.011019644	9E-21	237530	0.000367677	87.36571142
Agents acting on the renin-angiotensin system	rs516246	C	T	0.490532	-0.034396494	0.006050382	0.000000013	237530	0.000136046	32.31908918
Agents acting on the renin-angiotensin system	rs6038468	C	T	0.291906	0.038192194	0.006636469	8.7E-09	237530	0.000139411	33.11856086
Agents acting on the renin-angiotensin system	rs6039216	C	T	0.360894	-0.057690418	0.006283675	4.3E-20	237530	0.000354738	84.29005593
Agents acting on the renin-angiotensin system	rs2103805	T	A	0.354074	-0.042468523	0.006343863	2.2E-11	237530	0.000188637	44.81497082
Agents acting on the renin-angiotensin system	rs6031431	G	A	0.461195	0.038108085	0.006128266	5E-10	237530	0.000162768	38.66832381
Agents acting on the renin-angiotensin system	rs6068191	A	C	0.390648	0.034654016	0.006231769	0.000000027	237530	0.00013017	30.92294209
Agents acting on the renin-angiotensin system	rs6026739	T	A	0.11701	0.103485741	0.009419107	4.4E-28	237530	0.000507928	120.7085067
Agents acting on the renin-angiotensin system	rs8118848	A	G	0.236442	-0.059723948	0.007107529	4.4E-17	237530	0.000297175	70.60834363
Agents acting on the renin-angiotensin system	rs2229742	C	G	0.103481	0.056737185	0.009906064	0.00000001	237530	0.000138088	32.80421575

Agents acting on the renin-angiotensin system	rs76346476	A	G	0.037629	0.090308692	0.015856853	0.000000012	237530	0.000136536	32.4355645
Agents acting on the renin-angiotensin system	rs762395	A	G	0.335664	-0.038521787	0.006441346	2.2E-09	237530	0.000150548	35.76480607
HMG CoA reductase inhibitors	rs12725767	G	A	0.354298	0.035128029	0.005856082	0.000000002	290385	0.000123898	35.98240727
HMG CoA reductase inhibitors	rs79598313	T	C	0.0236446	0.146636976	0.018408443	1.6E-15	290385	0.000218466	63.45266577
HMG CoA reductase inhibitors	rs11591147	T	G	0.017203	-0.362323999	0.021494841	9.4E-64	290385	0.000977523	284.1336862
HMG CoA reductase inhibitors	rs693668	G	A	0.350319	-0.048941477	0.005866453	7.3E-17	290385	0.000239621	69.59852508
HMG CoA reductase inhibitors	rs113911544	G	A	0.039372	0.085085202	0.014373915	3.2E-09	290385	0.000120651	35.0392626
HMG CoA reductase inhibitors	rs7534572	C	G	0.352475	-0.074218207	0.005852643	7.5E-37	290385	0.00055348	160.8102813
HMG CoA reductase inhibitors	rs12740374	T	G	0.219778	-0.156618273	0.006742468	2.3E-119	290385	0.00185467	539.5653663
HMG CoA reductase inhibitors	rs570530	A	G	0.495536	-0.032541516	0.005613812	6.8E-09	290385	0.000115701	33.60136178
HMG CoA reductase inhibitors	rs1367117	A	G	0.337599	0.105726091	0.005900949	8.7E-72	290385	0.001104248	321.0092851
HMG CoA reductase inhibitors	rs1260326	T	C	0.395882	0.059920058	0.005712328	9.7E-26	290385	0.000378773	110.0310156
HMG CoA reductase inhibitors	rs4299376	G	T	0.324311	0.086690463	0.005971001	9.2E-48	290385	0.000725369	210.7877579
HMG CoA reductase inhibitors	rs4541244	T	C	0.421979	-0.034275082	0.005711376	0.000000002	290385	0.000124007	36.01409375
HMG CoA reductase inhibitors	rs148566631	A	G	0.118597	0.048858199	0.008647989	0.000000016	290385	0.000109906	31.91844333
HMG CoA reductase	rs10804330	C	T	0.429504	-0.041844942	0.005740635	3.1E-13	290385	0.000182942	53.13285

inhibitors										
HMG CoA reductase inhibitors	rs4518111	A	C	0.433772	0.037062173	0.005718625	9.1E-11	290385	0.000144624	42.00254356
HMG CoA reductase inhibitors	rs34850939	A	G	0.349518	0.032981402	0.005867987	0.000000019	290385	0.000108777	31.59053309
HMG CoA reductase inhibitors	rs645040	G	T	0.225004	-0.042834024	0.006691199	1.5E-10	290385	0.000141102	40.97951741
HMG CoA reductase inhibitors	rs7639816	T	C	0.180878	0.041868539	0.007355367	0.000000013	290385	0.000111569	32.40141297
HMG CoA reductase inhibitors	rs35430985	A	C	0.266787	0.036945826	0.006372475	6.7E-09	290385	0.000115742	33.61332644
HMG CoA reductase inhibitors	rs3135064	T	C	0.450203	-0.034444179	0.005658626	1.2E-09	290385	0.000127579	37.05158131
HMG CoA reductase inhibitors	rs2603192	C	T	0.413377	-0.033421764	0.005784338	7.6E-09	290385	0.000114955	33.38477628
HMG CoA reductase inhibitors	rs13125101	A	G	0.292467	0.03686969	0.006180362	2.4E-09	290385	0.000122542	35.58837301
HMG CoA reductase inhibitors	rs11743303	G	A	0.208058	0.041238974	0.006954898	0.000000003	290385	0.000121062	35.15856796
HMG CoA reductase inhibitors	rs12916	C	T	0.40104	0.058663467	0.005712752	9.7E-25	290385	0.000363005	105.4488316
HMG CoA reductase inhibitors	rs1501908	G	C	0.364761	-0.040749671	0.005813226	2.4E-12	290385	0.000169187	49.13724527
HMG CoA reductase inhibitors	rs2066905	C	G	0.45116	-0.035224747	0.005632965	4E-10	290385	0.000134645	39.10377532
HMG CoA reductase inhibitors	rs750332	C	T	0.180658	-0.055028614	0.007267379	3.7E-14	290385	0.000197407	57.33482968
HMG CoA reductase inhibitors	rs998584	A	C	0.482799	0.038632303	0.005618626	6.2E-12	290385	0.000162778	47.27569073
HMG CoA reductase inhibitors	rs577721086	C	T	0.0483191	0.077321853	0.013085579	3.4E-09	290385	0.000120224	34.91529042
HMG CoA reductase inhibitors	rs2982521	A	T	0.372013	0.032467972	0.00578365	0.00000002	290385	0.000108514	31.51398703

HMG CoA reductase inhibitors	rs463599	T	G	0.141124	0.046339832	0.008044666	8.4E-09	290385	0.000114253	33.18102955
HMG CoA reductase inhibitors	rs117733303	G	A	0.0192847	0.231370332	0.02030772	4.5E-30	290385	0.000446812	129.8045751
HMG CoA reductase inhibitors	rs74617384	T	A	0.0801433	0.181372313	0.010313884	3.2E-69	290385	0.001063803	309.2391955
HMG CoA reductase inhibitors	rs4639414	G	A	0.230148	0.052494586	0.006676649	3.8E-15	290385	0.000212836	61.81714247
HMG CoA reductase inhibitors	rs17725246	C	T	0.183202	0.043688347	0.007218703	1.4E-09	290385	0.00012612	36.62772286
HMG CoA reductase inhibitors	rs799158	T	C	0.0431865	0.079353562	0.013771121	8.3E-09	290385	0.000114333	33.2040638
HMG CoA reductase inhibitors	rs972283	A	G	0.488765	-0.035923041	0.00559905	1.4E-10	290385	0.000141737	41.16370736
HMG CoA reductase inhibitors	rs3918226	T	C	0.0779394	0.068522545	0.01051933	7.3E-11	290385	0.000146101	42.4314473
HMG CoA reductase inhibitors	rs2898476	A	G	0.145041	0.067787108	0.007936732	1.3E-17	290385	0.000251147	72.94706409
HMG CoA reductase inhibitors	rs15285	T	C	0.28486	-0.058676165	0.006203907	3.1E-21	290385	0.000307953	89.45197864
HMG CoA reductase inhibitors	rs1030431	A	G	0.33226	0.03366996	0.005988778	0.000000019	290385	0.00010884	31.60863519
HMG CoA reductase inhibitors	rs2737245	T	G	0.278192	-0.039369802	0.006276604	3.6E-10	290385	0.00013547	39.34359665
HMG CoA reductase inhibitors	rs2954021	A	G	0.495627	0.096635253	0.005595082	7.7E-67	290385	0.001026215	298.301399
HMG CoA reductase inhibitors	rs11787365	C	T	0.400918	0.037016407	0.005727778	1E-10	290385	0.000143807	41.76506773
HMG CoA reductase inhibitors	rs1537371	A	C	0.499383	0.058189402	0.005604606	3E-25	290385	0.000371076	107.7940265
HMG CoA reductase inhibitors	rs1883025	T	C	0.254856	-0.042749952	0.006416125	2.7E-11	290385	0.000152857	44.39383238
HMG CoA reductase	rs2519093	T	C	0.185023	0.083506924	0.007223517	6.5E-31	290385	0.000460017	133.6424813

inhibitors										
HMG CoA reductase inhibitors	rs2068888	A	G	0.450113	-0.035113056	0.005626034	4.3E-10	290385	0.000134122	38.95198507
HMG CoA reductase inhibitors	rs7903146	T	C	0.290995	0.070760069	0.006160627	1.6E-30	290385	0.000454104	131.9239595
HMG CoA reductase inhibitors	rs3802932	A	G	0.0581295	0.08027365	0.011960626	1.9E-11	290385	0.000155095	45.04382166
HMG CoA reductase inhibitors	rs964184	G	C	0.133698	0.160718726	0.008218578	3.7E-85	290385	0.001315206	382.4164478
HMG CoA reductase inhibitors	rs11220464	T	C	0.130142	0.048182021	0.008326513	7.2E-09	290385	0.000115297	33.48425976
HMG CoA reductase inhibitors	rs76895963	G	T	0.0141825	-0.202726945	0.024010456	3.1E-17	290385	0.000245438	71.28844435
HMG CoA reductase inhibitors	rs2293093	G	C	0.111646	0.051159056	0.008895074	8.9E-09	290385	0.0001139	33.07828054
HMG CoA reductase inhibitors	rs1169288	C	A	0.313809	0.048448524	0.006082533	1.6E-15	290385	0.000218435	63.44379083
HMG CoA reductase inhibitors	rs221907	A	G	0.360751	-0.033361871	0.005826718	0.00000001	290385	0.000112883	32.78308571
HMG CoA reductase inhibitors	rs10468017	T	C	0.297184	0.037635972	0.006131474	8.3E-10	290385	0.000129732	37.67675421
HMG CoA reductase inhibitors	rs11864054	G	A	0.385154	-0.034885282	0.005811639	1.9E-09	290385	0.000124068	36.03165927
HMG CoA reductase inhibitors	rs1421085	C	T	0.403509	0.044655313	0.005696826	4.6E-15	290385	0.000211551	61.44370155
HMG CoA reductase inhibitors	rs11641811	C	A	0.476408	-0.033493562	0.005619208	2.5E-09	290385	0.000122333	35.52788773
HMG CoA reductase inhibitors	rs2287997	A	G	0.18536	0.064653304	0.007201719	2.8E-19	290385	0.000277469	80.59463292
HMG CoA reductase inhibitors	rs72837687	A	G	0.199899	-0.044388387	0.007016908	2.5E-10	290385	0.000137789	40.01697275
HMG CoA reductase inhibitors	rs7213086	C	G	0.44498	-0.033201609	0.005647779	4.1E-09	290385	0.000118997	34.55889423

HMG CoA reductase inhibitors	rs11653468	A	G	0.233873	-0.038664313	0.006608344	4.9E-09	290385	0.000117872	34.23200712
HMG CoA reductase inhibitors	rs77542162	G	A	0.0230779	0.151112025	0.018663359	5.6E-16	290385	0.000225708	65.5564508
HMG CoA reductase inhibitors	rs72631343	G	C	0.128205	-0.04882312	0.008360322	5.2E-09	290385	0.00011743	34.10374485
HMG CoA reductase inhibitors	rs6511720	T	G	0.117712	-0.198647127	0.008684178	8.3E-116	290385	0.001798668	523.2438338
HMG CoA reductase inhibitors	rs2738447	A	C	0.406512	-0.062571332	0.005699048	4.8E-28	290385	0.000414946	120.5433175
HMG CoA reductase inhibitors	rs58542926	T	C	0.074933	-0.12420776	0.010639856	1.7E-31	290385	0.000469081	136.2771138
HMG CoA reductase inhibitors	rs28399664	G	C	0.0110032	0.157816134	0.02719045	6.5E-09	290385	0.000115997	33.68736225
HMG CoA reductase inhibitors	rs148601586	G	C	0.0122258	0.160733012	0.025484556	2.8E-10	290385	0.000136969	39.77892386
HMG CoA reductase inhibitors	rs116881820	C	T	0.025925	0.202944931	0.017623061	1.1E-30	290385	0.000456479	132.6144168
HMG CoA reductase inhibitors	rs7412	T	C	0.0792273	-0.310311732	0.010360179	4.1E-197	290385	0.003079981	897.1372121
HMG CoA reductase inhibitors	rs602662	G	A	0.462426	-0.043165394	0.005623177	1.6E-14	290385	0.000202883	58.92572997
HMG CoA reductase inhibitors	rs2618567	G	T	0.340873	0.040058837	0.005911214	1.2E-11	290385	0.000158125	45.92407073
HMG CoA reductase inhibitors	rs2207132	A	G	0.0335157	0.159389121	0.015550983	1.2E-24	290385	0.000361635	105.0506347
HMG CoA reductase inhibitors	rs1997833	C	T	0.300865	0.043514858	0.006103062	1E-12	290385	0.000175037	50.83661168
HMG CoA reductase inhibitors	rs8126001	T	C	0.489093	-0.046777285	0.005628309	9.5E-17	290385	0.000237814	69.07345671
Thyroid preparations	rs2234167	A	G	0.133222	0.081784837	0.013360309	9.3E-10	305582	0.000122612	37.47224929
Thyroid preparations	rs2473808	T	C	0.33565	0.055621753	0.009684348	9.3E-09	305582	0.000107938	32.98722378
Thyroid preparations	rs61776678	A	G	0.410504	-0.061821667	0.009273056	2.6E-11	305582	0.000145427	44.44601531

Thyroid preparations	rs61778692	G	A	0.245765	-0.065728844	0.010585182	5.3E-10	305582	0.000126163	38.55782492
Thyroid preparations	rs72922282	C	G	0.129635	-0.078571094	0.013553498	6.7E-09	305582	0.000109963	33.60624497
Thyroid preparations	rs10489626	G	C	0.163317	0.075578895	0.012304875	8.1E-10	305582	0.000123443	37.72626464
Thyroid preparations	rs78458460	T	G	0.239999	0.091465314	0.010684515	1.1E-17	305582	0.000239757	73.28251599
Thyroid preparations	rs2476601	A	G	0.102931	0.413973244	0.014925497	2.6E-169	305582	0.002511119	769.2794265
Thyroid preparations	rs1723022	T	G	0.370454	0.059215498	0.009605119	7E-10	305582	0.000124361	38.0068987
Thyroid preparations	rs12742404	G	A	0.121153	0.087284889	0.013958012	4E-10	305582	0.000127952	39.10462915
Thyroid preparations	rs11675342	T	C	0.423396	0.09530659	0.009231734	5.5E-25	305582	0.000348658	106.5801852
Thyroid preparations	rs13398375	C	T	0.289167	-0.062139519	0.010167943	9.9E-10	305582	0.000122205	37.34794811
Thyroid preparations	rs1534430	T	C	0.389139	-0.08207014	0.00935578	1.8E-18	305582	0.000251752	76.94979652
Thyroid preparations	rs10172181	C	T	0.0535539	0.111839025	0.020185921	0.00000003	305582	0.000100443	30.69635321
Thyroid preparations	rs7583027	A	C	0.357133	-0.059402887	0.009520573	4.4E-10	305582	0.000127381	38.93015232
Thyroid preparations	rs5865	C	T	0.334138	0.06017709	0.009663693	4.8E-10	305582	0.00012688	38.77691496
Thyroid preparations	rs2111485	A	G	0.391151	-0.068354703	0.009314726	2.2E-13	305582	0.000176194	53.85100027
Thyroid preparations	rs4366932	T	C	0.403833	0.052358316	0.009310279	0.000000019	305582	0.000103484	31.62591374
Thyroid preparations	rs11889341	T	C	0.222787	0.143350669	0.010942145	3.3E-39	305582	0.000561336	171.6293937
Thyroid preparations	rs3087243	A	G	0.448827	-0.178713501	0.009145473	4.9E-85	305582	0.001248051	381.855915
Thyroid preparations	rs7596240	G	A	0.270163	0.063452716	0.01022874	5.5E-10	305582	0.000125914	38.48161399
Thyroid preparations	rs75051580	C	T	0.201386	0.062789152	0.011461651	0.000000043	305582	9.81984E-05	30.01041757
Thyroid preparations	rs145268310	C	G	0.121225	0.102305218	0.013963637	2.4E-13	305582	0.000175628	53.67791177
Thyroid preparations	rs11714050	G	A	0.126095	0.090516447	0.01369856	3.9E-11	305582	0.000142862	43.66186851
Thyroid preparations	rs13090803	T	G	0.21057	0.094675173	0.011272253	4.5E-17	305582	0.000230793	70.54202787
Thyroid preparations	rs2969903	T	C	0.233721	0.062769596	0.010745875	5.2E-09	305582	0.000111645	34.12025165
Thyroid preparations	rs4688013	A	G	0.191322	0.083276851	0.011549827	5.6E-13	305582	0.000170097	51.98700391
Thyroid preparations	rs12634152	C	T	0.45193	0.142340723	0.009196319	4.9E-54	305582	0.000783361	239.56726
Thyroid preparations	rs9291444	T	C	0.471241	0.07350931	0.009131422	8.3E-16	305582	0.000212025	64.80447413
Thyroid preparations	rs7441808	G	A	0.301669	0.08148547	0.009910206	2E-16	305582	0.000221193	67.60707898
Thyroid preparations	rs13136820	C	T	0.319992	0.070655075	0.009885372	8.8E-13	305582	0.000167148	51.08552108
Thyroid preparations	rs366327	T	A	0.184729	0.06757675	0.011895218	0.000000013	305582	0.000105603	32.27356734
Thyroid preparations	rs113473633	G	A	0.0221276	-0.178552767	0.031151332	9.9E-09	305582	0.000107499	32.85315541
Thyroid preparations	rs6833591	G	A	0.346625	-0.058954707	0.009598676	8.2E-10	305582	0.000123433	37.72345349
Thyroid preparations	rs7655751	T	C	0.211042	-0.131974448	0.011143826	2.3E-32	305582	0.000458758	140.2517251
Thyroid preparations	rs35782497	A	G	0.339168	-0.083797495	0.009701988	5.8E-18	305582	0.000244066	74.59981676

Thyroid preparations	rs12697352	A	G	0.33964	-0.060907224	0.009645008	2.7E-10	305582	0.000130481	39.87765413
Thyroid preparations	rs13358767	G	A	0.285135	-0.057781751	0.01020388	0.000000015	305582	0.000104925	32.06622591
Thyroid preparations	rs10036386	T	C	0.381268	0.064672821	0.009382382	5.5E-12	305582	0.000155461	47.51323161
Thyroid preparations	rs1991797	T	G	0.328168	-0.067278454	0.009731536	4.7E-12	305582	0.000156384	47.79542755
Thyroid preparations	rs244690	G	A	0.123038	0.112015565	0.013847775	6E-16	305582	0.00021408	65.4325606
Thyroid preparations	rs13360007	G	A	0.135676	0.077983611	0.013293483	4.5E-09	305582	0.000112604	34.41329342
Thyroid preparations	rs9391997	A	G	0.470259	-0.073869488	0.009155586	7.1E-16	305582	0.000212979	65.09611097
Thyroid preparations	rs7764777	T	G	0.116	-0.079926483	0.014181968	0.000000017	305582	0.000103929	31.76183488
Thyroid preparations	rs28559870	T	C	0.169018	-0.159113322	0.012300214	2.8E-38	305582	0.000547296	167.3342787
Thyroid preparations	rs2471960	A	G	0.310021	0.107006154	0.009818025	1.2E-27	305582	0.000388573	118.7863199
Thyroid preparations	rs6605556	G	A	0.185368	0.166256612	0.011775149	2.9E-45	305582	0.00065195	199.3527178
Thyroid preparations	rs2856698	G	C	0.328285	0.268845124	0.009665421	2.8E-170	305582	0.002525439	773.6774304
Thyroid preparations	rs2856822	C	A	0.310552	-0.165320336	0.009803224	8.3E-64	305582	0.000929786	284.3884053
Thyroid preparations	rs7754251	G	C	0.418931	-0.120908846	0.009181357	1.3E-39	305582	0.00056719	173.420149
Thyroid preparations	rs6914622	T	G	0.321667	0.089143108	0.009792307	8.8E-20	305582	0.000271119	82.8710121
Thyroid preparations	rs3008034	C	T	0.305208	-0.063043782	0.00985865	1.6E-10	305582	0.000133802	40.8927913
Thyroid preparations	rs1951459	A	G	0.334248	-0.115464516	0.009609606	2.9E-33	305582	0.000472229	144.3720273
Thyroid preparations	rs215619	G	A	0.372532	0.051601124	0.009443782	0.000000047	305582	9.76914E-05	29.85544568
Thyroid preparations	rs10279209	A	G	0.134841	0.083503352	0.013371091	4.2E-10	305582	0.000127612	39.00059878
Thyroid preparations	rs1734911	C	G	0.113645	-0.081313484	0.01433346	0.000000014	305582	0.000105305	32.18253494
Thyroid preparations	rs34591253	T	C	0.135132	0.076426098	0.013319536	9.6E-09	305582	0.000107729	32.92322353
Thyroid preparations	rs2921053	C	G	0.451729	-0.092992823	0.009178196	4E-24	305582	0.000335823	102.655298
Thyroid preparations	rs10098103	C	T	0.242332	-0.060081989	0.010637018	0.000000016	305582	0.000104394	31.90405959
Thyroid preparations	rs1032129	C	A	0.353542	-0.059290909	0.009567829	5.8E-10	305582	0.000125651	38.40135924
Thyroid preparations	rs2445610	G	A	0.36907	-0.069989502	0.009426584	1.1E-13	305582	0.000180364	55.12572499
Thyroid preparations	rs853305	T	C	0.315924	0.071577287	0.0097864	2.6E-13	305582	0.000175025	53.49358135
Thyroid preparations	rs7005834	T	C	0.307655	-0.066507467	0.009841599	1.4E-11	305582	0.000149423	45.66743701
Thyroid preparations	rs7028486	C	T	0.301266	0.06116547	0.009969597	8.5E-10	305582	0.000123162	37.64043209
Thyroid preparations	rs970987	C	A	0.337145	0.079637028	0.009665984	1.7E-16	305582	0.000222082	67.87895318
Thyroid preparations	rs7850258	A	G	0.331608	-0.195523605	0.009675413	8.3E-91	305582	0.001334601	408.3725013
Thyroid preparations	rs2094920	C	G	0.449845	0.052081451	0.009170454	0.000000014	305582	0.000105538	32.25385598
Thyroid preparations	rs2274780	C	T	0.470695	0.053449968	0.009119769	4.6E-09	305582	0.000112396	34.34981186
Thyroid preparations	rs7090530	C	A	0.395908	-0.093977185	0.00931912	6.5E-24	305582	0.000332677	101.6932801

Thyroid preparations	rs744253	A	G	0.258633	-0.081497927	0.010382308	4.2E-15	305582	0.0002016	61.61726701
Thyroid preparations	rs71508903	T	C	0.190444	0.139273382	0.011730452	1.6E-32	305582	0.000461083	140.9626046
Thyroid preparations	rs10761620	A	G	0.449981	0.072898789	0.009220965	2.7E-15	305582	0.00020449	62.5007271
Thyroid preparations	rs10748781	C	A	0.434051	0.053942885	0.009322589	7.2E-09	305582	0.000109552	33.48054
Thyroid preparations	rs7088058	T	C	0.412751	-0.057947976	0.009273498	4.1E-10	305582	0.000127763	39.04689935
Thyroid preparations	rs10751647	C	T	0.40255	-0.05415626	0.009375309	7.6E-09	305582	0.000109182	33.36746844
Thyroid preparations	rs10836367	C	T	0.388263	0.083144647	0.0093819	7.8E-19	305582	0.000256949	78.53878629
Thyroid preparations	rs3781627	T	A	0.299038	0.056455427	0.010139171	0.000000026	305582	0.000101446	31.00299307
Thyroid preparations	rs174599	C	G	0.374506	-0.062605112	0.009437661	3.3E-11	305582	0.000143979	44.0035797
Thyroid preparations	rs479777	C	T	0.340767	-0.057627312	0.009627233	2.2E-09	305582	0.00011724	35.83033631
Thyroid preparations	rs4409785	C	T	0.172241	0.143064027	0.012049951	1.6E-32	305582	0.000461065	140.9572717
Thyroid preparations	rs7931398	C	T	0.259045	-0.062308691	0.010408629	2.1E-09	305582	0.000117255	35.83499886
Thyroid preparations	rs61907718	A	G	0.216956	0.06928254	0.011124578	4.7E-10	305582	0.000126911	38.78623754
Thyroid preparations	rs7302200	A	G	0.340319	0.074568815	0.009635056	1E-14	305582	0.000195972	59.89673979
Thyroid preparations	rs1320344	A	G	0.314107	0.069094347	0.009798616	1.8E-12	305582	0.000162689	49.72247259
Thyroid preparations	rs3184504	T	C	0.484908	0.205206492	0.009094226	9.7E-113	305582	0.001663412	509.1525034
Thyroid preparations	rs7333647	C	T	0.287791	0.124414188	0.010090322	6.2E-35	305582	0.000497263	152.0291836
Thyroid preparations	rs75863106	T	A	0.0304953	0.154907448	0.026969567	9.3E-09	305582	0.00010795	32.99087176
Thyroid preparations	rs9533100	G	T	0.4624	-0.067726902	0.009159497	1.4E-13	305582	0.000178885	54.67342891
Thyroid preparations	rs3784099	A	G	0.28051	-0.064911779	0.01013818	1.5E-10	305582	0.000134135	40.99436656
Thyroid preparations	rs1257926	A	G	0.474691	0.054403924	0.009154541	2.8E-09	305582	0.00011556	35.31705156
Thyroid preparations	rs8043085	T	G	0.232174	0.094800679	0.010843896	2.3E-18	305582	0.000250044	76.42744407
Thyroid preparations	rs1872691	A	G	0.181059	-0.073665624	0.011845738	5E-10	305582	0.000126539	38.67253385
Thyroid preparations	rs8056260	G	A	0.0435819	0.148567829	0.022284839	2.6E-11	305582	0.000145425	44.44548925
Thyroid preparations	rs8054578	A	G	0.223812	0.069831447	0.010947476	1.8E-10	305582	0.000133134	40.68845778
Thyroid preparations	rs35776863	A	G	0.229451	0.088579869	0.010950075	6E-16	305582	0.000214099	65.43845988
Thyroid preparations	rs12325861	C	T	0.166127	0.0937906	0.012218159	1.6E-14	305582	0.000192795	58.92562849
Thyroid preparations	rs73316435	T	C	0.179924	0.090641418	0.011906228	2.7E-14	305582	0.000189625	57.9564997
Thyroid preparations	rs6505765	G	C	0.344388	0.075718868	0.009598114	3E-15	305582	0.00020362	62.23484315
Thyroid preparations	rs56249713	C	T	0.418115	-0.057151405	0.009335863	9.3E-10	305582	0.000122621	37.47503718
Thyroid preparations	rs7240256	C	T	0.317032	0.05994282	0.009862602	1.2E-09	305582	0.000120868	36.93928788
Thyroid preparations	rs72977594	A	G	0.149821	-0.075491429	0.0128825	4.6E-09	305582	0.000112362	34.33935697
Thyroid preparations	rs7254729	T	C	0.406483	0.078747633	0.009273444	2E-17	305582	0.000235919	72.1090944

Thyroid preparations	rs144309607	T	C	0.0435982	-0.146562671	0.022486159	7.1E-11	305582	0.000139004	42.48282154
Thyroid preparations	rs11666808	T	C	0.373327	-0.07337282	0.009466754	9.1E-15	305582	0.000196542	60.07107693
Thyroid preparations	rs12980063	G	A	0.391542	-0.061521228	0.009363134	5E-11	305582	0.00014126	43.17225033
Thyroid preparations	rs6111715	C	G	0.179159	-0.082008659	0.011882613	5.1E-12	305582	0.000155848	47.63132268
Thyroid preparations	rs2823272	A	T	0.314683	-0.063779944	0.009841599	9.1E-11	305582	0.00013742	41.99853002
Thyroid preparations	rs12482947	T	C	0.398927	-0.059588803	0.009418694	2.5E-10	305582	0.000130967	40.02626632
Thyroid preparations	rs5763793	T	G	0.358287	-0.060941514	0.009507687	1.5E-10	305582	0.000134428	41.08410703
Thyroid preparations	rs2858483	C	A	0.425467	0.1004399	0.009228291	1.4E-27	305582	0.000387502	118.4587368
Thyroid preparations	rs767593	T	G	0.224352	-0.060153248	0.010927384	0.000000037	305582	9.91551E-05	30.30280834
Immunosuppressants	rs6679677	A	C	0.102915	0.334267837	0.037253014	2.9E-19	272602	0.000295263	80.51247283
Immunosuppressants	rs2507996	A	G	0.344361	-0.154498047	0.023873066	9.7E-11	272602	0.000153615	41.88190292
Immunosuppressants	rs143236495	A	G	0.0274827	0.381347685	0.069269438	0.000000037	272602	0.000111168	30.30789099
Immunosuppressants	rs687308	T	C	0.191895	0.56109229	0.028824366	2.1E-84	272602	0.001388087	378.9185726
Immunosuppressants	rs9276609	G	C	0.373183	0.1894863	0.023394271	5.5E-16	272602	0.000240604	65.60446601
Anti-inflammatory and antirheumatic products, non-steroids	rs56166763	C	G	0.368625	-0.040853898	0.007162742	0.000000012	164520	0.000197699	32.53143066
Anti-inflammatory and antirheumatic products, non-steroids	rs12522598	G	A	0.288625	0.045219532	0.007621602	0.000000003	164520	0.000213918	35.20094753
Anti-inflammatory and antirheumatic products, non-steroids	rs6891880	G	A	0.42715	-0.038491819	0.007057721	0.000000049	164520	0.000180763	29.74422418
Anti-inflammatory and antirheumatic products, non-steroids	rs2517611	G	A	0.229963	-0.050484305	0.008190532	7.1E-10	164520	0.000230871	37.99122584
Anti-inflammatory and antirheumatic products, non-steroids	rs114212906	T	C	0.0468755	0.092068098	0.016327301	0.000000017	164520	0.000193236	31.79686348
Anti-inflammatory and antirheumatic products, non-steroids	rs3001426	C	T	0.452847	-0.056191782	0.007003514	1E-15	164520	0.000391134	64.37368073
Anti-inflammatory and	rs34862454	C	T	0.329747	-0.040197316	0.00734261	0.000000044	164520	0.000182136	29.97008198

antirheumatic products, non-steroids										
Drugs affecting bone structure and mineralization	rs6684375	T	C	0.175671	-0.118161792	0.020956325	0.000000017	215668	0.000147392	31.79214654
Drugs affecting bone structure and mineralization	rs17130567	G	A	0.254436	-0.109555584	0.018352051	2.4E-09	215668	0.000165212	35.63656286
Drugs affecting bone structure and mineralization	rs9661787	G	C	0.183508	-0.117555128	0.020837268	0.000000017	215668	0.000147554	31.82713787
Drugs affecting bone structure and mineralization	rs442115	T	G	0.438769	0.097873963	0.016079607	1.2E-09	215668	0.00017176	37.04925433
Drugs affecting bone structure and mineralization	rs899040	T	A	0.338096	-0.096350051	0.016881751	0.000000011	215668	0.000151014	32.57353384
Drugs affecting bone structure and mineralization	rs9268500	T	C	0.0508279	0.203065151	0.036352049	0.000000023	215668	0.000144665	31.203908
Drugs affecting bone structure and mineralization	rs3779381	G	A	0.258819	-0.113869923	0.018283648	4.7E-10	215668	0.000179816	38.78718965
Drugs affecting bone structure and mineralization	rs11228240	T	C	0.276044	0.108600224	0.017921129	1.4E-09	215668	0.000170244	36.72203073
Drugs affecting bone structure and mineralization	rs9594738	T	C	0.48902	0.125975042	0.015974856	3.1E-15	215668	0.00028826	62.18578403
Drugs affecting bone structure and mineralization	rs7230704	A	G	0.221148	-0.119432003	0.019365489	6.9E-10	215668	0.000176328	38.03474036
Opioids	rs2618039	T	A	0.380661	0.061221961	0.011072679	0.000000032	78808	0.000387767	30.57018322

Opioids	rs7428430	T	C	0.482569	-0.065191409	0.010717268	1.2E-09	78808	0.000469287	36.99998357
Opioids	rs12238134	A	G	0.305094	-0.07148008	0.011640261	8.2E-10	78808	0.000478263	37.70800545
Antimigraine preparations	rs61759167	T	C	0.22306	0.151166697	0.023279695	8.4E-11	119844	0.000351713	42.16483553
Antimigraine preparations	rs10908504	C	A	0.347013	0.122427751	0.020322857	1.7E-09	119844	0.000302721	36.28966475
Antimigraine preparations	rs4663983	G	A	0.193803	-0.188978681	0.024389704	9.3E-15	119844	0.000500701	60.03510897
Antimigraine preparations	rs9349379	G	A	0.402462	-0.180234231	0.019654804	4.7E-20	119844	0.000701159	84.08719646
Antimigraine preparations	rs7770889	T	C	0.347302	0.150991253	0.020326134	1.1E-13	119844	0.000460233	55.18064261
Antimigraine preparations	rs74809038	G	A	0.154414	0.158410427	0.026817479	3.5E-09	119844	0.000291064	34.89188055
Antimigraine preparations	rs12532479	C	T	0.105246	0.177533392	0.03144593	0.000000016	119844	0.000265888	31.87307556
Antimigraine preparations	rs16914980	C	T	0.259439	-0.122270056	0.022149161	0.000000034	119844	0.000254214	30.47322432
Antimigraine preparations	rs7852872	G	C	0.365622	-0.142738093	0.02005844	1.1E-12	119844	0.000422363	50.63819836
Antimigraine preparations	rs827405	T	C	0.20682	0.147334231	0.024346469	1.4E-09	119844	0.000305483	36.62082299
Antimigraine preparations	rs10849061	C	T	0.486742	0.119227203	0.019418262	8.3E-10	119844	0.000314468	37.69838691
Antimigraine preparations	rs11172113	C	T	0.411039	-0.143503813	0.019623697	2.6E-13	119844	0.000446021	53.47588174
Antidepressants	rs10846305	G	A	0.391489	0.048607272	0.008296846	4.7E-09	304162	0.000112829	34.32208113
Glucocorticoids	rs12123821	T	C	0.0470148	0.153392807	0.026356427	5.9E-09	205700	0.000164638	33.87136624
Glucocorticoids	rs2949661	T	C	0.401994	-0.074315371	0.011428852	7.9E-11	205700	0.000205508	42.28125689
Glucocorticoids	rs2287037	T	C	0.395554	0.096040754	0.011432775	4.4E-17	205700	0.000342945	70.56731969
Glucocorticoids	rs34290285	A	G	0.255506	-0.12759555	0.012807354	2.2E-23	205700	0.00048229	99.25399249
Glucocorticoids	rs10154834	C	T	0.494958	-0.067905188	0.011217121	1.4E-09	205700	0.000178128	36.64703913
Glucocorticoids	rs1898671	T	C	0.350684	0.097538431	0.011702287	7.8E-17	205700	0.000337621	69.47136879

Glucocorticoids	rs2395005	A	G	0.496857	0.085835109	0.011163263	1.5E-14	205700	0.000287335	59.12122528
Glucocorticoids	rs1391371	T	A	0.202145	0.204121963	0.013949579	1.7E-48	205700	0.001039851	214.1179304
Glucocorticoids	rs78496075	G	A	0.0558766	-0.166577753	0.02445999	9.7E-12	205700	0.000225418	46.37855581
Glucocorticoids	rs1504215	A	G	0.350757	-0.086968465	0.011709394	1.1E-13	205700	0.000268104	55.1633372
Glucocorticoids	rs4739738	G	A	0.358014	0.078805425	0.011672134	1.5E-11	205700	0.000221555	45.58348364
Glucocorticoids	rs992969	A	G	0.251777	0.139871247	0.012898849	2.1E-27	205700	0.000571311	117.5846865
Glucocorticoids	rs1775553	T	C	0.423019	-0.117927194	0.011302505	1.7E-25	205700	0.00052895	108.8615025
Glucocorticoids	rs7936312	T	G	0.477022	0.098884502	0.011205948	1.1E-18	205700	0.000378408	77.86728716
Glucocorticoids	rs1689510	C	G	0.338025	0.080533166	0.011856495	1.1E-11	205700	0.000224236	46.13522879
Glucocorticoids	rs1963497	A	C	0.182496	-0.097599281	0.014568562	2.1E-11	205700	0.000218138	44.88028758
Glucocorticoids	rs72743461	A	C	0.236679	0.09897992	0.013141574	5E-14	205700	0.000275705	56.72770321
Glucocorticoids	rs35441874	A	T	0.246038	-0.077133744	0.013065188	3.6E-09	205700	0.000169414	34.85405023
Glucocorticoids	rs1011082	C	T	0.483207	0.07408	0.011180029	3.4E-11	205700	0.000213397	43.90478936
Glucocorticoids	rs117710327	A	C	0.0620109	-0.141427443	0.023380492	1.5E-09	205700	0.000177848	36.58944835
Antihistamines for systemic use	rs34290285	A	G	0.25507	-0.083370778	0.014355736	6.3E-09	151636	0.000222371	33.72648094
Antihistamines for systemic use	rs1968514	A	G	0.0571983	-0.165402738	0.02694872	8.4E-10	151636	0.00024837	37.67067219
Antihistamines for systemic use	rs6594499	A	C	0.48439	-0.074990387	0.012596448	2.6E-09	151636	0.000233674	35.44126922
Antihistamines for systemic use	rs28407950	T	C	0.243266	-0.094664482	0.014631073	9.8E-11	151636	0.000275994	41.8616209
Antihistamines for systemic use	rs2095044	T	C	0.257651	0.085321428	0.014331368	2.6E-09	151636	0.000233688	35.44338552
Antihistamines for systemic use	rs11236797	A	C	0.453871	0.093666103	0.012589281	1E-13	151636	0.000364924	55.35506113
Antihistamines for systemic use	rs1059513	C	T	0.107253	-0.117081912	0.020201596	6.8E-09	151636	0.000221467	33.5894221

Table S7. Detailed information on summary data for 4 site-specific cancers in replication MR analysis

Cancer	Consortium	Cases (n)	Controls (n)	Sample size (n)	Source
Breast cancer	FinnGenR9	15680	167189	182869	https://www.finngen.fi/en/access_results
Lung cancer		5842	287137	292979	
Colorectal cancer		6509	287137	293646	
Prostate cancer		13216	119948	133164	

Table S8. Characteristics of the 19 medications used in the NHANES study

	Overall (N=30899)
Drugs for peptic ulcer and GORD	
No	29847 (96.6%)
Yes	1052 (3.4%)
Drugs used in diabetes	
No	29736 (96.2%)
Yes	1163 (3.8%)
Antithrombotic agents	
No	30554 (98.9%)
Yes	345 (1.1%)
Vasodilators used in cardiac diseases	
No	30838 (99.8%)
Yes	61 (0.2%)
Antihypertensives	
No	30728 (99.4%)
Yes	171 (0.6%)
Antihypertensives	
No	29668 (96.0%)
Yes	1231 (4.0%)
Beta blocking agents	
No	29891 (96.7%)
Yes	1008 (3.3%)
Calcium channel blockers	
No	30085 (97.4%)
Yes	814 (2.6%)
Agents acting on the renin-angiotensin system	
No	29046 (94.0%)
Yes	1853 (6.0%)
HMG CoA reductase inhibitors	
No	29477 (95.4%)
Yes	1422 (4.6%)
Thyroid preparations	
No	30228 (97.8%)
Yes	671 (2.2%)
Immunosuppressants	
No	30811 (99.7%)
Yes	88 (0.3%)
Antiinflammatory and antirheumatic products, non-steroids	
No	29741 (96.3%)
Yes	1158 (3.7%)
Drugs affecting bone structure and mineralization	
No	30734 (99.5%)
Yes	165 (0.5%)

Opioids	
No	30228 (97.8%)
Yes	671 (2.2%)
Antimigraine preparations	
No	30792 (99.7%)
Yes	107 (0.3%)
Antidepressants	
No	29846 (96.6%)
Yes	1053 (3.4%)
Glucocorticoids	
No	30673 (99.3%)
Yes	226 (0.7%)
Antihistamines for systemic use	
No	30629 (99.1%)
Yes	270 (0.9%)

Table S9. The results of survey-weighted multivariate logistic regression analysis for medication use and breast cancer in the NHANES study

Medications	No. of non-breast cancer	No. of breast cancer	OR	95% CI	*P*
A02B Drugs for peptic ulcer and GORD					
No (10667)	10346 (97.0%)	321 (3.0%)			
Yes (2767)	2742 (99.1%)	25 (0.9%)	0.32	0.18-0.57	<0.001
A10 Drugs used in diabetes					
No (9991)	9730 (97.4%)	261 (2.6%)			
Yes (2926)	2893 (98.9%)	33 (1.1%)	0.45	0.29-0.70	<0.001
B01A Antithrombotic agents					
No (5273)	5104 (96.8%)	169 (3.2%)			
Yes (1378)	1371 (99.5%)	7 (0.5%)	0.15	0.07-0.32	<0.001
C01D Vasodilators used in cardiac diseases					
No (1429)	1387 (97.1%)	42 (2.9%)			
Yes (360)	356 (98.9%)	4 (1.1%)	0.45	0.16-1.28	0.130
C02 Antihypertensives					
No (2702)	2650 (98.1%)	52 (1.9%)			
Yes (685)	681 (99.4%)	4 (0.6%)	0.18	0.06-0.50	0.001
C03 Diuretics					
No (10632)	10291 (96.8%)	341 (3.2%)			
Yes (3661)	3609 (86.8%)	52 (13.2%)	0.29	0.21-0.41	<0.001
C07 Beta blocking agents					
No (10121)	9796 (96.8%)	325 (3.2%)			
Yes (3267)	3225 (98.7%)	42 (1.3%)	0.29	0.20-0.43	<0.001
C08 Calcium channel blockers					
No (8622)	8360 (97.0%)	262 (3.0%)			

Yes (2381)	2351 (98.7%)	30 (1.3%)	0.31	0.19-0.49	<0.001
C09 Agents acting on the renin-angiotensin system					
No (12093)	11724 (96.9%)	369 (3.1%)			
Yes (5391)	5340 (99.1%)	51 (0.9%)	0.2	0.14-0.30	<0.001
C10AA HMG CoA reductase inhibitors					
No (10925)	10549 (96.6%)	376 (3.4%)			
Yes (4567)	4536 (99.3%)	31 (0.7%)	0.18	0.12-0.28	<0.001
H03A Thyroid preparations					
No (6959)	6672 (95.9%)	287(4.1%)			
Yes (1846)	1782 (96.5%)	64 (3.5%)	0.68	0.48-0.97	0.031
L04 Immunosuppressants					
No (996)	967 (97.1%)	29 (2.9%)			
Yes (249)	246 (98.8%)	3 (1.2%)	0.31	0.06-1.51	0.150
M01A Antiinflammatory and antirheumatic products, non-steroids					
No (7993)	7771 (97.2%)	222 (2.8%)			
Yes (2024)	2012 (99.4%)	12 (0.6%)	0.22	0.10-0.45	<0.001
M05B Drugs affecting bone structure and mineralization					
No (1645)	1526 (92.8%)	119 (7.2%)			
Yes (419)	410 (97.9%)	9 (2.1%)	0.23	0.09-0.55	0.001
N02A Opioids					
No (4707)	4598 (97.7%)	109 (2.3%)			
Yes (1183)	1173 (99.2%)	10 (0.8%)	0.35	0.19-0.64	<0.001
N02C Antimigraine preparations					

No (1251)	1218 (97.4%)	33 (2.6%)			
Yes (313)	310 (99.0%)	3 (1.0%)	0.43	0.08-2.26	0.300
N06A Antidepressants					
No (9743)	9444 (96.9%)	299 (3.1%)			
Yes (2520)	2489 (98.8%)	31 (1.2%)	0.43	0.26-0.71	0.001
R03BA Glucocorticoids					
No (2679)	2606 (97.3%)	73 (2.7%)			
Yes (671)	664 (99.0%)	7 (1.0%)	0.43	0.15-1.23	0.110
R06A Antihistamines for systemic use					
No (2576)	2490 (96.7%)	86 (3.3%)			
Yes (644)	639 (99.2%)	5 (0.8%)	0.22	0.08-0.64	0.006

Table S10. The results of survey-weighted multivariate logistic regression analysis for medication use and lung cancer in the NHANES study

Medications	No. of non-lung cancer	No. of lung cancer	OR	95% CI	*P*
A02B Drugs for peptic ulcer and GORD					
No (10779)	10730 (99.5%)	49 (0.5%)			
Yes (2817)	2807 (99.6%)	10 (0.4%)	0.68	0.28-1.66	0.400
A10 Drugs used in diabetes					
No (10107)	10061 (99.5%)	46 (0.5%)			
Yes (2965)	2957 (99.7%)	8 (0.3%)	0.62	0.24-1.58	0.300
B01A Antithrombotic agents					
No (5356)	5318 (99.3%)	38 (0.7%)			
Yes (1407)	1402 (99.6%)	5 (0.4%)	0.46	0.15-1.36	0.200
C01D Vasodilators used in cardiac diseases					
No (1467)	1455 (99.2%)	12 (0.8%)			
Yes (369)	366 (99.2%)	3 (0.8%)	0.58	0.15-2.34	0.400
C02 Antihypertensives					
No (2739)	2722 (99.4%)	17 (0.6%)			
Yes (694)	689 (99.3%)	5 (0.7%)	0.7	0.20-2.50	0.600
C03 Diuretics					
No (10632)	10578 (99.5%)	54 (0.5%)			
Yes (3733)	3724 (99.8%)	9 (0.2%)	0.22	0.10-0.47	<0.001
C07 Beta blocking agents					
No (10138)	10090 (99.5%)	48 (0.5%)			
Yes (3333)	3318 (99.5%)	15 (0.5%)	0.67	0.32-1.39	0.300
C08 Calcium channel blockers					
No (8663)	8619 (99.5%)	44 (0.5%)			

Yes (2418)	2407 (99.5%)	11 (0.5%)	0.61	0.26-1.41	0.200
C09 Agents acting on the renin-angiotensin system					
No (12039)	11987 (99.6%)	52 (0.4%)			
Yes (5473)	5457 (99.7%)	16 (0.3%)	0.41	0.22-0.75	0.005
C10AA HMG CoA reductase inhibitors					
No (10851)	10802 (96.5%)	49 (0.5%)			
Yes (4650)	4633 (99.6%)	17 (0.4%)	0.31	0.15-0.67	0.003
H03A Thyroid preparations					
No (6985)	6961 (99.7%)	24 (0.3%)			
Yes (1875)	1866 (99.5%)	9 (0.5%)	0.66	0.27-1.65	0.400
L04 Immunosuppressants					
No (1014)	1008 (99.4%)	6 (0.6%)			
Yes (254)	253 (99.6%)	1 (0.4%)	0.85	0.06-11.6	>0.900
M01A Antiinflammatory and antirheumatic products, non-steroids					
No (8130)	8093 (99.5%)	37 (0.5%)			
Yes (2053)	2048 (99.8%)	5 (0.2%)	0.65	0.23-1.86	0.400
M05B Drugs affecting bone structure and mineralization					
No (1745)	1734 (99.4%)	11 (0.6%)			
Yes (452)	451 (99.8%)	1 (0.2%)	0.22	0.02-1.91	0.200
N02A Opioids					
No (4789)	4771 (99.6%)	18 (0.4%)			
Yes (1204)	1201(99.8%)	3 (0.2%)	0.47	0.09-2.45	0.400
N02C Antimigraine preparations					

No (1270)	1267 (99.8%)	3 (0.2%)			
Yes (318)	315 (99.1%)	3 (0.9%)	2.09	0.17-25.2	0.600
N06A Antidepressants					
No (9870)	9834 (99.6%)	36 (0.4%)			
Yes (2583)	2575 (99.7%)	8 (0.3%)	1.09	0.41-2.92	0.900
R03BA Glucocorticoids					
No (2733)	2722 (99.6%)	11 (0.4%)			
Yes (686)	680 (99.1%)	6 (0.9%)	1.2	0.35-4.09	0.800
R06A Antihistamines for systemic use					
No (2596)	2588 (99.7%)	8 (0.3%)			
Yes (649)	648 (99.8%)	1 (0.2%)	0.22	0.02-2.31	0.200

Table S11. The results of survey-weighted multivariate logistic regression analysis for medication use and colorectal cancer in the NHANES study

Medications	No. of non-colorectal cancer	No. of colorectal cancer	OR	95% CI	*P*
A02B Drugs for peptic ulcer and GORD					
No (10739)	10580 (98.5%)	159 (1.5%)			
Yes (2796)	2778 (99.4%)	18 (0.6%)	0.48	0.29-0.79	0.005
A10 Drugs used in diabetes					
No (10080)	9941 (98.6%)	139 (1.4%)			
Yes (2942)	2923 (99.4%)	19 (0.6%)	0.56	0.28-1.13	0.1
B01A Antithrombotic agents					
No (5296)	5177 (97.8%)	119 (2.2%)			
Yes (1377)	1363 (99.0%)	14 (1.0%)	0.47	0.22-1.00	0.051
C01D Vasodilators used in cardiac diseases					
No (1455)	1416 (97.3%)	39 (2.7%)			
Yes (366)	363 (99.2%)	3 (0.8%)	0.25	0.07-0.88	0.032
C02 Antihypertensives					
No (2720)	2666 (98.0%)	54 (2.0%)			
Yes (689)	682 (99.0%)	7 (1.0%)	0.51	0.20-1.26	0.14
C03 Diuretics					
No (10628)	10463 (98.4%)	165 (1.6%)			
Yes (3698)	3669 (99.2%)	29 (0.8%)	0.38	0.26-0.57	<0.001
C07 Beta blocking agents					
No (10152)	9988 (98.4%)	164 (1.6%)			
Yes (3299)	3272 (99.2%)	27 (0.8%)	0.42	0.24-0.74	0.003
C08 Calcium channel blockers					
No (86630)	8504 (98.2%)	156 (1.8%)			

Yes (2404)	2390 (99.4%)	14 (0.6%)	0.24	0.10-0.59	0.002
C09 Agents acting on the renin-angiotensin system					
No (12080)	11903 (98.5%)	177 (1.5%)			
Yes (5435)	5405 (99.4%)	30 (0.6%)	0.23	0.14-0.39	<0.001
C10AA HMG CoA reductase inhibitors					
No (10895)	10711 (98.3%)	184 (1.7%)			
Yes (4599)	4575 (99.5%)	24 (0.5%)	0.22	0.11-0.46	<0.001
H03A Thyroid preparations					
No (6975)	6878 (98.6%)	97 (1.4%)			
Yes (1866)	1584 (99.4%)	12 (0.6%)	0.35	0.16-0.78	0.01
L04 Immunosuppressants					
No (1013)	997 (98.4%)	16 (1.6%)			
Yes (254)	253 (99.6%)	1 (0.4%)	0.19	0.03-1.37	0.1
M01A Antiinflammatory and antirheumatic products, non-steroids					
No (8105)	7983 (98.5%)	122 (1.5%)			
Yes (2045)	2032 (99.4%)	13 (0.6%)	0.58	0.30-1.13	0.11
M05B Drugs affecting bone structure and mineralization					
No (1710)	1675 (98.0%)	35 (2.0%)			
Yes (447)	444 (99.3%)	3 (0.7%)	0.34	0.10-1.18	0.088
N02A Opioids					
No (4783)	4714 (98.6%)	69 (1.4%)			
Yes (1200)	1195(99.6%)	5 (0.4%)	0.44	0.16-1.22	0.11
N02C Antimigraine preparations					

No (1263)	1250 (99.0%)	13 (1.0%)			
Yes (316)	312 (98.7%)	4 (1.3%)	1.7	0.49-5.92	0.4
N06A Antidepressants					
No (9806)	9681 (98.7%)	125 (1.3%)			
Yes (2563)	2550 (99.5%)	13 (0.5%)	0.43	0.27-0.69	<0.001
R03BA Glucocorticoids					
No (2683)	2651 (98.8%)	32 (1.2%)			
Yes (674)	671 (99.6%)	3 (0.4%)	0.3	0.06-1.44	0.13
R06A Antihistamines for systemic use					
No (2586)	2560 (99.0%)	26 (1.0%)			
Yes (647)	643 (99.4%)	4 (0.6%)	0.55	0.16-1.92	0.3

Table S12. The results of survey-weighted multivariate logistic regression analysis for medication use and prostate cancer in the NHANES study

Medications	No. of non-prostate cancer	No. of prostate cancer	OR	95% CI	*P*
A02B Drugs for peptic ulcer and GORD					
No (4954)	4653 (93.9%)	301 (6.1%)			
Yes (1286)	1241 (96.5%)	45 (3.5%)	0.55	0.34-0.88	0.013
A10 Drugs used in diabetes					
No (5102)	4741 (92.9%)	361 (7.1%)			
Yes (1457)	1425 (97.8%)	32 (2.2%)	0.39	0.23-0.67	<0.001
B01A Antithrombotic agents					
No (3020)	2744 (90.9%)	276 (1.9%)			
Yes (817)	792 (96.9%)	25 (3.1%)	0.29	0.17-0.51	<0.001
C01D Vasodilators used in cardiac diseases					
No (781)	705 (90.0%)	78 (10.0%)			
Yes (199)	193 (97.0%)	6 (3.0%)	0.17	0.07-0.44	<0.001
C02 Antihypertensives					
No (1836)	1655 (90.1%)	181 (9.9%)			
Yes (470)	454 (96.6%)	16 (3.4%)	0.28	0.11-0.70	0.007
C03 Diuretics					
No (4820)	4466 (92.7%)	354 (7.3%)			
Yes (1509)	1450 (96.1%)	59 (3.9%)	0.31	0.22-0.43	<0.001
C07 Beta blocking agents					
No (4837)	4464 (92.3%)	373 (7.7%)			
Yes (1611)	1556 (96.6%)	55 (3.4%)	0.3	0.20-0.46	<0.001

C08 Calcium channel blockers					
No (4224)	3894 (92.2%)	330 (7.8%)			
Yes (1142)	1096 (96.0%)	46 (4.0%)	0.33	0.22-0.49	<0.001
C09 Agents acting on the renin-angiotensin system					
No (6177)	5808 (94.0%)	369 (6.0%)			
Yes (2622)	2532 (96.6%)	90 (3.4%)	0.42	0.27-0.63	<0.001
C10AA HMG CoA reductase inhibitors					
No (5616)	5244 (93.4%)	372 (6.6%)			
Yes (2418)	2332 (96.4%)	86 (3.6%)	0.35	0.24-0.51	<0.001
H03A Thyroid preparations					
No (1523)	1413 (92.8%)	110 (7.2%)			
Yes (383)	367 (95.8%)	16 (4.2%)	0.66	0.31-1.43	0.3
L04 Immunosuppressants					
No (344)	323 (93.9%)	21 (6.1%)			
Yes (86)	85 (98.8%)	1 (1.2%)	0.21	0.02-1.96	0.2
M01A Antiinflammatory and antirheumatic products, non-steroids					
No (3480)	3264 (93.8%)	216 (6.2%)			
Yes (875)	861 (98.4%)	14 (1.6%)	0.2	0.10-0.41	<0.001
M05B Drugs affecting bone structure and mineralization					
No (166)	148 (89.2%)	18 (10.8%)			
Yes (43)	41 (95.3%)	2 (4.7%)	0.26	0.04-1.64	0.15
N02A Opioids					

No (2087)	1985 (95.1%)	102 (4.9%)			
Yes (534)	528 (98.9%)	6 (1.1%)	0.28	0.09-0.85	0.026
N02C Antimigraine preparations					
No (420)	391 (93.1%)	29 (6.9%)			
Yes (105)	102 (97.1%)	3 (2.9%)	0.19	0.05-0.80	0.025
N06A Antidepressants					
No (3321)	3146 (94.7%)	175 (5.3%)			
Yes (832)	818 (98.3%)	14 (1.7%)	0.38	0.17-0.86	0.021
R03BA Glucocorticoids					
No (1124)	1050 (93.4%)	74 (6.6%)			
Yes (281)	275 (97.9%)	6 (2.1%)	0.21	0.07-0.59	0.004
R06A Antihistamines for systemic use					
No (836)	792 (94.7%)	44 (5.3%)			
Yes (209)	204 (97.6%)	5 (2.4%)	0.57	0.16-2.04	0.4

Table S13. Outliers identified by IVW Radial MR in primary MR analysis

Exposure	Outcome	SNP	Q_statistic	*P*
Antithrombotic agents	Breast cancer	rs12740374	5.679416	0.0172
Antithrombotic agents	Breast cancer	rs1831733	5.748007	0.0165
Antithrombotic agents	Breast cancer	rs56214516	19.882151	0.0000
Antithrombotic agents	Breast cancer	rs74617384	6.72557	0.0095
Beta blocking agents	Breast cancer	rs11649807	12.014669	0.0005
Beta blocking agents	Breast cancer	rs1543927	15.672749	0.0001
Beta blocking agents	Breast cancer	rs2493136	4.090539	0.0431
Beta blocking agents	Breast cancer	rs2891168	16.071335	0.0001
Beta blocking agents	Breast cancer	rs62043959	3.989559	0.0458
Beta blocking agents	Breast cancer	rs7183988	19.044624	0.00001
Beta blocking agents	Breast cancer	rs7652333	9.225746	0.0024
Beta blocking agents	Breast cancer	rs7820612	6.823677	0.0090
Beta blocking agents	Breast cancer	rs9286351	6.568583	0.0104
Agents acting on the renin-angiotensin system	Breast cancer	rs10217586	9.195802	0.0024
Agents acting on the renin-angiotensin system	Breast cancer	rs11072508	20.390465	0.0000
Agents acting on the renin-angiotensin system	Breast cancer	rs11079428	5.285206	0.0215
Agents acting on the renin-angiotensin system	Breast cancer	rs11743404	4.97771	0.0257
Agents acting on the renin-angiotensin system	Breast cancer	rs12366015	9.508989	0.0020
Agents acting on the renin-angiotensin system	Breast cancer	rs12459970	13.928964	0.0002
Agents acting on the renin-angiotensin system	Breast cancer	rs13280592	4.092043	0.0431
Agents acting on the renin-angiotensin system	Breast cancer	rs17637472	6.405426	0.0114
Agents acting on the renin-angiotensin system	Breast cancer	rs1894400	14.312606	0.0002
Agents acting on the renin-angiotensin system	Breast cancer	rs2236295	18.712786	0.00002

Agents acting on the renin-angiotensin system	Breast cancer	rs2252867	5.138808	0.0234
Agents acting on the renin-angiotensin system	Breast cancer	rs2306363	5.244895	0.0220
Agents acting on the renin-angiotensin system	Breast cancer	rs2478543	4.487069	0.0342
Agents acting on the renin-angiotensin system	Breast cancer	rs2643826	30.314888	0.00000004
Agents acting on the renin-angiotensin system	Breast cancer	rs2856653	17.37038	0.00003
Agents acting on the renin-angiotensin system	Breast cancer	rs2984644	5.957733	0.0147
Agents acting on the renin-angiotensin system	Breast cancer	rs300934	6.774351	0.0092
Agents acting on the renin-angiotensin system	Breast cancer	rs3099849	4.225895	0.0398
Agents acting on the renin-angiotensin system	Breast cancer	rs34748838	5.537092	0.0186
Agents acting on the renin-angiotensin system	Breast cancer	rs3803266	14.203754	0.0002
Agents acting on the renin-angiotensin system	Breast cancer	rs415895	10.137819	0.0015
Agents acting on the renin-angiotensin system	Breast cancer	rs4459340	17.286035	0.00003
Agents acting on the renin-angiotensin system	Breast cancer	rs516246	3.998173	0.0455
Agents acting on the renin-angiotensin system	Breast cancer	rs563132	5.724022	0.0167
Agents acting on the renin-angiotensin system	Breast cancer	rs6068191	6.480568	0.0109
Agents acting on the renin-angiotensin system	Breast cancer	rs67529638	4.810997	0.0283
Agents acting on the renin-angiotensin system	Breast cancer	rs6961048	4.823036	0.0281
Agents acting on the renin-angiotensin system	Breast cancer	rs71647010	5.708876	0.0169
Agents acting on the renin-angiotensin system	Breast cancer	rs74439044	6.506459	0.0107
Agents acting on the renin-angiotensin system	Breast cancer	rs7460226	4.24185	0.0394
Agents acting on the renin-angiotensin system	Breast cancer	rs77463690	4.393686	0.0361
Agents acting on the renin-angiotensin system	Breast cancer	rs9506725	6.097131	0.0135
Agents acting on the renin-angiotensin system	Breast cancer	rs9683944	7.659219	0.0056
Agents acting on the renin-angiotensin system	Breast cancer	rs989501	6.668279	0.0098
Thyroid preparations	Breast cancer	rs10098103	5.349902	0.0207
Thyroid preparations	Breast cancer	rs10751647	5.918526	0.0150

Thyroid preparations	Breast cancer	rs10761620	4.15584	0.0415
Thyroid preparations	Breast cancer	rs12742404	7.768619	0.0053
Thyroid preparations	Breast cancer	rs2111485	6.77218	0.0093
Thyroid preparations	Breast cancer	rs2921053	7.069697	0.0078
Thyroid preparations	Breast cancer	rs3008034	5.400388	0.0201
Thyroid preparations	Breast cancer	rs3784099	11.509911	0.0007
Thyroid preparations	Breast cancer	rs56249713	6.169212	0.0130
Thyroid preparations	Breast cancer	rs6914622	4.486226	0.0342
Thyroid preparations	Breast cancer	rs7005834	8.319247	0.0039
Thyroid preparations	Breast cancer	rs8054578	5.59784	0.0180
Antiinflammatory and antirheumatic products, non-steroids	Breast cancer	rs34862454	7.724285	0.0054
Antiinflammatory and antirheumatic products, non-steroids	Breast cancer	rs6891880	4.079771	0.0434
Antithrombotic agents	Lung cancer	rs12740374	3.888552	0.0486
Antithrombotic agents	Lung cancer	rs964184	4.44166	0.0351
Diuretics	Lung cancer	rs11632414	3.963615	0.0465
Diuretics	Lung cancer	rs277168	5.668914	0.0173
Diuretics	Lung cancer	rs3785837	5.534598	0.0186
Diuretics	Lung cancer	rs6961048	4.3753	0.0365
Beta blocking agents	Lung cancer	rs117733303	4.609571	0.0318
Beta blocking agents	Lung cancer	rs2891168	4.134867	0.0420
Calcium channel blockers	Lung cancer	rs3184504	12.392174	0.0004
Calcium channel blockers	Lung cancer	rs35593046	4.051127	0.0441
Calcium channel blockers	Lung cancer	rs553108	16.24105	0.0001
Calcium channel blockers	Lung cancer	rs7983337	6.772666	0.0093

Agents acting on the renin-angiotensin system	Lung cancer	rs10217586	5.968261	0.0146
Agents acting on the renin-angiotensin system	Lung cancer	rs10931284	4.463169	0.0346
Agents acting on the renin-angiotensin system	Lung cancer	rs11072508	4.584613	0.0323
Agents acting on the renin-angiotensin system	Lung cancer	rs11079428	6.670628	0.0098
Agents acting on the renin-angiotensin system	Lung cancer	rs12459970	4.877807	0.0272
Agents acting on the renin-angiotensin system	Lung cancer	rs13118687	4.23933	0.0395
Agents acting on the renin-angiotensin system	Lung cancer	rs1722883	4.195681	0.0405
Agents acting on the renin-angiotensin system	Lung cancer	rs2655438	4.094772	0.0430
Agents acting on the renin-angiotensin system	Lung cancer	rs458356	4.818146	0.0282
Agents acting on the renin-angiotensin system	Lung cancer	rs6961048	3.971738	0.0463
HMG CoA reductase inhibitors	Lung cancer	rs117733303	4.075371	0.0435
HMG CoA reductase inhibitors	Lung cancer	rs11787365	11.112053	0.0009
HMG CoA reductase inhibitors	Lung cancer	rs1537371	4.091495	0.0431
HMG CoA reductase inhibitors	Lung cancer	rs2737245	5.388191	0.0203
HMG CoA reductase inhibitors	Lung cancer	rs2898476	4.213605	0.0401
HMG CoA reductase inhibitors	Lung cancer	rs2982521	9.485765	0.0021
HMG CoA reductase inhibitors	Lung cancer	rs463599	4.23894	0.0395
HMG CoA reductase inhibitors	Lung cancer	rs4639414	7.606993	0.0058
HMG CoA reductase inhibitors	Lung cancer	rs72837687	8.632317	0.0033
HMG CoA reductase inhibitors	Lung cancer	rs964184	4.091448	0.0431
Thyroid preparations	Lung cancer	rs144309607	7.588281	0.0059
Thyroid preparations	Lung cancer	rs174599	11.417419	0.0007
Thyroid preparations	Lung cancer	rs2473808	5.003292	0.0253
Thyroid preparations	Lung cancer	rs3087243	10.642808	0.0011
Thyroid preparations	Lung cancer	rs3184504	6.684232	0.0097
Thyroid preparations	Lung cancer	rs56249713	4.719893	0.0298

Thyroid preparations	Lung cancer	rs6505765	6.575376	0.0103
Thyroid preparations	Lung cancer	rs7302200	9.242373	0.0024
Thyroid preparations	Lung cancer	rs7441808	4.647415	0.0311
Thyroid preparations	Lung cancer	rs8056260	4.330547	0.0374
Thyroid preparations	Lung cancer	rs970987	5.620016	0.0178
Salicylic acid and derivatives	Lung cancer	rs2523589	5.514969	0.0189
Salicylic acid and derivatives	Lung cancer	rs964184	4.513984	0.0336
Calcium channel blockers	Colorectal cancer	rs10015412	4.556066	0.0328
Calcium channel blockers	Colorectal cancer	rs11556924	8.233928	0.0041
Calcium channel blockers	Colorectal cancer	rs259983	4.48893	0.0341
Calcium channel blockers	Colorectal cancer	rs2977324	3.965166	0.0465
Calcium channel blockers	Colorectal cancer	rs3184504	19.087231	0.00001
Calcium channel blockers	Colorectal cancer	rs72689147	12.420417	0.0004
Calcium channel blockers	Colorectal cancer	rs7983337	4.138475	0.0419
Drugs for peptic ulcer and GORD	Prostate cancer	rs11171710	7.355726	0.0067
Drugs for peptic ulcer and GORD	Prostate cancer	rs1619179	15.097409	0.0001
Agents acting on the renin-angiotensin system	Prostate cancer	rs10254101	4.827188	0.0280
Agents acting on the renin-angiotensin system	Prostate cancer	rs10931284	5.338022	0.0209
Agents acting on the renin-angiotensin system	Prostate cancer	rs11072508	5.882995	0.0153
Agents acting on the renin-angiotensin system	Prostate cancer	rs11752759	10.860675	0.0010
Agents acting on the renin-angiotensin system	Prostate cancer	rs12459970	18.679802	0.0000
Agents acting on the renin-angiotensin system	Prostate cancer	rs1799945	6.650476	0.0099
Agents acting on the renin-angiotensin system	Prostate cancer	rs1894400	6.909693	0.0086
Agents acting on the renin-angiotensin system	Prostate cancer	rs2252867	8.911954	0.0028
Agents acting on the renin-angiotensin system	Prostate cancer	rs2655438	4.010281	0.0452
Agents acting on the renin-angiotensin system	Prostate cancer	rs2760061	7.474304	0.0063

Agents acting on the renin-angiotensin system	Prostate cancer	rs2856653	14.4742	0.0001
Agents acting on the renin-angiotensin system	Prostate cancer	rs415895	4.056279	0.0440
Agents acting on the renin-angiotensin system	Prostate cancer	rs4459340	18.011264	0.00002
Agents acting on the renin-angiotensin system	Prostate cancer	rs55730499	20.351013	0.000006
Agents acting on the renin-angiotensin system	Prostate cancer	rs57139556	4.154762	0.0415
Agents acting on the renin-angiotensin system	Prostate cancer	rs6800730	9.062506	0.0026
Agents acting on the renin-angiotensin system	Prostate cancer	rs7070847	6.746994	0.0094
Agents acting on the renin-angiotensin system	Prostate cancer	rs73563812	3.975534	0.0462
Agents acting on the renin-angiotensin system	Prostate cancer	rs7460226	8.696729	0.0032
Agents acting on the renin-angiotensin system	Prostate cancer	rs7631008	4.530411	0.0333
Agents acting on the renin-angiotensin system	Prostate cancer	rs79744918	5.392119	0.0202
Agents acting on the renin-angiotensin system	Prostate cancer	rs9472136	5.433661	0.0198
Agents acting on the renin-angiotensin system	Prostate cancer	rs9939774	6.743455	0.0094

Table S14. SNPs associated with confounders identified by phenotype scanning in primary MR analysis

Exposure	Outome	SNP	Effect allele	Other allele	Trait	Source	Beta	*P*	Samplesize
Anti-inflammatory and antirheumatic products, non-steroids	Breast cancer	rs56166763	C	G	Maternal smoking around birth	UKBB	-0.007182	8.75E-09	289727
Antithrombotic agents	Lung cancer	rs56214516	A	C	Alcohol intake frequency	UKBB	-0.02706	1.67E-09	336965
Thyroid preparations	Lung cancer	rs215619	A	G	Body mass index	UKBB	-0.01414	1.38E-08	336107
Thyroid preparations	Lung cancer	rs3781627	T	A	Body mass index	UKBB	0.0238	2.42E-19	336107
Thyroid preparations	Lung cancer	rs2921053	G	C	Body mass index	UKBB	-0.01726	9.32E-13	336107
Thyroid preparations	Lung cancer	rs3781627	T	A	Alcohol intake frequency	UKBB	0.02223	1.34E-08	336965
Anti-inflammatory and antirheumatic products, non-steroids	Colorectal cancer	rs56166763	C	G	Maternal smoking around birth	UKBB	-0.007182	8.75E-09	289727
Agents acting on the renin-angiotensin system	Prostate cancer	rs13107325	T	C	Body mass index	UKBB	0.0523	2.66E-30	336107
Agents acting on the renin-angiotensin system	Prostate cancer	rs1861410	T	C	Body mass index	UKBB	-0.02141	1.09E-18	336107
Agents acting on the renin-angiotensin system	Prostate cancer	rs3814424	C	T	Body mass index	UKBB	-0.02949	5.11E-19	336107
Agents acting on the renin-angiotensin system	Prostate cancer	rs55872725	C	T	Body mass index	UKBB	-0.07222	3.37E-191	336107
Agents acting on the	Prostate	rs67529638	C	G	Body mass index	UKBB	0.01763	2.98E-12	336107

renin-angiotensin system	cancer								
Agents acting on the renin-angiotensin system	Prostate cancer	rs13107325	T	C	Alcohol intake frequency	UKBB	0.05053	7.88E-14	336965
Agents acting on the renin-angiotensin system	Prostate cancer	rs516246	T	C	Alcohol intake frequency	UKBB	-0.0215	1.61E-09	336965
Agents acting on the renin-angiotensin system	Prostate cancer	rs55872725	C	T	Alcohol intake frequency	UKBB	-0.02164	2.33E-09	336965
Agents acting on the renin-angiotensin system	Prostate cancer	rs7310615	C	G	Past tobacco smoking	UKBB	-0.02126	2.64E-11	310749
Agents acting on the renin-angiotensin system	Prostate cancer	rs7310615	C	G	Smoking status: previous	UKBB	0.007394	2.79E-10	217605

Table S15. Summary data on eleven significant causal associations between medication use and cancer ultimately used in primary MR analysis

Exposure	Outcome	SNP	Effect_allele .exposure	Other_allele. exposure	Effect_allele .outcome	Other_allele. outcome	Beta.exposure	Beta.outcome	Eaf.exposure	Eaf.outcome
Antithrombotic agents	Breast cancer	rs117733303	G	A	G	A	0.188305588	-0.0523	0.0194872	0.0165
		rs17482753	T	G	T	G	-0.070946572	0.0149	0.10142	0.0997
		rs28601761	G	C	G	C	-0.051481012	0.0067	0.415801	0.4185
		rs4299376	G	T	G	T	0.041627513	-0.0132	0.323265	0.3043
		rs6025	T	C	T	C	0.147872685	-0.0125	0.0233795	0.0292
		rs73015016	A	G	A	G	-0.089790481	0	0.119006	0.1184
		rs7412	T	C	T	C	-0.104794922	9.00E-04	0.0800838	0.0819
		rs964184	G	C	G	C	0.067194752	-0.0182	0.132603	0.1379
Anti-inflammatory and antirheumatic products, non-steroids	Breast cancer	rs114212906	T	C	T	C	0.092068098	0.0107	0.0468755	0.0499
		rs12522598	G	A	G	A	0.045219532	0.0052	0.288625	0.2906
		rs2517611	G	A	G	A	-0.050484305	-0.0229	0.229963	0.1965
Antihistamines for systemic use	Breast cancer	rs11236797	A	C	A	C	0.093666103	0.018	0.453871	0.4413
		rs1968514	A	G	A	G	-0.165402738	-0.0226	0.0571983	0.055
		rs2095044	T	C	T	C	0.085321428	-2.00E-04	0.257651	0.2541
		rs28407950	T	C	T	C	-0.094664482	-0.0041	0.243266	0.2834
		rs34290285	A	G	A	G	-0.083370778	-0.006	0.25507	0.2624
		rs6594499	A	C	A	C	-0.074990387	-0.0096	0.48439	0.5064
Antithrombotic agents	Lung cancer	rs117733303	G	A	G	A	0.188305588	-0.137656	0.0194872	0.019544
		rs17482753	T	G	T	G	-0.070946572	-0.00517034	0.10142	0.103266
		rs1831733	C	T	C	T	0.058895787	-0.0371994	0.480371	0.462047
		rs28601761	G	C	G	C	-0.051481012	0.00745018	0.415801	0.398953
		rs4299376	G	T	G	T	0.041627513	-0.0164709	0.323265	0.315698
		rs532436	A	G	A	G	0.066340996	-0.0150812	0.185935	0.197502
		rs6025	T	C	T	C	0.147872685	-0.102542	0.0233795	0.026479
		rs73015016	A	G	A	G	-0.089790481	0.0209451	0.119006	0.11644
		rs7412	T	C	T	C	-0.104794922	0.0466593	0.0800838	0.0811749
		rs74617384	T	A	T	A	0.114872005	-0.0289943	0.0815418	0.0554476
Antihypertensives	Lung	rs34071855	G	C	G	C	0.110219373	-0.0213258	0.339889	0.355949

	cancer	rs35021474	C	G	C	G	0.103529253	-0.0190697	0.385032	0.3971
		rs4980379	T	C	T	C	0.107983973	-0.0280837	0.365232	0.376523
		rs758374	C	T	C	T	0.11363902	-0.0297378	0.304289	0.315081
HMG CoA reductase inhibitors	Lung cancer	rs1030431	A	G	A	G	0.03366996	0.0139394	0.33226	0.34565
		rs10468017	T	C	T	C	0.037635972	-0.00291825	0.297184	0.291022
		rs10804330	C	T	C	T	-0.041844942	-0.0188852	0.429504	0.435558
		rs11220464	T	C	T	C	0.048182021	-0.0320388	0.130142	0.139289
		rs113911544	G	A	G	A	0.085085202	-0.00316701	0.039372	0.0473256
		rs11641811	C	A	C	A	-0.033493562	-0.0210122	0.476408	0.491382
		rs11653468	A	G	A	G	-0.038664313	-0.022803	0.233873	0.23507
		rs116881820	C	T	C	T	0.202944931	0.0556816	0.025925	0.0235999
		rs1169288	C	A	C	A	0.048448524	0.0224276	0.313809	0.332286
		rs11743303	G	A	G	A	0.041238974	0.0322707	0.208058	0.195193
		rs11864054	G	A	G	A	-0.034885282	-0.0225064	0.385154	0.394943
		rs1260326	T	C	T	C	0.059920058	-0.00372593	0.395882	0.416864
		rs12725767	G	A	G	A	0.035128029	-0.00544178	0.354298	0.363816
		rs12740374	T	G	T	G	-0.156618273	-0.0205181	0.219778	0.226442
		rs12916	C	T	C	T	0.058663467	-0.0172805	0.40104	0.410017
		rs13125101	A	G	A	G	0.03686969	-0.00877236	0.292467	0.297214
		rs1367117	A	G	A	G	0.105726091	0.0139098	0.337599	0.319109
		rs1421085	C	T	C	T	0.044655313	-0.00794447	0.403509	0.417589
		rs148566631	A	G	A	G	0.048858199	0.0317457	0.118597	0.126804
		rs148601586	G	C	G	C	0.160733012	0.162162	0.0122258	0.00337522
		rs1501908	G	C	G	C	-0.040749671	0.0161833	0.364761	0.369523
		rs15285	T	C	T	C	-0.058676165	-0.00108759	0.28486	0.290215
		rs17725246	C	T	C	T	0.043688347	-0.00383535	0.183202	0.202761
		rs1883025	T	C	T	C	-0.042749952	0.0448682	0.254856	0.251467
		rs1997833	C	T	C	T	0.043514858	-0.0300747	0.300865	0.291288
		rs2066905	C	G	C	G	-0.035224747	0.00705208	0.45116	0.476386
		rs2068888	A	G	A	G	-0.035113056	-0.0121586	0.450113	0.469429
		rs221907	A	G	A	G	-0.033361871	0.0208227	0.360751	0.368093
		rs2287997	A	G	A	G	0.064653304	-0.00177257	0.18536	0.195698
		rs2293093	G	C	G	C	0.051159056	0.0285739	0.111646	0.11121

		rs2519093	T	C	T	C	0.083506924	-0.0159912	0.185023	0.194986
		rs2618567	G	T	G	T	0.040058837	0.00307826	0.340873	0.354979
		rs2738447	A	C	A	C	-0.062571332	0.0236062	0.406512	0.430623
		rs2954021	A	G	A	G	0.096635253	-0.00876631	0.495627	0.482326
		rs3135064	T	C	T	C	-0.034444179	0.0114018	0.450203	0.42845
		rs34850939	A	G	A	G	0.032981402	-0.00112363	0.349518	0.336329
		rs35430985	A	C	A	C	0.036945826	-0.01507	0.266787	0.273873
		rs3802932	A	G	A	G	0.08027365	-0.0667468	0.0581295	0.0596317
		rs3918226	T	C	T	C	0.068522545	-0.0641898	0.0779394	0.0821428
		rs4299376	G	T	G	T	0.086690463	-0.0164709	0.324311	0.315698
		rs4518111	A	C	A	C	0.037062173	0.0121815	0.433772	0.412254
		rs4541244	T	C	T	C	-0.034275082	0.0121736	0.421979	0.438272
		rs570530	A	G	A	G	-0.032541516	-0.003465	0.495536	0.486077
		rs577721086	C	T	C	T	0.077321853	0.0204201	0.0483191	0.053924
		rs58542926	T	C	T	C	-0.12420776	-0.0375139	0.074933	0.0745159
		rs602662	G	A	G	A	-0.043165394	-0.0112632	0.462426	0.514796
		rs645040	G	T	G	T	-0.042834024	0.0431965	0.225004	0.21124
		rs6511720	T	G	T	G	-0.198647127	0.0210401	0.117712	0.11361
		rs693668	G	A	G	A	-0.048941477	-0.0111054	0.350319	0.36127
		rs7213086	C	G	C	G	-0.033201609	0.00385655	0.44498	0.447848
		rs72631343	G	C	G	C	-0.04882312	-0.00915175	0.128205	0.141888
		rs7412	T	C	T	C	-0.310311732	0.0466593	0.0792273	0.0811749
		rs74617384	T	A	T	A	0.181372313	-0.0289943	0.0801433	0.0554476
		rs7534572	C	G	C	G	-0.074218207	0.036518	0.352475	0.313808
		rs7639816	T	C	T	C	0.041868539	-0.0232041	0.180878	0.178263
		rs77542162	G	A	G	A	0.151112025	0.0448529	0.0230779	0.0174656
		rs7903146	T	C	T	C	0.070760069	-0.0337181	0.290995	0.285658
		rs79598313	T	C	T	C	0.146636976	-0.103527	0.0236446	0.0266145
		rs799158	T	C	T	C	0.079353562	-0.0550466	0.0431865	0.043363
		rs8126001	T	C	T	C	-0.046777285	0.0254953	0.489093	0.502222
		rs972283	A	G	A	G	-0.035923041	-0.00487687	0.488765	0.471268
		rs998584	A	C	A	C	0.038632303	-0.0329107	0.482799	0.486588
Thyroid preparations	Lung	rs10036386	T	C	T	C	0.064672821	-0.00342586	0.381268	0.381171

cancer	rs10098103	C	T	C	T	-0.060081989	0.0125055	0.242332	0.25601
	rs10172181	C	T	C	T	0.111839025	-0.0228685	0.0535539	0.0511468
	rs10279209	A	G	A	G	0.083503352	0.0365614	0.134841	0.132885
	rs1032129	C	A	C	A	-0.059290909	0.0157956	0.353542	0.358243
	rs10489626	G	C	G	C	0.075578895	-0.0523444	0.163317	0.168134
	rs10748781	C	A	C	A	0.053942885	-0.0152598	0.434051	0.419415
	rs10761620	A	G	A	G	0.072898789	0.022322	0.449981	0.453752
	rs10836367	C	T	C	T	0.083144647	-0.0099059	0.388263	0.402718
	rs11666808	T	C	T	C	-0.07337282	-0.00102853	0.373327	0.417837
	rs11675342	T	C	T	C	0.09530659	-0.016232	0.423396	0.42462
	rs11714050	G	A	G	A	0.090516447	0.0420695	0.126095	0.138837
	rs11889341	T	C	T	C	0.143350669	-0.0402544	0.222787	0.222669
	rs12325861	C	T	C	T	0.0937906	0.0144225	0.166127	0.166081
	rs12482947	T	C	T	C	-0.059588803	-0.000232027	0.398927	0.389427
	rs1257926	A	G	A	G	0.054403924	-0.00105155	0.474691	0.48847
	rs12634152	C	T	C	T	0.142340723	0.00706101	0.45193	0.457372
	rs12697352	A	G	A	G	-0.060907224	0.0226329	0.33964	0.323095
	rs12742404	G	A	G	A	0.087284889	0.0183997	0.121153	0.109559
	rs12980063	G	A	G	A	-0.061521228	0.0326056	0.391542	0.386786
	rs13090803	T	G	T	G	0.094675173	-0.00363259	0.21057	0.197352
	rs13136820	C	T	C	T	0.070655075	-0.0134896	0.319992	0.318637
	rs1320344	A	G	A	G	0.069094347	-0.0161345	0.314107	0.315333
	rs13358767	G	A	G	A	-0.057781751	-0.0180204	0.285135	0.291145
	rs13360007	G	A	G	A	0.077983611	-0.0292826	0.135676	0.136025
	rs13398375	C	T	C	T	-0.062139519	0.0338051	0.289167	0.282956
	rs145268310	C	G	C	G	0.102305218	-0.0138322	0.121225	0.112401
	rs1534430	T	C	T	C	-0.08207014	-0.00754136	0.389139	0.399663
	rs1723022	T	G	T	G	0.059215498	0.00950568	0.370454	0.364151
	rs1734911	C	G	C	G	-0.081313484	0.0324711	0.113645	0.169712
	rs1872691	A	G	A	G	-0.073665624	-0.0052005	0.181059	0.190803
	rs1951459	A	G	A	G	-0.115464516	0.0330691	0.334248	0.344947
	rs1991797	T	G	T	G	-0.067278454	0.0264384	0.328168	0.329964
	rs2094920	C	G	C	G	0.052081451	0.0200644	0.449845	0.465666

	rs2111485	A	G	A	G	-0.068354703	0.0400338	0.391151	0.397889
	rs2234167	A	G	A	G	0.081784837	-0.00138496	0.133222	0.140054
	rs2274780	C	T	C	T	0.053449968	-0.00136894	0.470695	0.465654
	rs2445610	G	A	G	A	-0.069989502	0.00643624	0.36907	0.355252
	rs244690	G	A	G	A	0.112015565	0.0193349	0.123038	0.140457
	rs2476601	A	G	A	G	0.413973244	-0.0629654	0.102931	0.09551
	rs2823272	A	T	A	T	-0.063779944	0.0210274	0.314683	0.30268
	rs2858483	C	A	C	A	0.1004399	-0.0260175	0.425467	0.425096
	rs2969903	T	C	T	C	0.062769596	-0.0117265	0.233721	0.214065
	rs3008034	C	T	C	T	-0.063043782	0.0195477	0.305208	0.302233
	rs34591253	T	C	T	C	0.076426098	-0.00208217	0.135132	0.133543
	rs35776863	A	G	A	G	0.088579869	-0.0226782	0.229451	0.211209
	rs35782497	A	G	A	G	-0.083797495	0.0435833	0.339168	0.341345
	rs366327	T	A	T	A	0.06757675	0.00444212	0.184729	0.188673
	rs3784099	A	G	A	G	-0.064911779	0.00686836	0.28051	0.286896
	rs4366932	T	C	T	C	0.052358316	0.0119948	0.403833	0.391404
	rs4409785	C	T	C	T	0.143064027	-0.0234027	0.172241	0.168654
	rs4688013	A	G	A	G	0.083276851	0.0074333	0.191322	0.191829
	rs479777	C	T	C	T	-0.057627312	0.00763875	0.340767	0.333978
	rs5763793	T	G	T	G	-0.060941514	0.0152225	0.358287	0.360507
	rs5865	C	T	C	T	0.06017709	-0.0302644	0.334138	0.338814
	rs6111715	C	G	C	G	-0.082008659	-0.00100651	0.179159	0.180727
	rs61776678	A	G	A	G	-0.061821667	-0.0180397	0.410504	0.420006
	rs61778692	G	A	G	A	-0.065728844	0.00726951	0.245765	0.228372
	rs61907718	A	G	A	G	0.06928254	0.00282002	0.216956	0.22655
	rs6833591	G	A	G	A	-0.058954707	0.0163014	0.346625	0.326217
	rs6914622	T	G	T	G	0.089143108	-0.00971201	0.321667	0.309277
	rs7005834	T	C	T	C	-0.066507467	0.0279565	0.307655	0.289851
	rs7028486	C	T	C	T	0.06116547	0.000751717	0.301266	0.306242
	rs7088058	T	C	T	C	-0.057947976	-0.0108213	0.412751	0.395995
	rs7090530	C	A	C	A	-0.093977185	0.0219924	0.395908	0.396703
	rs71508903	T	C	T	C	0.139273382	-0.040896	0.190444	0.193226
	rs7240256	C	T	C	T	0.05994282	0.00656341	0.317032	0.302419

		rs7254729	T	C	T	C	0.078747633	0.000872619	0.406483	0.422304
		rs72922282	C	G	C	G	-0.078571094	0.041825	0.129635	0.141012
		rs72977594	A	G	A	G	-0.075491429	0.024797	0.149821	0.149985
		rs73316435	T	C	T	C	0.090641418	-0.0381287	0.179924	0.188305
		rs7333647	C	T	C	T	0.124414188	0.0128836	0.287791	0.282532
		rs744253	A	G	A	G	-0.081497927	0.00930656	0.258633	0.276254
		rs75051580	C	T	C	T	0.062789152	-0.00172348	0.201386	0.206713
		rs7583027	A	C	A	C	-0.059402887	-0.000183017	0.357133	0.360878
		rs7596240	G	A	G	A	0.063452716	-0.0120119	0.270163	0.284244
		rs7655751	T	C	T	C	-0.131974448	0.0103136	0.211042	0.189572
		rs767593	T	G	T	G	-0.060153248	-0.0187955	0.224352	0.214794
		rs7754251	G	C	G	C	-0.120908846	0.0168738	0.418931	0.397176
		rs78458460	T	G	T	G	0.091465314	0.00785308	0.239999	0.223604
		rs7850258	A	G	A	G	-0.195523605	-0.0131703	0.331608	0.331544
		rs7931398	C	T	C	T	-0.062308691	0.0162434	0.259045	0.288954
		rs8043085	T	G	T	G	0.094800679	0.0186902	0.232174	0.235789
		rs8054578	A	G	A	G	0.069831447	0.0303575	0.223812	0.228114
		rs853305	T	C	T	C	0.071577287	0.000175985	0.315924	0.298188
		rs9291444	T	C	T	C	0.07350931	0.0262163	0.471241	0.481565
		rs9391997	A	G	A	G	-0.073869488	0.00563012	0.470259	0.507758
		rs9533100	G	T	G	T	-0.067726902	0.0235437	0.4624	0.470078
Salicylic acid and derivatives	Lung cancer	rs117733303	G	A	G	A	0.184581009	-0.137656	0.0197081	0.019544
		rs1831733	C	T	C	T	0.045792655	-0.0371994	0.483126	0.462047
		rs28601761	G	C	G	C	-0.060665793	0.00745018	0.415104	0.398953
		rs583104	G	T	G	T	-0.078039336	-0.0147766	0.224727	0.233311
		rs635634	T	C	T	C	0.066638239	-0.0133112	0.184501	0.197676
		rs73015016	A	G	A	G	-0.095657302	0.0209451	0.117682	0.11644
		rs7412	T	C	T	C	-0.11077729	0.0466593	0.0784037	0.0811749
		rs74617384	T	A	T	A	0.105044421	-0.0289943	0.0826667	0.0554476
Anti-inflammatory and antirheumatic products, non-steroids	Colorectal cacncer	rs114212906	T	C	T	C	0.092068098	-0.085288023	0.0468755	0.041799371
		rs12522598	G	A	G	A	0.045219532	0.00265551	0.288625	0.257177776
		rs2517611	G	A	G	A	-0.050484305	0.022980292	0.229963	0.18591703
		rs3001426	C	T	C	T	-0.056191782	0.053718095	0.452847	0.399617264

		rs34862454	C	T	C	T	-0.040197316	0.024582275	0.329747	0.43824901
		rs6891880	G	A	G	A	-0.038491819	0.02809799	0.42715	0.490876682
Agents acting on the renin-angiotensin system	Prostate cancer	rs1000010	G	A	G	A	-0.037241893	0.0168	0.351806	0.3656
		rs10155132	A	G	A	G	-0.034312642	0.0178	0.39295	0.3969
		rs10213458	A	G	A	G	0.044955829	-0.0035	0.319084	0.3335
		rs10217586	A	T	A	T	-0.051771935	0.0013	0.467959	0.4772
		rs10817003	A	G	A	G	0.101508797	-0.0329	0.0355255	0.0352
		rs10876531	C	A	C	A	-0.040295171	0.0006	0.286877	0.3122
		rs11079428	T	A	T	A	-0.044247206	0.0127	0.223194	0.2387
		rs11743404	C	T	C	T	-0.045967347	-0.0036	0.360119	0.3709
		rs12366015	A	G	A	G	0.044017771	-0.0077	0.203816	0.2245
		rs12453576	T	C	T	C	0.051418267	0.0091	0.177701	0.1756
		rs12549801	A	G	A	G	-0.040760934	0.0037	0.233986	0.2277
		rs1290786	T	C	T	C	0.038310872	-0.0075	0.429711	0.4371
		rs12985940	C	T	C	T	-0.05392687	-0.0159	0.164948	0.1487
		rs13118687	A	G	A	G	-0.042658706	0.014	0.470423	0.4656
		rs13125101	A	G	A	G	0.117785783	-0.0024	0.2921	0.3022
		rs13205180	T	C	T	C	0.036774742	-0.0002	0.493653	0.4877
		rs1422278	T	G	T	G	0.053985	-0.0032	0.131869	0.1518
		rs146754848	G	T	G	T	0.19072176	0.0233	0.0177044	0.0157
		rs1486195	G	A	G	A	0.035349059	-0.0063	0.38746	0.399
		rs164749	G	T	G	T	-0.048410926	0.0004	0.430761	0.4529
		rs1722883	C	T	C	T	-0.037937707	-0.0025	0.47102	0.4596
		rs1731243	C	T	C	T	0.064796713	0.0011	0.394283	0.3994
		rs1757463	G	A	G	A	0.042849096	0.0087	0.284522	0.2906
		rs17637472	A	G	A	G	0.048654096	-0.0013	0.39989	0.4029
		rs1888693	A	G	A	G	0.040535718	0.0019	0.342875	0.3464
		rs193084249	G	A	G	A	0.135676011	-0.0218	0.0222331	0.0269
		rs2103805	T	A	T	A	-0.042468523	-0.0088	0.354074	0.3581
		rs2105092	A	G	A	G	-0.043208886	-0.0092	0.28919	0.2809
		rs2229742	C	G	C	G	0.056737185	0.0046	0.103481	0.1015
		rs2236295	T	G	T	G	-0.04304643	0.0065	0.402916	0.3959
		rs2247355	T	C	T	C	-0.043164491	0.0203	0.181421	0.1926

	rs2478543	C	T	C	T	0.03772458	0.004	0.403731	0.4191
	rs2643826	T	C	T	C	0.053948991	0.0032	0.453083	0.4345
	rs268263	T	A	T	A	-0.045830458	-0.0023	0.244312	0.2476
	rs28081	A	G	A	G	-0.05214078	-0.005	0.150332	0.1529
	rs28637873	T	C	T	C	-0.062732317	0.007	0.0771092	0.0818
	rs28667801	T	A	T	A	0.03892911	0.0045	0.406024	0.4383
	rs2901761	A	G	A	G	0.043702376	0.0004	0.418024	0.4448
	rs2943660	T	G	T	G	-0.038290346	-0.0102	0.363073	0.3707
	rs2984644	A	G	A	G	0.044590843	-0.0023	0.269037	0.2599
	rs300934	T	G	T	G	0.043723107	-0.0186	0.312643	0.323
	rs3099849	G	A	G	A	-0.048254952	0.0184	0.181898	0.1699
	rs34748838	T	C	T	C	-0.04805376	-0.005	0.489913	0.4689
	rs35005772	T	C	T	C	0.034732723	-0.0092	0.345444	0.3551
	rs35301188	A	G	A	G	0.053642906	-0.0104	0.169551	0.1893
	rs35441	T	C	T	C	-0.066196469	-0.0104	0.380577	0.3895
	rs36071027	T	C	T	C	-0.036414643	0.0136	0.357521	0.3484
	rs3790604	A	C	A	C	0.10309992	-0.0154	0.0727071	0.088
	rs3802932	A	G	A	G	0.08005359	-0.0314	0.0576598	0.0665
	rs3803266	G	C	G	C	0.044118753	-0.0186	0.231717	0.2613
	rs3819536	G	A	G	A	-0.036672422	0.0117	0.295117	0.3026
	rs3918226	T	C	T	C	0.145321004	-0.0108	0.0773003	0.0797
	rs4277405	C	T	C	T	0.056869393	-0.018	0.375555	0.3873
	rs458356	A	G	A	G	-0.050707637	-0.0059	0.236286	0.2604
	rs4691666	C	T	C	T	0.039503828	0.005	0.466701	0.4886
	rs478863	G	A	G	A	0.046254037	0.0016	0.274025	0.2865
	rs4970834	T	C	T	C	-0.056275744	-0.0006	0.185684	0.1881
	rs4981000	C	T	C	T	-0.033718685	0.0024	0.491405	0.5048
	rs56175671	C	A	C	A	0.06036904	-0.0224	0.0917925	0.0901
	rs563132	T	A	T	A	-0.033873682	0.0001	0.390895	0.3772
	rs56329057	T	C	T	C	-0.057476931	-0.001	0.183334	0.1964
	rs57541197	A	G	A	G	-0.096298166	0.0141	0.144319	0.1333
	rs6026739	T	A	T	A	0.103485741	-0.021	0.11701	0.1285
	rs6031431	G	A	G	A	0.038108085	-0.0019	0.461195	0.4659

rs6038468	C	T	C	T	0.038192194	-0.0009	0.291906	0.3074
rs6039216	C	T	C	T	-0.057690418	-0.0065	0.360894	0.3644
rs604723	T	C	T	C	-0.086341431	0.0077	0.271336	0.2795
rs6068191	A	C	A	C	0.034654016	0.0141	0.390648	0.412
rs61772578	G	A	G	A	0.067210919	-0.0013	0.110553	0.121
rs62039768	A	C	A	C	0.059182257	-0.0146	0.0957964	0.0866
rs62043959	C	A	C	A	-0.048664179	0.0195	0.278368	0.2654
rs6584606	A	C	A	C	0.169909072	0.0222	0.0157173	0.017
rs6766859	C	T	C	T	0.040738042	0.0081	0.371212	0.3672
rs6961048	G	C	G	C	0.078311225	0.0184	0.101599	0.1065
rs7149242	T	G	T	G	-0.036728752	0.0059	0.281233	0.2912
rs71647010	A	G	A	G	-0.090900283	-0.0015	0.063423	0.0568
rs7174222	T	C	T	C	0.037073058	0.0092	0.468424	0.4566
rs72683923	C	T	C	T	-0.142640324	0.0362	0.0199679	0.0189
rs72915163	T	C	T	C	0.048985953	-0.0082	0.255546	0.2529
rs7299436	G	T	G	T	-0.062372733	0.0102	0.157439	0.1486
rs73033340	G	A	G	A	-0.095375799	0.0373	0.0317516	0.037
rs73075659	G	A	G	A	-0.042514512	-0.0061	0.343772	0.3172
rs7340705	C	T	C	T	0.041461943	0.0031	0.322424	0.3311
rs7368883	A	G	A	G	0.038084781	0.0039	0.36012	0.3543
rs7412	T	C	T	C	-0.103000636	0.0039	0.0820167	0.0807
rs74439044	C	T	C	T	0.057803078	-0.0191	0.0972493	0.1015
rs751984	C	T	C	T	-0.058711041	0.022	0.112856	0.1258
rs76217384	G	A	G	A	0.043917252	-0.0186	0.231528	0.2229
rs762395	A	G	A	G	-0.038521787	0.007	0.335664	0.336
rs76346476	A	G	A	G	0.090308692	0.0266	0.037629	0.0341
rs7701003	G	A	G	A	-0.055057066	0.0015	0.369354	0.3759
rs7716114	G	A	G	A	-0.042465127	0.0084	0.287064	0.2881
rs77463690	G	A	G	A	-0.069927094	-0.0056	0.0722319	0.0806
rs77924615	A	G	A	G	-0.064609976	-0.0011	0.19261	0.2036
rs7803355	T	C	T	C	0.062577989	-0.0173	0.114798	0.1248
rs78745308	G	C	G	C	0.081932792	0.0109	0.0449878	0.0544
rs7938342	T	A	T	A	-0.067405373	-0.0063	0.415375	0.4085

		rs7940934	G	A	G	A	0.033584574	-0.0174	0.487472	0.4547
		rs79668541	T	C	T	C	-0.093270249	0.0193	0.0759178	0.0883
		rs8118848	A	G	A	G	-0.059723948	-0.0027	0.236442	0.246
		rs880315	C	T	C	T	0.064730866	-0.0067	0.339584	0.3554
		rs9506725	C	T	C	T	-0.043205131	0.0112	0.370015	0.3549
		rs9683944	G	A	G	A	0.043979446	-0.0104	0.204222	0.2044
		rs9844972	C	G	C	G	0.070060845	0.0064	0.0671097	0.0626
		rs989501	G	A	G	A	-0.035428743	-0.0083	0.351584	0.3576
		rs9897348	C	A	C	A	-0.037037357	0.0074	0.395725	0.3848
		rs9907781	C	G	C	G	0.04417426	0.0034	0.242838	0.2334
Vasodilators used in cardiac diseases	Prostate cancer	rs1333047	T	A	T	A	0.165319679	-0.0105	0.492809	0.4848
		rs55730499	T	C	T	C	0.298491837	0.0662	0.0811461	0.0667

Table S15. Summary data on eleven significant causal associations between medication use and cancer ultimately used in primary MR analysis (continued)

SNP	Palindromic	Ambiguous	Chr	Pos_hg19	Se.outcome	Pval.outcome	Proxy.outcome	Target_snp.outcome	Proxy_snp.outcome	Target_a1.outcome
rs117733303	FALSE	FALSE	6	160922870	0.025	0.0365999	NA	NA	NA	NA
rs17482753	FALSE	FALSE	8	19832646	0.0103	0.1465	NA	NA	NA	NA
rs28601761	TRUE	FALSE	8	126500031	0.0066	0.3044	NA	NA	NA	NA
rs4299376	FALSE	FALSE	2	44072576	0.0071	0.0624496	NA	NA	NA	NA
rs6025	FALSE	FALSE	1	169519049	0.0205	0.5418	NA	NA	NA	NA
rs73015016	FALSE	FALSE	19	11191300	0.0101	0.997	NA	NA	NA	NA
rs7412	FALSE	FALSE	19	45412079	0.0115	0.9376	NA	NA	NA	NA
rs964184	TRUE	FALSE	11	116648917	0.009	0.0428795	NA	NA	NA	NA
rs114212906	FALSE	FALSE	6	31391389	0.0143	0.4543	NA	NA	NA	NA
rs12522598	FALSE	FALSE	5	120174466	0.007	0.4553	NA	NA	NA	NA
rs2517611	FALSE	FALSE	6	30169327	0.0081	0.00465104	NA	NA	NA	NA
rs11236797	FALSE	FALSE	11	76299649	0.0064	0.00480596	NA	NA	NA	NA
rs1968514	FALSE	FALSE	3	29575263	0.0137	0.0998298	NA	NA	NA	NA
rs2095044	FALSE	FALSE	9	6192796	0.0071	0.9813	NA	NA	NA	NA
rs28407950	FALSE	FALSE	6	32626348	0.0071	0.566301	NA	NA	NA	NA
rs34290285	FALSE	FALSE	2	242698640	0.0089	0.503499	NA	NA	NA	NA
rs6594499	FALSE	FALSE	5	110470137	0.0063	0.1268	NA	NA	NA	NA
rs117733303	FALSE	FALSE	6	160922870	0.056622	0.0476585	NA	NA	NA	NA

rs17482753	FALSE	FALSE	8	19832646	0.028652	0.861527	NA	NA	NA	NA
rs1831733	FALSE	FALSE	9	22076071	0.017728	0.0471542	NA	NA	NA	NA
rs28601761	TRUE	FALSE	8	126506694	0.019226	0.701698	TRUE	rs28601761	rs112875651	G
rs4299376	FALSE	FALSE	2	44072576	0.019077	0.404815	NA	NA	NA	NA
rs532436	FALSE	FALSE	9	136149830	0.022002	0.509098	NA	NA	NA	NA
rs6025	FALSE	FALSE	1	169519049	0.051348	0.0891477	NA	NA	NA	NA
rs73015016	FALSE	FALSE	19	11191300	0.028279	0.46199	NA	NA	NA	NA
rs7412	FALSE	FALSE	19	45412079	0.03598	0.189326	NA	NA	NA	NA
rs74617384	TRUE	FALSE	6	160997118	0.043984	0.540883	NA	NA	NA	NA
rs34071855	TRUE	FALSE	1	10798489	0.018324	0.263451	NA	NA	NA	NA
rs35021474	TRUE	FALSE	2	26919089	0.017763	0.300902	TRUE	rs35021474	rs1275982	C
rs4980379	FALSE	FALSE	11	1888614	0.018054	0.1376	NA	NA	NA	NA
rs758374	FALSE	FALSE	22	19971552	0.018461	0.124958	NA	NA	NA	NA
rs1030431	FALSE	FALSE	8	59311697	0.019202	0.470079	NA	NA	NA	NA
rs10468017	FALSE	FALSE	15	58678512	0.019789	0.885345	NA	NA	NA	NA
rs10804330	FALSE	FALSE	2	227185749	0.017869	0.308417	NA	NA	NA	NA
rs11220464	FALSE	FALSE	11	126253877	0.024852	0.22364	NA	NA	NA	NA
rs113911544	FALSE	FALSE	1	55706403	0.04096	0.941054	NA	NA	NA	NA
rs11641811	FALSE	FALSE	16	71635836	0.017803	0.256355	NA	NA	NA	NA
rs11653468	FALSE	FALSE	17	47192620	0.021474	0.309893	NA	NA	NA	NA
rs116881820	FALSE	FALSE	19	45397952	0.067439	0.413844	NA	NA	NA	NA
rs1169288	FALSE	FALSE	12	121416650	0.019957	0.259644	NA	NA	NA	NA
rs11743303	FALSE	FALSE	5	55859952	0.023307	0.162065	NA	NA	NA	NA
rs11864054	FALSE	FALSE	16	30846134	0.019478	0.268033	NA	NA	NA	NA
rs1260326	FALSE	FALSE	2	27730940	0.018167	0.840968	NA	NA	NA	NA
rs12725767	FALSE	FALSE	1	6676040	0.018366	0.772328	NA	NA	NA	NA
rs12740374	FALSE	FALSE	1	109817590	0.020808	0.344258	NA	NA	NA	NA
rs12916	FALSE	FALSE	5	74656539	0.017787	0.348318	NA	NA	NA	NA
rs13125101	FALSE	FALSE	4	81174592	0.019276	0.65818	NA	NA	NA	NA
rs1367117	FALSE	FALSE	2	21263900	0.020277	0.49533	NA	NA	NA	NA
rs1421085	FALSE	FALSE	16	53800954	0.018169	0.670152	NA	NA	NA	NA
rs148566631	FALSE	FALSE	2	203447875	0.027977	0.254299	NA	NA	NA	NA
rs148601586	TRUE	FALSE	19	45346666	0.216277	0.477053	NA	NA	NA	NA

rs1501908	TRUE	FALSE	5	156398169	0.018849	0.391642	NA	NA	NA	NA
rs15285	FALSE	FALSE	8	19824667	0.019538	0.956498	NA	NA	NA	NA
rs17725246	FALSE	FALSE	7	44581986	0.023738	0.875093	NA	NA	NA	NA
rs1883025	FALSE	FALSE	9	107664301	0.021706	0.0342287	NA	NA	NA	NA
rs1997833	FALSE	FALSE	20	39690342	0.019099	0.13402	NA	NA	NA	NA
rs2066905	TRUE	TRUE	6	16134247	0.018172	0.701068	TRUE	rs2066905	rs6920309	C
rs2068888	FALSE	FALSE	10	94839642	0.01765	0.503804	NA	NA	NA	NA
rs221907	FALSE	FALSE	14	71596347	0.01906	0.273535	NA	NA	NA	NA
rs2287997	FALSE	FALSE	16	72140553	0.022332	0.938209	NA	NA	NA	NA
rs2293093	TRUE	FALSE	12	52317057	0.028972	0.323842	NA	NA	NA	NA
rs2519093	FALSE	FALSE	9	136141870	0.022182	0.487887	NA	NA	NA	NA
rs2618567	FALSE	FALSE	20	17844492	0.019149	0.874272	NA	NA	NA	NA
rs2738447	FALSE	FALSE	19	11227480	0.018521	0.199898	NA	NA	NA	NA
rs2954021	FALSE	FALSE	8	126482077	0.017572	0.62709	NA	NA	NA	NA
rs3135064	FALSE	FALSE	4	3285389	0.018394	0.538115	NA	NA	NA	NA
rs34850939	FALSE	FALSE	3	47063910	0.02004	0.956209	NA	NA	NA	NA
rs35430985	FALSE	FALSE	3	185488303	0.019653	0.459037	NA	NA	NA	NA
rs3802932	FALSE	FALSE	11	63988045	0.03481	0.084095	NA	NA	NA	NA
rs3918226	FALSE	FALSE	7	150690176	0.033098	0.0792392	NA	NA	NA	NA
rs4299376	FALSE	FALSE	2	44072576	0.019077	0.404815	NA	NA	NA	NA
rs4518111	FALSE	FALSE	3	12377344	0.01868	0.516958	NA	NA	NA	NA
rs4541244	FALSE	FALSE	2	58917535	0.018346	0.509482	NA	NA	NA	NA
rs570530	FALSE	FALSE	1	234866776	0.017976	0.850342	NA	NA	NA	NA
rs577721086	FALSE	FALSE	6	127454893	0.047598	0.676309	TRUE	rs577721086	rs72959041	C
rs58542926	FALSE	FALSE	19	19379549	0.032876	0.288275	NA	NA	NA	NA
rs602662	FALSE	FALSE	19	49206985	0.017654	0.535449	NA	NA	NA	NA
rs645040	FALSE	FALSE	3	135926622	0.022726	0.0521495	NA	NA	NA	NA
rs6511720	FALSE	FALSE	19	11202306	0.028713	0.466906	NA	NA	NA	NA
rs693668	FALSE	FALSE	1	55521109	0.019842	0.587468	NA	NA	NA	NA
rs7213086	TRUE	TRUE	17	45765249	0.017955	0.832239	TRUE	rs7213086	rs6503796	C
rs72631343	TRUE	FALSE	17	67191270	0.027465	0.748116	NA	NA	NA	NA
rs7412	FALSE	FALSE	19	45412079	0.03598	0.189326	NA	NA	NA	NA
rs74617384	TRUE	FALSE	6	160997118	0.043984	0.540883	NA	NA	NA	NA

rs7534572	TRUE	FALSE	1	62999675	0.0199	0.0618714	NA	NA	NA	NA
rs7639816	FALSE	FALSE	3	150073703	0.022636	0.327732	NA	NA	NA	NA
rs77542162	FALSE	FALSE	17	67081278	0.078907	0.582771	NA	NA	NA	NA
rs7903146	FALSE	FALSE	10	114758349	0.019027	0.092933	NA	NA	NA	NA
rs79598313	FALSE	FALSE	1	27284913	0.05001	0.0778825	NA	NA	NA	NA
rs799158	FALSE	FALSE	7	73019074	0.049939	0.322982	NA	NA	NA	NA
rs8126001	FALSE	FALSE	20	62711459	0.018755	0.193572	NA	NA	NA	NA
rs972283	FALSE	FALSE	7	130466854	0.017982	0.79093	NA	NA	NA	NA
rs998584	FALSE	FALSE	6	43757896	0.019693	0.11304	NA	NA	NA	NA
rs10036386	FALSE	FALSE	5	76543603	0.0183	0.854656	NA	NA	NA	NA
rs10098103	FALSE	FALSE	8	23397393	0.020617	0.547292	TRUE	rs10098103	rs4872144	C
rs10172181	FALSE	FALSE	2	55891140	0.038924	0.581296	NA	NA	NA	NA
rs10279209	FALSE	FALSE	7	37424240	0.027539	0.179928	NA	NA	NA	NA
rs1032129	FALSE	FALSE	8	119951900	0.018805	0.402154	NA	NA	NA	NA
rs10489626	TRUE	FALSE	1	67793171	0.023826	0.0420543	NA	NA	NA	NA
rs10748781	FALSE	FALSE	10	101283330	0.01869	0.430081	NA	NA	NA	NA
rs10761620	FALSE	FALSE	10	64057202	0.018492	0.225348	NA	NA	NA	NA
rs10836367	FALSE	FALSE	11	35314455	0.017794	0.588238	NA	NA	NA	NA
rs11666808	FALSE	FALSE	19	18383506	0.01933	0.95842	NA	NA	NA	NA
rs11675342	FALSE	FALSE	2	1407628	0.017649	0.374048	NA	NA	NA	NA
rs11714050	FALSE	FALSE	3	39357107	0.02708	0.1144	NA	NA	NA	NA
rs11889341	FALSE	FALSE	2	191943742	0.020568	0.0657491	NA	NA	NA	NA
rs12325861	FALSE	FALSE	17	40289412	0.023927	0.550334	NA	NA	NA	NA
rs12482947	FALSE	FALSE	21	43852037	0.018412	0.990146	NA	NA	NA	NA
rs1257926	FALSE	FALSE	14	98692996	0.017645	0.95333	NA	NA	NA	NA
rs12634152	FALSE	FALSE	3	188121019	0.0179	0.696341	NA	NA	NA	NA
rs12697352	FALSE	FALSE	5	35837234	0.020144	0.259762	NA	NA	NA	NA
rs12742404	FALSE	FALSE	1	200811765	0.028684	0.525341	NA	NA	NA	NA
rs12980063	FALSE	FALSE	19	50196992	0.018985	0.0814779	NA	NA	NA	NA
rs13090803	FALSE	FALSE	3	105934953	0.022891	0.877191	NA	NA	NA	NA
rs13136820	FALSE	FALSE	4	40307564	0.01958	0.505173	NA	NA	NA	NA
rs1320344	FALSE	FALSE	12	103887477	0.018759	0.406288	NA	NA	NA	NA
rs13358767	FALSE	FALSE	5	72180703	0.021196	0.413809	NA	NA	NA	NA

rs13360007	FALSE	FALSE	5	156577720	0.024682	0.261318	NA	NA	NA	NA
rs13398375	FALSE	FALSE	2	8451701	0.02075	0.0985644	NA	NA	NA	NA
rs145268310	TRUE	FALSE	3	12310773	0.02782	0.6336	NA	NA	NA	NA
rs1534430	FALSE	FALSE	2	12644736	0.01811	0.684863	NA	NA	NA	NA
rs1723022	FALSE	FALSE	1	167405418	0.019375	0.627045	NA	NA	NA	NA
rs1734911	TRUE	FALSE	7	100308811	0.027637	0.237357	NA	NA	NA	NA
rs1872691	FALSE	FALSE	16	50350210	0.022763	0.824193	NA	NA	NA	NA
rs1951459	FALSE	FALSE	6	167402536	0.019368	0.0832434	NA	NA	NA	NA
rs1991797	FALSE	FALSE	5	102622453	0.019513	0.172177	NA	NA	NA	NA
rs2094920	TRUE	TRUE	9	110670409	0.018336	0.272751	TRUE	rs2094920	rs1999349	C
rs2111485	FALSE	FALSE	2	163110536	0.018987	0.0311702	NA	NA	NA	NA
rs2234167	FALSE	FALSE	1	2494330	0.02552	0.957857	NA	NA	NA	NA
rs2274780	FALSE	FALSE	9	127075021	0.017859	0.940047	NA	NA	NA	NA
rs2445610	FALSE	FALSE	8	128197088	0.018663	0.733301	NA	NA	NA	NA
rs244690	FALSE	FALSE	5	133421744	0.025625	0.453165	NA	NA	NA	NA
rs2476601	FALSE	FALSE	1	114377568	0.028117	0.0413276	NA	NA	NA	NA
rs2823272	TRUE	FALSE	21	16798586	0.020389	0.301825	NA	NA	NA	NA
rs2858483	FALSE	FALSE	22	37586672	0.01745	0.153559	NA	NA	NA	NA
rs2969903	FALSE	FALSE	3	108279216	0.021336	0.59499	NA	NA	NA	NA
rs3008034	FALSE	FALSE	6	166043862	0.019787	0.323071	NA	NA	NA	NA
rs34591253	FALSE	FALSE	7	128721877	0.025705	0.937195	NA	NA	NA	NA
rs35776863	FALSE	FALSE	17	7226957	0.02429	0.373313	NA	NA	NA	NA
rs35782497	FALSE	FALSE	4	187001230	0.019895	0.0247702	NA	NA	NA	NA
rs366327	TRUE	FALSE	4	87980030	0.023459	0.852593	NA	NA	NA	NA
rs3784099	FALSE	FALSE	14	68749927	0.019521	0.728227	NA	NA	NA	NA
rs4366932	FALSE	FALSE	2	191592247	0.018623	0.522171	NA	NA	NA	NA
rs4409785	FALSE	FALSE	11	95311422	0.02372	0.346863	NA	NA	NA	NA
rs4688013	FALSE	FALSE	3	119229486	0.022291	0.74245	NA	NA	NA	NA
rs479777	FALSE	FALSE	11	64107477	0.019435	0.697647	NA	NA	NA	NA
rs5763793	FALSE	FALSE	22	30526632	0.018955	0.423484	NA	NA	NA	NA
rs5865	FALSE	FALSE	2	98373006	0.018164	0.112595	NA	NA	NA	NA
rs6111715	TRUE	FALSE	20	17860022	0.025027	0.968747	NA	NA	NA	NA
rs61776678	FALSE	FALSE	1	38377021	0.020546	0.398394	NA	NA	NA	NA

rs61778692	FALSE	FALSE	1	38650752	0.021327	0.736763	NA	NA	NA	NA
rs61907718	FALSE	FALSE	11	128172470	0.021619	0.898121	NA	NA	NA	NA
rs6833591	FALSE	FALSE	4	123546282	0.019321	0.400055	NA	NA	NA	NA
rs6914622	FALSE	FALSE	6	148514301	0.019273	0.624535	NA	NA	NA	NA
rs7005834	FALSE	FALSE	8	134214204	0.020011	0.158807	NA	NA	NA	NA
rs7028486	FALSE	FALSE	9	5387279	0.019624	0.969992	NA	NA	NA	NA
rs7088058	FALSE	FALSE	10	124149352	0.018106	0.561564	NA	NA	NA	NA
rs7090530	FALSE	FALSE	10	6110875	0.01852	0.233146	NA	NA	NA	NA
rs71508903	FALSE	FALSE	10	63779871	0.022348	0.0861232	NA	NA	NA	NA
rs7240256	FALSE	FALSE	18	77174220	0.019455	0.739078	NA	NA	NA	NA
rs7254729	FALSE	FALSE	19	4832362	0.018345	0.962682	NA	NA	NA	NA
rs72922282	TRUE	FALSE	1	65431920	0.026639	0.110532	NA	NA	NA	NA
rs72977594	FALSE	FALSE	19	1187675	0.031293	0.430844	NA	NA	NA	NA
rs73316435	FALSE	FALSE	17	45329477	0.022155	0.105447	NA	NA	NA	NA
rs7333647	FALSE	FALSE	13	24786356	0.021556	0.553382	NA	NA	NA	NA
rs744253	FALSE	FALSE	10	6393009	0.019973	0.644729	NA	NA	NA	NA
rs75051580	FALSE	FALSE	3	5015301	0.022236	0.939655	TRUE	rs75051580	rs7649486	C
rs7583027	FALSE	FALSE	2	62544391	0.018788	0.992373	NA	NA	NA	NA
rs7596240	FALSE	FALSE	2	242444173	0.021078	0.581499	NA	NA	NA	NA
rs7655751	FALSE	FALSE	4	149633421	0.022765	0.654501	NA	NA	NA	NA
rs767593	FALSE	FALSE	22	41779971	0.021228	0.395117	NA	NA	NA	NA
rs7754251	TRUE	FALSE	6	91002494	0.018548	0.363573	TRUE	rs7754251	rs597325	G
rs78458460	FALSE	FALSE	1	108347599	0.021915	0.723796	NA	NA	NA	NA
rs7850258	FALSE	FALSE	9	100549013	0.018573	0.492195	NA	NA	NA	NA
rs7931398	FALSE	FALSE	11	116879676	0.020308	0.425508	NA	NA	NA	NA
rs8043085	FALSE	FALSE	15	38828140	0.022048	0.39796	NA	NA	NA	NA
rs8054578	FALSE	FALSE	16	79316815	0.021711	0.158121	NA	NA	NA	NA
rs853305	FALSE	FALSE	8	133931183	0.019515	0.992925	NA	NA	NA	NA
rs9291444	FALSE	FALSE	4	10713674	0.018506	0.15315	NA	NA	NA	NA
rs9391997	FALSE	FALSE	6	409119	0.017861	0.75547	NA	NA	NA	NA
rs9533100	FALSE	FALSE	13	42996548	0.018522	0.201172	NA	NA	NA	NA
rs117733303	FALSE	FALSE	6	160922870	0.056622	0.0476585	NA	NA	NA	NA
rs1831733	FALSE	FALSE	9	22076071	0.017728	0.0471542	NA	NA	NA	NA

rs28601761	TRUE	FALSE	8	126506694	0.019226	0.701698	TRUE	rs28601761	rs112875651	G
rs583104	FALSE	FALSE	1	109821307	0.020985	0.496989	NA	NA	NA	NA
rs635634	FALSE	FALSE	9	136155000	0.022167	0.562332	NA	NA	NA	NA
rs73015016	FALSE	FALSE	19	11191300	0.028279	0.46199	NA	NA	NA	NA
rs7412	FALSE	FALSE	19	45412079	0.03598	0.189326	NA	NA	NA	NA
rs74617384	TRUE	FALSE	6	160997118	0.043984	0.540883	NA	NA	NA	NA
rs114212906	FALSE	FALSE	6	31391389	0.048535665	0.043235295	NA	NA	NA	NA
rs12522598	FALSE	FALSE	5	120174466	0.889959214	0.01919334	NA	NA	NA	NA
rs2517611	FALSE	FALSE	6	30169327	0.287752967	0.021617037	NA	NA	NA	NA
rs3001426	FALSE	FALSE	12	57509055	0.001908052	0.017305067	NA	NA	NA	NA
rs34862454	FALSE	FALSE	15	75101530	0.163247196	0.017631394	NA	NA	NA	NA
rs6891880	FALSE	FALSE	5	149972794	0.103102239	0.017238068	NA	NA	NA	NA
rs1000010	FALSE	FALSE	3	11604119	0.0085	0.0473304	NA	NA	NA	NA
rs10155132	FALSE	FALSE	4	3369500	0.0083	0.0310499	NA	NA	NA	NA
rs10213458	FALSE	FALSE	4	38366390	0.0087	0.6904	NA	NA	NA	NA
rs10217586	TRUE	TRUE	9	22121349	0.008	0.8696	NA	NA	NA	NA
rs10817003	FALSE	FALSE	9	113148239	0.023	0.1527	NA	NA	NA	NA
rs10876531	FALSE	FALSE	12	54443718	0.0089	0.95	NA	NA	NA	NA
rs11079428	TRUE	FALSE	17	59466701	0.0102	0.2142	NA	NA	NA	NA
rs11743404	FALSE	FALSE	5	128016163	0.0084	0.6658	NA	NA	NA	NA
rs12366015	FALSE	FALSE	11	116990851	0.0098	0.429	NA	NA	NA	NA
rs12453576	FALSE	FALSE	17	3881293	0.0106	0.3928	NA	NA	NA	NA
rs12549801	FALSE	FALSE	8	142441642	0.0096	0.6997	NA	NA	NA	NA
rs1290786	FALSE	FALSE	3	169097381	0.0087	0.3929	NA	NA	NA	NA
rs12985940	FALSE	FALSE	19	7262734	0.0116	0.1721	NA	NA	NA	NA
rs13118687	FALSE	FALSE	4	111406496	0.0084	0.0969304	NA	NA	NA	NA
rs13125101	FALSE	FALSE	4	81174592	0.0095	0.8025	NA	NA	NA	NA
rs13205180	FALSE	FALSE	6	51832494	0.008	0.9822	NA	NA	NA	NA
rs1422278	FALSE	FALSE	5	122467417	0.0113	0.7769	NA	NA	NA	NA
rs146754848	FALSE	FALSE	1	115826476	0.0321	0.4685	NA	NA	NA	NA
rs1486195	FALSE	FALSE	2	108737987	0.0086	0.4682	NA	NA	NA	NA
rs164749	FALSE	FALSE	16	89708224	0.0082	0.9626	NA	NA	NA	NA
rs1722883	FALSE	FALSE	7	134215403	0.0082	0.7596	NA	NA	NA	NA

rs1731243	FALSE	FALSE	2	26930411	0.0089	0.8992	NA	NA	NA	NA
rs1757463	FALSE	FALSE	15	41783814	0.0089	0.3231	NA	NA	NA	NA
rs17637472	FALSE	FALSE	17	47461433	0.0081	0.8761	NA	NA	NA	NA
rs1888693	FALSE	FALSE	10	18440444	0.0083	0.8204	NA	NA	NA	NA
rs193084249	FALSE	FALSE	1	27180088	0.0263	0.4068	TRUE	rs193084249	rs75460349	G
rs2103805	TRUE	FALSE	20	10833718	0.0084	0.2943	NA	NA	NA	NA
rs2105092	FALSE	FALSE	6	134184972	0.0091	0.3125	NA	NA	NA	NA
rs2229742	TRUE	FALSE	21	16339172	0.014	0.740699	NA	NA	NA	NA
rs2236295	FALSE	FALSE	10	64564892	0.0082	0.4284	NA	NA	NA	NA
rs2247355	FALSE	FALSE	8	103876780	0.0102	0.0470598	NA	NA	NA	NA
rs2478543	FALSE	FALSE	1	230844310	0.0082	0.6218	NA	NA	NA	NA
rs2643826	FALSE	FALSE	3	27562988	0.008	0.6897	NA	NA	NA	NA
rs268263	TRUE	FALSE	2	164954174	0.0095	0.8104	NA	NA	NA	NA
rs28081	FALSE	FALSE	5	96092990	0.0114	0.6599	NA	NA	NA	NA
rs28637873	FALSE	FALSE	14	24825677	0.0156	0.6565	NA	NA	NA	NA
rs28667801	TRUE	TRUE	4	26785356	0.009	0.6136	NA	NA	NA	NA
rs2901761	FALSE	FALSE	10	95895127	0.0081	0.9633	NA	NA	NA	NA
rs2943660	FALSE	FALSE	2	227128543	0.0085	0.2274	NA	NA	NA	NA
rs2984644	FALSE	FALSE	5	157482258	0.0093	0.8012	NA	NA	NA	NA
rs300934	FALSE	FALSE	4	144171748	0.0088	0.0340502	NA	NA	NA	NA
rs3099849	FALSE	FALSE	6	31360095	0.0105	0.0798307	TRUE	rs3099849	rs2596517	G
rs34748838	FALSE	FALSE	7	130459242	0.0086	0.559099	NA	NA	NA	NA
rs35005772	FALSE	FALSE	1	6733081	0.0086	0.287	NA	NA	NA	NA
rs35301188	FALSE	FALSE	7	106417963	0.0112	0.3499	NA	NA	NA	NA
rs35441	FALSE	FALSE	12	115553115	0.0082	0.2076	NA	NA	NA	NA
rs36071027	FALSE	FALSE	5	158444274	0.0085	0.108	NA	NA	NA	NA
rs3790604	FALSE	FALSE	1	113046879	0.0148	0.2983	NA	NA	NA	NA
rs3802932	FALSE	FALSE	11	63988045	0.0163	0.0548302	NA	NA	NA	NA
rs3803266	TRUE	FALSE	13	30154349	0.0095	0.0503895	NA	NA	NA	NA
rs3819536	FALSE	FALSE	12	2436998	0.0086	0.1761	NA	NA	NA	NA
rs3918226	FALSE	FALSE	7	150690176	0.0153	0.48	NA	NA	NA	NA
rs4277405	FALSE	FALSE	17	61548948	0.0082	0.0269799	TRUE	rs4277405	rs4459609	C
rs458356	FALSE	FALSE	5	55815892	0.0095	0.535	NA	NA	NA	NA

rs4691666	FALSE	FALSE	4	156398688	0.0085	0.5587	NA	NA	NA	NA
rs478863	FALSE	FALSE	12	69639207	0.0095	0.8696	NA	NA	NA	NA
rs4970834	FALSE	FALSE	1	109814880	0.0106	0.9583	NA	NA	NA	NA
rs4981000	FALSE	FALSE	12	121970346	0.0084	0.7732	NA	NA	NA	NA
rs56175671	FALSE	FALSE	11	26163506	0.0145	0.1208	NA	NA	NA	NA
rs563132	TRUE	FALSE	9	37245476	0.0083	0.9859	NA	NA	NA	NA
rs56329057	FALSE	FALSE	4	156633444	0.0107	0.9279	NA	NA	NA	NA
rs57541197	FALSE	FALSE	10	63515681	0.0119	0.2375	TRUE	rs57541197	rs72831343	A
rs6026739	TRUE	FALSE	20	57739469	0.0119	0.0780692	NA	NA	NA	NA
rs6031431	FALSE	FALSE	20	42795152	0.0084	0.8194	NA	NA	NA	NA
rs6038468	FALSE	FALSE	20	6360394	0.0086	0.9122	NA	NA	NA	NA
rs6039216	FALSE	FALSE	20	8622480	0.0083	0.4322	NA	NA	NA	NA
rs604723	FALSE	FALSE	11	100610546	0.0091	0.395	NA	NA	NA	NA
rs6068191	FALSE	FALSE	20	51068611	0.0084	0.0939702	NA	NA	NA	NA
rs61772578	FALSE	FALSE	1	56939235	0.0124	0.9168	NA	NA	NA	NA
rs62039768	FALSE	FALSE	16	51560761	0.0152	0.3354	NA	NA	NA	NA
rs62043959	FALSE	FALSE	16	81525204	0.0094	0.0388499	NA	NA	NA	NA
rs6584606	FALSE	FALSE	10	106277309	0.0317	0.4831	NA	NA	NA	NA
rs6766859	FALSE	FALSE	3	138055136	0.0088	0.3569	NA	NA	NA	NA
rs6961048	TRUE	FALSE	7	27328187	0.0134	0.1693	NA	NA	NA	NA
rs7149242	FALSE	FALSE	14	101159416	0.0089	0.5077	NA	NA	NA	NA
rs71647010	FALSE	FALSE	1	11934476	0.0176	0.9312	TRUE	rs71647010	rs34293975	A
rs7174222	FALSE	FALSE	15	81018543	0.0081	0.2573	NA	NA	NA	NA
rs72683923	FALSE	FALSE	14	50735947	0.0351	0.3017	NA	NA	NA	NA
rs72915163	FALSE	FALSE	18	48792829	0.0101	0.4201	NA	NA	NA	NA
rs7299436	FALSE	FALSE	12	90113070	0.012	0.3977	NA	NA	NA	NA
rs73033340	FALSE	FALSE	7	1195692	0.0232	0.1075	NA	NA	NA	NA
rs73075659	FALSE	FALSE	12	20373541	0.009	0.5002	NA	NA	NA	NA
rs7340705	FALSE	FALSE	3	53734443	0.0085	0.7186	NA	NA	NA	NA
rs7368883	FALSE	FALSE	2	43378720	0.0084	0.6443	NA	NA	NA	NA
rs7412	FALSE	FALSE	19	45412079	0.0149	0.7954	NA	NA	NA	NA
rs74439044	FALSE	FALSE	17	7781019	0.0137	0.1637	NA	NA	NA	NA
rs751984	FALSE	FALSE	11	61278246	0.0123	0.0748204	NA	NA	NA	NA

rs76217384	FALSE	FALSE	2	144200697	0.0098	0.0578203	NA	NA	NA	NA
rs762395	FALSE	FALSE	21	44769676	0.0084	0.4047	NA	NA	NA	NA
rs76346476	FALSE	FALSE	21	44720384	0.0235	0.2586	NA	NA	NA	NA
rs7701003	FALSE	FALSE	5	157824481	0.0082	0.8533	NA	NA	NA	NA
rs7716114	FALSE	FALSE	5	141779528	0.0092	0.3626	NA	NA	NA	NA
rs77463690	FALSE	FALSE	3	168853335	0.015	0.7072	NA	NA	NA	NA
rs77924615	FALSE	FALSE	16	20392332	0.0106	0.9143	NA	NA	NA	NA
rs7803355	FALSE	FALSE	7	7221775	0.0125	0.1659	NA	NA	NA	NA
rs78745308	TRUE	FALSE	7	6404258	0.0227	0.6317	NA	NA	NA	NA
rs7938342	TRUE	FALSE	11	1887806	0.0082	0.4427	NA	NA	NA	NA
rs7940934	FALSE	FALSE	11	32530001	0.0081	0.0312702	NA	NA	NA	NA
rs79668541	FALSE	FALSE	10	104793904	0.0142	0.1734	NA	NA	NA	NA
rs8118848	FALSE	FALSE	20	62461572	0.0098	0.7865	NA	NA	NA	NA
rs880315	FALSE	FALSE	1	10796866	0.009	0.4571	NA	NA	NA	NA
rs9506725	FALSE	FALSE	13	22314146	0.0084	0.1837	NA	NA	NA	NA
rs9683944	FALSE	FALSE	4	138433162	0.0102	0.3059	NA	NA	NA	NA
rs9844972	TRUE	FALSE	3	150097635	0.0193	0.738501	NA	NA	NA	NA
rs989501	FALSE	FALSE	14	71361414	0.0085	0.3313	NA	NA	NA	NA
rs9897348	FALSE	FALSE	17	1338063	0.0084	0.383	TRUE	rs9897348	rs12936625	C
rs9907781	TRUE	FALSE	17	45079189	0.0097	0.724299	NA	NA	NA	NA
rs1333047	TRUE	TRUE	9	22124504	0.008	0.1876	NA	NA	NA	NA
rs55730499	FALSE	FALSE	6	161005610	0.0157	2.50697E-05	NA	NA	NA	NA

Table S15. Summary data on eleven significant causal associations between medication use and cancer ultimately used in primary MR analysis (continued)

SNP	Target_a2. outcome	Proxy_a1. outcome	Proxy_a2. outcome	Se.exposure	Pval.exposure	Action	Mr_keep	Samplesize.exposure	Samplesize.outcome	R2	F
rs117733303	NA	NA	NA	0.024340005	1.00E-14	2	TRUE	153639	228951	0.00039	59.85
rs17482753	NA	NA	NA	0.011154625	2.00E-10	2	TRUE	153639	228951	0.00026	40.45
rs28601761	NA	NA	NA	0.006971504	1.50E-13	2	TRUE	153639	228951	0.00035	54.53
rs4299376	NA	NA	NA	0.007198171	7.30E-09	2	TRUE	153639	228951	0.00022	33.44
rs6025	NA	NA	NA	0.022256377	3.10E-11	2	TRUE	153639	228951	0.00029	44.14
rs73015016	NA	NA	NA	0.010400082	5.90E-18	2	TRUE	153639	228951	0.00048	74.54
rs7412	NA	NA	NA	0.01239736	2.80E-17	2	TRUE	153639	228951	0.00046	71.45

rs964184	NA	NA	NA	0.009910024	1.20E-11	2	TRUE	153639	228951	0.00030	45.97
rs114212906	NA	NA	NA	0.016327301	1.70E-08	2	TRUE	164520	228951	0.00019	31.80
rs12522598	NA	NA	NA	0.007621602	3.00E-09	2	TRUE	164520	228951	0.00021	35.20
rs2517611	NA	NA	NA	0.008190532	7.10E-10	2	TRUE	164520	228951	0.00023	37.99
rs11236797	NA	NA	NA	0.012589281	1.00E-13	2	TRUE	151636	228951	0.00036	55.36
rs1968514	NA	NA	NA	0.02694872	8.40E-10	2	TRUE	151636	228951	0.00025	37.67
rs2095044	NA	NA	NA	0.014331368	2.60E-09	2	TRUE	151636	228951	0.00023	35.44
rs28407950	NA	NA	NA	0.014631073	9.80E-11	2	TRUE	151636	228951	0.00028	41.86
rs34290285	NA	NA	NA	0.014355736	6.30E-09	2	TRUE	151636	228951	0.00022	33.73
rs6594499	NA	NA	NA	0.012596448	2.60E-09	2	TRUE	151636	228951	0.00023	35.44
rs117733303	NA	NA	NA	0.024340005	1E-14	2	TRUE	153639	27209	0.00039	59.85
rs17482753	NA	NA	NA	0.011154625	2E-10	2	TRUE	153639	27209	0.00026	40.45
rs1831733	NA	NA	NA	0.006789594	4.2E-18	2	TRUE	153639	27209	0.00049	75.24
rs28601761	C	A	G	0.006971504	1.5E-13	2	TRUE	153639	27209	0.00035	54.53
rs4299376	NA	NA	NA	0.007198171	7.3E-09	2	TRUE	153639	27209	0.00022	33.44
rs532436	NA	NA	NA	0.008661608	1.9E-14	2	TRUE	153639	27209	0.00038	58.66
rs6025	NA	NA	NA	0.022256377	3.1E-11	2	TRUE	153639	27209	0.00029	44.14
rs73015016	NA	NA	NA	0.010400082	5.9E-18	2	TRUE	153639	27209	0.00048	74.54
rs7412	NA	NA	NA	0.01239736	2.8E-17	2	TRUE	153639	27209	0.00046	71.45
rs74617384	NA	NA	NA	0.012311457	1.1E-20	2	TRUE	153639	27209	0.00057	87.06
rs34071855	NA	NA	NA	0.019059309	7.3E-09	2	TRUE	152380	27209	0.00022	33.44
rs35021474	G	C	T	0.018403903	0.000000019	2	TRUE	152380	27209	0.00021	31.64
rs4980379	NA	NA	NA	0.018611412	6.6E-09	2	TRUE	152380	27209	0.00022	33.66
rs758374	NA	NA	NA	0.01936612	4.4E-09	2	TRUE	152380	27209	0.00023	34.43
rs1030431	NA	NA	NA	0.005988778	0.000000019	2	TRUE	290385	27209	0.00011	31.61
rs10468017	NA	NA	NA	0.006131474	8.3E-10	2	TRUE	290385	27209	0.00013	37.68
rs10804330	NA	NA	NA	0.005740635	3.1E-13	2	TRUE	290385	27209	0.00018	53.13
rs11220464	NA	NA	NA	0.008326513	7.2E-09	2	TRUE	290385	27209	0.00012	33.48
rs113911544	NA	NA	NA	0.014373915	3.2E-09	2	TRUE	290385	27209	0.00012	35.04
rs11641811	NA	NA	NA	0.005619208	2.5E-09	2	TRUE	290385	27209	0.00012	35.53
rs11653468	NA	NA	NA	0.006608344	4.9E-09	2	TRUE	290385	27209	0.00012	34.23
rs116881820	NA	NA	NA	0.017623061	1.1E-30	2	TRUE	290385	27209	0.00046	132.61
rs1169288	NA	NA	NA	0.006082533	1.6E-15	2	TRUE	290385	27209	0.00022	63.44

rs11743303	NA	NA	NA	0.006954898	0.000000003	2	TRUE	290385	27209	0.00012	35.16
rs11864054	NA	NA	NA	0.005811639	1.9E-09	2	TRUE	290385	27209	0.00012	36.03
rs1260326	NA	NA	NA	0.005712328	9.7E-26	2	TRUE	290385	27209	0.00038	110.03
rs12725767	NA	NA	NA	0.005856082	0.000000002	2	TRUE	290385	27209	0.00012	35.98
rs12740374	NA	NA	NA	0.006742468	2.3E-119	2	TRUE	290385	27209	0.00185	539.57
rs12916	NA	NA	NA	0.005712752	9.7E-25	2	TRUE	290385	27209	0.00036	105.45
rs13125101	NA	NA	NA	0.006180362	2.4E-09	2	TRUE	290385	27209	0.00012	35.59
rs1367117	NA	NA	NA	0.005900949	8.7E-72	2	TRUE	290385	27209	0.00110	321.01
rs1421085	NA	NA	NA	0.005696826	4.6E-15	2	TRUE	290385	27209	0.00021	61.44
rs148566631	NA	NA	NA	0.008647989	0.000000016	2	TRUE	290385	27209	0.00011	31.92
rs148601586	NA	NA	NA	0.025484556	2.8E-10	2	TRUE	290385	27209	0.00014	39.78
rs1501908	NA	NA	NA	0.005813226	2.4E-12	2	TRUE	290385	27209	0.00017	49.14
rs15285	NA	NA	NA	0.006203907	3.1E-21	2	TRUE	290385	27209	0.00031	89.45
rs17725246	NA	NA	NA	0.007218703	1.4E-09	2	TRUE	290385	27209	0.00013	36.63
rs1883025	NA	NA	NA	0.006416125	2.7E-11	2	TRUE	290385	27209	0.00015	44.39
rs1997833	NA	NA	NA	0.006103062	1E-12	2	TRUE	290385	27209	0.00018	50.84
rs2066905	G	T	C	0.005632965	4E-10	2	FALSE	290385	27209	0.00013	39.10
rs2068888	NA	NA	NA	0.005626034	4.3E-10	2	TRUE	290385	27209	0.00013	38.95
rs221907	NA	NA	NA	0.005826718	0.00000001	2	TRUE	290385	27209	0.00011	32.78
rs2287997	NA	NA	NA	0.007201719	2.8E-19	2	TRUE	290385	27209	0.00028	80.59
rs2293093	NA	NA	NA	0.008895074	8.9E-09	2	TRUE	290385	27209	0.00011	33.08
rs2519093	NA	NA	NA	0.007223517	6.5E-31	2	TRUE	290385	27209	0.00046	133.64
rs2618567	NA	NA	NA	0.005911214	1.2E-11	2	TRUE	290385	27209	0.00016	45.92
rs2738447	NA	NA	NA	0.005699048	4.8E-28	2	TRUE	290385	27209	0.00041	120.54
rs2954021	NA	NA	NA	0.005595082	7.7E-67	2	TRUE	290385	27209	0.00103	298.30
rs3135064	NA	NA	NA	0.005658626	1.2E-09	2	TRUE	290385	27209	0.00013	37.05
rs34850939	NA	NA	NA	0.005867987	0.000000019	2	TRUE	290385	27209	0.00011	31.59
rs35430985	NA	NA	NA	0.006372475	6.7E-09	2	TRUE	290385	27209	0.00012	33.61
rs3802932	NA	NA	NA	0.011960626	1.9E-11	2	TRUE	290385	27209	0.00016	45.04
rs3918226	NA	NA	NA	0.01051933	7.3E-11	2	TRUE	290385	27209	0.00015	42.43
rs4299376	NA	NA	NA	0.005971001	9.2E-48	2	TRUE	290385	27209	0.00073	210.79
rs4518111	NA	NA	NA	0.005718625	9.1E-11	2	TRUE	290385	27209	0.00014	42.00
rs4541244	NA	NA	NA	0.005711376	0.000000002	2	TRUE	290385	27209	0.00012	36.01

rs570530	NA	NA	NA	0.005613812	6.8E-09	2	TRUE	290385	27209	0.00012	33.60
rs577721086	T	A	G	0.013085579	3.4E-09	2	TRUE	290385	27209	0.00012	34.92
rs58542926	NA	NA	NA	0.010639856	1.7E-31	2	TRUE	290385	27209	0.00047	136.28
rs602662	NA	NA	NA	0.005623177	1.6E-14	2	TRUE	290385	27209	0.00020	58.93
rs645040	NA	NA	NA	0.006691199	1.5E-10	2	TRUE	290385	27209	0.00014	40.98
rs6511720	NA	NA	NA	0.008684178	8.3E-116	2	TRUE	290385	27209	0.00180	523.24
rs693668	NA	NA	NA	0.005866453	7.3E-17	2	TRUE	290385	27209	0.00024	69.60
rs7213086	G	T	C	0.005647779	4.1E-09	2	FALSE	290385	27209	0.00012	34.56
rs72631343	NA	NA	NA	0.008360322	5.2E-09	2	TRUE	290385	27209	0.00012	34.10
rs7412	NA	NA	NA	0.010360179	4.1E-197	2	TRUE	290385	27209	0.00308	897.14
rs74617384	NA	NA	NA	0.010313884	3.2E-69	2	TRUE	290385	27209	0.00106	309.24
rs7534572	NA	NA	NA	0.005852643	7.5E-37	2	TRUE	290385	27209	0.00055	160.81
rs7639816	NA	NA	NA	0.007355367	0.000000013	2	TRUE	290385	27209	0.00011	32.40
rs77542162	NA	NA	NA	0.018663359	5.6E-16	2	TRUE	290385	27209	0.00023	65.56
rs7903146	NA	NA	NA	0.006160627	1.6E-30	2	TRUE	290385	27209	0.00045	131.92
rs79598313	NA	NA	NA	0.018408443	1.6E-15	2	TRUE	290385	27209	0.00022	63.45
rs799158	NA	NA	NA	0.013771121	8.3E-09	2	TRUE	290385	27209	0.00011	33.20
rs8126001	NA	NA	NA	0.005628309	9.5E-17	2	TRUE	290385	27209	0.00024	69.07
rs972283	NA	NA	NA	0.00559905	1.4E-10	2	TRUE	290385	27209	0.00014	41.16
rs998584	NA	NA	NA	0.005618626	6.2E-12	2	TRUE	290385	27209	0.00016	47.28
rs10036386	NA	NA	NA	0.009382382	5.5E-12	2	TRUE	305582	27209	0.00016	47.51
rs10098103	T	C	T	0.010637018	0.000000016	2	TRUE	305582	27209	0.00010	31.90
rs10172181	NA	NA	NA	0.020185921	0.00000003	2	TRUE	305582	27209	0.00010	30.70
rs10279209	NA	NA	NA	0.013371091	4.2E-10	2	TRUE	305582	27209	0.00013	39.00
rs1032129	NA	NA	NA	0.009567829	5.8E-10	2	TRUE	305582	27209	0.00013	38.40
rs10489626	NA	NA	NA	0.012304875	8.1E-10	2	TRUE	305582	27209	0.00012	37.73
rs10748781	NA	NA	NA	0.009322589	7.2E-09	2	TRUE	305582	27209	0.00011	33.48
rs10761620	NA	NA	NA	0.009220965	2.7E-15	2	TRUE	305582	27209	0.00020	62.50
rs10836367	NA	NA	NA	0.0093819	7.8E-19	2	TRUE	305582	27209	0.00026	78.54
rs11666808	NA	NA	NA	0.009466754	9.1E-15	2	TRUE	305582	27209	0.00020	60.07
rs11675342	NA	NA	NA	0.009231734	5.5E-25	2	TRUE	305582	27209	0.00035	106.58
rs11714050	NA	NA	NA	0.01369856	3.9E-11	2	TRUE	305582	27209	0.00014	43.66
rs11889341	NA	NA	NA	0.010942145	3.3E-39	2	TRUE	305582	27209	0.00056	171.63

rs12325861	NA	NA	NA	0.012218159	1.6E-14	2	TRUE	305582	27209	0.00019	58.93
rs12482947	NA	NA	NA	0.009418694	2.5E-10	2	TRUE	305582	27209	0.00013	40.03
rs1257926	NA	NA	NA	0.009154541	2.8E-09	2	TRUE	305582	27209	0.00012	35.32
rs12634152	NA	NA	NA	0.009196319	4.9E-54	2	TRUE	305582	27209	0.00078	239.57
rs12697352	NA	NA	NA	0.009645008	2.7E-10	2	TRUE	305582	27209	0.00013	39.88
rs12742404	NA	NA	NA	0.013958012	4E-10	2	TRUE	305582	27209	0.00013	39.10
rs12980063	NA	NA	NA	0.009363134	5E-11	2	TRUE	305582	27209	0.00014	43.17
rs13090803	NA	NA	NA	0.011272253	4.5E-17	2	TRUE	305582	27209	0.00023	70.54
rs13136820	NA	NA	NA	0.009885372	8.8E-13	2	TRUE	305582	27209	0.00017	51.09
rs1320344	NA	NA	NA	0.009798616	1.8E-12	2	TRUE	305582	27209	0.00016	49.72
rs13358767	NA	NA	NA	0.01020388	0.000000015	2	TRUE	305582	27209	0.00010	32.07
rs13360007	NA	NA	NA	0.013293483	4.5E-09	2	TRUE	305582	27209	0.00011	34.41
rs13398375	NA	NA	NA	0.010167943	9.9E-10	2	TRUE	305582	27209	0.00012	37.35
rs145268310	NA	NA	NA	0.013963637	2.4E-13	2	TRUE	305582	27209	0.00018	53.68
rs1534430	NA	NA	NA	0.00935578	1.8E-18	2	TRUE	305582	27209	0.00025	76.95
rs1723022	NA	NA	NA	0.009605119	7E-10	2	TRUE	305582	27209	0.00012	38.01
rs1734911	NA	NA	NA	0.01433346	0.000000014	2	TRUE	305582	27209	0.00011	32.18
rs1872691	NA	NA	NA	0.011845738	5E-10	2	TRUE	305582	27209	0.00013	38.67
rs1951459	NA	NA	NA	0.009609606	2.9E-33	2	TRUE	305582	27209	0.00047	144.37
rs1991797	NA	NA	NA	0.009731536	4.7E-12	2	TRUE	305582	27209	0.00016	47.80
rs2094920	G	T	C	0.009170454	0.000000014	2	FALSE	305582	27209	0.00011	32.25
rs2111485	NA	NA	NA	0.009314726	2.2E-13	2	TRUE	305582	27209	0.00018	53.85
rs2234167	NA	NA	NA	0.013360309	9.3E-10	2	TRUE	305582	27209	0.00012	37.47
rs2274780	NA	NA	NA	0.009119769	4.6E-09	2	TRUE	305582	27209	0.00011	34.35
rs2445610	NA	NA	NA	0.009426584	1.1E-13	2	TRUE	305582	27209	0.00018	55.13
rs244690	NA	NA	NA	0.013847775	6E-16	2	TRUE	305582	27209	0.00021	65.43
rs2476601	NA	NA	NA	0.014925497	2.6E-169	2	TRUE	305582	27209	0.00251	769.28
rs2823272	NA	NA	NA	0.009841599	9.1E-11	2	TRUE	305582	27209	0.00014	42.00
rs2858483	NA	NA	NA	0.009228291	1.4E-27	2	TRUE	305582	27209	0.00039	118.46
rs2969903	NA	NA	NA	0.010745875	5.2E-09	2	TRUE	305582	27209	0.00011	34.12
rs3008034	NA	NA	NA	0.00985865	1.6E-10	2	TRUE	305582	27209	0.00013	40.89
rs34591253	NA	NA	NA	0.013319536	9.6E-09	2	TRUE	305582	27209	0.00011	32.92
rs35776863	NA	NA	NA	0.010950075	6E-16	2	TRUE	305582	27209	0.00021	65.44

rs35782497	NA	NA	NA	0.009701988	5.8E-18	2	TRUE	305582	27209	0.00024	74.60
rs366327	NA	NA	NA	0.011895218	0.000000013	2	TRUE	305582	27209	0.00011	32.27
rs3784099	NA	NA	NA	0.01013818	1.5E-10	2	TRUE	305582	27209	0.00013	40.99
rs4366932	NA	NA	NA	0.009310279	0.000000019	2	TRUE	305582	27209	0.00010	31.63
rs4409785	NA	NA	NA	0.012049951	1.6E-32	2	TRUE	305582	27209	0.00046	140.96
rs4688013	NA	NA	NA	0.011549827	5.6E-13	2	TRUE	305582	27209	0.00017	51.99
rs479777	NA	NA	NA	0.009627233	2.2E-09	2	TRUE	305582	27209	0.00012	35.83
rs5763793	NA	NA	NA	0.009507687	1.5E-10	2	TRUE	305582	27209	0.00013	41.08
rs5865	NA	NA	NA	0.009663693	4.8E-10	2	TRUE	305582	27209	0.00013	38.78
rs6111715	NA	NA	NA	0.011882613	5.1E-12	2	TRUE	305582	27209	0.00016	47.63
rs61776678	NA	NA	NA	0.009273056	2.6E-11	2	TRUE	305582	27209	0.00015	44.45
rs61778692	NA	NA	NA	0.010585182	5.3E-10	2	TRUE	305582	27209	0.00013	38.56
rs61907718	NA	NA	NA	0.011124578	4.7E-10	2	TRUE	305582	27209	0.00013	38.79
rs6833591	NA	NA	NA	0.009598676	8.2E-10	2	TRUE	305582	27209	0.00012	37.72
rs6914622	NA	NA	NA	0.009792307	8.8E-20	2	TRUE	305582	27209	0.00027	82.87
rs7005834	NA	NA	NA	0.009841599	1.4E-11	2	TRUE	305582	27209	0.00015	45.67
rs7028486	NA	NA	NA	0.009969597	8.5E-10	2	TRUE	305582	27209	0.00012	37.64
rs7088058	NA	NA	NA	0.009273498	4.1E-10	2	TRUE	305582	27209	0.00013	39.05
rs7090530	NA	NA	NA	0.00931912	6.5E-24	2	TRUE	305582	27209	0.00033	101.69
rs71508903	NA	NA	NA	0.011730452	1.6E-32	2	TRUE	305582	27209	0.00046	140.96
rs7240256	NA	NA	NA	0.009862602	1.2E-09	2	TRUE	305582	27209	0.00012	36.94
rs7254729	NA	NA	NA	0.009273444	2E-17	2	TRUE	305582	27209	0.00024	72.11
rs72922282	NA	NA	NA	0.013553498	6.7E-09	2	TRUE	305582	27209	0.00011	33.61
rs72977594	NA	NA	NA	0.0128825	4.6E-09	2	TRUE	305582	27209	0.00011	34.34
rs73316435	NA	NA	NA	0.011906228	2.7E-14	2	TRUE	305582	27209	0.00019	57.96
rs7333647	NA	NA	NA	0.010090322	6.2E-35	2	TRUE	305582	27209	0.00050	152.03
rs744253	NA	NA	NA	0.010382308	4.2E-15	2	TRUE	305582	27209	0.00020	61.62
rs75051580	T	T	C	0.011461651	0.000000043	2	TRUE	305582	27209	0.00010	30.01
rs7583027	NA	NA	NA	0.009520573	4.4E-10	2	TRUE	305582	27209	0.00013	38.93
rs7596240	NA	NA	NA	0.01022874	5.5E-10	2	TRUE	305582	27209	0.00013	38.48
rs7655751	NA	NA	NA	0.011143826	2.3E-32	2	TRUE	305582	27209	0.00046	140.25
rs767593	NA	NA	NA	0.010927384	0.000000037	2	TRUE	305582	27209	0.00010	30.30
rs7754251	C	A	G	0.009181357	1.3E-39	2	TRUE	305582	27209	0.00057	173.42

rs78458460	NA	NA	NA	0.010684515	1.1E-17	2	TRUE	305582	27209	0.00024	73.28
rs7850258	NA	NA	NA	0.009675413	8.3E-91	2	TRUE	305582	27209	0.00133	408.37
rs7931398	NA	NA	NA	0.010408629	2.1E-09	2	TRUE	305582	27209	0.00012	35.83
rs8043085	NA	NA	NA	0.010843896	2.3E-18	2	TRUE	305582	27209	0.00025	76.43
rs8054578	NA	NA	NA	0.010947476	1.8E-10	2	TRUE	305582	27209	0.00013	40.69
rs853305	NA	NA	NA	0.0097864	2.6E-13	2	TRUE	305582	27209	0.00018	53.49
rs9291444	NA	NA	NA	0.009131422	8.3E-16	2	TRUE	305582	27209	0.00021	64.80
rs9391997	NA	NA	NA	0.009155586	7.1E-16	2	TRUE	305582	27209	0.00021	65.10
rs9533100	NA	NA	NA	0.009159497	1.4E-13	2	TRUE	305582	27209	0.00018	54.67
rs117733303	NA	NA	NA	0.028465654	8.9E-11	2	TRUE	112010	27209	0.00038	42.05
rs1831733	NA	NA	NA	0.007975758	9.4E-09	2	TRUE	112010	27209	0.00029	32.96
rs28601761	C	A	G	0.008191943	1.3E-13	2	TRUE	112010	27209	0.00049	54.84
rs583104	NA	NA	NA	0.009467476	1.7E-16	2	TRUE	112010	27209	0.00061	67.94
rs635634	NA	NA	NA	0.010233878	7.4E-11	2	TRUE	112010	27209	0.00038	42.40
rs73015016	NA	NA	NA	0.012271248	6.4E-15	2	TRUE	112010	27209	0.00054	60.76
rs7412	NA	NA	NA	0.014702809	4.9E-14	2	TRUE	112010	27209	0.00051	56.77
rs74617384	NA	NA	NA	0.014373422	2.7E-13	2	TRUE	112010	27209	0.00048	53.41
rs114212906	NA	NA	NA	0.016327301	1.70E-08	2	TRUE	164520	32072	0.00019	31.80
rs12522598	NA	NA	NA	0.007621602	3.00E-09	2	TRUE	164520	32072	0.00021	35.20
rs2517611	NA	NA	NA	0.008190532	7.10E-10	2	TRUE	164520	32072	0.00023	37.99
rs3001426	NA	NA	NA	0.007003514	1.00E-15	2	TRUE	164520	32072	0.00039	64.37
rs34862454	NA	NA	NA	0.00734261	4.40E-08	2	TRUE	164520	32072	0.00018	29.97
rs6891880	NA	NA	NA	0.007057721	4.90E-08	2	TRUE	164520	32072	0.00018	29.74
rs1000010	NA	NA	NA	0.00638692	5.5E-09	2	TRUE	237530	140254	0.00014	34.00
rs10155132	NA	NA	NA	0.006186808	0.000000029	2	TRUE	237530	140254	0.00013	30.76
rs10213458	NA	NA	NA	0.006448394	3.1E-12	2	TRUE	237530	140254	0.00020	48.60
rs10217586	NA	NA	NA	0.006058458	1.3E-17	2	FALSE	237530	140254	0.00031	73.02
rs10817003	NA	NA	NA	0.016295196	4.7E-10	2	TRUE	237530	140254	0.00016	38.80
rs10876531	NA	NA	NA	0.006720886	0.000000002	2	TRUE	237530	140254	0.00015	35.95
rs11079428	NA	NA	NA	0.007350751	1.8E-09	2	TRUE	237530	140254	0.00015	36.23
rs11743404	NA	NA	NA	0.006289591	2.7E-13	2	TRUE	237530	140254	0.00022	53.41
rs12366015	NA	NA	NA	0.007511509	4.6E-09	2	TRUE	237530	140254	0.00014	34.34
rs12453576	NA	NA	NA	0.007874283	6.6E-11	2	TRUE	237530	140254	0.00018	42.64

rs12549801	NA	NA	NA	0.007146626	0.000000012	2	TRUE	237530	140254	0.00014	32.53
rs1290786	NA	NA	NA	0.006089684	3.2E-10	2	TRUE	237530	140254	0.00017	39.58
rs12985940	NA	NA	NA	0.008127895	3.2E-11	2	TRUE	237530	140254	0.00019	44.02
rs13118687	NA	NA	NA	0.006081145	2.3E-12	2	TRUE	237530	140254	0.00021	49.21
rs13125101	NA	NA	NA	0.006652725	3.8E-70	2	TRUE	237530	140254	0.00132	313.46
rs13205180	NA	NA	NA	0.00603891	1.1E-09	2	TRUE	237530	140254	0.00016	37.08
rs1422278	NA	NA	NA	0.008887087	1.2E-09	2	TRUE	237530	140254	0.00016	36.90
rs146754848	NA	NA	NA	0.022752644	5.2E-17	2	TRUE	237530	140254	0.00030	70.26
rs1486195	NA	NA	NA	0.006230534	0.000000014	2	TRUE	237530	140254	0.00014	32.19
rs164749	NA	NA	NA	0.006097195	2E-15	2	TRUE	237530	140254	0.00027	63.04
rs1722883	NA	NA	NA	0.006060722	3.9E-10	2	TRUE	237530	140254	0.00016	39.18
rs1731243	NA	NA	NA	0.006167826	8.1E-26	2	TRUE	237530	140254	0.00046	110.37
rs1757463	NA	NA	NA	0.006746813	2.1E-10	2	TRUE	237530	140254	0.00017	40.34
rs17637472	NA	NA	NA	0.006254095	7.3E-15	2	TRUE	237530	140254	0.00025	60.52
rs1888693	NA	NA	NA	0.006343554	1.7E-10	2	TRUE	237530	140254	0.00017	40.83
rs193084249	A	C	A	0.020481295	3.5E-11	2	TRUE	237530	140254	0.00018	43.88
rs2103805	NA	NA	NA	0.006343863	2.2E-11	2	TRUE	237530	140254	0.00019	44.81
rs2105092	NA	NA	NA	0.006663373	8.9E-11	2	TRUE	237530	140254	0.00018	42.05
rs2229742	NA	NA	NA	0.009906064	0.00000001	2	TRUE	237530	140254	0.00014	32.80
rs2236295	NA	NA	NA	0.006192724	3.6E-12	2	TRUE	237530	140254	0.00020	48.32
rs2247355	NA	NA	NA	0.007826184	0.000000035	2	TRUE	237530	140254	0.00013	30.42
rs2478543	NA	NA	NA	0.006156097	8.9E-10	2	TRUE	237530	140254	0.00016	37.55
rs2643826	NA	NA	NA	0.006072657	6.5E-19	2	TRUE	237530	140254	0.00033	78.92
rs268263	NA	NA	NA	0.007088238	1E-10	2	TRUE	237530	140254	0.00018	41.80
rs28081	NA	NA	NA	0.008431305	6.2E-10	2	TRUE	237530	140254	0.00016	38.24
rs28637873	NA	NA	NA	0.01133149	0.000000031	2	TRUE	237530	140254	0.00013	30.65
rs28667801	NA	NA	NA	0.006205996	3.5E-10	2	FALSE	237530	140254	0.00017	39.35
rs2901761	NA	NA	NA	0.006106506	8.3E-13	2	TRUE	237530	140254	0.00022	51.22
rs2943660	NA	NA	NA	0.006246173	8.8E-10	2	TRUE	237530	140254	0.00016	37.58
rs2984644	NA	NA	NA	0.00679841	5.4E-11	2	TRUE	237530	140254	0.00018	43.02
rs300934	NA	NA	NA	0.006509148	1.9E-11	2	TRUE	237530	140254	0.00019	45.12
rs3099849	A	G	A	0.007807562	6.4E-10	2	TRUE	237530	140254	0.00016	38.20
rs34748838	NA	NA	NA	0.006040814	1.8E-15	2	TRUE	237530	140254	0.00027	63.28

rs35005772	NA	NA	NA	0.006331053	0.000000041	2	TRUE	237530	140254	0.00013	30.10
rs35301188	NA	NA	NA	0.008088387	3.3E-11	2	TRUE	237530	140254	0.00019	43.98
rs35441	NA	NA	NA	0.006191438	1.1E-26	2	TRUE	237530	140254	0.00048	114.31
rs36071027	NA	NA	NA	0.006290722	7.1E-09	2	TRUE	237530	140254	0.00014	33.51
rs3790604	NA	NA	NA	0.011591173	5.9E-19	2	TRUE	237530	140254	0.00033	79.11
rs3802932	NA	NA	NA	0.01291788	5.7E-10	2	TRUE	237530	140254	0.00016	38.40
rs3803266	NA	NA	NA	0.007159075	7.2E-10	2	TRUE	237530	140254	0.00016	37.98
rs3819536	NA	NA	NA	0.006600922	0.000000028	2	TRUE	237530	140254	0.00013	30.87
rs3918226	NA	NA	NA	0.011365751	2E-37	2	TRUE	237530	140254	0.00069	163.48
rs4277405	T	C	A	0.006227962	6.8E-20	2	TRUE	237530	140254	0.00035	83.38
rs458356	NA	NA	NA	0.007393448	7E-12	2	TRUE	237530	140254	0.00020	47.04
rs4691666	NA	NA	NA	0.006045701	6.4E-11	2	TRUE	237530	140254	0.00018	42.70
rs478863	NA	NA	NA	0.006766207	8.1E-12	2	TRUE	237530	140254	0.00020	46.73
rs4970834	NA	NA	NA	0.007776593	4.6E-13	2	TRUE	237530	140254	0.00022	52.37
rs4981000	NA	NA	NA	0.006057584	0.000000026	2	TRUE	237530	140254	0.00013	30.98
rs56175671	NA	NA	NA	0.01050424	9.1E-09	2	TRUE	237530	140254	0.00014	33.03
rs563132	NA	NA	NA	0.006169883	0.00000004	2	TRUE	237530	140254	0.00013	30.14
rs56329057	NA	NA	NA	0.007785802	1.6E-13	2	TRUE	237530	140254	0.00023	54.50
rs57541197	G	G	T	0.008556207	2.2E-29	2	TRUE	237530	140254	0.00053	126.67
rs6026739	NA	NA	NA	0.009419107	4.4E-28	2	TRUE	237530	140254	0.00051	120.71
rs6031431	NA	NA	NA	0.006128266	5E-10	2	TRUE	237530	140254	0.00016	38.67
rs6038468	NA	NA	NA	0.006636469	8.7E-09	2	TRUE	237530	140254	0.00014	33.12
rs6039216	NA	NA	NA	0.006283675	4.3E-20	2	TRUE	237530	140254	0.00035	84.29
rs604723	NA	NA	NA	0.006842239	1.7E-36	2	TRUE	237530	140254	0.00067	159.23
rs6068191	NA	NA	NA	0.006231769	0.000000027	2	TRUE	237530	140254	0.00013	30.92
rs61772578	NA	NA	NA	0.00963028	3E-12	2	TRUE	237530	140254	0.00021	48.71
rs62039768	NA	NA	NA	0.01031169	9.5E-09	2	TRUE	237530	140254	0.00014	32.94
rs62043959	NA	NA	NA	0.006790334	7.7E-13	2	TRUE	237530	140254	0.00022	51.36
rs6584606	NA	NA	NA	0.02421907	2.3E-12	2	TRUE	237530	140254	0.00021	49.22
rs6766859	NA	NA	NA	0.006306927	1.1E-10	2	TRUE	237530	140254	0.00018	41.72
rs6961048	NA	NA	NA	0.009979678	4.3E-15	2	TRUE	237530	140254	0.00026	61.58
rs7149242	NA	NA	NA	0.006697017	0.000000041	2	TRUE	237530	140254	0.00013	30.08
rs71647010	G	T	G	0.01230957	1.5E-13	2	TRUE	237530	140254	0.00023	54.53

rs7174222	NA	NA	NA	0.006064014	9.7E-10	2	TRUE	237530	140254	0.00016	37.38
rs72683923	NA	NA	NA	0.021553926	3.6E-11	2	TRUE	237530	140254	0.00018	43.80
rs72915163	NA	NA	NA	0.007011692	2.8E-12	2	TRUE	237530	140254	0.00021	48.81
rs7299436	NA	NA	NA	0.008288963	5.3E-14	2	TRUE	237530	140254	0.00024	56.62
rs73033340	NA	NA	NA	0.017169671	0.000000028	2	TRUE	237530	140254	0.00013	30.86
rs73075659	NA	NA	NA	0.006410327	3.3E-11	2	TRUE	237530	140254	0.00019	43.99
rs7340705	NA	NA	NA	0.006501483	1.8E-10	2	TRUE	237530	140254	0.00017	40.67
rs7368883	NA	NA	NA	0.006283983	1.4E-09	2	TRUE	237530	140254	0.00015	36.73
rs7412	NA	NA	NA	0.011019644	9E-21	2	TRUE	237530	140254	0.00037	87.37
rs74439044	NA	NA	NA	0.010142134	0.000000012	2	TRUE	237530	140254	0.00014	32.48
rs751984	NA	NA	NA	0.009556717	8.1E-10	2	TRUE	237530	140254	0.00016	37.74
rs76217384	NA	NA	NA	0.007115555	6.7E-10	2	TRUE	237530	140254	0.00016	38.09
rs762395	NA	NA	NA	0.006441346	2.2E-09	2	TRUE	237530	140254	0.00015	35.76
rs76346476	NA	NA	NA	0.015856853	0.000000012	2	TRUE	237530	140254	0.00014	32.44
rs7701003	NA	NA	NA	0.006235318	1E-18	2	TRUE	237530	140254	0.00033	77.97
rs7716114	NA	NA	NA	0.006743932	3E-10	2	TRUE	237530	140254	0.00017	39.65
rs77463690	NA	NA	NA	0.011657379	0.000000002	2	TRUE	237530	140254	0.00015	35.98
rs77924615	NA	NA	NA	0.007734256	6.6E-17	2	TRUE	237530	140254	0.00029	69.78
rs7803355	NA	NA	NA	0.009447452	3.5E-11	2	TRUE	237530	140254	0.00018	43.87
rs78745308	NA	NA	NA	0.014506791	0.000000016	2	TRUE	237530	140254	0.00013	31.90
rs7938342	NA	NA	NA	0.006136446	4.5E-28	2	TRUE	237530	140254	0.00051	120.66
rs7940934	NA	NA	NA	0.006039219	0.000000027	2	TRUE	237530	140254	0.00013	30.93
rs79668541	NA	NA	NA	0.011364979	2.3E-16	2	TRUE	237530	140254	0.00028	67.35
rs8118848	NA	NA	NA	0.007107529	4.4E-17	2	TRUE	237530	140254	0.00030	70.61
rs880315	NA	NA	NA	0.006427611	7.4E-24	2	TRUE	237530	140254	0.00043	101.42
rs9506725	NA	NA	NA	0.006246842	4.6E-12	2	TRUE	237530	140254	0.00020	47.84
rs9683944	NA	NA	NA	0.007481672	4.1E-09	2	TRUE	237530	140254	0.00015	34.55
rs9844972	NA	NA	NA	0.01214377	0.000000008	2	TRUE	237530	140254	0.00014	33.28
rs989501	NA	NA	NA	0.006333831	0.000000022	2	TRUE	237530	140254	0.00013	31.29
rs9897348	A	T	C	0.006194936	2.2E-09	2	TRUE	237530	140254	0.00015	35.74
rs9907781	NA	NA	NA	0.007034687	3.4E-10	2	TRUE	237530	140254	0.00017	39.43
rs1333047	NA	NA	NA	0.019089253	4.7E-18	2	FALSE	242659	140254	0.0003089<br>87	75.001<br>10694

rs55730499	NA	NA	NA	0.034986724	1.4E-17	2	TRUE	242659	140254	0.0002998<br>69	72.787<br>16046

Table S16. Results of MR estimates and sensitivity analyses of eleven significant causal effects of medication use and cancer

Exposure	Outcome	Methods	Number of SNPs	*P*	OR 95% CI	Heterogeneity			Pleiotropy			Global Test *P* derived from MR-PRESSO	Power
						Q	Q_df	Q_*P*	egger_intercept	se	*P*		
Antithrombotic agents	Breast cancer	Inverse variance weighted	8	0.003	0.87(0.80-0.95)	6.49	7.00	0.484	-0.007	0.010	0.438	0.524	0.42
		MR Egger	8	0.690	0.95(0.76-1.20)								
		Weighted median	8	0.056	0.89(0.79-1.00)								
		Maximum likelihood	8	0.003	0.87(0.79-0.95)								
		Robust adjusted profile score	8	0.004	0.87(0.79-0.96)								
Anti-inflammatory and antirheumatic products, non-steroids	Breast cancer	Inverse variance weighted	3	0.040	1.25(1.00-1.55)	3.03	2.00	0.220	0.010	0.031	0.799	NA	0.25
		MR Egger	3	0.950	1.05(0.35-3.13)								
		Weighted median	3	0.330	1.12(0.89-1.42)								
		Maximum likelihood	3	0.016	1.26(1.04-1.52)								
		Robust adjusted profile score	3	0.040	1.25(1.01-1.55)								
Antihistamines for systemic use	Breast cancer	Inverse variance weighted	6	0.002	1.11(1.04-1.18)	4.28	5.00	0.510	-0.007	0.020	0.662	0.543	
		MR Egger	6	0.339	1.19(0.87-1.65)								
		Weighted median	6	0.016	1.11(1.02-1.21)								

		Maximum likelihood	6	0.002	1.11(1.04-1.19)								
		Robust adjusted profile score	6	0.003	1.11(1.03-1.19)								
Antithrombotic agents	Lung cancer	Inverse variance weighted	10	0.0003	0.67(0.54-0.83)	5.07	9.00	0.828	0.020	0.021	0.372	0.8314	0.50
		MR Egger	10	0.051	0.52(0.30-0.91)								
		Weighted median	10	0.013	0.70(0.52-0.93)								
		Maximum likelihood	10	0.0003	0.67(0.54-0.83)								
		Robust adjusted profile score	10	0.0005	0.67(0.53-0.84)								
Antihypertensives	Lung cancer	Inverse variance weighted	4	0.007	0.80(0.68-0.94)	0.19	3.00	0.980	0.070	0.270	0.819	0.988	0.08
		MR Egger	4	0.759	0.42(0.003-52.61)								
		Weighted median	4	0.013	0.80(0.66-0.95)								
		Maximum likelihood	4	0.008	0.80(0.67-0.94)								
		Robust adjusted profile score	4	0.010	0.80(0.67-0.95)								
HMG CoA reductase inhibitors	Lung cancer	Inverse variance weighted	60	0.026	0.91(0.84-0.99)	60.11	59.00	0.435	-0.0006	0.010	0.903	0.472	0.2
		MR Egger	60	0.279	0.92(0.79-1.07)								
		Weighted	60	0.112	0.90(0.79-1.03)								

		median											
		Maximum likelihood	60	0.024	0.91(0.84-0.99)								
		Robust adjusted profile score	60	0.04	0.91(0.84-0.996)								
Thyroid preparations	Lung cancer	Inverse variance weighted	87	0.00006	0.90(0.86-0.95)	70.55	86.00	0.886	-0.0002	0.005	0.972	0.883	0.23
		MR Egger	87	0.105	0.91(0.81-1.02)								
		Weighted median	87	0.001	0.87(0.80-0.95)								
		Maximum likelihood	87	6.00E-05	0.90(0.86-0.95)								
		Robust adjusted profile score	87	5.00E-05	0.90(0.86-0.95)								
Salicylic acid and derivatives	Lung cancer	Inverse variance weighted	8	0.018	0.75(0.59-0.95)	7.78	7.00	0.353	0.001	0.030	0.745	0.356	0.28
		MR Egger	8	0.322	0.67(0.32-1.39)								
		Weighted median	8	0.172	0.81(0.59-1.10)								
		Maximum likelihood	8	0.013	0.75(0.60-0.94)								
		Robust adjusted profile score	8	0.015	0.73(0.57-0.94)								
Anti-inflammatory and antirheumatic products,	Colorectal cacncer	Inverse variance weighted	6	0.0001	0.53(0.38-0.73)	4.37	5.00	0.498	0.033	0.036	0.411	0.542	0.59
		MR Egger	6	0.154	0.27(0.06-1.17)								
		Weighted	6	0.001	0.50(0.33-0.76)								

non-steroids		median											
		Maximum likelihood	6	0.0002	0.52(0.37-0.73)								
		Robust adjusted profile score	6	0.0002	0.51(0.36-0.73)								
Agents acting on the renin-angiotensin system	Prostate cancer	Inverse variance weighted	105	0.0007	0.94(0.91-0.97)	104.24	104.00	0.475	-0.0008	0.003	0.773	0.492	0.42
		MR Egger	105	0.372	0.95(0.86-1.06)								
		Weighted median	105	0.351	0.97(0.92-1.03)								
		Maximum likelihood	105	0.0008	0.94(0.91-0.97)								
		Robust adjusted profile score	105	0.002	0.94(0.90-0.98)								
Vasodilators used in cardiac diseases	Prostate cancer	Wald ratio	1	2.48E-05	1.25(1.13-1.38)	NA	NA	NA	NA	NA	NA	NA	0.11

Table S17. The proportion of variance explained for the instrumental variables and the statistical power of the primary MR analysis

Exposure	Outcome	PVE	OR=0.1 Power	OR=0.3 Power	OR=0.5 Power	OR=0.7 Power	OR=0.9 Power
Antithrombotic agents	Breast cancer	0.27%	100%	100%	100%	99%	26%
Anti-inflammatory and antirheumatic products, non-steroids	Breast cancer	0.06%	100%	100%	99%	57%	9%
Antithrombotic agents	Lung cancer	0.38%	100%	100%	92%	41%	8%
Antihypertensives	Lung cancer	0.08%	100%	74%	33%	13%	6%
HMG CoA reductase inhibitors	Lung cancer	2.10%	100%	100%	100%	98%	23%
Thyroid preparations	Lung cancer	2.04%	100%	100%	100%	98%	23%
Salicylic acid and derivatives	Lung cancer	0.36%	100%	100%	90%	40%	8%
Anti-inflammatory and antirheumatic products, non-steroids	Colorectal cacncer	0.13%	100%	100%	68%	22%	6%
Agents acting on the renin-angiotensin system	Prostate cancer	2.34%	100%	100%	100%	100%	85%
Vasodilators used in cardiac diseases	Prostate cancer	0.03%	100%	100%	67%	22%	6%

Table S18. Heritability and genetic correlation between medication use and cancer

Exposure	Outcome	Heritability							
		h2_exposure	h2_exposure_se	intercept_exposure	ration_exposure	h2_outcome	h2_outcome_se	intercept_outcome	ration_outcome
Antithrombotic agents	Lung cancer	0.051868936	0.004200424	1.020626337	0.1151	0.108527604	0.020179768	1.010599329	0.1541
Antihypertensives	Lung cancer	0.021081313	0.003618538	1.020837335	0.2461	0.108527604	0.020179768	1.010599329	0.1541
Thyroid preparations	Lung cancer	0.066727312	0.006557222	1.045707168	0.0966	0.108527604	0.020179768	1.010599329	0.5902
Antiinflammatory and antirheumatic products, non-steroids	Colorectal cancer	0.129669108	0.006375339	1.087766614	0.1223	0.059586119	0.017679611	1.055087272	0.1674
Agents acting on the renin-angiotensin system	Prostate cancer	0.129669108	0.006375339	1.087766614	0.1223	0.133935458	0.013973424	1.074693142	0.1674
Vasodilators used in cardiac diseases	Prostate cancer	0.013987632	0.002179535	1.020410397	0.2347	0.133935458	0.013973424	1.074693142	0.1674

Table S18. Heritability and genetic correlation between medication use and cancer (continued)

Exposure	Outcome	Genetic Correlation			
		rgcov_se	rg	rg_se	*P*
Antithrombotic agents	Lung cancer	0.005601816	0.267028772	0.0747	0.00034828
Antihypertensives	Lung cancer	0.005509116	0.217074167	0.1152	0.059469
Thyroid preparations	Lung cancer	0.006133764	-0.133955524	0.0721	0.063102
Antiinflammatory and antirheumatic products, non-steroids	Colorectal cancer	0.005989785	0.046207333	0.0681	0.49771
Agents acting on the renin-angiotensin system	Prostate cancer	0.004081956	0.018408437	0.031	0.5523
Vasodilators used in cardiac diseases	Prostate cancer	0.002767437	-0.008111789	0.0639	0.89904

Table S19. Reverse MR analysis from cancer on medication use

Exposure	Outcome	Methods	Number of SNPs	*P*	OR 95% CI	Heterogeneity			Pleiotropy			Global Test *P* derived from MR-PRESSO
						Q	Q_ df	Q_*P*	egger_in tercept	se	*P*	
Lung cancer	Antithrombotic agents	Inverse variance weighted	4	0.377	0.98(0.92-1.03)	8.07	3	0.045	-0.033	0.027	0.356	0.147
		MR Egger	4	0.448	1.11(0.89-1.38)							
		Weighted median	4	0.418	0.98(0.94-1.02)							
		Maximum likelihood	4	0.143	0.97(0.94-1.01)							
		Robust adjusted profile score	4	0.409	0.98(0.93-1.03)							
Lung cancer	Antihypertensives	Inverse variance weighted	4	0.301	0.95(0.87-1.04)	0.68	3.00	0.877	-0.015	0.047	0.778	0.908
		MR Egger	4	0.951	1.01(0.69-1.48)							
		Weighted median	4	0.309	0.95(0.86-1.05)							
		Maximum likelihood	4	0.301	0.95(0.87-1.04)							
		Robust adjusted profile score	4	0.315	0.95(0.87-1.05)							
Lung cancer	Thyroid preparations	Inverse variance weighted	3	0.434	1.03(0.96-1.10)	4.12	2	0.128	-0.055	0.028	0.299	NA
		MR Egger	3	0.274	1.27(1.02-1.57)							
		Weighted median	3	0.083	1.05(0.999-1.11)							
		Maximum likelihood	3	0.260	1.03(0.98-1.08)							
		Robust adjusted profile score	3	0.370	1.03(0.97-1.10)							
Colorectal cancer	Antiinflammatory and antirheumatic products, non-steroids	Inverse variance weighted	12	0.279	1.02(0.98-1.06)	13.30	11	0.274	-0.006	0.012	0.633	0.268
		MR Egger	12	0.483	1.06(0.90-1.25)							
		Weighted median	12	0.293	1.03(0.98-1.77)							
		Maximum likelihood	12	0.226	1.02(0.99-1.06)							
		Robust adjusted profile score	12	0.194	1.03(0.99-1.07)							
Prostate cancer	Agents acting on the renin-angiotensin system	Inverse variance weighted	81	0.445	1.01(0.99-1.02)	76.12	80	0.602	0.001	0.002	0.476	0.606
		MR Egger	81	0.850	1.00(0.97-1.03)							
		Weighted median	81	0.256	0.99(0.96-1.01)							
		Maximum likelihood	81	0.445	1.01(0.99-1.02)							
		Robust adjusted profile score	81	0.528	1.01(0.99-1.02)							
Prostate cancer	Vasodilators used in cardiac	Inverse variance weighted	100	0.979	1.00(0.96-1.05)	73.97	99	0.972	0.005	0.004	0.230	0.975

diseases				
	MR Egger	100	0.317	0.96(0.88-1.04)
	Weighted median	100	0.750	0.99(0.92-1.06)
	Maximum likelihood	100	0.979	1.00(0.96-1.05)
	Robust adjusted profile score	100	0.967	1.00(0.95-1.05)

Table S20. Steiger direction test from medication use to cancer

Exposure	Outcome	SNP_r2.exposure	SNP_r2.outcome	Correct_causal_direction	Steiger *P*
Antithrombotic agents	Lung cancer	0.0039	0.0005	TRUE	2.33E-09
Antihypertensives	Lung cancer	0.0009	0.0003	TRUE	0.0379
Thyroid preparations	Lung cancer	0.0206	0.0031	TRUE	2.05E-44
Antiinflammatory and antirheumatic products, non-steroids	Colorectal cancer	0.0014	0.0006	TRUE	0.0358
Agents acting on the renin-angiotensin system	Prostate cancer	0.0240	0.0008	TRUE	0.0000
Vasodilators used in cardiac diseases	Prostate cancer	0.0006	0.0001	TRUE	0.0001

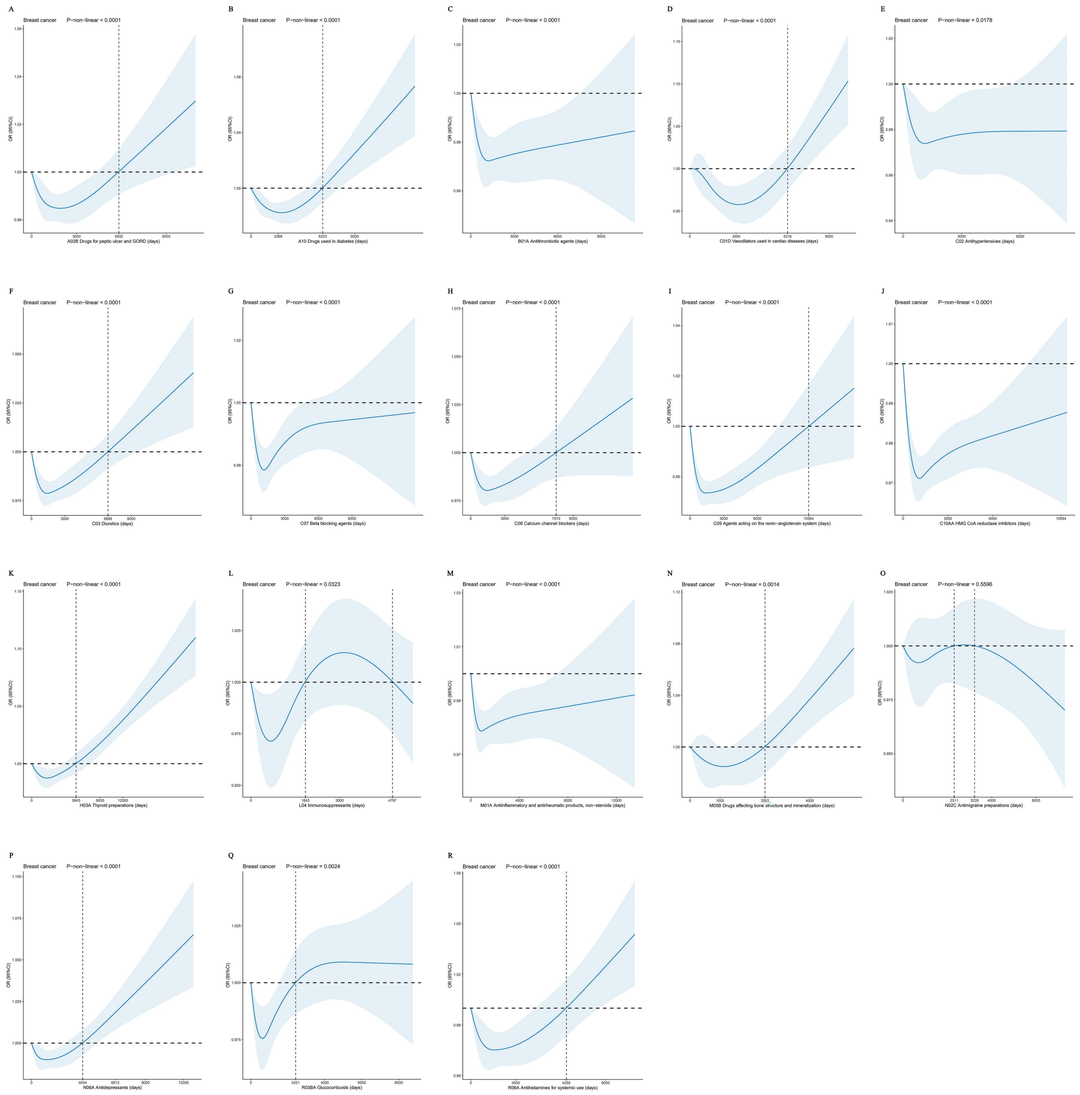

Figure S1. Analysis of the shape of the non-linear association between the duration of drug use and breast cancer using restricted cubic spline based on the NHANES database.

The solid blue line is the estimated odds ratio, and the shaded blue area is the 95% confidence interval.

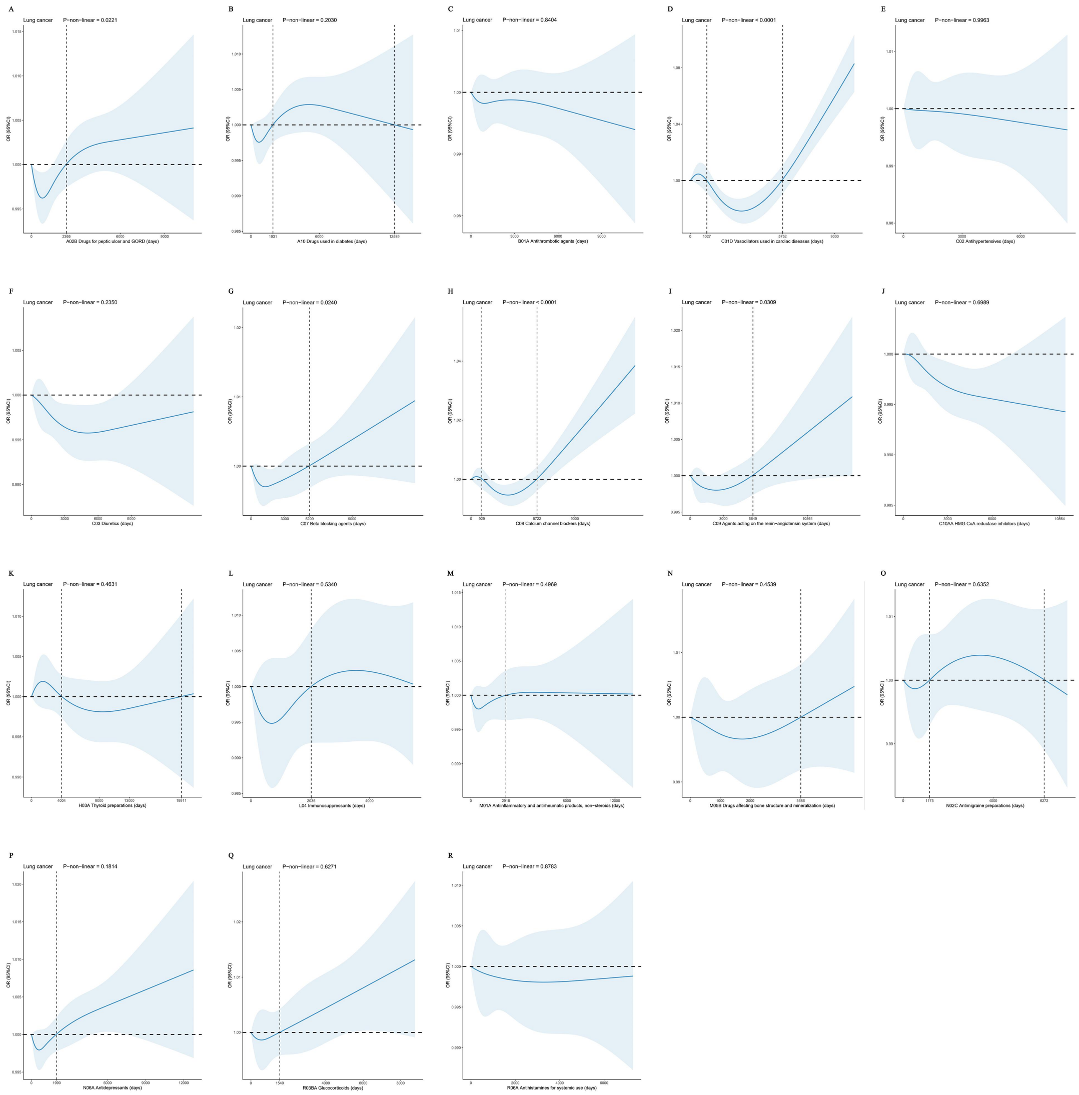

Figure S2. Analysis of the shape of the non-linear association between the duration of drug use and lung cancer using restricted cubic spline based on the NHANES database.

The solid blue line is the estimated odds ratio, and the shaded blue area is the 95% confidence interval.

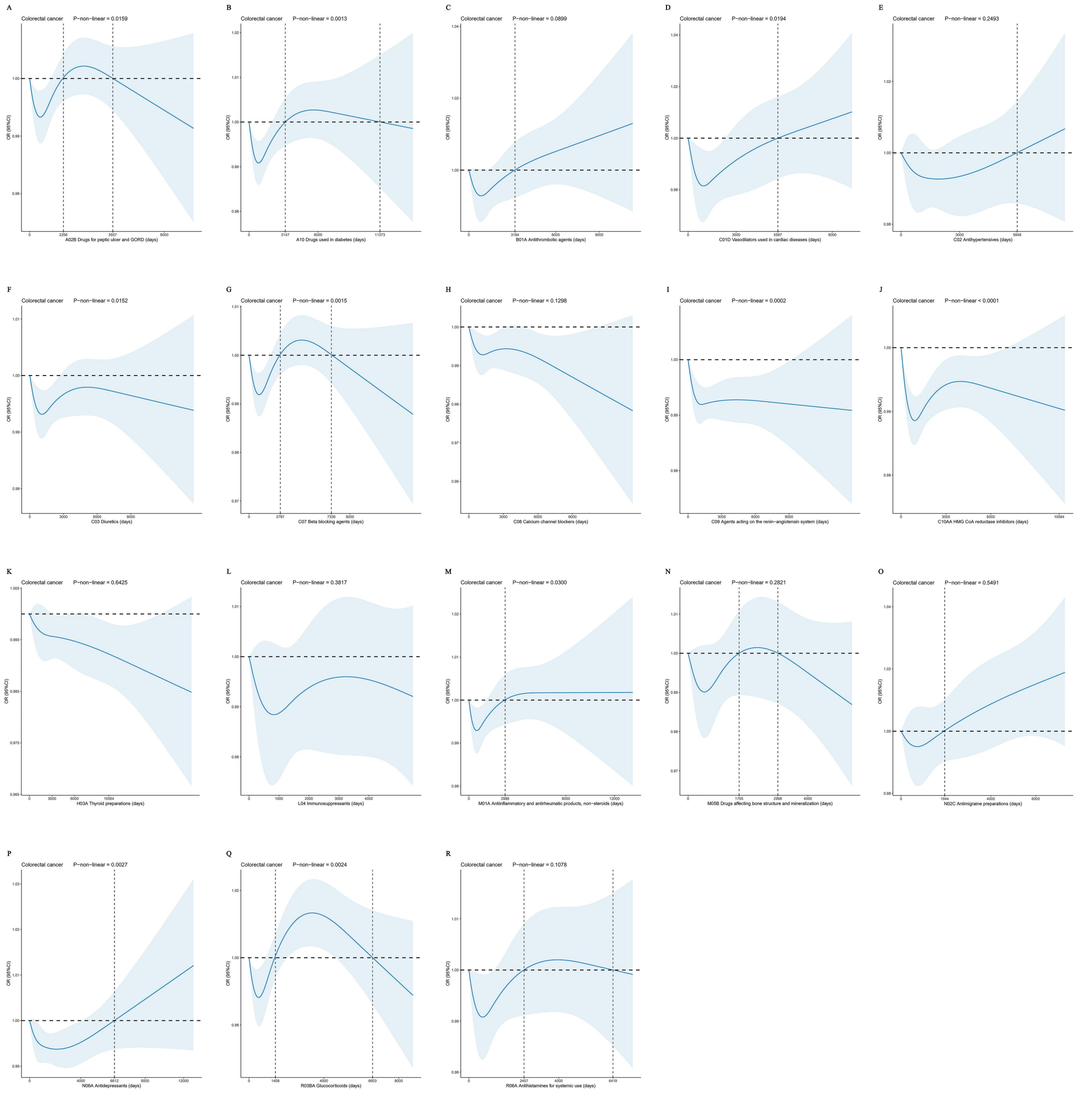

Figure S3. Analysis of the shape of the non-linear association between duration of drug use and colorectal cancer using restricted cubic spline based on the NHANES database.

The solid blue line is the estimated odds ratio, and the shaded blue area is the 95% confidence interval.

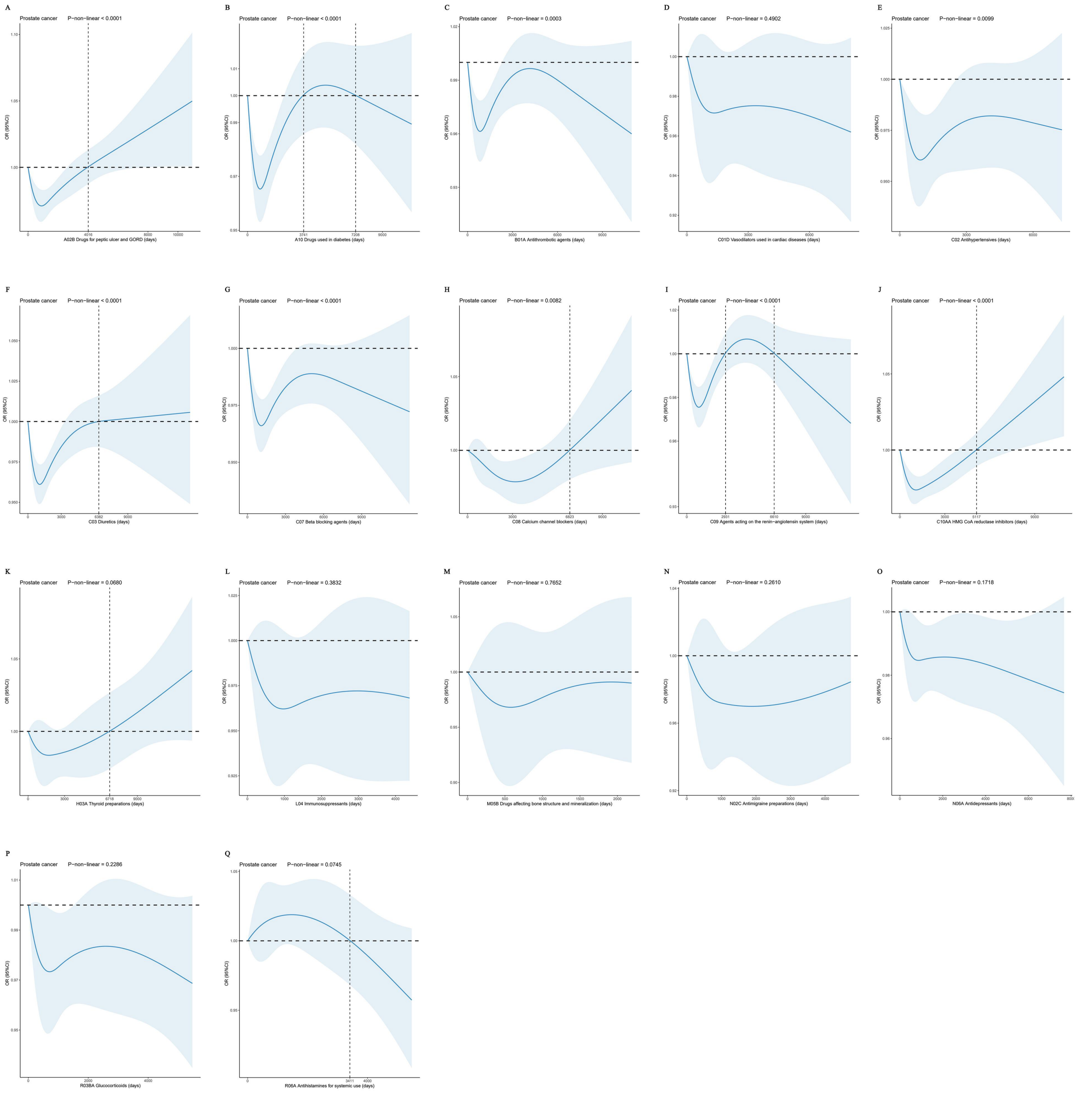

Figure S4. Analysis of the shape of the non-linear association between duration of drug use and prostate cancer using restricted cubic spline based on the NHANES database.

The solid blue line is the estimated odds ratio, and the shaded blue area is the 95% confidence interval.

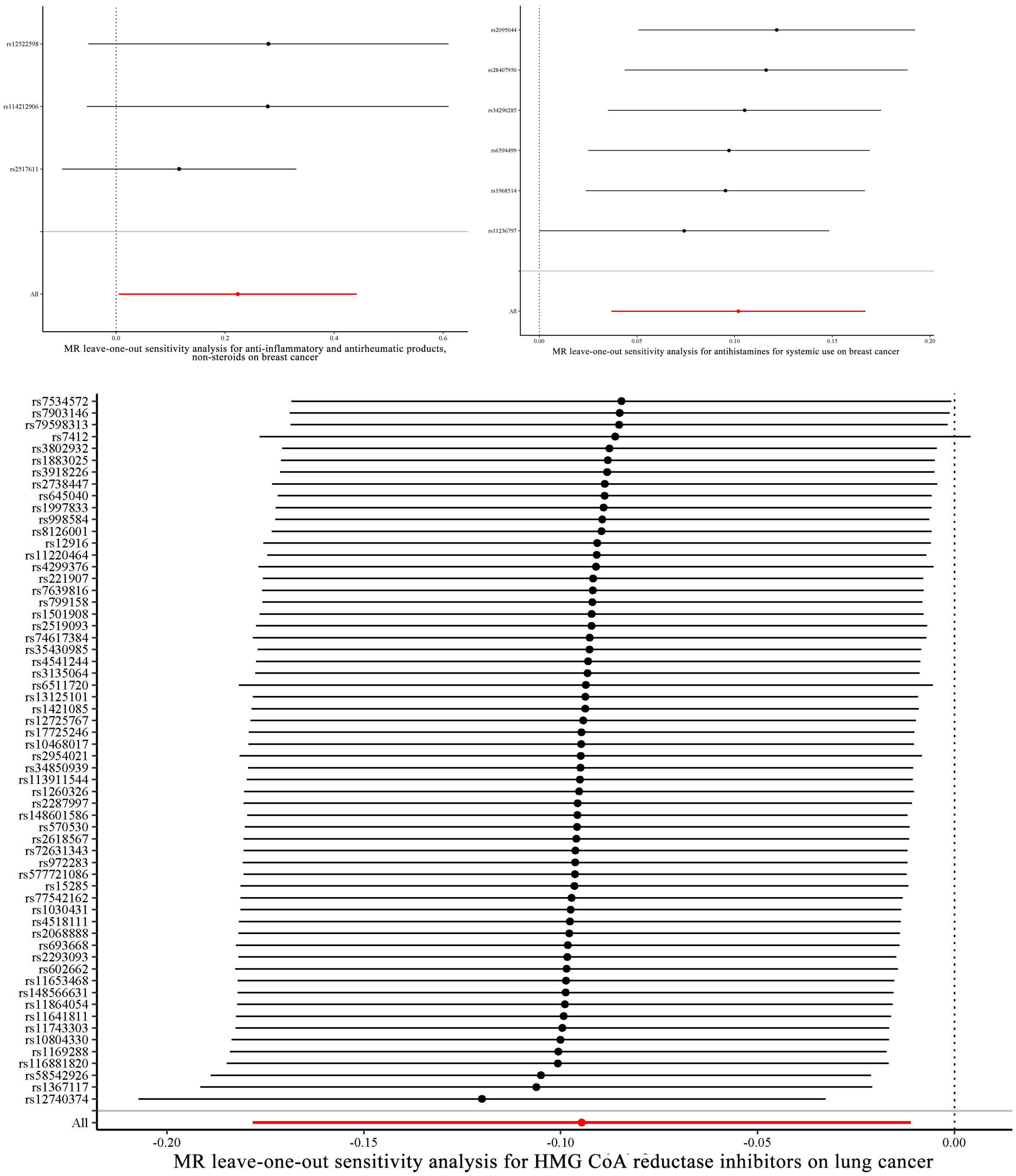

Figure S5. Unrobust leave one out analysis in significant associations between genetically determined medication use and cancer.

Black dots indicated the IVW estimates (raw beta) after leaving a single SNP in turns. Red dots indicate the pooled IVW estimate (raw beta). Horizontal lines indicated the range of a 95% confidence interval. MR, Mendelian randomization.

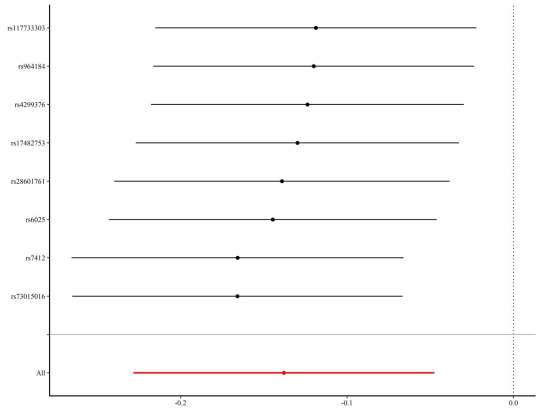

MR leave-one-out sensitivity analysis for antithrombotic agents on breast cancer

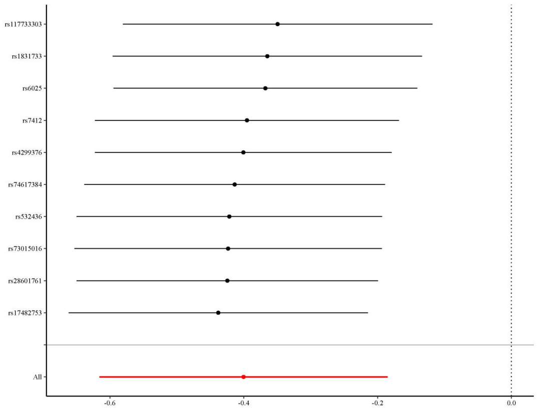

MR leave-one-out sensitivity analysis for antithrombotic agents on lung cancer

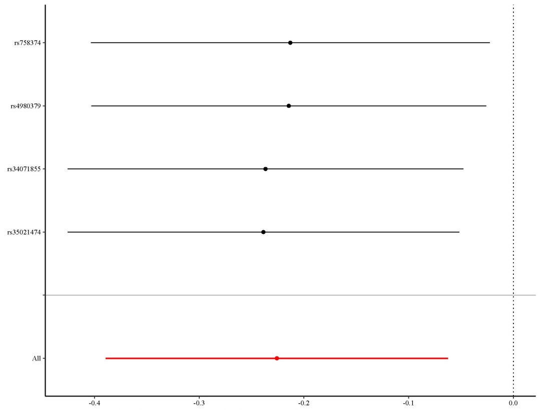

MR leave-one-out sensitivity analysis for antihypertensives on lung cancer

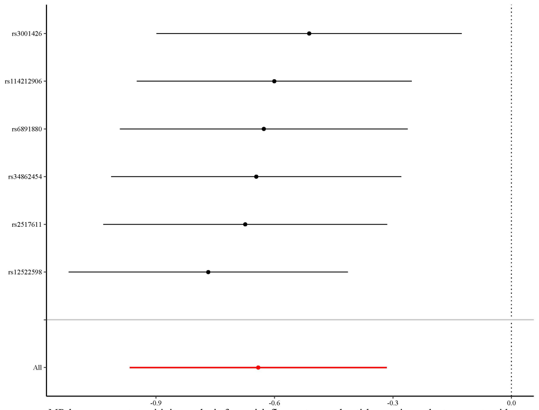

MR leave-one-out sensitivity analysis for anti-inflammatory and antirheumatic products, non-steroids on colorectal cancer

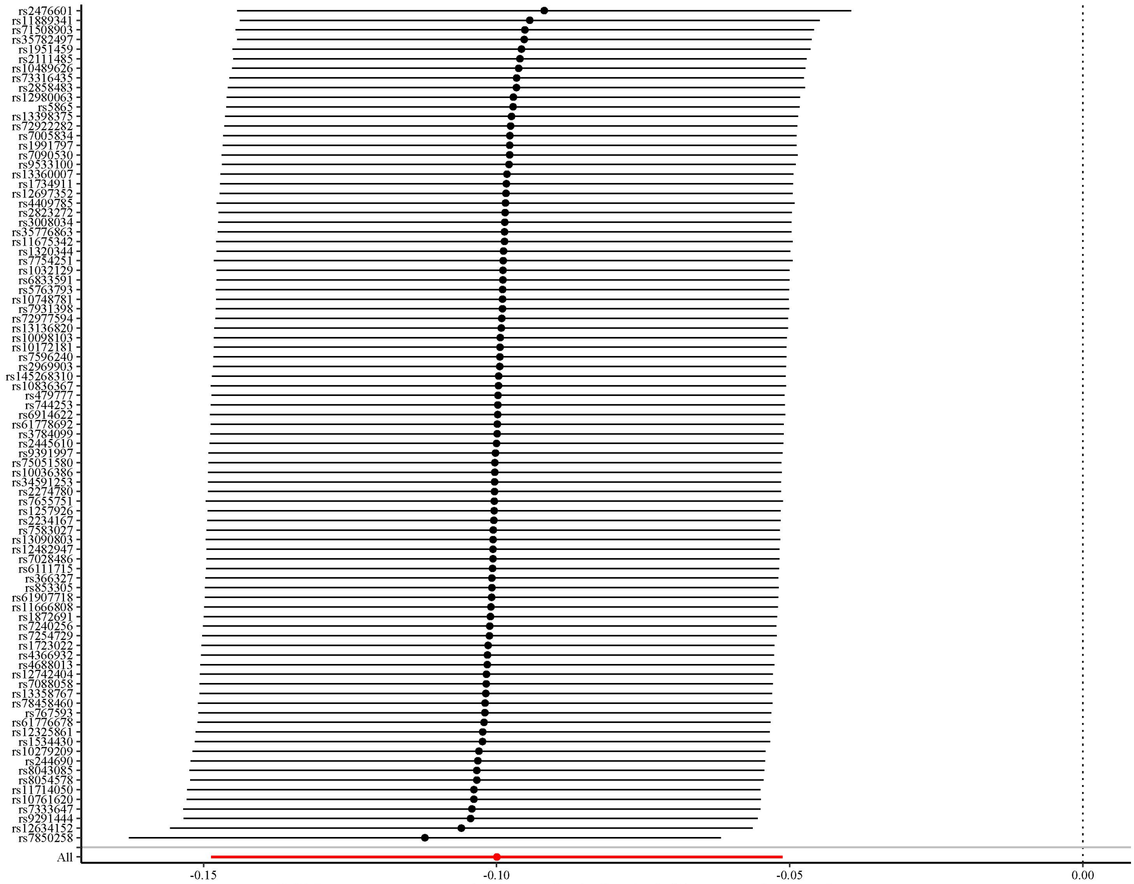

MR leave-one-out sensitivity analysis for thyroid preparations on lung cancer

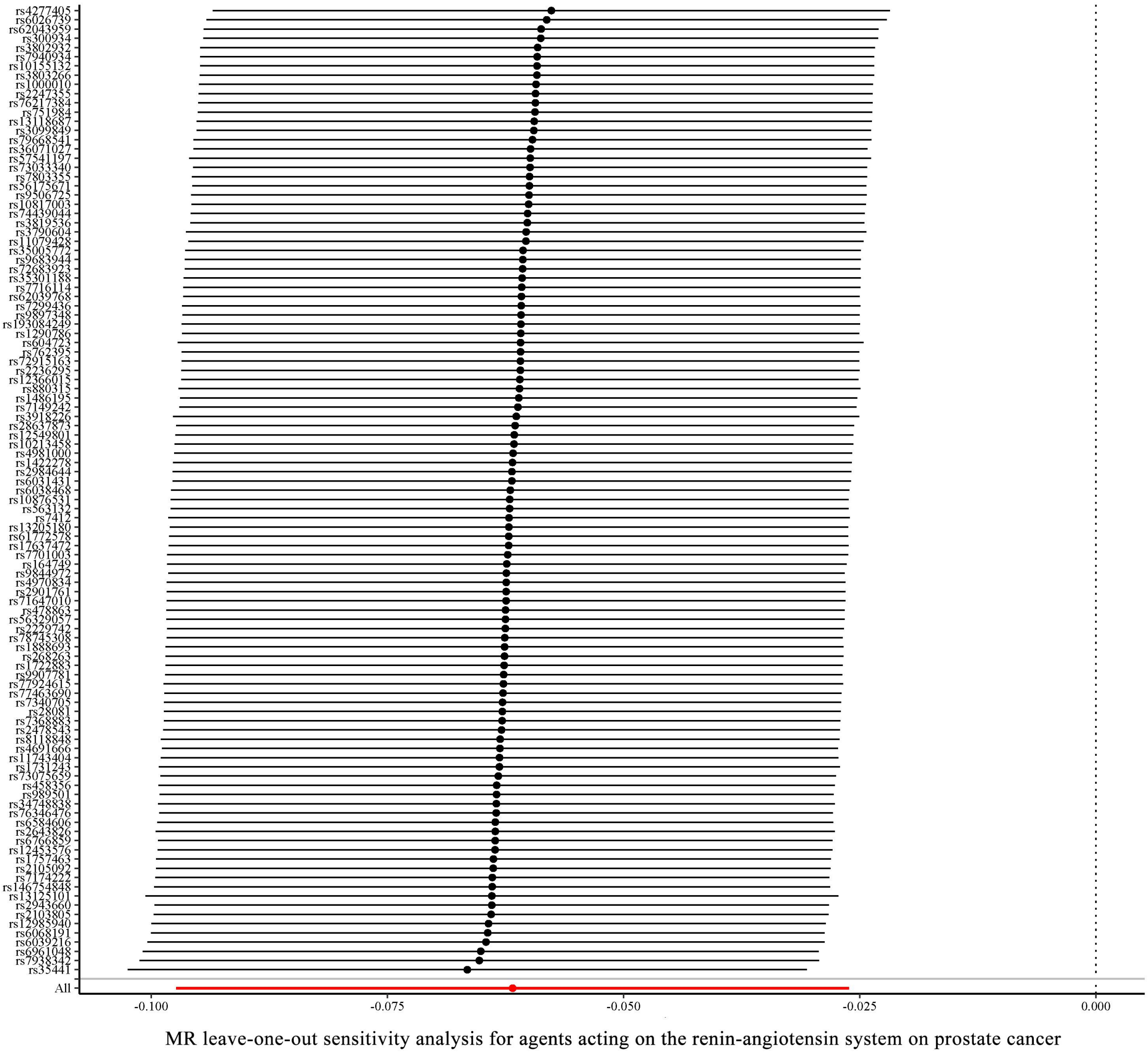

Figure S6. Robust leave-one-out sensitivity analyses in significant associations between genetically determined medication use and cancer.

Black dots indicated the IVW estimates (raw beta) after leaving a single SNP in turns. Red dots indicate the pooled IVW estimate (raw beta). Horizontal lines indicated the range of a 95% confidence interval. MR, Mendelian randomization.

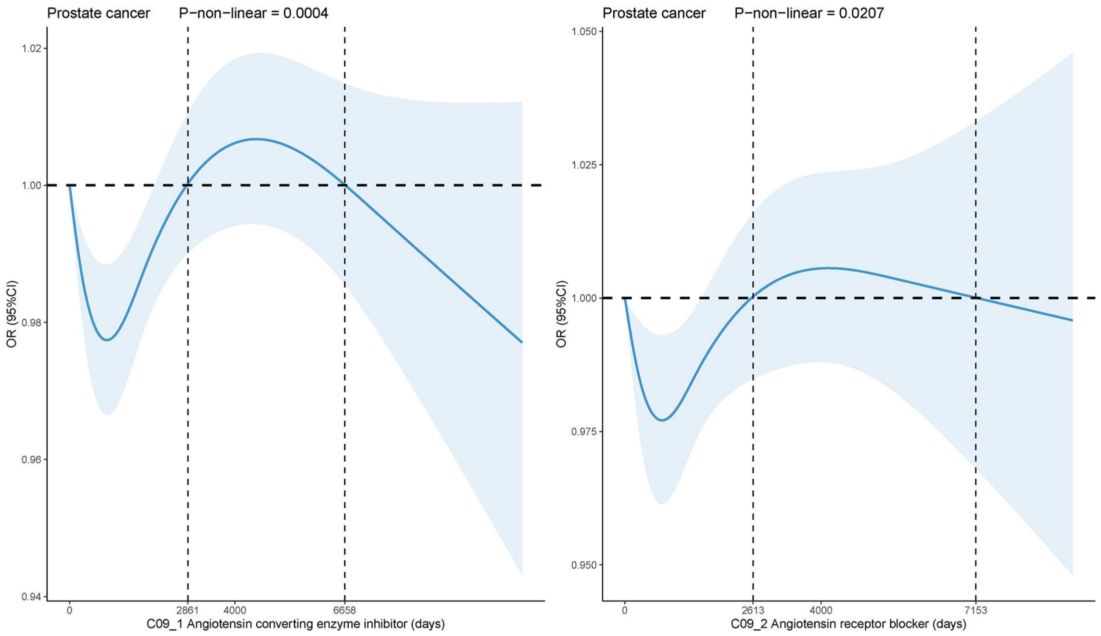

Figure S7. Analysis of the shape of the non-linear association between duration of angiotensin-converting enzyme inhibitor and angiotensin receptor blocker and prostate cancer using the restricted cubic spline model based on the NHANES database.
